# Genomic and experimental data provide new insights into luciferin biosynthesis and bioluminescence evolution in fireflies

**DOI:** 10.1038/s41598-020-72900-z

**Published:** 2020-09-28

**Authors:** Ru Zhang, Jinwu He, Zhiwei Dong, Guichun Liu, Yuan Yin, Xinying Zhang, Qi Li, Yandong Ren, Yongzhi Yang, Wei Liu, Xianqing Chen, Wenhao Xia, Kang Duan, Fei Hao, Zeshan Lin, Jie Yang, Zhou Chang, Ruoping Zhao, Wenting Wan, Sihan Lu, Yanqiong Peng, Siqin Ge, Wen Wang, Xueyan Li

**Affiliations:** 1 School of Ecology and Environment, Northwestern Polytechnical University, Xi’an, 710072 Shaanxi China; 2 State Key Laboratory of Genetic Resources and Evolution, Kunming Institute of Zoology, Chinese Academy of Sciences, Kunming, 650223 Yunnan China; 3 Institute of Zoology, Chinese Academy of Sciences, Beijing, China; 4 CAS Key Laboratory of Tropical Forest Ecology, Xishuangbanna Tropical Botanical Garden, Chinese Academy of Sciences, Mengla, 666303 Yunnan China; 5 Center for Excellence in Animal Evolution and Genetics, Kunming, 650223 Yunnan China

**Keywords:** Biotechnology, Evolution, Genetics, Molecular biology

## Abstract

Fireflies are among the most charismatic insects for their spectacular bioluminescence, but the origin and evolution of bioluminescence remain elusive. Especially, the genic basis of luciferin (d-luciferin) biosynthesis and light patterns is largely unknown. Here, we present the high-quality reference genomes of two fireflies *Lamprigera yunnana* (1053 Mb) and *Abscondita terminalis* (501 Mb) with great differences in both morphology and luminous behavior. We sequenced the transcriptomes and proteomes of luminous organs of two species. We created the CRISPR/Cas9-induced mutants of *Abdominal B* gene without luminous organs in the larvae of *A. terminalis* and sequenced the transcriptomes of mutants and wild-types. Combining gene expression analyses with comparative genomics, we propose a more complete luciferin synthesis pathway, and confirm the convergent evolution of bioluminescence in insects. Using experiments, the function of the firefly acyl-CoA thioesterase (ACOT1) to convert l-luciferin to d-luciferin was validated for the first time. Comparisons of three-dimension reconstruction of luminous organs and their differentially expressed genes among two species suggest that two positive genes in the calcium signaling pathway and structural difference of luminous organs may play an important role in the evolution of flash pattern. Altogether, our results provide important resources for further exploring bioluminescence in insects.

## Introduction

Bioluminescence is a particularly intriguing phenomenon^1^, and its origin and evolution fascinate biologists since the time of Charles Darwin^2^. Fireflies (Lampyridae) are one of the best-known luminescent organisms since the time of Aristotle^1^, and thus an important subject of scientific studies, especially related to their bioluminescent behavior and biochemistry. Together with other luminous beetles (Rhagophthalmidae, Phengodidae and some Elateridae) in the same superfamily Elateroidea^1^, fireflies can produce light in the peroxisome of photocytes within the luminous organs with diverse morphology and location^3^ by a common oxidative mechanism of luciferin catalyzed by luciferases in the presence of ATP, O_2_ and Mg^2+^. Beetle luciferases have long been studied extensively in their sequence, structure and function^4,5^ and yielded numerous molecular, biomedical, pharmaceutical and bioanalytical applications^5^. Beetle luciferin, the substrate for bioluminescence reaction, appears to be conserved in structure among all luminous beetles, but is not found in non-luminous insects^6^, suggesting that its evolutionary origin may coincide with the origin of bioluminescence. Firefly bioluminescence first evolved as aposematic warning signal in larvae (glow)^7^ and later was co-opted as sexual signal in adults (glow, flash)^7,8^. Light on/off is controlled by the accessibility of O_2_ to peroxisome in photocytes, which is regulated by oxygen nitrogen (NO) synthesis in tracheolar end cells induced by octopamine released from neural system through G-protein coupled receptor cAMP/PKA-Ca/Calmodulin signaling cascade^9–11^. So far, the reference genomes of four luminous beetles (Lamyridae (fireflies): 3; Elateridae (click beetles): 1) were reported in two separated articles^12,13^. In one of them, the comparative genomics of two fireflies and one luminous click beetle support parallel origin of bioluminescence in beetles^13^. However, the origin and evolution of luciferase genes and how bioluminescent light signal pattern (glow, flash) evolve in luminous beetles remain elusive. Most seriously, the genic basis of luciferin biosynthesis is largely unknown.

**Figure 1:**
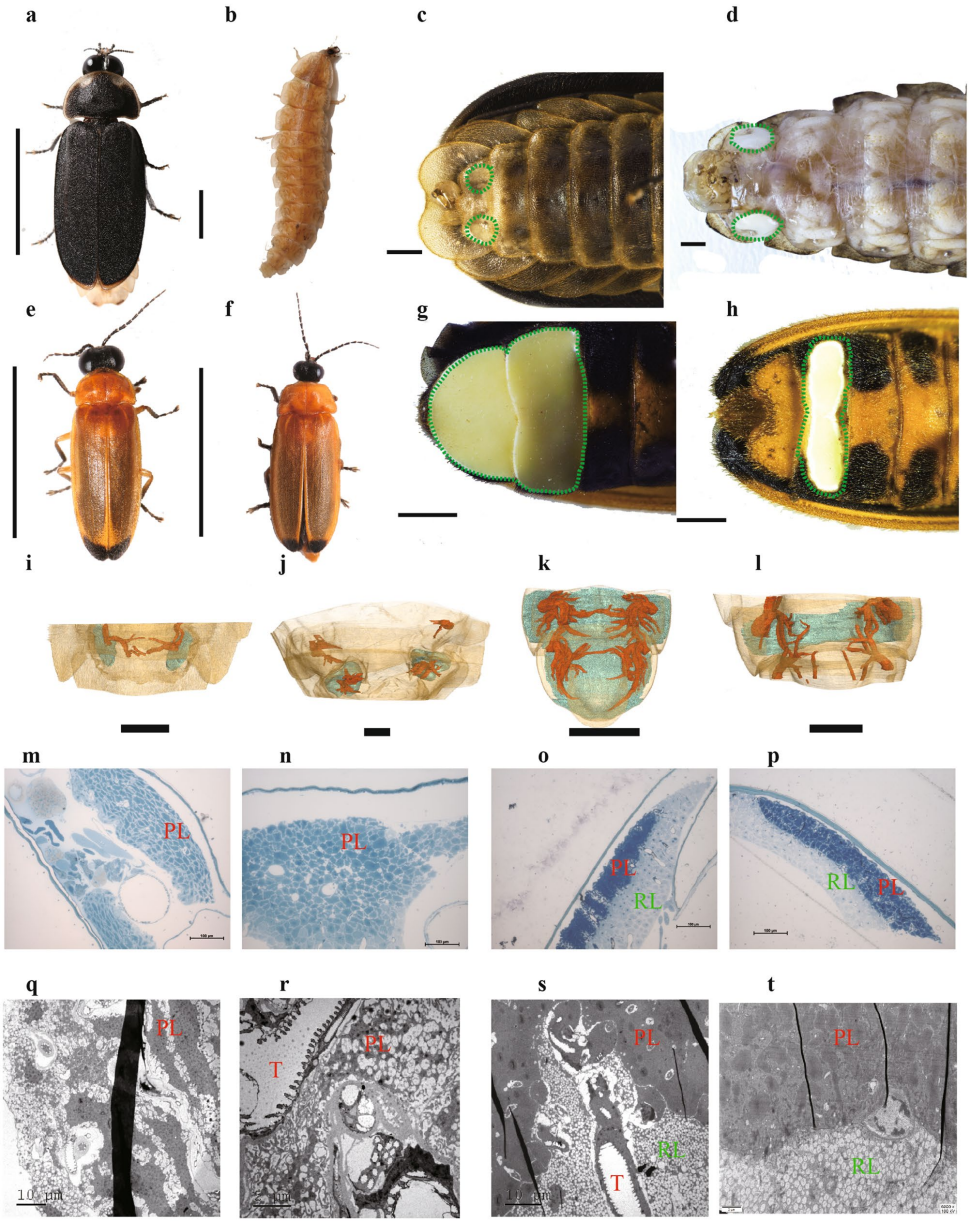
Adults of *Lamprigera yunnana* and *Abscodita terminalis* and their luminous organs. (**a**–**d**) Adults (a, male; b, female) and their luminous organs (dotted green regions) (**c**, male; **d**, female) of *L. yunnana*. (**e**–**h**) Adults (**e**, male; **f**, female) and their luminous organs (dotted green regions) (**g**, male; **h**, female) of *A. terminalis*. Scale bars (**a**–**h**): 1 mm. (**i**–**l**) Three-dimension reconstruction of luminous organs (blue) and tracheal system (red) of male and female *L. yunnana* (**i**, male; **j**, female) and *A. terminalis* (**k**, male; **l**, female). (**m**–**p**) Semi-thin section structures of luminous organs of male and female *L. yunnana* (**m**, male; **n**, female) and *A. terminalis* (**o**, male; **p**, female). (**q**–**t**) Transmission electron microscopic structures of luminous organs of male and female *L. yunnana* (**q**, male; **r**, female) and *A. terminalis* (**s**, male; **t**, female). PL, Photogenic layer; RL, Reflecting layer; T, Trachea.

*Lamprigera yunnana* (Lampyridae: *incertae sedis*) and *Abscondita terminalis* (Lampyridae: Luciolinae) display glow or flash signals, respectively, and also show great differences in both outer morphology and inner structure of their luminous organs (Fig. 1a–t; Supplementary Note 1, Figs. S1–S8, Videos S1–S4). Here, we present their high-quality reference genomes using single-molecule real-time (SMRT) sequencing technologies. A thorough investigation integrating multilevel data (including comparative genomics, proteomics and transcriptomics of luminous organs and their three-dimension reconstruction, functional verification of genes in vitro experiments, and CRISPR/Cas9 gene editing) provides new perspectives on luciferin biosynthesis, the origin and evolution of bioluminescence and light pattern.

## Results

### Genome sequencing, assembly and annotation

**Figure 2:**
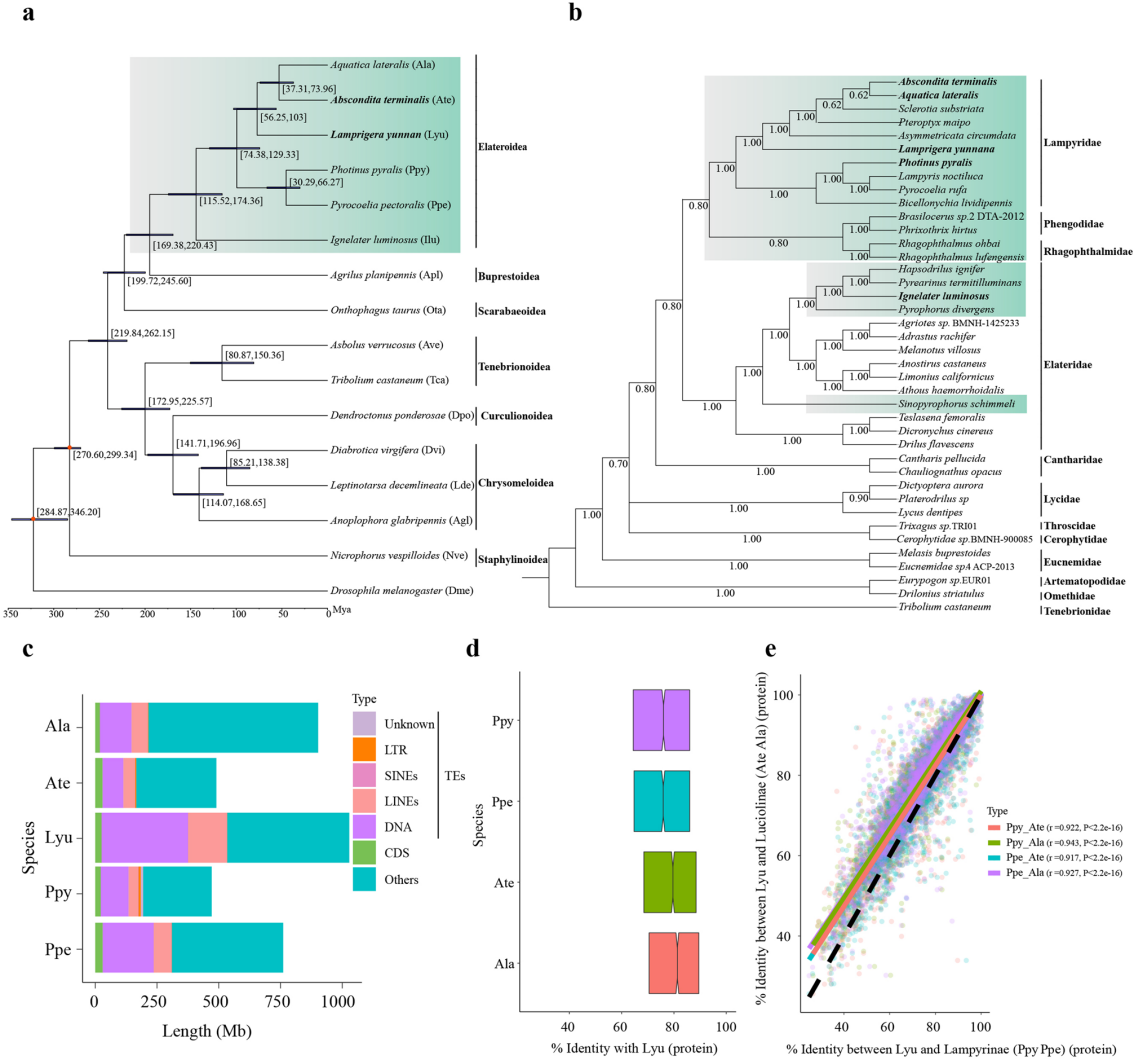
Phylogeny of luminous beetles. (**a**) Maximum likelihood tree inferred from 531 single-copy orthologous genes and estimated divergent times with fossil calibrations (red nodes) with *D. melanogaster* as an outgroup. (**b**) Bayesian tree of Elateroidea families inferred from 13 mitogenomic protein-coding genes with *Tribolium castaneum* as an outgroup. Luminous taxa in (**a**) and (**b**) highlighted in cyan. (**c**) Genomic composition (coding sequence (CDS), five types TEs (DNA, LINEs, SINEs, LTR and Unknown) and Others (the rest of the whole genome except CDS and TEs) of five fireflies. (**d**), (**e**) Amino acid (AA) sequence identities of 3,125 single-copy orthologous genes (SCGs) among five fireflies (**d**: average; **e**: each SCG (each dot)). Notches in (**d**) indicate the median value. Color lines and dashed line in (**e**) indicate the smoother of a locally weighted regression, and slope ratio of 1, respectively.

Using SMRT long reads, we assemble the high-quality reference genomes of two fireflies *L. yunnana* (1053 Mb; contig N50: 3.51 Mb) and *A. terminalis* (501 Mb; contig N50: 1.21 Mb) with high genome heterozygosity (Table 1; Supplementary Note 2, Tables S1–S4, Figs. S9–S10). Three evaluation methods (Illumina reads mapping, RNA reads mapping and Benchmarking Universal Single-Copy Orthologs (BUSCO)) show the completeness and reliability of the two assemblies (Supplementary Note 2, Tables S5–S7). The assembled sizes are consistent with those estimated by *k*-mer analyses (Supplementary Table S2) and flow-cytometry^14^. Among the assembled genomes of six phylogenetically related luminous beetles including four previously reported (three fireflies and one click beetle)^12,13^ and two fireflies sequenced in this study (Fig. 2a,b), *L. yunnana* genome has the largest size (1053 Mb) and the highest percentage of repetitive elements (66.62%)**,** while those of *A. terminalis* (501 Mb; 36.54%) are similar to those of American firefly *Photinus pyralis* (471 Mb; 47.70%) (Table 1; Supplementary Tables S8–S9). Comparative analyses of whole genomes among five fireflies (three previously reported^12,13^ and two sequenced here) indicate that genome size variation mainly results from the relative abundance of transposable elements (TEs), especially DNA transposons and long interspersed nuclear elements (LINEs), which are also two most abundant types of TEs among the genomes of all luminous beetles previously reported^12,13^ and sequenced in this study, and correlate in abundance with their host genome size (Table 1, Fig. 2c; Supplementary Table S9, Fig. S11). Combining de novo, homology-based and transcriptome-based methods, we predicted 19,443 and 21,024 genes in *L. yunnana* and *A. terminalis*, respectively (Supplementary Tables S10–S13). The gene structure features are similar to those of other fireflies (Supplementary Tables S11–S12, Fig. S12).

**Table 1:** Comparison of reference genomes among beetles.

Family	Species	Assembled size (Mb)	GC content (%)	N50 (Mb)	Repeat content (%)	DNA transposons (%)	Gene number	Completeness of BUSCO (%)	References
Lampyridae	*Lamprigera yunnana*	1053	34.12	3.43*	66.62	34.91	19,443	98.67	This study
	*Abscondita terminalis*	501	31.39	1.18*	35.54	17.62	21,024	97.83	This study
	*Aquatica lateralis*	902	29.60^§^	0.69^&^	27.86^§^	14.84^§^	14,285	97.40	^13^
	*Photinus pyralis*	471	36.41^§^	50.60^†^	47.70^§^	25.08^§^	15,773	97.20	^13^
	*Pyrocoelia pectoralis*	760	34.79	3.04*	47.21^§^	28.44^§^	23,092	98.73	^12^
Elateridae	*Ignelater luminosus*	845	33.42^§^	0.12^&^	34.10	8.45	27,557	94.80	^13^
Buprestidae	*Agrilus planipennis*	353	35.99	1.11^&§^	36.60^§^	3.20^§^	22,146	95.05^§^	GenBank
Scarabaeidae	*Onthophagus taurus*	267	33.40	0.33^&§^	48.08^§^	14.29^§^	21,668	99.46^§^	GenBank
Cerambycidae	*Anoplophora glabripennis*	710	33.39	0.66^&^	53.80^§^	6.84^§^	22,035	99.10^§^	^76^
Curculionidae	*Dendroctonus ponderosae*	252	36.00	0.62^&^	23.00	0.31	13,088	95.35^§^	^77^
Tenebrionidae	*Tribolium castaneum*	166	35.19	15.26^†§^/4.75^&^	27.50	2.20	16,593	99.33^§^	^78^

*Contig

†Chromosome and unplaced scaffolds

^&^Scaffold

^§^Predicted in this study using the same pipeline as in *L. yunnana* and *A. terminalis*. BUSCO: the Benchmarking Universal Single-Copy Ortholog.

### Firefly phylogeny based on phylogenomic data

We performed the phylogenomic analyses based on the 531 single-copy orthologous genes of Elateroidea and non-Elateroidea beetles plus fruit fly as an outgroup (Fig. 2a; Supplementary Note 4). Our results demonstrate that all six currently investigated luminous beetles (Lampyridae (5) and Elateridae (1) in the families of Elateroidea^15^) formed a clade (100% support), and diverged from non-Elateroidea beetles about 220-169 million years ago (Mya) (Fig. 2a), consistent with a previously reported phylogeny^13^ and the estimated divergent time of Elateroidea (182-152 Mya)^16^. Considering that there are still no reference genomes available for other non-luminous families in Elateroidea, we constructed an additional mitogenomic phylogeny for 11 Elateroidea families (including luminous and non-luminous families) (Supplementary Table S14) to explore the phylogenetic distribution of bioluminescent within Elateroidea taxa. Our results (Fig. 2b) indicate that Lampyridae, together with other luminous families (Asian Rhagophthalmidae and South American Phengodidae), is a sister clade to world-wide Elateridae, a family with only some luminous species mainly in South America but recently also found by us in Asia^17^. They corroborate with the phylogenies inferred from 95 nuclear protein-coding genes of beetles^18^ and from 13 protein-coding genes of mitogenomes and two nuclear ribosomal DNA (rDNA) (*18S*, *28S*)^19^, and with the beetle tree^16^ but differ from the phylogenies inferred from mitochondrial genes (*16S*, *COI*) and two nuclear rDNA (*18S*, *28S*)^15^. Although the so far reported phylogenies among Elateroidea families are still disputable, our data, together with previous findings^13,15^, demonstrate a dispersedly phylogenetic distribution of bioluminescence in Elateroidea and even within Elateridae, suggesting a phenotypically convergent evolution of bioluminescence within beetles, as noted by Darwin^2^. This phenomenon is similar to many recently scrutinized phenotypic traits such as feeding on poisonous milkweed for many insects, wing coloration patterns in butterflies and lateral plates in multiple sticklebacks^20^.

Our phylogenetic (whole genome and mitogenome) analyses also demonstrate that *L. yunnana*, is close to typical Luciolinae species (*A. terminalis* and *Aquatica lateralis*) with 100% support, and had diverged from Luciolinae about 56–103 Mya (Fig. 2a,b). This species has been placed originally in Lampyrinae because of its similarities in morphology and luminous behavior to typical Lampyrinae species (*Pyrocoelia pectoralis* and *P. pyralis*)^21^. We also compared 3125 single-copy orthologs among five fireflies, and the results show that *L. yunnana* presented a higher average amino acid (AA) identity to Luciolinae (*A. terminalis*: 77.44%; *A. lateralis*: 78.95%) than Lampyrinae (*P. pyralis*: 74.39%; *P. pectoralis*: 74.48%) (Fig. 2d), and that approximately its 65.96% genes are closer to those of *A. terminalis* and *A. lateralis* in sequence identity, while only 3.76% are closer to those of *P. pyralis* and *P. pectoralis* (Fig. 2e). The phylogenetic analysis of mitogenomic gene and rDNA genes^19,22^ and our comparison on morphology of *Lamprigera* with that of a recently described fossil species in Luciolinae^23^ support the close relationship between *Lamprigera* and Luciolinae. These combined data demonstrate that *L. yunnana* has a closer phylogenetic relationship to Luciolinae than Lampyrinae, and thus *L. yunnana* should be a member of Luciolinae rather than a species in Lampyrinae.

### Evolution of genes and gene families along Elateroidea

**Figure 3:**
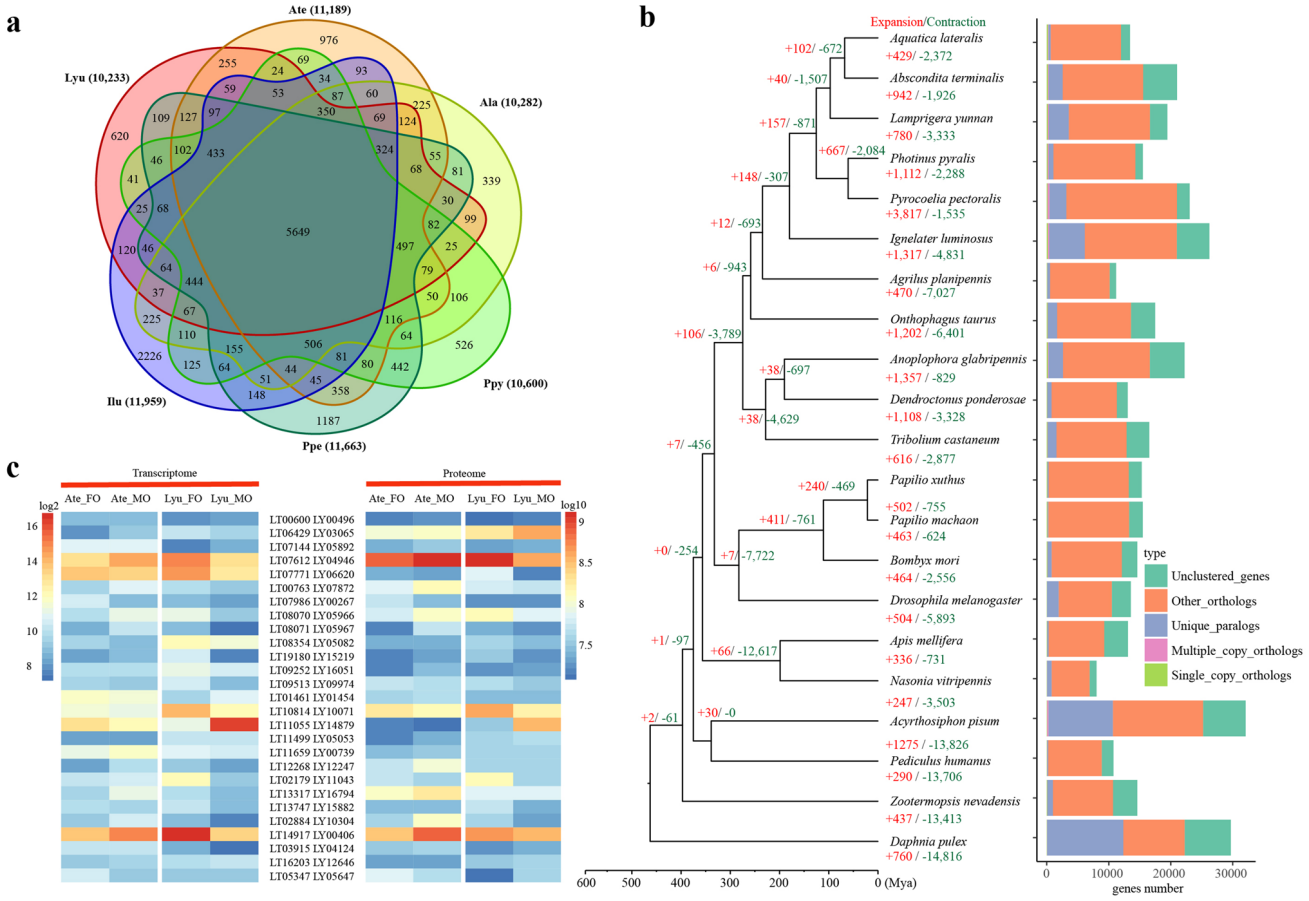
Evolution of gene families and highly expressed genes in luminous organs of two species. (**a**) Venn diagrams showing unique and overlapping protein families among six luminous insects including *Lamprigera yunnana* (Lyu), *Abscondita terminalis* (Ate), *Aquatica lateralis* (Ala), *Photinus pyralis* (Ppy), *Pyrocoelia pectoralis* (Ppe) and *Ignelater luminosus* (Ilu). The number in bracket shows the number of clustered gene families of each species. (**b**) The expanded and contracted gene families along the phylogenetic tree and ortholog assignment. Numbers on each terminus indicate the number of gene gains (+) or losses (−) for each taxon. Bars are subdivided to represent different types of ortholog clusters as indicated. “Single_copy_orthologs” indicates one to one gene among all genomes. “Multiple_copy_orthologs” indicates those orthologous genes present in multiple copies among all genomes. “Other_orthologs” indicates those genes present in at least two genomes, but not all genomes. “Unique_paralogs” indicates specific duplication in only one species. “Unclustered_genes” indicates no homologous relationship between one and any other taxa. (**c**) The expression of highly expressed orthologs in the luminous organs of *L. yunnanna* (Lyu) and *A. terminalis* (Ate) at both transcriptomic and proteomic levels. MO: luminous organ of male adult; FO: luminous organ of female adult.

To explore the genomic basis of the origin and evolution of bioluminescence in insects, we performed a comparative genomics analysis among 21 species (six luminous beetles in Elateroidea and five non-luminous beetles in other five superfamilies (Coleoptera), nine representative species from five insect orders (Diptera, Lepidoptera, Hymenoptera, Hemiptera, Phthiraptera, Isoptera), and one Crustacea species (Branchiopoda)) (Fig. 3a; Supplementary Notes 5–6, Tables S15–S46, Figs. S13–S29, Data S1–S10). Analyses of gene family expansion and contraction show that 148 families are expanded in the ancestor of Elateridae-Lampyridae beetles (Elateroidea: currently all luminous beetle species are in this superfamily), of which gene ontology terms are significantly related to bioluminescence, peroxisome and catalytic activity, and KEGG is significantly related to the pathway of membrane transport (ABC transporters) and signal transduction (cAMP signaling pathway) (hyper test, corrected *p* < 0.01) (Fig. 3b; Supplementary Note 5, Tables S15–S30, Data S1–S4). The evolutionary analyses on genes among Elateroidea (only luminous taxa) and non-Elateroidea (all non-luminous taxa) (Supplementary Tables S31–S32, Figs. S13–S17, Data S5–S10) show that 190 orthologs are positively selected genes (PSGs) in the ancestor of Lampyridae-Elateridae (Elateroidea), which are mainly related to catalytic activity and ATP binding (Supplementary Data S5–S7). Specifically, these genes in calcium signaling (e.g. sarcoplasmic/endoplasmic reticulum calcium-transporting ATPase (SERCA), calreticulin) and in ATP binding cassette (ABC) transporter (i.e. ABC-D) were included. A thorough analysis of transcriptomes and proteomes of the adult luminous organs of *L. yunnana* and *A. terminalis* (Fig. 3c; Supplementary Note 6, Tables S46, Figs. S18–S29, Data S11–S18) indicates that the highly expressed genes at both transcriptomic and proteomic levels in both species and sex are related to bioluminescence and ATP metabolic process. These results, combined with the dispersedly phylogenetic distribution of bioluminescence in Elateroidea and within Elateridae (Fig. 2b), suggest that convergent genetic evolution of these genes (families) in the luminous lineages of Elateroidea may contribute to the phenotypically convergent evolution of bioluminescence.

### Origin and evolution of luciferase genes

**Figure 4:**
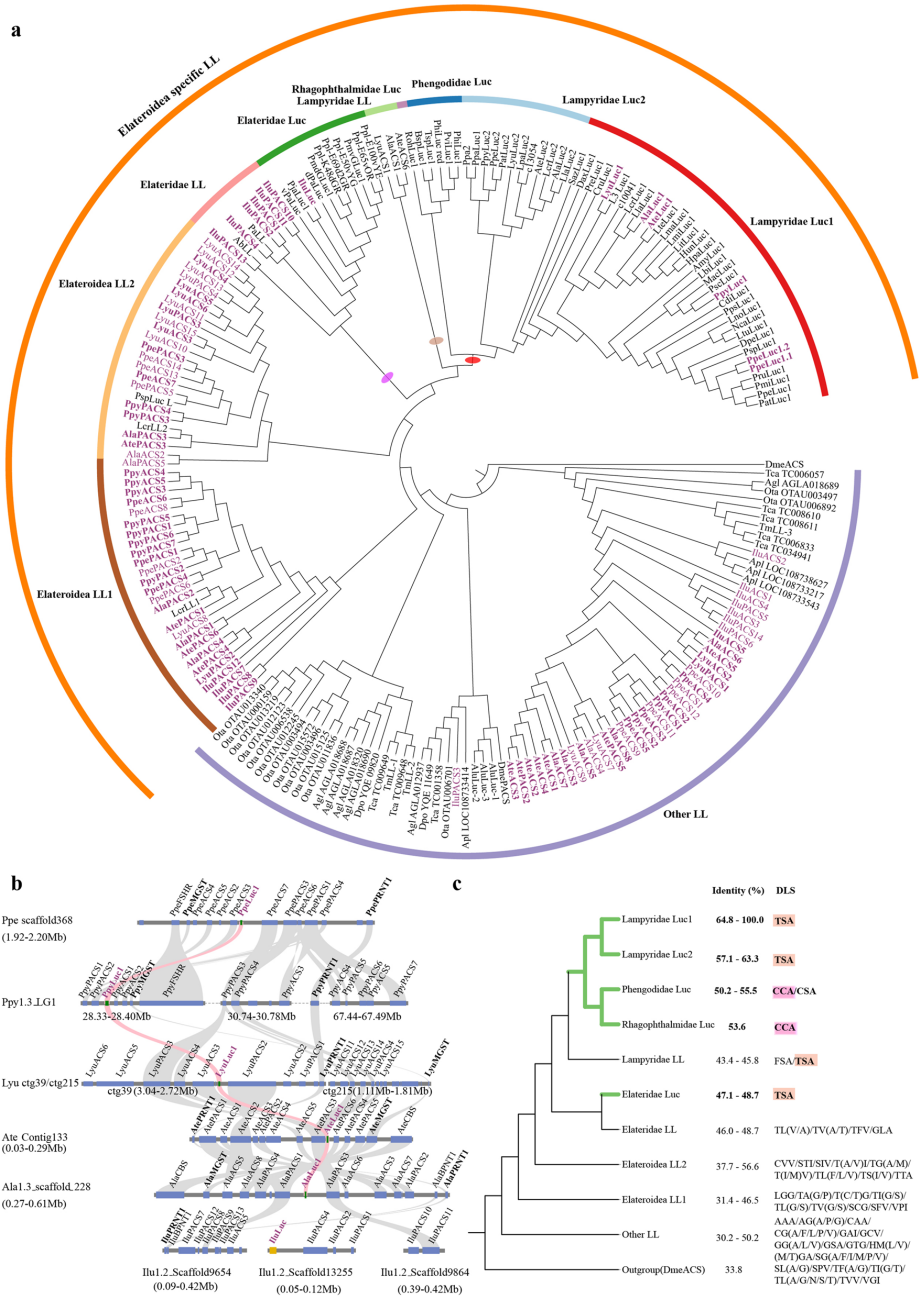
Origin and evolution of luciferase gene and its function. (**a**) Maximum likelihood tree of insect luciferase-like (LL) protein with DmeACS (*CG9009*) as an outgroup. The red oval represents the luciferase clade of Lampyridae, Rhagophthalmidae and Phengodidae. The brown oval represents the paralogous genes clade (Lampyridae LL) of luciferase. The purple oval represents the Elateridae-luciferase and Elateridae-luciferase-like clade. ACS denotes acyl-CoA synthetases (without peroxisomal targeting signal (PTS)) and PACS denotes peroxisomal ACS (with PTS). The following species with the luciferase-like genes identified in their reference genomes: Lyu: *Lamprigera yunnana*; Ate: *Abscondita terminalis*; Ala: *Aquatica lateralis*; Ppy: *Photinus pyralis*; Ppe: *Pyrocoelia pectoralis*; Ilu: *Ignelater luminosus*; Apl: *Agrilus planipennis*; Ota: *Onthophagus taurus*; Agl: *Anoplophora glabripennis*; Dpo: *Dendroctonus ponderosae*; Tca: *Tribolium castaneum*; Dme: *Drosophila melanogaster*. Pinked genes were identified in the genomes of luminous species. Highlighted in bold were shown those genes physically clustered with luciferase (Luc1) in (**b**). Other luciferase-like genes were the cloned sequences retrieved from GenBank. The abbreviations for cloned and luciferase-like genes identified see Supplementary Data 19 and Supplementary Data 21, respectively. (**b**) Microsynteny analysis of syntenic block surrounding luciferase (Luc1) across six luminous species. Syntenic Luc1 was highlighted in pink. Microsomal glutathione S-transferase: MGST; Polyribonucleotide nucleotidyltransferase 1: PRNT1. (**c**) All bioluminescent luciferases show a pattern of “TSA/CSA/CCA” in potential d-luciferin interacting sites (DLS) and a high amino acid identity to the luciferase of American firefly *Photinus pyralis* (Ppy-luc1) (> 47%). Luminous clades are highlighted in green.

To explore the origin of bioluminescence, we scrutinize the origin and evolution of luciferase among beetles (Supplementary Note 7, Figs. S30–S35, Data S19–S24) with more comprehensive methods than the previously reported^13^. Luciferase genes have been cloned from about 40 luminous beetles (Supplementary Data S19) and belong to acyl:CoA synthetase (ACS) superfamily^5^ with a close relationship to 4-coumarate:CoA ligase (4CL) family^24^. A thorough genome-wide investigation of ACS genes among beetles (six luminous beetles and five non-luminous beetles) (outgroup: *Arabidopsis thaliana* 4CL (Ath4CL1)) shows that a luciferase-like clade (including luciferase gene), together with its sister clade, 4-coumarate:CoA ligase, located at the terminus of the ACS gene tree and expanded greatly, almost occupying half of ACS genes in eight families (Supplementary Fig. S30). Further phylogenetic analysis on all luciferase-like and 4-coumarate:CoA ligase of beetles (Supplementary Fig. S31), together with those previously cloned luciferase homologs (Supplementary Data S19), demonstrated that except that of non-luminous *Zophobas morio* (ZopLL) belonging to 4-coumarate:CoA ligase, all other previously cloned luciferase-like homologs from non-luminous beetle *Tenebrio molitor* or from luminous beetles are luciferase-like genes. With our main aim to explore the origin of luciferases in luciferase-like family, we further focused our analysis on luciferase-like gene evolution (Fig. 4a). All above mentioned phylogenetic trees inferred from ACS, luciferase-like genes + 4-coumarate:CoA ligase or luciferase-like genes, show that an Elateroidea-specific luciferase-like clade evolved at the tree terminus, and within it, all luciferase genes in three phylogenetically related luminous taxa (i.e. Lampyridae, Rhagophthalmidae and Phengodidae) formed one terminal clade (marked by a red oval) with some of their paralogues at its base (marked by a brown oval) (Fig. 4a), which is sister to the Elateridae-luciferase + Elateridae-luciferase-like clade (marked by a purple oval) of Elateridae, a taxa including some luminous species. Our results are consistent with previously reported phylogeny of luciferases and their paralogues identified from the reference genomes of two fireflies and one luminous click beetle^13^ or non-luminous beetle genomes^25^. We estimate that the ancestor of the luciferase gene in Lampyridae (plus Rhagophthalmidae, Phenogodidae) may have diverged around 205 Mya (Supplementary Figs. S32–S33), long before the divergence of Lampyridae and Elateridae inferred from phylogenomic data (174-115 Mya). Elaterid luciferase gene, in contrast, evolved at a more recent time (~ 131 Mya) (Supplementary Fig. S32). Synteny analysis revealed the conserved syntenic blocks surrounding the luciferase locus across Lampyridae clades, which, however, is not syntenic to luciferase block in Elateridae (Fig. 4b). This suggests that luciferases in Lamyridae and Elateridae were evolved from different luciferase-like copies and different time. Amino acid sequence analysis indicates that all bioluminescent luciferases possess a pattern of “TSA/CSA/CCA” (Fig. 4c) in a loop region between beta-sheets of N-terminal domain^4,26^ possibly interacting with luciferin^27^ and an overall amino acid identity of more than 47% to that of *P. pyralis*, suggesting that this amino acid sequence pattern played a key role in the bioluminescent function of beetle luciferase. All these data (phylogeny, divergence time, syntenic analysis) support that the bioluminescent function of luciferase genes was independently evolved in Lampyridae (plus Rhagophthalmidae and Phengodidae) and Elateridae, as proposed in a previous study^13^.

### Luciferin biosynthesis revealed by multilevel data

We thoroughly investigated the genic basis of luciferin biosynthesis by integrating multilevel data including comparative genomics of luminous and non-luminous beetles, gene expression at both transcriptomic and proteomic levels in adult luminous organs of *L. yunnana* and *A. terminalis*, functional verification of genes in vitro experiments, and CRISPR/Cas9 gene editing (Supplementary Notes 6–8, Tables S33–S53, Figs. S36–S67, Data S11–S31). Our data provided several lines of evidence about luciferin biosynthesis (precursor origin, conformation change and storage, and biosynthetic place). Importantly, based on the following analysis on these data and previous investigation into luciferin metabolism^28^, we propose a complete pathway of luciferin biosynthesis in fireflies as shown in Fig. 5a.

**Figure 5:**
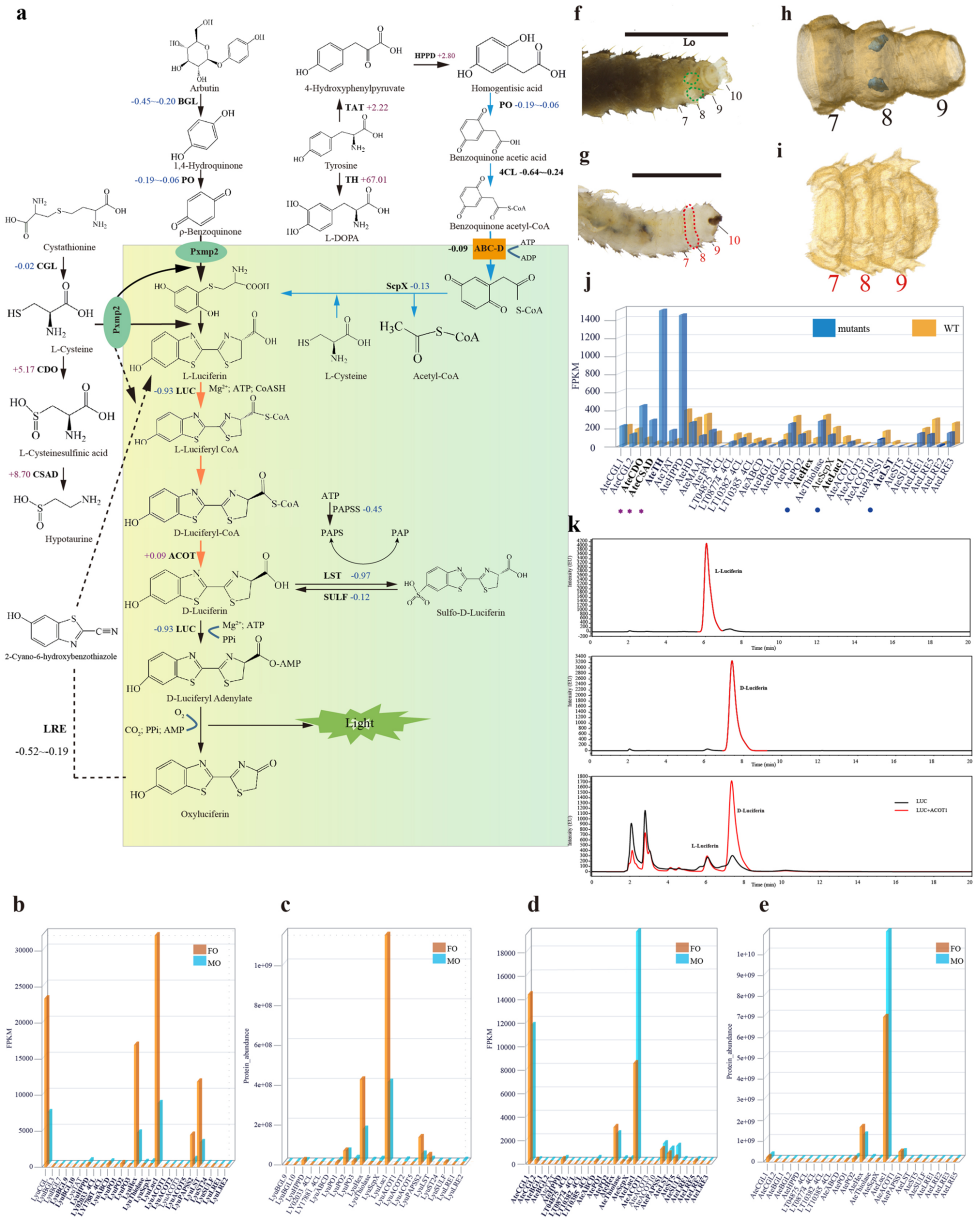
The pathway of luciferin biosynthesis proposed based on multilevel data. (**a**) The proposed pathway. The peroxisome is highlighted with yellow-green. Blue arrows denote our inference. The orange arrows denote these steps were experimentally verified in **k**. The changed folds of transcriptomic expression of candidate genes in mutants (**g**–**i**) of *Abscondita terminalis* compared with their wild types (WT) (**f**–**h**) were labeled (decrease (−): blue; increase (+): pink). Abbreviations: cystathionine gamma-lyase (CGL), cysteine dioxygenase (CDO), cysteinesulfinic acid (CSA) decarboxylase (CSAD), tyrosine aminotransferase (TAT), tyrosine hydroxylase (TH), 4-hydroxyphenylpyruvate dioxygenase (HPPD), homogentisate 1,2-dioxygenase (HD), maleylacetoacetate (MAA) isomerase (MAAI), fumarylacetoacetase (FAH), 4-coumarate-CoA ligase (4CL), ATP-binding cassette protein D subfamily (ABC-D), β-glucosidase (BGL), phenoloxidases (PO), luciferase (LUC), acyl-CoA thioesterases (ACOT), luciferin sulfotransferase (LST), sulfatase (SULF), 5′-phosphosulfate synthetase (PAPSS), luciferin-regenerating enzyme (LRE), peroxisomal membrane protein 2 (Pxmp2). (**b**–**e**) Transcriptomic (**b**, **d**) and proteomic (**c**, **e**) expression of candidate genes in luminous organs of *L. yunnana* (Lyu) and *A. terminalis* (Ate). Those genes with abundance at proteomic level were shown bold in (**b**, **d**). (**f**–**i**) The ventral view of CRISPR/Cas9-induced morphological mutants (**g**, **i**: no luminous organs and the regions correspondent to luminous organs of wild type highlighted in red in (**g**)) of *Abdominal-B* in *A. terminalis* and their WT (**f, h:** having luminous organs labelled in green circled area in (**f**) and shown blue spots in (**h**)). The numbers show the abdominal 7 segments (A7)-A10 and abnormal segments were shown in red number; **f**–**g:** observed using the microscope SMZ1000 (Nikon, Japan) and photos were taken using Canon 700D; **h**–**i:** three-dimension reconstruction of A7-A10. (**j**) Expression of candidate genes between mutants and wide types. The pink stars and blue circles show the significant (|log2(FC)|> 2, *P* < 0.01) up-regulated genes and down-regulated genes in *A. terminalis* mutants, respectively. (**k**) The stereoisomeric conversion of l-luciferin to d-luciferin in vitro by luciferase and acyl-CoA thioesterase (ACOT). The three HPLC (high-performance liquid chromatography) chromatograms represent standard l-luciferin (up), standard d-luciferin (middle) and stereoisomeric inversion of l-luciferin to d-luciferin in vitro by luciferase and acyl-CoA thioesterase (ACOT) (down).

Our gene expression analysis at both the transcriptomic and proteomic levels in the luminous organs of both species and sex showed that all enzymes (especially cystathionine gamma-lyase to catalyze production of l -cysteine from cystathionine) in cysteine anabolism (from methionine to l-cysteine) presented a high expression levels, while cysteine dioxygenase and l-cysteinesulfinic acid decarboxylase in cysteine catabolism (from l-cysteine to taurine) were low/no expression (Fig. 5b–e; Supplementary Note 7, Figs. S36–S37, Data S25), suggesting that l-cysteine, one precursor of luciferin biosynthesis^29, 30^, origins from cysteine anabolism. Up-regulation of cysteine dioxygenase and cysteinesulfinic acid decarboxylase in *A. terminalis* larval mutants with the loss of luminous organs induced by *Abdominal B* (*Abd-B*) knock-out (Fig. 5f–j; Supplementary Note 8, Tables S52–S53, Figs. S62, S65–S67, Data S30–31) further consolidates the source of cysteine used for luciferin biosynthesis in luminous organs.

A new possible precursor, homogentisic acid/benzoquinone acetic acid, is proposed in this study. The photogenic layer of firefly lantern is rich in tyrosine^31^. Homogentisic acid/benzoquinone acetic acid, intermediates from tyrosine degradation (Fig. 5a; Supplementary Note 7, Data S25) has similar structures with 1,4-hydroquinone/*p*-benzoquinone (another precursor of luciferin biosynthesis proposed previously^29,30,32^). Homogentisic acid (produced from 4-hydroxyphenylpyruvate (HPP) catalyzed by 4-HPP dioxygenase) can be oxidized by polyphenol oxidase into benzoquinone acetic acid^33–35^. The latter, after being activated into benozoquinone acetyl-CoA possibly by one of 4-coumarate: CoA ligase with high expression in luminous organs (Fig. 5a; Supplementary Fig. S35), may be catalyzed by thiolase activity of sterol carrier protein-X (ScpX) into backbone *p*-benzoquinone via thiolase reaction mechanism of β-oxidation in peroxisomes (removing an acetyl group)^36,37^. After removing an acetyl group, the group-sulfhydryl (SH) of a cysteine, instead of SH of acetyl-CoA in normal beta-oxidation^36^, may again react with terminal carbon of the backbone *p*-benzoquinone to form 2-S-cysteinylhydroquinone, which is an intermediate for the firefly luciferin biosynthesis and can be further changed into l-luciferin in case of adding another l-cysteine^38^. Our expression analysis showed a high expression (at both transcriptomic and proteomic levels) of some enzymes in tyrosine degradation (e.g. 4-hydroxyphenylpyruvate dioxygenase), hemocyanin (including polyphenol oxidase, hexamerin), 4-coumarate: CoA ligase and thiolase (ScpX, thiolase) in the luminous organs of both species and sexes (Fig. 5b–e; Supplementary Figs. S39, S52–S54, Data S25–S26). In mutants of *A. terminalis* generated by our gene-editing (Fig. 5f–i; Supplementary Note 8, Tables S52–S53, Figs. S60–S64, Data S30–S31), hexamerin was down-regulated while tyrosine hydroxylase (converting tyrosine to l-DOPA) was significantly up-regulated (Fig. 5j; Supplementary Data S30), suggesting an alternative metabolic direction of tyrosine in the case of luciferin biosynthesis blockage. It is noted that 1-4-hydroquinone, previously proposed to be the precursor of l-luciferin biosynthesis, was thought to be stored as arbutin^32^ and could be produced by glucosidases hydrolysis^28^. We identified the expression (transcriptomic and proteomic) of glucosidases in the luminous organs of both species and sexes (Supplementary Figs. S52–S54). Thus, we retain the branch pathway of 1-4-hydroquinone (stored as arbutin) as the precursor of l-luciferin biosynthesis^32^ here.

**Figure 6:**
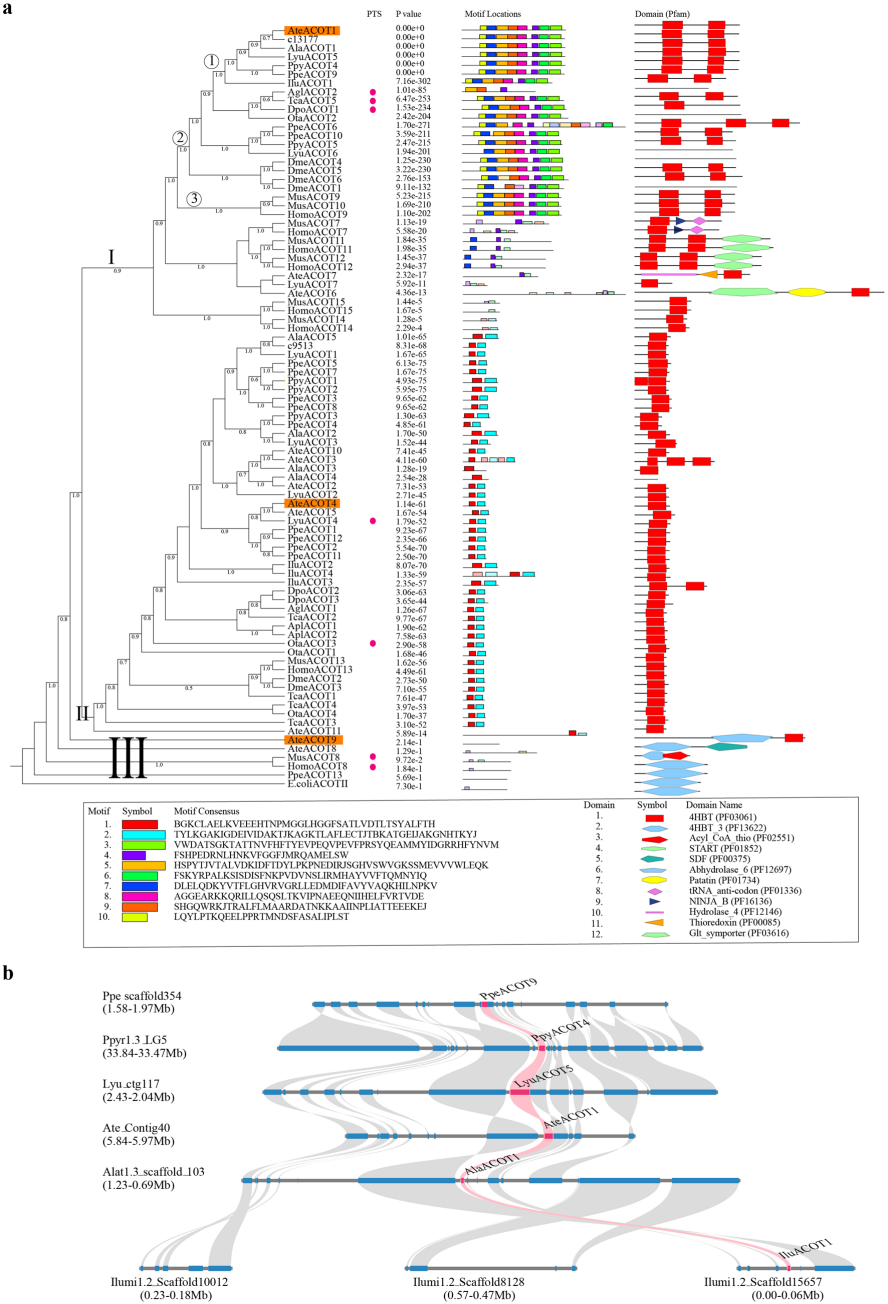
The evolution of acyl-CoA thioesterases (ACOTs) in beetles and other organisms. (**a**) The phylogenetic tree, motifs and domains. The tree was divided into three clusters, I, II and III. In cluster I, ① shows the clade of single copy syntenic orthologs (see **b**) (also as candidates in luciferin biosynthesis) in luminous beetles, ② shows the specific clade in insect ACOTs and ③ shows the clade of mammals ACOTs locating in mitochondria. The highlighted three ACOTs (AteACOT1, AteACOT4 and AteACOT9) were selected as candidates to verify their function in converting l-luciferin to d-luciferin in vitro experiments. The red circles show those genes with the C-terminal peroxisomal targeting signal 1 (PTS1). The probability of motifs was showed using P value. The domains were identified using Pfam database. 4HBT and 4HBT_3 domains belong to CL0050 Hotdog superfamily. E.coliACOTII shows fatty acyl-CoA thioesterase (ACOT) II (TESB) from *Escherichia coli*. b shows the syntenic relationships of ACOT gene highlighted in pink and its flanking genes surrounding up-200 kb and down-200 kb genomic regions among six luminous beetles. Lyu: *Lamprigera yunnana*; Ate: *Abscondita terminalis*; Ala: *Aquatica lateralis*; Ppy: *Photinus pyralis*; Ppe: *Pyrocoelia pectoralis*; Ilu: *Ignelater luminosus*; Apl: *Agrilus planipennis*; Ota: *Onthophagus taurus*; Agl: *Anoplophora glabripennis*; Dpo: *Dendroctonus ponderosae*; Tca: *Tribolium castaneum*; Dme: *Drosophila melanogaster*; Mus: *Mus musculus*; Homo: *Homo sapiens*.

D-luciferin as the substrate of luciferase in firefly bioluminescence, is generated from the chirality transition of luciferin^39^. The enzymes participating in conversion of l-luciferin to d-luciferin, including luciferase (LUC) for l-enantioselective thioesterification of l-luciferin and acyl-CoA thioesterase (ACOT) for hydrolysis, have been proposed^28,39,40^. Moreover, a possible luciferin storage mechanism was proposed in fireflies that luciferin sulfotransferase catalyzes the production of sulfoluciferin (a luciferin storage molecule, inactive for luciferase) from firefly luciferin and sulfo-donor 3′-phosphoadenosine 5′-phosphosulfate (PAPS) produced from ATP and inorganic sulfate under the catalysis of PAPS synthase (PAPSS)^41^. Our expression analysis shows that above mentioned enzymes involved with biosynthesis of d-luciferin and storage present a high expression at both transcriptomic and proteomic levels in the luminous organs of both species and sexes (Fig. 5b–e; Supplementary Figs. S52–S54, Data S27). In the *A. terminalis* mutants (Fig. 5f–j; Supplementary Note 8, Tables S52–53, Fig. S65, Data S30), luciferase and luciferin sulfotransferase were significantly down-regulated. The most noteworthy point is the role of the acyl-CoA thioesterases. Although a deracemizative luminescent system containing luciferase from firefly *Luciola cruciata* and fatty acyl-CoA thioesterase II (TESB) from *Escherichia coli* confirmed the possible role of luciferase and acyl-CoA thioesterase in converting l-luciferin to d-luciferin^40^, neither comprehensive genomic identification nor functional study on any insect acyl-CoA thioesterases was reported. Our phylogenomic investigation indicates that regardless of a great copy number variation (Supplementary Data S26), the acyl-CoA thioesterases of all investigated insects are belong to type-II ACOTs, and together mammals’ (human and mouse) type-II ACOTs^42,43^, they mainly cluster into two groups (Fig. 6a). One group (cluster-I) includes most mammalian type-II ACOTs (7, 9–12) at its base and some insect ACOTs at its terminus; and most of these insect ACOTs, like their closest sisters (i.e., mammalian mitochondrial acyl-CoA thioesterases (HomoACOT9, MusACOT9-10)), exhibit eight similar gene sequence motifs and contain two 4HBT (4-hydroxybenzoyl-CoA thioesterase) domains (Fig. 6a). Interestingly, the luminous beetle-specific and single-copy syntenic orthologs (Fig. 6a) of those insect ACOTs show high expression in luminous organs at transcriptomic or/and proteomic levels (Supplementary Figs. S52–S54, Data S27). Another group (cluster-II), including mammalian ACOT13^43,44^ and multiple insect ACOT paralogs, show only two similar gene sequence motifs and one 4HBT domain, and only some of these luminous beetle ACOTS show expression in luminous organs at transcriptomic or/and proteomic levels (Fig. 6a; Supplementary Figs. S52–S54, Data S27). Additionally, some ACOTs from the two fireflies, together with some mammalian peroxisomal ACOTs (HomoACOT8 and MusACOT8), locate at the base (cluster-III) of all other ACOTs of insects and mammals, and show similar gene sequence motifs or protein domains to that of the fatty acyl-CoA thioesterase II (TESB, ACOTII) of *E. coli* in spite of no expression in luminous organs (Fig. 6a; Supplementary Fig. S53). Based on phylogenetic, gene sequence motifs and domain features, we selected three representative acyl-CoA thioesterases of *A. terminalis* from above mentioned three groups (AteACOT1: cluster-I, high expression at both transcriptomic and proteomic levels; AteACOT4: cluster-II, high expression at transcriptomic level; AteACOT9: cluster-III, similar protein domain to that of *E. coli* ACOTII) to verify their role in luciferin deracemization in vitro experiment (Supplementary Note 7). Our results demonstrated that only the highest expressed acyl-CoA thioesterase (AteACOT1) (Fig. 5d,e; Supplementary Fig. S54) can efficiently convert l-luciferin to d-luciferin (Fig. 5k; Supplementary Fig. S47), which is the first verified the function of acyl-CoA thioesterase in insects.

Bioluminescent reaction occurs in the peroxisome in insects. However, the location of luciferin synthesis is still mysterious. A thorough whole-genome identification on peroxisome targeting signal (PTS) (incl. PTS1 or PTS2) and peroxisomal membrane proteins (two peroxin (Pex) genes, *Pex5* and *Pex14*)^45^ were performed. Combined with the expression in luminous organs (Supplementary Note 7, Fig. S57, Data S28), our results suggest that peroxisomes are the function place of sterol carrier protein-X and luciferase. The positive selection on one member of the D subfamily of ATP-binding cassette gene family (ABC-D) in the ancestor of luminous beetles (Supplementary Note 7, Fig. S58, Data S6) and the high expression of its orthologs in *L. yunnana* (*LY01293*) and *A. terminalis* (*LT01539*) (Fig. 5b,c; Supplementary Fig. S59) suggest that, like human ABC-D genes (i.e. the import of long and branched chain acyl-CoA molecules into the peroxisome^46^), this selected gene may promote import of benozoquinone acetyl-CoA (i.e. branched chain acyl-CoA) into peroxisome in luminous beetles. The high expression (Supplementary Note 7, Fig. S57, Data S27) of membrane channel Pxmp2 protein (PMP22) in all luminous beetles, which can transfer metabolite of < 300 Da across the peroxisomal membrane^47^, may contribute to the diffusion of cysteine (121 Da), 1,4-hydroquinone (108.09 Da) and 1,4-benzoquinone (108.09 Da) into peroxisome. These results provide the evidence that luciferin is biosynthesized in peroxisomes (Supplementary Note 7, Table S48, Figs. S55–S59). However, we noticed that no peroxisomal targeting signal can be identified in those luminous beetle-specific lineage of acyl-CoA thioesterases including AteACOT1 which is here verified to function in luciferin deracemization (Fig. 6a). How acyl-CoA thioesterases are transferred into peroxisome in insects is still an open question because no peroxisomal targeting signals are identified in those of fruit fly and other insects^48^.

To further explore the genetic causes of phenotypically convergent bioluminescence between Lampyridae and Elateridae, we assessed gene location (microsynteny) to confirm the orthology of major candidate genes (polyphenol oxidase, hexamerin, sterol carrier protein-X, luciferase, acyl-CoA thioesterase, luciferin sulfotransferase, sulfatase and 3′-phosphoadenosine 5′-phosphosulfate synthase) in the proposed luciferin biosynthetic pathway among six luminous species (Lampyridae: 5; Elateridae: 1) (Fig. 6b; Supplementary Figs. S41–S43, S48–S50). Our data showed that except luciferase gene and luciferin sulfotransferase (LST, not exist in *Ignelater luminosus* (Ilu))^13^, all other genes have good syntenic relationships between Lampyridae and Elateridae, suggesting their same copies were recruited in luciferin biosynthesis. Although there is no LST gene loci present in *I. luminosus*, we found that the three sulfotransferases (ST) (IluST8, IluST10, IluST13) had high homology with LST (identity of amino acid sequence > 50%) (Supplementary Fig. S48), suggesting that sulfotransferase in *I. luminosus* could have a capability similar to LST. For luciferase, as discussed in the preceding section (Fig. 4; Supplementary Figs. S30–S35), they were independent evolution between Lamyridae and Elateridae, and all bioluminescent luciferases possess a special pattern in region possibly interacting with luciferin. Combining these results, we conclude that luciferase, functioning not only in light production but also in luciferin biosynthesis, plays a leading role in the origin of luciferin and thus bioluminescence. Meanwhile, our results display convergent molecular function in the pathway of luciferin synthesis, uncovering the genetic causes of convergent bioluminescence between Lampyridae and Elateridae.

### Genetic basis of light on/off and its pattern

**Figure 7:**
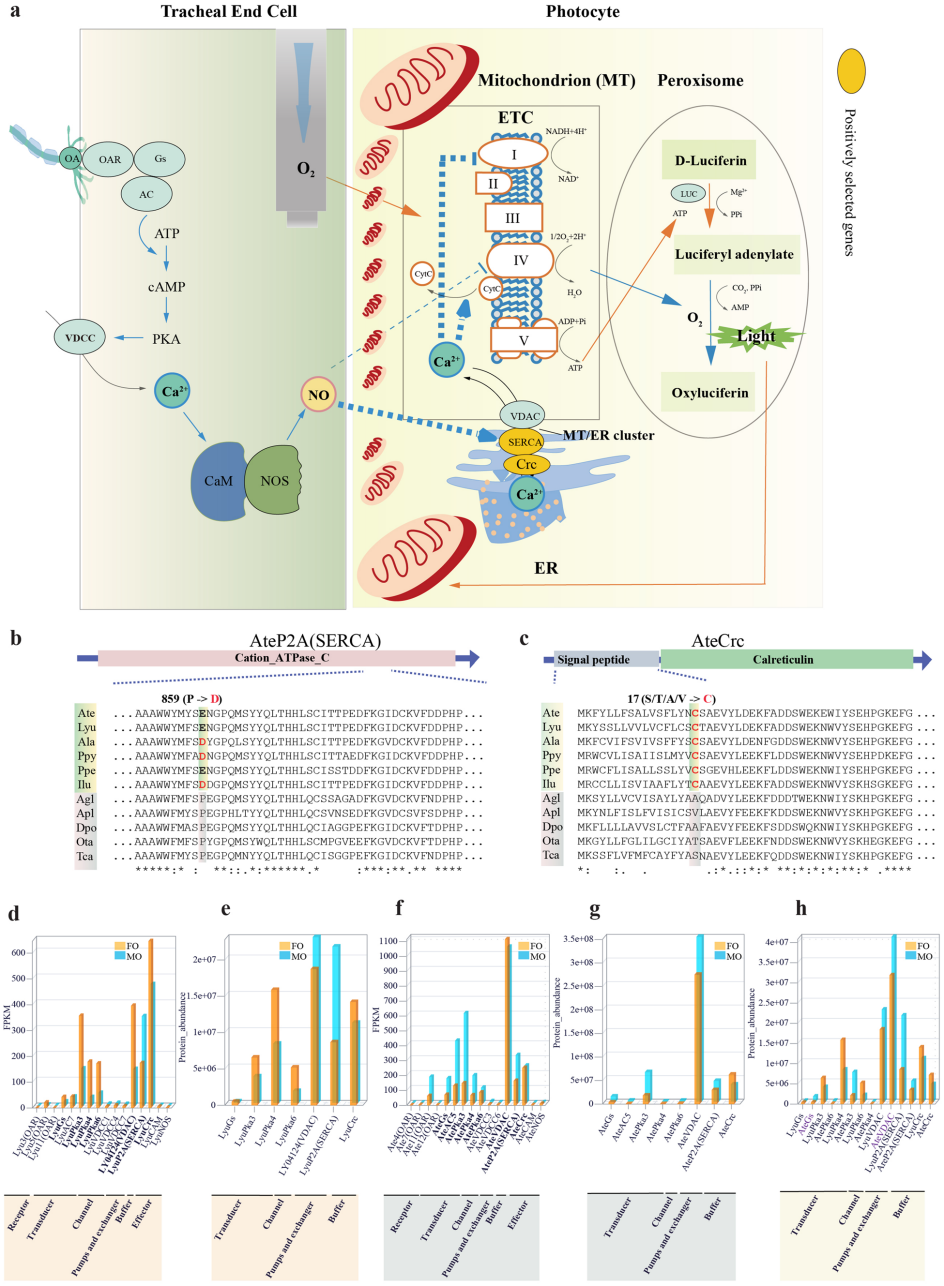
Genetic basis of light on/off and its pattern (glow and flash). (**a**) The proposed model of light on/off control. Orange lines, active during light “off”; blue lines, active during light “on”; thin lines, previously reported^9,10^; thick dashed lines, currently proposed; lines with arrows, promotion; lines with vertical bars, prohibition. The octopamine (OA), released from the nerve synapses, arrives at the tracheolar end cell and binds the octopamine receptors (OAR), which activated a cyclic adenosine monophosphate/cAMP-dependent protein kinase (cAMP/PKA) pathway. The activated PKA can phosphorylate voltage dependent calcium channels (VDCC) to enhance the ability of Ca^2+^ to enter the cytoplasm. The Ca^2+^, by binding to calmodulin (CaM), activated nitric oxide synthetase (NOS) to release nitric oxide (NO). NO can readily diffuse across cell membranes into photocytes and inhibit the electron transport chain (ETC) by the following two ways. Firstly, NO directly inhibits cytochrome c oxidase. Secondly, NO stimulates the sarco/endoplasmic reticulum (SR/ER) Ca^2+^-ATPase (SERCA) to release Ca^2+^ into mitochondria from ER via the mitochondria/ER clusters; Ca^2+^ inhibits respiration at complex I and also dissociated cytochrome c (CytC) from the inner membrane. Both of these ways prevent ETC binding and use of oxygen, and thus oxygen is then free to diffuse beyond the mitochondria to the peroxisomes, where the luciferin can be oxidized by luciferase (LUC) to emit light. As time goes by, NO is degraded by light and terminate the action of the NO. The Ca^2+^ signal is inactivated and its concentration in mitochondria reduced. The respiration once again increases and the adenosine triphosphate (ATP) continuous accumulated for next bioluminescent reaction. Abbreviations: α subunits of guanine nucleotide-binding (G) proteins (Gs), adenylate cyclase (AC), voltage-dependent anion channel (VDAC), calreticulin (Crc). (**b**, **c**) Positive selection sites (highlighted) of SERCA (**b**) and calreticulin (**c**) in ancestor of luminous beetles (shaded in light green). (**d**–**g**) Transcriptomic (**d**, **f**) and proteomic (**e**, **g**) expression of candidate genes in luminous organs of adult females and males of *L. yunnana* (Lyu) (**d**, **e**) and *A. terminalis* (Ate) (**f**, **g**). (**h**) The comparison of proteomic expression for orthologs of *L. yunnana* and *A. terminalis*.

To explore genetic basis underlying light on/off and its pattern (i.e. glow, flash), we combined comparative genomics with transcriptomic and proteomic data of luminous organs of glow (*L. yunnana*) and flash (*A. terminalis*) taxa to investigate the gene families (Fig. 7a; Supplementary Note 9, Tables S54–S58, Figs. S68–S89, Data S32) related to the previously reported flash control model^9,10^ and cell calcium signaling pathway because calcium ions were involved in the intense, long lasting scintillation in *Photuris* fireflies^49^. Our results indicate that two positively selected genes (sarcoplasmic/endoplasmic reticulum calcium-transporting ATPase and calreticulin) of the calcium signaling pathway in the ancestor of luminous beetles (Fig. 7b,c) and voltage-dependent anion channel (VDAC) have a strongly expression at transcriptomic and proteomic levels (Fig. 7d–g), especially for VDAC that showing a higher expression in flash firefly *A. terminalis* than in glow firefly *L. yunnana* (Fig. 7h). Sarcoplasmic/endoplasmic reticulum calcium-transporting ATPase, calreticulin and voltage-dependent anion channel play an important role in calcium signal between mitochondria and reticulum^50–52^. Additionally, our transcriptomic data also show that most of other genes in the previously reported flash model^9,10^ (i.e. octopamine receptors, one of α subunit genes of Guanine nucleotide-binding (G) proteins (Gs), adenylyl cyclases, cAMP-dependent protein kinase) generally have a high expression in flash firefly *A. terminalis* and glow firefly *L. yunnana* (Fig. 7d–g), especially for Gs that shows a higher expression in *A. terminalis* than in *L. yunnana* at proteomic expression (Fig. 7h). All these results suggest that calcium may play an important role in light display control by its communication between mitochondria and reticulum of photocytes (Fig. 7a; Supplementary Note 9). Nevertheless, other three genes (voltage-dependent calcium channel, calmodulin and nitric oxide synthase) in the previously proposed pathway^9,10^ show a very low gene expression in both species, and together with octopamine receptors, cannot be identified at the proteomic level (Supplementary Figs. S87–S89). Low gene expression of nitric oxide synthase was also reported in other fireflies^53^. These are unexpected results, especially for nitric oxide synthase, which was proposed to play an important role^11^. On the other hand, comparison of nitric oxide synthase among luminous beetles and other non-luminous insects shows that a specific amino acid site (Q) exists in the oxygenase domain of nitric oxide synthase in flash fireflies, while M/L/V/R/A is in the same position of glow beetles and non-luminous taxa (Supplementary Fig. S86, Data S33). Thus, it raised doubt on whether nitric oxide synthase plays a key role as reported^11^, on which further investigation is needed. In addition, our data indicate that the anatomic structures of luminous organs exhibit great differences with glow firefly having simple luminous organs and flash firefly having complex luminous organs (Fig. 1c–t; Supplementary Note 1, Figs. S3–S6), which may contribute to the evolution of light pattern, as proposed by Buck^3^. Taken together, our results suggest that the genes in the calcium signaling pathway and their expression difference play an important role in the evolution of light pattern among taxa (Fig. 1c–t, Fig. 7a). Further studies on cellular anatomy of luminous organs and the physiological role of the calcium signaling pathway in light reaction will promote the clarification of light pattern difference and evolution.

## Discussion

Our comprehensive investigation by integrating multilevel data provides multiple insights into the origin of luciferin and bioluminescence as well as firefly phylogeny. Our results clarify that the origin and evolution of luciferase genes play a leading role in the origin of luciferin and thus bioluminescence. Our experimental results demonstrate that one of acyl-CoA thioesterases can efficiently convert l-luciferin to d-luciferin (the substrate for bioluminescence reaction). Our phylogenomic analyses reveal a closer phylogenetic position of *Lamprigera* to Luciolinae, and thus *L. yunnana* should be a member of Luciolinae rather than a species in Lampyrinae. However, due to no available reference genomes of representatives from other families of Elateroidea (e.g., Rhagophthalmidae, Phengodidae, Cantharidae, Lycidae) and from non-luminous species in Elateridae now, a more expanded comparative genomics to include these related taxa will still be needed to clarify more details on the evolution of luciferase and other genes in luciferin biosynthesis and their contribution to bioluminescence origin. In addition, due to such natures of fireflies as long life circle, difficulty to rear in large scale in the laboratory etc., we were only able to get mutants of *Abd-B* knock-out by gene editing, while all other functional verification efforts on more genes, especially related to luciferin biosynthesis were proved to be tremendously difficult. More exploration on lab-rearing and gene editing of fireflies are needed to collect accurate data to testify the biosynthesis pathway of luciferin which we propose here in the future. This study has laid a pivotal foundation for future studies on all luminescent insect taxa together with efficient functional assays to completely reveal all mysteries underlying the fascinating phenomenon of firefly and all luminescent insects’ bioluminescence ever since Aristotle and Darwin.

## Methods

### Firefly collection, breeding and sample treatment

Adults and larvae of *Lamprigera yunnana* were collected in Kunming City, Yunnan, China from 2014 to 2016. Adults of *Abscondita terminalis* were collected in Menglun, Xishuanbana, Yunnan, China from 2015 to 2018. Live fireflies were brought in plastic containers back to the lab for observing their biological and morphological traits, breeding and storing samples frozen in liquid nitrogen until used. The last instar larva of *L. yunnana* at lower instars were got by feeding lower instar larva collected from wild with snails from the same habitat in covered plastic boxes (16 × 10 × 5 cm). Female and male pair of *A. terminalis* were transferred into covered plastic boxes (16 × 10 × 5 cm) padded with a wet paper napkin in an incubator at 25–27 °C and 70% relative humidity. The newly laid eggs were collected for continuous breeding at the same condition or for gene editing experiment. Hatched larvae were fed with chopped *Tenebrio molitor* larva to get different instar larvae and pupae. Adults collected in the wild were carefully dissected to get whole body but excluding wings (in case of pterygote stages), or only separated luminous organs, and frozen at − 80 °C until used.

The whole bodies of single *L. yunnana* female adult and single *A. terminalis* female were used for genome survey using Illumina sequencing technology. The whole bodies of another single *L. yunnana* female adult and another 15 *A. terminalis* female adults were used for de novo sequencing using single-molecular real-time (SMRT) technology in the PacBio platform. The whole body of single individual of both species at different developmental stages (*L. yunnana*: larvae, male adult and female adult; *A. terminalis*: larvae, male pupae, female pupae, male adult, and female adult), and luminous organs of both sex for both species (*L. yunnana*: mixed 3 individuals; *A. terminalis*: mixed 10 individuals) were used for transcriptomic sequencing. The luminous organs of both sex for both species (*L. yunnana*: mixed 6 individuals; *A. terminalis*: mixed 70 female individuals, mixed 40 male individuals) were used for proteomic sequencing.

### Genome sequencing and assembly

Genomic DNA for genome survey (Illumina sequencing) was extracted from the whole body of single female adult for both *L. yunnana* and *A. terminalis* using a Gentra Puregene Blood Kit (Qiagen, Germany) following manual instructions. The libraries of the 350 bp short insert were sequenced on the Illumina HiSeq4000 to obtain pair-end reads, which was used to estimate genomic characteristics based on *k*-mer frequency distribution (Supplementary Note 2) using a similar method as described previously^54^, and also used to polish assembled genomes based on only PacBio reads. For de novo sequencing (PacBio Sequencing), high-molecular-weight genomic DNA was extracted from the whole body of single *L. yunnana* female adult and 15 *A. terminalis* female adults with Sodium Dodecyl Sulfonate method, and the 20 kb libraries (four for *L. yunnana* and one for *A. terminalis*) were constructed and sequenced with a PacBio RS II platform (Pacific Biosciences, USA) using the P6 polymerase/C4 chemistry combination.

A long noisy reads assembler, wtdbg1^55^ (the source code is available on GitHub: https://github.com/ruanjue/wtdbg) was selected to assemble the genomes of two species as the followings (Supplementary Note 2). Firstly, using wtdbg, we performed the primary genome assembly of both species, followed by the first round of polishing using the wtdbg-cns program to produce the polished contigs and then the second round of polishing by combining minimap with wtdbg-cns to obtain the preliminary contigs. Secondly, the Quiver^56^ within SMRT Analysis v2.3.0 was used to polish base calling of preliminary contigs to improve the site-specific consensus accuracy of the assembly. Finally, we applied for the program Pilon^57^ with the “fix-all” mode to implement two consecutive rounds of polishing using Illumina short reads (Supplementary Table S1) to achieve the final assembly. The Illumina short reads, assembled transcripts and Benchmarking Universal Single-Copy Orthologs (BUSCO) were used to evaluate the completeness of assemblies (Supplementary Note 2).

### Genome annotation

Repetitive elements (transposable elements (TEs) and tandem repeats) were annotated using a combined strategy of de novo-based prediction, homology-based approach and Tandem Repeat Finder (TRF) in *L. yunnana* and *A. terminalis* (Supplementary Note 3). Gene model identification was conducted by a combination of de novo prediction, homology-based prediction and transcriptome-based prediction methods, and gene functional annotations were performed using BLASTP (E-value < 1e−5) against SwissProt^58^, TrEMBL^59^ and NCBI non-redundant protein Database (NR) (Supplementary Note 3). Genes were extracted based on the best BLAST hit along with their protein functional annotation. Structural protein domains and motifs were searched against SMART, ProDom, Pfam, PRINTS, PROSITE and PANTHER databases using InterProScan v5.25^60^. The Gene Ontology (GO) terms for genes were obtained from the corresponding InterPro entry. The metabolic pathways in which the genes might be involved were assigned by BLAST against the KEGG protein database^61^ with an E-value cut-off of 1e−5.

### Phylogenetic analysis and genome evolution

Gene families and single-copy orthologs were constructed using the OrthoMCL^62^ based on all-to-all BLASTP (E-value ≤ 1e−5) alignments. The phylogenetic tree of single-copy genes was constructed using RAxML v8.0^63^ under the GTR + gamma model, and their divergence times were estimated using the PAML v4.8^64^ mcmctree program. 15 beetles (6 luminous taxa in Elateroidea (5 fireflies, 1 luminous click beetles) and other 9 non-luminous beetles outside Elateroidea) with genomes available were included in the phylogenetic analysis with *Drosophila melanogaster* (Dme) as an outgroup (Supplementary Note 4). Besides, the mitogenomic phylogenetic tree of 39 Elateroidea species including five of above mentioned was also inferred with *T. castaneum* as an outgroup (Supplementary Note 4). To perform extensively comparison of genomes, 20 insect species (11 beetle species after removing 4 non-Elateroidea taxa of above mentioned 15 species because of their poor assembly and annotation; Lepidoptera: 3; Diptera: 1; Hymenoptera: 2; Hemiptera: 1; Phthiraptera: 1; Isoptera: 1) plus one outgroup (Crustacean, Cladocera: *Daphnia pulex*) were included in gene family clustering. CAFÉ (Computational Analysis of gene Family Evolution)^65^ was applied to infer gene family expansion and contraction by estimating the universal gene birth and death rate under a random birth and death model using the maximum likelihood method (Supplementary Note 5). 11 beetle taxa with their genomes of better assembly and annotation were further used to perform analysis of rapid evolving genes (REGs) and positively selected genes (PSGs) based on their 1,359 single-copy orthologous sets identified by SonicParanoid^66^. *Ka*, *Ks* and ω (*Ka*/*Ks*) were calculated using the Codeml program of PAML^64^ with the free ratio model for each branch based on 10,000 concatenated alignments constructed from all single-copy orthologs. The branch model in the Codeml program of PAML^64^ was used to identify REGs with the null model assuming that all branches have been evolving at the same rate and the alternative model allowing foreground branch to evolve under a different rate. To detect positive selection on a few codons along specific lineages, we used the optimized branch-site model following the author’s recommendation^67^.

### Transcriptome sequencing, proteome sequencing and analysis

Total RNA was extracted using the guanidinium thiocyanate-phenol–chloroform extraction method (Trizol, Invitrogen) according to the manufacturer’s protocol. RNA sequencing libraries (350 bp insert size) were generated using Illumina mRNA-Seq Prep Kit and sequenced using Illumina HiSeq4000 sequencer with read length of PE150. Two methods, i.e., de novo assembly of clean reads using Bridger^68^ with the default setting (*k*-mer size of 25) and mapping them back to the assembled genomes using Tophat^69^, were carried out for transcriptome assembly. The correlation of global expression (reads count) among samples was analyzed using cor function with spearman method from R program. The fragments per kilobase of exon per million fragments mapped (FPKM) values were calculated using Cufflinks^70^ software package and used to measure gene expression. The genes were remained with the total FPKM > 0 from all samples as expressed genes (EGs). The high expression genes (HEGs) were determined by choosing the first 5% EGs ranked from high to low based on the expression in luminous organs. The differentially expressed genes (DEGs) between luminous organs were analyzed using the EdgeR^71^ program. The genes with the absolute value of logFC ≥ 4 and false discovery rate (FDR) ≤ 0.01 were identified as DEGs of interspecific luminous organs due to longer species divergence time.

Total protein from each luminous organ sample was prepared, and 100 μg of proteins from each sample were used for tryptic digestion. The peptide samples were labelled using iTRAQ kits (Applied Biosystems, Foster City, CA) and analyzed using TripleTOF 5600+ mass spectrometer coupled with the Eksigent nanoLC System (SCIEX, USA). Protein identification and quantification were performed using ProteinPilot 4.5 software^72^. The correlation of quantitative results was evaluated using Pearson algorithm. The high abundance proteins (HAPs) were determined with the first 5% proteins ranked from high to low based on the abundance in luminous organs. The different abundance proteins (DAPs) were defined with a fold-change (FC) ≥ 2 or ≤ 0.5 and a *P* value ≤ 0.05 (*t*-test of all comparison groups). The R package (https://www.r-project.org/) was used for statistical expression data and visualization.

### Bioluminescence gene families and pathways

To explore the origin of bioluminescence in fireflies, we summarized and expanded the pathway of metabolism of luciferin, the emitter of light, which only exist in luminous insects^6^, and investigated the candidate genes in the expanded luciferin metabolism (especially its biosynthesis) in the genomes of luminous beetles and non-luminous beetles (Supplementary Note 7). To explore the possible molecular mechanism of flash on/off and their difference between taxa as well as the possible contribution of calcium to flash control, we thoroughly investigate the candidate genes in the pathways of cAMP/PKA-Ca/Calmodulin signaling cascade and related calcium signaling (Supplementary Note 9). The expression of these candidate genes identified in luminous organs was analyzed using R software based on the FPKM values calculated using Cufflinks. The phylogenetic trees were constructed using RAxML^63^ with maximum likelihood method.

### Microsynteny analysis

To explore the homologous systemic blocks in luciferin biosynthesis (major candidate genes), we performed the identification of genome-wide syntenic and collinear blocks across the six luminous species (Lampyridae: *L. yunnana*, *A. terminalis*, *A. lateralis*, *P. pyralis* and *P. pectoralis*; Elateridae: *I. luminosus*). First, the database of protein similarity was obtained based on all-to-all Blastp (-evalue 1e-10, -num_alignments 20) of the translated protein sequences from six luminous beetles. Second, we used the Multiple Collinearity Scan (Mcscan) (Mcscan toolkit version 1.1, 2016) with more than 3 homologous gene pairs per block to identify conserved collinear blocks, generating a syntenic or collinear block database across all of six species. To perform synteny analysis, we searched and located the target genes along the collinear blocks with the flanking genes surrounding up-200 kb and down-100/200 kb genomic regions as well as the counterparts from different genome. In addition, for the target genes and their flanking genes absent in collinear blocks, we manually scanned the protein similarity database and regarded the gene pairs from different species with more than 50% identity and 80% coverage as the synteny. The syntenic relationships of genes in luciferin biosynthesis and their flanking genes between six luminous species were visualized using Mcscan (https://github.com/tanghaibao/jcvi/wiki/MCscan-%28Pythonversion%29#dependencies).

### Functional verification of genes in luciferin deracemization in vitro

The coding sequences of the firefly luciferase (LUC), alpha-methyl-acyl-CoA-racemase (AMACR) and acyl-CoA thioesterases (ACOT) of *A. terminalis* were synthesized and separately constructed into pET-28a vector (Takara, Japan). ACOTs were further subcloned into pCold-TF vector (Takara, Japan) because they failed to be well expressed in pET-28a vector. The LUC, AMACR and ACOT were expressed in *E. coli* BL21(DE3) at 15 ℃. Then, the proteins were purified using nicke initrilotriacetic acid (Ni^2+^-NTA) column (Qiagen, Germany) and used for the following experiments.

The in vitro deracemization reaction mixture (200 μL) contained 0.1 mM l-luciferin, 8 mM MgSO_4_, 3 mM ATP^.^H2, 0.5 mM COASH, and each (1 µg) of enzymes (LUC, ACOTs, AMACR) in 100 mM Tris–HCl (pH = 8.0). The reaction time was 45 min at 30 ℃. The chirality of luciferin was monitored by high-performance liquid chromatography (HPLC) system (Alliance HPLC System with 2695 Separation Module, 2475 Multi k Fluorescence Detector, Waters) using a chiral fused silica column (Chiralcel OD-RH, 4.6 × 150 mm; Daicel Chemical Industry, Tokyo, Japan). d-luciferin and l-luciferin were detected with a fluorescence detector (excitation k = 330 nm, emission k = 530 nm) (Supplementary Note 7). Relative light units (RLU) of the reaction mixture were measured using a Luminescencer Octa AB-2270 (ATTO, Tokyo, Japan). During 20 s, the integrated activity was described by relative light unit (RLU) (Supplementary Note 7).

### Gene editing in firefly using CRISPR/Cas9 system

Considering that Hox gene *Abd-B* is related to luminous organ development^73^, we selected the homeobox region of the *Abd-B* gene to perform CRISPR/Cas9 gene editing^74,75^ in *A. terminalis*. Target site selection and sgRNA preparation mainly follow the methodology in our previous studies^54^ (Supplementary Note 8.1, Supplementary Fig. S61). Recombinant Cas9 protein (PNA Bio Inc, CA, USA) was used.

During the day, females and males collected from the wild were reared in covered plastic boxes (16 × 10 × 5 cm) padded with a wet paper napkin in an incubator at 25–27 °C and 70% relative humidity. After 0 o’clock in the evening, females were moved into a new covered plastic box (16 × 10 × 5 cm) containing mosses with sufficient humidity and completely dark for oviposition of 7–8 h. In the next morning, fresh *A. terminalis* eggs were collected from the soaked moss by repeated pipetting with a pipette, and then pipetted and arranged on a microscope slide (25.4 × 76.2 × 1–1.2 mm). We injected ~ 2 nl of the mixture of sgRNAs and Cas9 protein (PNA Bio, CA, USA) into each egg under a dissecting microscope (SMZ 800, Nikon, Japan) using a TransferMan NK2 equipped with a TwinTip-Holder and FemtoJet microinjection system (Eppendorf, Germany) at 16–18 °C. Injection needles were made of glass capillary (100 × 1 × 0.6 mm, BJ-40, Zheng-Tian-Yi, Beijing, China) by Narishige PN 30 (Japan) under the parameters: Heater: 80 °C; Magnet Sub: 40 °C; Magnet Main: 50 °C. Optimally, egg injection should be undertaken as early as possible, e.g., at the “one nucleus” stage. Based on the hatching time of about two weeks for *A. terminalis* eggs, all experiment steps, from egg laying to injection, should be finished within 10 h after egg laying (AEL). After injection, the eggs on the slides were carefully washed into covered plastic boxes (16 × 10 × 5 cm) padded with a wet paper napkin, and then placed in an incubator at 25–27 °C and 70% relative humidity. The hatched larvae (generation 0, G0) were carefully moved to clean plastic box padded with wet paper napkin with chopped *Tenebrio molitors* larvae as food. The phenotype of G0 larva was carefully checked especially in the abdomen using microscope SMZ 645 and SMZ18 (Nikon, Japan). The morphologically abnormal individuals and the un-injected wild type individuals were photographed using an AMZ 100 system with a digital camera (Nikon, Japan).

Genotyping was carried out for single or mixed injected larvae (Supplementary Note 8). Genomic DNA extraction from the whole body of single or mixed larvae and subsequent PCR were carried out using TransDirect Animal Tissue PCR kit (TransGen, Beijing, China) following the manufacturer’s instructions. The primer pairs were the same as mentioned above, i.e., LT07795_ex2-F1/R1 for second exon and LT07795_ex3-F3/R3 third exon (Supplementary Fig. 64). The PCR products of the target sites were cloned into pMD-19 T (Takara, Japan), and 10 clones for each sample were selected for Sanger sequencing. Successfully sequenced data were aligned and analyzed using Lasergene SeqMan Pro software (version 7.1) (DNASTAR).

Mixed 14 mutants and mixed 14 wild type larvae were used for total RNA extraction, respectively, and RNA sequencing according to methodology above description (Supplementary Note 8.3). The clean reads were first mapped to the de novo assembled genome of *A. terminalis* using Tophat^69^, and then used to calculate FPKM values and analyze the differentially expressed genes (DEGs) between mutant larva and wild type using Cufflinks software package^70^.

In addition, we also tried CRISPR/Cas9 gene editing for some genes in proposed biosynthesis pathway, i.e. luciferase. However, due to such natures of fireflies as long life circle, difficulty to rear in large scale in the laboratory etc., we still couldn’t obtain enough injected larvae for phenotyping at current time. More technologies about firefly raise in large scale still need to develop and more improved gene editing experiments need to be performed for further testing the function of these genes.

## Supplementary information

Supplementary file

Supplementary file

Supplementary file

Supplementary file

Supplementary file

Supplementary file

## Data Availability

The genome assemblies and sequence data, RNA-seq data for *Lamprigera yunnana* and *Abscontida terminalis* were deposited at NCBI under BioProject accession number PRJNA556754 and PRJNA556938, respectively. The quantitative proteome data for *L. yunnana* and *A. terminalis* were deposited at iProX under Project accession number IPX0001742000 (PXD015226) and IPX0001743000 (PXD015227), respectively.
