# Supplementary material for: Genomic and experimental data provide new insights into luciferin biosynthesis and bioluminescence evolution in fireflies
Source: Sci Rep. 2020 Sep 28;10:15882. doi: 10.1038/s41598-020-72900-z (PMC7522259; doi:10.1038/s41598-020-72900-z)
Supplement: Supplementary file 6 — Supplementary file [file 41598_2020_72900_MOESM6_ESM.pdf]

Genomic and experimental data provide new insights into luciferin biosynthesis and bioluminescence evolution in fireflies

Ru Zhang[1, 2, †], Jinwu He[1, 2,†], Zhiwei Dong[2, †], Guichun Liu[2, †], Yuan Yin[1, †], Xinying Zhang[3, †], Qi Li[1, †], Yandong Ren[2], Yongzhi Yang[1], Wei Liu[2], Xianqing Chen[1], Wenhao Xia[1], Kang Duan[2], Fei Hao[1], Zeshan Lin[1], Jie Yang[1], Zhou Chang[2], Ruoping Zhao[2], Wenting Wan[1, 2], Sihan Lu[1], Yanqiong Peng[4], Siqin Ge[3, *], Wen Wang[1, 2,5,*], Xueyan Li[2,*]

[1] School of Ecology and Environment, Northwestern Polytechnical University, Xi'an, Shaanxi 710072, China.

[2] State Key Laboratory of Genetic Resources and Evolution, Kunming Institute of Zoology, Chinese Academy of Sciences, Kunming, Yunnan 650223, China.

[3] Institute of Zoology, Chinese Academy of Sciences, Beijing, China.

[4] CAS Key Laboratory of Tropical Forest Ecology, Xishuangbanna Tropical Botanical Garden, Chinese Academy of Sciences, Mengla, Yunnan 666303, China.

[5] Center for Excellence in Animal Evolution and Genetics, Kunming, Yunnan 650223, China.

Supplementary Note 1. Species background

Fireflies (Coleoptera: Lampyridae), the most well-known luminous terrestrial animals, include about 2000 species worldwide. Their luminescence plays an important role in sexual communication of adults. Generally, two luminous patterns, which are glow and flash, exist in different taxa. In order to explore the genomic basis underlying bioluminescence origin and evolution, we dissected the genomes of the representative species of each luminous pattern, *Lamprigera yunnana* (Lyu) (glow) and *Abscondita terminalis* (Ate) (flash).

S1.1 Species identification

Identification of male *L. yunnana* was referred to the morphological redescription [1] and the original morphological description of type specimens [2]. Identification of *A. terminalis* was referred to the morphological redescription of type specimens [3]. Morphology was observed using the microscope SMZ1000 (Nikon, Japan) and photos were taken using Canon 700D. The specimens of male genitalia were obtained after being treated for 8-10 hours in the 10% NaOH solution at room temperature, and their pictures were taken using AMZ100 (Nikon, Japan).

L. yunnana male is alate, but its female is larviform (Figure S1a-e; Figure S2a-c). Our observation on their mating success confirms the same species of these males and females. In taxonomy, *L. yunnana* was originally placed the subfamily Lampyrinae [4, 5], but recently was changed to *incertae sedis* in Lampryidae [1, 6, 7]. Both males and females of *A. terminalis* are alate (Figure S1f-l; Figure S2d-f). *A. terminalis* is the typical representative of the subfamily Luciolinae [3].

S1.2 Morphology and structure of luminous organs

S1.2.1 Outer morphology of luminous organs

Outer morphology of luminous organs was observed using AMZ100 (Nikon, Japan). Firefly bioluminescence are produced by the luminous organs, which usually locate at the 8th sternite (= the 8th ventrite) of the larva but are changeable in adults of different taxa and different sex (Figure S3). Larviform female of *L. yunnana* has luminous organs on its 8th sternite-pleurite (= the 8th ventrite-pleurite), while luminous organs of pterygote male are in its last ventrite (the 7th ventrite = the 8th sternite) (Figure S3). Male luminous organs of pterygote *A. terminalis* occupy the last two ventrites (the 5th and 6th ventrites = the 6th and 7th sternites), while female

luminous organ is only in its penultimate ventrite (the 5th ventrite = the 6th sternite) (Figure S3).

S1.2.2 Inner structure of luminous organs

In order to investigate the difference of luminous organs between *L. yunnana* and *A. terminalis*, we dissected the ventrites of adults. The firefly samples were dissected in phosphate buffer saline (PBS) and then fixed for 2 hours at 0°C using 3% aceticacid to dissolve the lipochondrion. The inner structures of firefly luminous organs were observed using a NIKON Y-SMZ25 microscope and photos were taken by a Canon EOS 70D SLR cameral equipped with Canon EF 100mm f2.8 Macro lens from dorsal view.

From dorsal view, *L. yunnana* male has two stacks of clumped light organs so that mainly underside stack in the 8th sternite can be observed in abdomen from ventral view and one main trachea passes through between two stacks of light organs (Figure S4a). *L. yunnana* female also has two stacks of similar luminous organs to those of male in the 8th sternite (Figure S4b). Both male and female of *L. yunnana* show similar abdominal nerve and ganglion with non-luminous ventrites, but the ventrites of luminous organs have no ganglion and their nerves are branched from the ganglion ahead of luminous ventrites (Figure S4e, f). Unlike *L. yunnana*, male adult of *A. terminalis* was observed to have two laminated milky luminous organs occupying the 6th and 7th sternites, and developed trachea system specially into luminous organs (Figure S4c), while its female adult has only one light laminated organ occupying in the 6th sternite and less developed trachea system than those of male even in luminous organ (Figure S4d). Both male and female of *A. terminalis* show similar abdominal nerve and ganglion, locating under the trachea system and extending to the end of abdomen and branched into luminous organs (Figure S4c, d).

S1.2.3 Three-dimension reconstruction of luminous organs of adults

The specimens of male adults were used for micro-CT scanning. They were dehydrated with an ethanol/acetone series (75–80–85–90–100%-acetone) and dried at the critical point (HCP-2, Hitachi Ltd., Japan). The experiments were performed at X-ray imaging and biomedical application beam-line (BL13W1) of Shanghai Synchrotron Radiation Facility (SSRF). The electron beam energy in the storage ring was 3.5GeV. The beam intensity was at 200 mA. The monochromatic light energy was selected 10 keV. The exposure time was 2s. The resolutions of the output image were 3.25 μm.

Abdominal structures of all species were reconstructed with Amira 6.0 (Visage Imaging, Berlin, Germany). The data files were then transferred to VGSTUDIO MAX 3.0 (Volume Graphics, Heidelberg, Germany) to perform the volume rendering. Final images were assembled and arranged with Adobe Photoshop and Illustrator (Adobe Inc., CA, USA).

In *L. yunnana* male, two small, U-shaped light organs are located at two sides of the sternite 7, respectively, and two tracheae tubes inserted into light organs (Figure S5a, b, c). In *L. yunnana* female, two small, donut-shaped light organs are located at two sides of the sternite 8 with strong tracheae tubes pass through light organs (Figure S5d, e, f). In *A. terminalis* male, the flattened-plate-shaped light organ completely fills the inner surface of the sternites 6 and 7, tracheae lay on the light organ in dorsal view (Figure S5g, h, i). In *A. terminalis* female, flattened-plate-shaped the light organ occurs and nearly fill at the inner surface of the sternite 6 and very strongly developed tracheae lay on the light organ in dorsal view (Figure S5j, k, l).

S1.2.4 The structures of luminous organs observed with semi-thin section and Transmission Electron Microscope (TEM)

In order to investigate the structural difference of luminous organs between *L. yunnana* and *A. terminalis*, semi-thin section and transmission electron microscope (TEM) technology with the help of Kunming Biological Diversity Regional Center of Instrunents and Kunming Medical University were used to observe luminous organs. Firstly, we carefully dissect the luminous organs in PBS and the separated luminous samples were fixed overnight at 4 °C using 1% glutaraldehyde and 3% paraformaldehyde in 0.2M $Na_2HPO_4{\bullet}2H_2O$ (pH7.4). Afterward, the samples were postfixed with 1% osmium tetroxide (OsO4) for 2 h at 4 °C, followed by serial ethanol dehydration and embedding in LR White resin. Part of postfixed samples were used for semi-thin section. Serial semi-thin sections of uniform thicknesses of about 800 nm were made using an ultramicrotome leica EM UC7, stained in 1% toluidine blue, and observed employing a NIKON Y-FL microscope. A part of postfixed samples was used for ultra-thin section. Serial ultra-thin sections of uniform thicknesses of about 60 nm were made using an ultramicrotome leica EM UC7, then loaded onto 100-mesh Cu grids and double stained with 2% uranyl acetate and lead citrate before observations employing a JEM 14000plus transmission electron microscope at 120 kv.

No obvious stratification of reflecting layer and photogenic layer was observed in the semi-thin section of luminous organs of both male and female of *L. yunnana* (Fig. 1M-N), while obvious reflecting layer and photogenic layer with similar thickness can be observed in the semi-thin section of luminous organs of both male and female of *A. terminalis* (Fig. 1O-P). Further data from ultra-thin section show that a very thinner reflecting layer than photogenic layer, many thick trachea and highly developed tracheoles in photogenic layer exist in luminous organs of both male and female of *L. yunnana* (Figure S6a-h), while in luminous organs of both male and female of *A. terminalis*, there are compact urate vesicles and developed trachea and tracheoles in the reflecting layer adjoining the photogenic layer (Figure S6i-p).

S1.3 Luminous behavior and characteristics

Luminous behavior was observed by naked eyes when specimens were being collecting in the field. Luminous behavior was photoed or recorded by a Canon EOS 70D SLR cameral equipped with Canon EF 100mm f2.8 Macro lens when in the room or a transparent container. Live fireflies were brought back to lab to measure their spectrum. Single live firefly was wrapped in plastic film and then directly placed into sample holder for spectrum measurement using a LumiFL spectrocapture AB1850 (ATTO, Japan). For both species, four males and four females were used for spectrum measurement.

The males keep their luminous organs completely off during their flying, but can produce continuously green light when perching near females before mating from their two spot-like light organs of the ventral side of the 7th abdominal segment (Figure S7a and Video S1). The emission spectrum of both male and female showed a peak at 567 nm (Figure S8). Female of *L. yunnana* can produce continuously strong yellow-green light from their two spot-like light organs of the ventral side of the 8th abdominal segment at night and even in a dimmed room during the daytime (Figure S7b and Video S2). For *A. terminalis*, both male and females flash green-yellow (Figure S7c-d and Video S3-S4) but with a slight difference (2 nm) of maximal emission spectrum (female: $\lambda max=577$ nm; male: $\lambda max=575$ nm) (Figure S8).

Supplementary Note 2. Genome sequencing and *de novo* assembly

S2.1 Genome survey using Illumina sequencing

In order to understand rough properties of two genomes such as heterozygosity and genome size, we first made genome survey for two species. A total of 105.15 Gb and 91.74 Gb clean data were generated in *L. yunnana* and *A. terminalis*, respectively

(Table S1), of which partial data were used for *k-mer* frequency analyses (Table S2). A bimodal distribution is observed for both *L. yunnana* and *A. terminalis* in which the depth of the first peak is about half of that of the second peak (Figure S9). In bimodal model, the first peak denotes the depth of heterozygosity, and the second peak is the average depth of *k-mer*. We further used Illumina short reads to calculate the level of heterozygosity of these two firefly genomes, and our results indicated that both of them show high heterozygosities (*L. yunnana*: 1.11%; *A. terminalis*: 1.52%) (Table S2). *K-mer* analysis can also be used to calculate a genome size (Gb) by the formula of G=K_num/K_depth, where the K_num is the total number of *k-mer* and K_depth is the average depth of *k-mer*. The genome sizes of *L. yunnana* and *A. terminalis* are estimated as about 1094 Megabase (Mb) and 626 Mb, respectively (Table S2).

S2.2 PacBio genome sequencing and *de novo* assembly

In total, 81.08 Gb (74 ×) and 42.21 Gb (67 ×) SMRT (Single-Molecule Real Time) data from PacBio RS II platform were generated for *L. yunnana* and *A. terminalis*, respectively (Table S3). The length distribution was shown in Figure S10. The average read length and N50 are 8.12 kb and 11.45 kb for *L. yunnana*, 7.19 kb and 11.11 kb for *A. terminalis* (Table S3). These SMRT subreads in FASTA files were pooled together for genome assembly. Considering the high heterozygosity of the two species (Table S2), we chose a fuzzy Bruijn graph (FBG) approach in a long noisy reads assembler called wtdbg (https://github.com/ruanjue/wtdbg), which has great advantages in *de novo* assembly of high-complexity genomes. Similar to FALCON [8], wtdbg only relies on SMRT long reads for assembly. However, wtdbg has a few unique characteristics. Firstly, two rounds polishing can be performed for assembled genome using wtdbg. Furthermore, wtdbg integrates an alignment software called minimap, a read overlapper and also a read-to-genome and genome-to-genome mapper, so that it can process larger dataset at a faster speed [9].

For *L. yunnana*, the assembled genome size and contig N50 were 1053 Mb and 3.51 Mb, respectively; for *A. terminalis*, the genome size and contig N50 were 501 Mb and 1.21 Mb, respectively (Table S4). The assembled genome size of *L. yunnana* (96.2% of that estimated by 17-*kmer* analysis) was two times than that of *A. terminalis* (80.0% of that estimated by 17-*kmer* analysis) (Supplementary Tables 2 and 4).

S2.3 Assembly quality evaluation

S2.3.1 Align Illumina short reads to evaluate the completeness of assemblies

To assess the assembly quality, Illumina reads from short insert size library (350 bp) with high quality were mapped onto the final assembly using BWA [10] with default parameters. The Samtools software [11] was used to count the mapping rate. In *L. yunnana*, 98.58% of the reads were successfully mapped back to the final assembly with 92.06% of properly paired (PE) mapped ratio; in *A. terminalis*, 98.51% of the reads were successfully mapped back to the final assembly with 89.95% PE mapped ratio. Our results show that the final assemblies of *L. yunnana* and *A. terminalis* contain almost all of the information in reads (Table S5).

S2.3.2 Evaluate the completeness of assemblies by Benchmarking Universal Single-Copy Orthologs (BUSCO)

To evaluate the completeness of gene regions of the assembly, we applied Benchmarking Universal Single-Copy Orthologs (BUSCO) [12] to align the assembled genome to 1658 BUSCOs from Insecta_odb9 Dataset with default settings. The proportion of the complete BUSCOs was 98.6% and 97.8% in the assembled genomes of *L. yunnana* and *A. terminalis*, respectively (Table S6), which is comparable with that of another firefly *Pyrocoelia pectoralis* (Ppe) [13] and higher than two fireflies *Photinus pyralis* (Ppy) and *Aquatica lateralis* (Ala) [14]. The results indicate that the assemblies have admirable integrity.

S2.3.3 Align transcripts to evaluate the completeness of assemblies

In addition, to better assess the assembly quality, RNA-reads were mapped to the assembled genomes using Tophat software [15] with default settings. The read mapping rates of RNA-seq libraries from three developmental stages (larvae, female adult and male adult) of *L. yunnana* ranged from 91.6% to 92.0%, while the mapping rates of the RNA reads from five developmental stages (female pupa, male pupa, larvae, female adult and male adult) of *A. terminalis* ranged from 81.6% to 88.8% (Table S7), indicating consistency between the genome and the sequenced transcriptome.

Supplementary Note 3. Genome annotation

S3.1 Repetitive element annotation

S3.1.1 Annotation method of repetitive elements

The repetitive elements (repeat, or repeated sequences) included transposable elements (TEs) and tandem repeats. Repeat annotation was performed by combining a *de novo*-based prediction with a homology-based approach. RepeatModeler

(http://www.repeatmasker.org/RepeatModeler/) was used to *de novo* identify and classify repeat families. Homology-based approach was used to identify known TEs by searching assembled genomes against TE Databases at both DNA and protein levels. RepeatMasker was employed to identify the DNA-level TEs against the Repbase TE library 21.04 (http://www.girinst.org/repbase) [16], and RepeatProteinMask [17] was used to identify the protein-level TEs with -no LowSimple -p value 0.0001 parameters. In addition, tandem repeats were discovered using the Tandem Repeat Finder (TRF) program [18] with 2 7 7 80 10 50 2000 –d –h parameters. The annotated repeats using different methods were shown in Table S8. Our results show that the genome of *L. yunnana* has a higher percentage of repetitive elements (66.62%) than that of *A. terminalis* (36.54%).

S3.1.2 TEs content

Different categories of TEs in two firefly genomes were counted using an in-house script. The proportion of TEs was 65.36% and 33.76% in the assembled genomes of *L. yunnana* and *A. terminalis*, respectively (Table S9). The most abundant TEs in assembled genomes of *L. yunnana* and *A. terminalis* is DNA transposons (Table S9), consistent with that of three firefly previously reported [13, 14]. Furthermore, the percentage of DNA transposons in *L. yunnana* (34.91%) was nearly two times than that of *A. terminalis* (17.62%).

To illuminate the reason underling the variation of genome size especially between *L. yunnana* and *A. terminalis*, we divided the whole genome of five firefly species into seven categories based on our annotated results (repeat annotation and following gene annotation), including coding sequence (CDS), five types TEs (DNA transposons, long interspersed nuclear elements (LINEs), short interspersed nuclear elements (SINEs), long terminal repeats（LTR) and Unknown) and Others (the rest of the whole genome except for CDS and TEs). Our results indicate that the obvious difference results from the content of TEs, especially DNA transposons and LINEs, which also occupy the largest proportion of total TEs in other firefly species (Table S9). DNA transposons is the most abundant type of TEs in the assembled genomes of *L. yunnana* and *A. terminalis* (Table S9 and Figure S11) and other luminous beetles (*P. pyralis*, *P. pectoralis*, *A. lateralis*, *I. luminosus*) [19, 20], with a two-fold variation between the highest in *L. yunnana* (34.91%) and the lowest in *A. terminalis* (17.62%) (Table S9). The following abundant TEs type in both *L. yunnana* and *A. terminalis* are LINEs (Table S9). We further calculated the number and length of different superfamilies of DNA transposons and LINEs among five firefly species using in-house script. Our results (Figure S11) demonstrate that the DNA, TcMar, hAT and

Maverick superfamilies of DNA transposons and the LINE, Penelope, RTE, Jockey and R1 superfamilies of LINEs exhibited a similar variation pattern to that of their host genome size, suggesting the important contribution of these TE superfamilies to genome size variation.

S3.2 Gene annotation and functional annotation

Gene model identification was conducted by a combination of *de novo* prediction, homology-based prediction and transcriptome-based prediction methods using EvidenceModeler (EVM) [21]. The *de novo* assembled transcriptomes of both species were mapped to their assembled genomes using the Program to Assembled Spliced Alignments (PASA) [22] to generate the PASA full length cDNA set that was used to train the *ab initio* gene prediction programs. The Augustus program [23] were executed to predict coding regions in the repeat-masked genomes. RNA reads sequenced from the whole body were mapped to the assembled genome using Tophat2 [15]. Cufflinks [24] was used to assemble the non-redundant transcripts, predicted into gene models by Transdecoder [25] that was remained with the fragments per kilobase of exon per million fragments mapped (FPKM) $\geq$1. In addition, The assembled transcriptome using PASA [22] was mapped the assembled genomes to obtain the PASA-blat set by the BLAT alignment tool [26]. Protein data sets of fruit fly *Drosophila melanogaster* (Dme), red flour beetle *Tribolium castaneum* (Tca), Asian longhorned beetle *Anoplophora glabripennis* (Agl), burying beetle *Nicrophorus vespilloides* (Nvi), and mountain pine beetle *Dendroctonus ponderosae* (Dpo) were aligned against the assembled genomes of *L. yunnana* and *A. terminalis* using TblastN [27] (E-value = 1e-5). The BLAST hits were conjoined using Solar software (The Beijing Genomics Institute (BGI) development). GeneWise [28] was applied to predict the exact gene structure of the corresponding genomic regions for each BLAST hit. These gene models were integrated by combing all pieces of evidence into a non-redundant consensus set of gene prediction using EVM.

The consensus gene set was further updated using two rounds of PASA to obtain improved genes and gene structure. Furthermore, the proteins missed in BUSCO for updated gene models were used to annotate the genome, donated as BUSCO set, added to the updated gene models. Then, the updated gene models were filtered using four criteria: (a) remove genes with coding region length less than 150 bp, (b) discard single exon genes not supported by the transcripts, (c) remove genes with incorrect start and stop codons and its inner with termination codons, (d) remain the longest coding genes with splice junctions. The final gene models (Table S10) were generated for further analysis.

The completeness of the proteins of the predicted genes were evaluated using BUSCO, reaching to 96.3% in *L. yunnana* and 97.0% in *A. terminalis*, respectively (Table S11). Moreover, we compared gene features including the distribution of mRNA length, CDS length, exon length, intron length and the exon number among *L. yunnana*, *A. terminalis*, *A. lateralis*, *P. pyralis*, *P. pectoralis*, *I. luminosus*, *A. glabripennis*, *D. ponderosae*, *T. Castaneum* and *D. melanogaster*. Our results indicated high-confident gene models in the two assembled genomes (Table S12; Figure S12). In conclusion, 19,443 and 21,024 genes are finally predicted in *L. yunnana* and *A. terminalis*, respectively (Table S13). Ultimately, 15,914 functional genes were identified, occupying 81.85% of the predicted genes in *L. yunnana*, while 18,698 functional genes were identified, representing 88.94% of the predicted genes in *A. terminalis* (Table S13).

Supplementary Note 4. Phylogenic analysis of Elateroidea beetles

To analyze the phylogeny of Elateroidea beetles and bioluminescence distribution in phylogenetic context, we performed phylogenomic analysis for all beetles in Elateroidea and other superfamilies with their reference genomes available based on their orthologous genes. Considering still no reference genomes available for other non-luminous families in Elateroidea, we also made additional phylogenetic analysis based on mitogenomes for most Elateroidea families with their mitogenome available.

S4.1 Orthologous gene tree

Combined with the genomes of two fireflies (*L. yunnana* and *A. terminalis*) sequenced in this study, total 15 beetles (6 luminous taxa in Elateroidea (5 fireflies, 1 luminous click beetles) and other nine non-luminous beetles outside Elateroidea) with genomes available were included in the phylogenetic analysis. The protein sequences of 13 beetles and one fruit fly were downloaded from Databases: one firefly *P. pectoralis* from GigaDB (http://gigadb.org/), two another fireflies (*P. pyralis, A. lateralis*) and one click beetle *I. luminosus* from fireflybase (http://www.fireflybase.org), other nine non-luminous beetles (*A. glabripennis*, *Agrilus planipennis*, *D. ponderosae*, *Onthophagus taurus*, *Asbolus verrucosus*, *Leptinotarsa decemlineata*, *Diabrotica virgifera*, *Nicrophorus vespilloides*, *T. castaneum*) and one fruit fly *D. melanogaster* from GenBank. 531 single-copy orthologs were identified using the OrthoMCL [29] method on the all-versus-all *BLASTP* (E-value ≤ 1e-5) alignments, and their coding DNA sequences (CDS) were aligned based on the protein alignment using MUSCLE [30] and PAL2NAL [31] software, and further trimmed using Gblocks v0.91b [32] (-t=c -b4=5). The phylogenetic tree of whole genomes was constructed using the RAxML

[33] software with the GTR+gamma model with *D. melanogaster* as an outgroup, and visualized using FigTree [34]. The divergence times were estimated using the PAML v4.8 [35] mcmctree program with the approximate likelihood calculation method, correlated molecular clock model and REV substitution model. After a burn-in of 50,000 iterations, the MCMC process was performed 20,000 times with sample frequency of 5,000. Finally, the MCMC convergence was checked using Tracer [36] and confirmed with two independent runs. The constraints that *Drosophila* and Coleoptera split around 275-345 million years ago (MYA) and the species within Coleoptera diverged around 271-300 MYA (http://www.timetree.org/) were used for time calibrations.

S4.2 Mitochondrial genome tree

The 13 protein-coding genes (PCGs) of mitogenomes from 39 Elateroidea species in 11 families of Elateroidea and of *T. castaneum* (Tenebrionoidea: Tenebrionidae) were downloaded from GenBank (Table S14). In order to conserve reading frames, the 13 PCGs were first aligned separately based on the amino-acid translation with MEGA-X [37], and then back translation. All resulting alignments were then concatenated using SequenceMatrix 1.7.8 [38]. The best-fit partitioning scheme and model of molecular evolution were inferred with PartitionFinder [39]. A Bayesian analysis was conducted using Mrbayes 3.2 [40]
with 10,000,000 generations and burn-in of 25% of the sampled trees.
The final tree was visualized and modified by iTOL [41].

Supplementary Note 5. Gene evolution

S5.1 Gene family clusters

Combined with the genome data of two fireflies (*L. yunnana* and *A. terminalis*) in this study, 21 species (20 insects and one crustacea) were used to cluster gene families with one crustacea (*Daphnia pulex*) as outgroup (Table S15). These 20 insects include 11 of 15 beetles above mentioned in phylogenetic analysis of whole genomes and another 8 non-beetle insects in six orders (Lepidoptera: *Bombyx mori*, *Papilio machaon*, *Papilio xuthus*; Diptera: *D. melanogaster*; Hymenoptera: *Apis mellifera*, *Nasonia vitripennis*; Hemiptera: *Acyrthosiphon pisum*; Phthiraptera: *Pediculus humanus*; Isoptera: *Zootermopsis nevadensis*). The excluded four beetles are *A. verrucosus*, *L. decemlineata*, *D. virgifera*, *N. vespilloides*, which have the poor assembly and annotation of their genomes compared to other beetle genomes. Except luminous beetles as described above, the genomes of all other species were downloaded from GenBank. Gene sets were filtered by selecting the longest

open-reading frame (ORF) for each gene. ORFs with premature stop codons, non-triple length, or fewer than 30 amino acids were removed. Gene families were constructed using the OrthoMCL v2.0.9 [29] method on the all-versus-all *BLASTP* (E-value ≤ 1e-5) alignments.

Totally, 366,315 genes from 21 species were clustered into 208,284 gene families (Table S15). *L. yunnana* has 10,233 families and *A. terminalis* has 11,189 gene families (Fig. 3a; Table S15). Among them, 77 gene families (668 genes: Ala, 98 genes; Ate, 99 genes; Lyu, 105 genes; Ppe, 132 genes; Ppy, 103 genes; Ilu, 131 genes) are specific for Lampyridae-Elateridae clade (shared only in five fireflies and luminous click beetle Ilu). The GO enrichment analyses of these specific genes in Lampyridae-Elateridae suggested that some of them were related to catalytic activity and smell (odorant binding) (Table S16); their KEGG enrichment suggested that some of them were related to signal transduction, metabolism of cofactors and vitamins, phenylpropanoid biosynthesis and phenylalanine metabolism (Table S17). 152 gene families (1121 genes: Lyu, 193 genes; Ate, 220 genes; Ala, 197 genes; Ppy, 222 genes; Ppe, 289 genes; 44 single-copy orthologs (1:1:1)) are Lampyridae-specific (shared only five firefly species Lyu, Ate, Ala, Ppy and Ppe). The GO enrichment analyses of these genes in Lampyridae-specific families suggested that some of them were related to amine and polyamine metabolic process and smell (odorant binding) (Table S18) and their KEGG enrichment showed that some of them were related to biosynthesis and metabolism of some amino acids, inositol phosphate metabolism and phenylpropanoid biosynthesis (Table S19). 359 gene families (Ppy: 497 genes; Ppe: 985 genes) are Lampyrinae-specific that only shared in *P. pyralis* and *P. pectoralis* species, which was enriched mainly in aspects of odorant binding, sulfotransferase activity, monooxygenase activity and olfactory receptor activity by GO enrichment (Table S20) and biosynthetic pathway like ubiquinone and other terpenoid-quinone, phenylpropanoid and steroid by KEGG enrichment (Table S21). 189 gene families (Ate: 477 genes; Ala: 368 genes) are Luciolinae-specific that only shared in *A. lateralis* and *A. terminalis* species, which was enriched mainly in aspects of odorant binding, oxidoreduction coenzyme metabolic process and quinone metabolism and biosynthesis by GO enrichment (Table S22), and pathways of peroxisome, ubiquinone and other terpenoid-quinone biosynthesis and insect hormone biosynthesis by KEGG enrichment (Table S23). *L. yunnana* has 438 species-specific families (3247 genes), while *A. terminalis* has 658 species-specific families (2261 genes). Combined with unclustered genes, *L. yunnana* and *A. terminalis* have 6027 and 7729 species-specific genes, respectively (Table S15), of which 2352 genes and 1958 genes without predicted function in *L. yunnana* and *A. terminalis*, respectively. The GO enrichment analyses of *L. yunnana* specific genes suggested that most genes are related to binding,

quinone metabolism and sulfotransferase activity (Table S24). The GO enrichment analyses of *A. terminalis* specific genes suggested that many genes are related to oxidation-reduction process, transport (biological process), catalytic activity and sulfotransferase activity, oxidoreductase activity etc. (molecular function) (Data S1).

S5.2 Divergence time of Coleoptera and the gene family expansion/contraction

285 single-copy orthologous genes identified within the 21 species were used to build a phylogenetic tree, and divergence times were estimated using the same method as described in phylogenetic tree of whole genomes (Fig. 3b). The following constraints were used for time calibrations: (1) 476-541 million years ago (MYA) for the Insecta and Branchiopoda split (Misof *et al.* [42]); (2) 317-372 MYA for Hymenoptera and Coleoptera split (Misof *et al.*[42] and http://www.timetree.org/); (3) 246-293 MYA for Coleoptera inner species divergence (Misof *et al.*[42], Mckenna *et al.* [43] and http://www.timetree.org/).

To study gene gain and loss, CAFÉ (Computational Analysis of gene Family Evolution) v4.0. 1 [44] software was applied to estimate the universal gene birth and death rate λ (lambda) under a random birth and death model, using the maximum likelihood method. Gene gain and loss results for each branch of the phylogenetic tree were estimated. In addition, GO and KEGG enrichments of genes in gene families that expanded and contracted in the *A. terminalis* and *L. yunnana* lineage were also calculated.

148 families are expanded in the ancestor of Elateridae-Lampyridae beetles. GO (Data S2) and KEGG analyses (Table S25) of these expanded gene families are related to oxidation-reduction process, bioluminescence, peroxisome, ATPase activity, ATP-binding cassette (ABC) transporter etc. 157 families are expanded in the ancestor of Lampyridae. GO (Data S3) and KEGG analyses (Table S26) of these expanded gene families suggested that many of them are related to oxidation-reduction process, transmembrane transport, ATPase activity, fatty acid beta-oxidation and peroxisome. 667 gene families are expanded in the ancestor of Lampyrinae, of which GO (Data S2) and KEGG (Table S27) are related to oxidoreductase activity, ATPase activity, acyl-CoA oxidase activity and ABC transporters. 102 gene families are expanded in the ancestor of Luciolinae, of which GO (Data S2) and KEGG (Table S28) are related to peroxisome, ATPase activity, acyl-CoA oxidase and dehydrogenase activity, phenylalanine metabolism and ABC transporters.

L. yunnana has 780 expanded families and 3,333 contracted families; *A. terminalis* has 942 expanded families and 1,926 contracted families (Fig. 3b). GO analyses of *L. yunnana* expanded suggested that many of gene families are related to bioluminescence, catalytic activity, oxidoreductase activity, transporter activity, odorant binding (Data S4) and KEGG analyses of *L. yunnana* expanded suggested that many of gene families are related to protein digestion and absorption (Table S29). GO analyses of *A. terminalis* expanded suggested that many of gene families are related to binding (nucleic acid, cation, ion, metal ion), transporter, ATPase and odorant binding (Data S4) and KEGG analyses of *A. terminalis* expanded suggested that many of gene families are related to pathways that calcium signaling, peroxisome, ABC transporters and photosynthesis (Table S30).

S5.3 Detection of rapidly evolving genes (REGs) and positively selected genes (PSGs)

11 Coleoptera species used in family clustering were selected to perform the selection analysis and a total of 1,359 single-copy orthologs (each orthologs larger than 90 bp) were identified by SonicParanoid which is a faster, accurate and easy-to-use tool for multi-species ortholog inference [45]. These eleven species with better assembly and annotation include *L. yunnana*, *A. terminalis*, *A. lateralis*, *P. pyralis*, *P. pectoralis*, *I. luminosus*, *A. planipennis*, *A. glabripennis*, *D. ponderosae*, *O. taurus* and *T. castaneum*. Each orthologous gene set was aligned using PRANK [46] with the parameter "-codon" and trimmed using GBlocks v0.91b [32] with the parameter "-t=c -b4=5". We first estimated the values of *Ka*, *Ks* and ω (*Ka*/*Ks*) for the concatenated alignment of 1, 359 single-copy orthologs using the Codeml program of PAML [35] with the free ratio model for each branch. 10000 concatenated alignments constructed from 150 randomly chosen orthologs were used to estimate lineage-specific mean values. The average *Ka*/*Ks* was shown as Figure S13, indicating that most recent common ancestor (MRCA) lineage of firefly showed accelerated *Ka*/*K*s value, while the MRCA lineage of luminous beetles (Luc-Anc) didn't show accelerated *Ka*/*Ks* value**.**

We also identified the rapidly evolving genes (REGs) along with Lampyridae-Elateridae ancestor, Lampyridae ancestor and the two fireflies (*A. terminalis* and *L. yunnana*), with the following criterions. The branch model in the Codeml program of PAML [35] was used to identify REGs with the null model assuming that all branches have been evolving at the same rate and the alternative model allowing foreground branch to evolve under a different rate. The likelihood ratio test (LRT) with df = 1 was used to discriminate between the alternative model

for each orthologous in the gene set. Multiple testing was corrected by applying the false discovery rate (FDR) method implemented in R [47]. We considered the genes as evolving with a significantly faster rate in foreground branch if the adjusted P value less than 0.05 and a higher ω values in the foreground branch than the background branches.

We also identified the positively selected genes (PSGs) along with Lampyridae-Elateridae ancestor, Lampyridae ancestor, Lampyrinae, Luciolinae and the two fireflies (*A. terminalis* and *L. yunnana*), Lampyridae ancestor and luminous beetle ancestor with the following criterions. To detect positive selection on a few codons along specific lineage, we used the optimized branch-site model following the author's recommendation [48]. A LRT was constructed to compare a model that allows sites to be under positive selection on the foreground branch with the null model in which sites may evolve neutrally and under purifying selection. The P values were computed based on the Chi-square statistic adjusted by the FDR method and genes with adjusted P value less than 0.05 were treated as candidates for positive selection.

Our data showed that there are 205 REGs and 192 PSGs in the ancestor of firefly, 46 REGs and 190 PSGs in the ancestor of Lampyridae-Elateridae (Data S5-S6). In the ancestor of Lampyridae-Elateridae beetle, the REGs are mainly related to mitochondrion and ATPase activity, while PSGs are catalytic activity and ATP binding (Data S7). Especially interesting, a Ca^{2+} transporting ATPase (*LY02642*, *LT07457*: Ca^{2+} transporting ATPase) are PSG in the ancestor of not only firefly but also Lampyridae-Elateridae beetle (Data S6). In the ancestor of firefly, these REGs are mainly related to transport (Data S7), and these PSGs are mainly related to catalytic activity and oxidoreductase activity (Data S7). In the ancestor of Luciolinae, 120 genes were PSG which were mainly related to RNA transport and organelle organization (Data S8; Supplementary Tables 31-32). In the ancestor of Lampyrinae, 144 genes were PSG which were mainly related to purine biosynthesis and, cysteine and methionine metabolism (Data S8; Tables S31-S32).

L. yunnana has 242 REGs and 75 PSGs, of which REGs are related to oxidation-reduction process and PSGs are mainly related to RNA metabolic process (Data S9-S10). Some of REGs and PSGs have relatively expression in *L. yunnana* (Figures S14-S17). *A. terminalis* has 149 REGs and 105 PSGs, of which some REGs are related to ATPase and ATP binding, and some PSGs are related to catalytic activity (Data S9-S10). Some of REGs and PSGs have relatively expression at both transcriptomic and proteomic levels in *A. terminalis* (Figures S14-S17).

S5.4 Orthologous relationship between *L. yunnana* and *A. terminalis*

To determine the orthologous relationship between *L. yunnana* and *A. terminalis*, we used all proteins of assembled genomes to perform all-against-all alignment using BLASTP program from BLAST [49] with an E-value cutoff of 1e-5. Reciprocal best hit protein pairs between *L. yunnana* and *A. terminalis* were defined as orthologs. In addition, one to one genes from gene families based on OrthoMCL v2.0.9 [29] between *L. yunnana* and *A. terminalis*, were added and eliminated redundancy. In total, 9,497 orthologs were obtained and used to execute the analysis of comparative transcriptome and proteome between two species.

Supplementary Note 6. Transcriptome and proteome sequencing and analyses

S6.1 Transcriptome sequencing, assembly and analyses

S6.1.1 RNA isolation, library construction, transcriptome sequencing and data clean

In order to assist in assembled genome evaluation, gene prediction and developmental studies, transcriptomes of whole body from different development stages of *L. yunnana* (larva, female adult and male adult) and *A. terminalis* (larva, female pupa, male pupa, female adult, male adult) as well as their adult luminous organs of both sexes were sequenced. *L. yunnana* male and female adults were collected from the wild for RNA extraction, and its young larva was collected and bred in the lab to final instar for RNA extraction. *A. terminalis* female and male adults were collected from the wild and part of them were directly used for RNA extraction, of which some were bred to lay eggs in the lab, which were bred to larva and pupa for RNA extraction.

In order to explore the genetic basis of different luminous behavior of *L. yunnana* and *A. terminalis* at gene expression level, we sequenced their transcriptome of luminous organs of female and male adults. For *L. yunnana* female and male adults, luminous organs of 3 individuals were dissected as a biological sample for RNA extraction. For *A. terminalis* female and male adults, luminous organs of 10 individuals were dissected as a biological sample for RNA extraction. Three replicates were carried out for all luminous organ samples.

Total RNA was extracted using the guanidinium thiocyanate-phenol-chloroform extraction method (Trizol, Invitrogen) according to the manufacturer's protocol. RNA sequencing libraries were generated using Illumina mRNA-Seq Prep Kit. Briefly,

oligo (dT) magnetic beads were used to purify polyA containing mRNA molecules which were further fragmented and randomly primed during first strand synthesis by reverse transcription. This procedure was followed by second-strand synthesis with DNA polymerase I to create double-stranded cDNA fragments. The double stranded cDNA was subjected to end repair by Klenow and T4 DNA polymerases and A-tailed by Klenow lacking exonuclease activity. Ligation to Illumina Paired-End Sequencing adapters, size selection by gel electrophoresis and then PCR amplification completed library preparation. The 350 bp insert size paired-end libraries were sequenced using Illumina HiSeq4000 sequencers with read length of PE150.

After removing adapter sequences, about 6 Gb raw reads were generated for each sample (Table S33). Raw reads were filtered using NGSQCToolkit_v2.3.3 [50] to get high-quality clean reads for further analysis.

S6.1.2 *De novo* Transcriptome assembly

After filtering adapter and low-quality reads, 19.15 Gb high-quality clean reads of *L. yunnana* (three whole body samples: larva, male adult, and female adult) and 39.40 Gb clean reads of *A. terminalis* (five whole body samples: larva, male pupa, female pupa, male adult, female adult) (Table S33) were used to assemble *de novo* transcriptome using Bridger [51] with the default setting (*k-me*r size of 25). The final assembled transcriptomes were gained by removing redundant transcripts using CD-HIT software [52].

The *de novo* transcriptomic assembly of *L. yunnana* contained 154,017,925 base pairs (bp) with an N50 of 1975 bp and a total of 170,046 transcripts, while that of *A. terminalis* contained 152,264,687 bp with an N50 of 2221 bp and a total of 163,399 transcripts (Table S34). To estimate the quality of *de novo* assembled transcriptome, we evaluated the completeness of transcripts using BUSCO [12] with Insecta_odb9 Dataset. Our result indicated the fine completeness for both species (*L. yunnana*: 96.2%; *A. terminalis:* 95.1%) (Table S35). These transcripts were used to conduct genome annotation.

S6.1.3 Correlation among samples and calculation of FPKM value

To check correlation among samples, the clean reads from each biological sample including whole body and luminous organs of *L. yunnana* and *A. terminalis* were mapped to their corresponding *de novo* assembled genome using Tophat [53]. The number of reads aligned to annotated gene models was determined with HTSeq-count

[54]. The correlation of global expression (reads count) among samples was analyzed using cor function with spearman method from R program (https://www.R-project.org). Our data showed that the correlation coefficient among all samples of both species ranges from 0.59 to 1 (Figure S18). Among them, those among luminous organs samples within same sexes of interspecies was larger than 0.70 (Figure S18), which indicated a high correlation of expression among luminous organ samples in inter-species (Figure S18).

To calculate the gene expression level based on FPKM values, the clean reads from each biological sample and luminous organs of *L. yunnana* and *A. terminalis* were first mapped to their corresponding *de novo* assembled genome using Tophat [53], and then the program Cufflinks, Cuffmerge, Cuffnorm of Cufflinks software package [24] were used to calculate FPKM values in each sample. The genes were remained with the total FPKM≥0 from all samples as expressed genes (EGs).

S6.1.4 Analysis of high expression genes in luminous organs

The high expression genes (HEGs) were determined by choosing the first 5% EGs ranked from high to low based on the expression in luminous organs. The HEGs were performed GO and KEGG enrichment analyses of functional genes using Omicshare platform (http://www.omicshare.com/tools/). The enriched GO terms and KEGG pathway were considered significant at an adjusted P-value< 0.05.

The number of HEGs was 758 in *L. yunnana* and 769 in *A. terminalis* (Figure S19). Among HEG orthologs of two species, 262 are high expressed in both female and male for both species (Figure S19a). Most of the 262 HEGs are related to ATP metabolism, energy metabolism, transport, cysteine and methionine metabolism, etc (Figure S20; Data S11).

S6.1.5 Analysis of differentially expressed genes between luminous organs

Differentially expressed genes (DEGs) between luminous organs were analyzed using the edgeR program [55]. We first calculated the log2 fold-change (logFC) values (a minimum read count percentage) and their corresponding p-values with false discovery rate-based correction for orthologous genes between species. The genes with the absolute value of logFC ≥ 4 and false discovery rate (FDR) ≤ 0.01 were identified as DEGs of interspecific luminous organs due to longer species divergence time (Figure S21). The DEGs were performed GO and KEGG enrichment analyses of functional genes using Omicshare platform (http://www.omicshare.com/tools/). The

enriched GO terms and KEGG pathway were considered significant at an adjusted P-value< 0.05.

Between females of *L. yunnana* and *A. terminalis*, there are 1244 DEGs, with 658 up-regulated in *A. terminalis* and 586 up-regulated in *L. yunnana* (Figure S21; Table S36). Between males of *L. yunnana* and *A. terminalis*, there are 1187 DEGs, with 612 up-regulated in *A. terminalis* and 575 up-regulated in *L. yunnana* (Figure S21). Combined male and female luminous organs, there are 801 DEGs between *L. yunnana* and *A. terminalis*, with 388 up-regulated in *L. yunnana* and 413 up-regulated in *A. terminalis* (Table S36). GO and KEGG enrichments for 388 up-regulated genes in *L. yunnana* were shown as Figure S22 and Figure S23. GO and KEGG enrichments for 413 up-regulated genes in *A. terminalis* were shown as Figure S22 and Figure S23.

S6.1.6 Integrated analysis of high expression genes and differentially expressed genes in luminous organs

Combined with HEGs and DEGs between *L. yunnana* and *A. terminalis*, 388 DEGs upregulated in *L. yunnana*, of that two genes were HEGs in luminous organs of both *L. yunnana* and *A. terminalis*. While 413 DEGs upregulated in *A. terminalis,* of that 6 genes were HEGs in luminous organs of both *L. yunnana* and *A. terminalis* (Table S37).

S6.2 Proteome sequence and analysis

In order to explore the genetic basis of different luminous behavior of *L. yunnana* and *A. terminalis* at protein level, we also sequenced the proteome of luminous organs of female and male adults of the two species.

S6.2.1 Protein extraction, digestion and desalting

For *L. yunnana* female and male adults, luminous organs of 6 individuals were dissected as a biological sample for protein extraction. For *A. terminalis* female adults, luminous organs of 70 individuals were dissected as a biological sample for protein extraction; for *A. terminalis* male adults, luminous organs of 40 individuals were dissected as a biological sample for protein extraction. All experiments are in triplicate (Table S38). Total protein from each tissue samples was prepared as follows. Each biological sample was individually ground to powder in liquid nitrogen and incubated in Lysis buffer (7 M Urea, 2 M Thiourea, 4% SDS, 40 mM Tris-HCl, pH 8.5) containing 1 mM PMSF and 2 mM EDTA (final concentration) for 5 min, then

10 mM DTT (final concentration) were added to the sample. The suspension was sonicated for 15 min and then centrifuged at 4 °C, 13,000g for 20 min. The supernatant was mixed with 4 volumes of precooled acetone at -20 °C overnight. After centrifugation, the protein pellets were air-dried and resuspended in 8 M urea/100 mM TEAB (pH 8.0). Protein samples were reduced with 10 mM DTT at 56 °C for 30 min, alkylated with 50 mM iodoacetamide (IAM) for 30 min in the dark. Protein concentration was measured using Bradford method. 100 μg of proteins from each sample were used for tryptic digestion. Trypsin was added at an enzyme-protein ratio of 1:50 (w/w), and the digest reaction was performed at 37 °C for 12-16 hours. After digestion, peptides were desalted using C18 columns and the desalted peptides were dried with Vacuum concentration meter.

S6.2.2 LC-ESI-MS/MS analysis based on Triple TOF 5600 plus

The peptide samples were dissolved in 2% acetonitrile/0.1% formic acid and analyzed using TripleTOF 5600+ mass spectrometer coupled with the Eksigent nanoLC System (SCIEX, USA). Peptide was loaded onto a C18 trap column (5 μm, 100 μm×20 mm), and eluted at 300 nL/min onto a C18 analytical column (3 μm, 75 μm×150 mm) over a 120 min gradient. The two mobile phases were buffer A (2% acetonitrile/0.1% formic acid/98% H2O) and buffer B (98% acetonitrile/0.1% formic acid/2% H2O). For IDA (information dependent acquisition), survey scans were acquired in 250 ms and 40 product ion scans were collected in 50 ms/per scan. MS1 spectra were collected in the range 350–1500 m/z, and MS2 spectra were collected in the range of 100–1500 m/z. Precursor ions were excluded from reselection for 15 s.

S6.2.3 Data analysis

Protein identification and quantification were performed using ProteinPilot 4.5 software (July 2012; AB Sciex). MS/MS spectra were searched against the protein sets of *L. yunnana* (19,443 items) and *A. terminalis* (21,024 items) predicted based on our assembled genomes. Search parameters were set as follows: the instrument was TripleTOF 5600 plus, cysteine modified with iodoacetamide; biological modifications were selected as ID focus, trypsin digestion. Detected protein threshold [Unused ProtScore (Conf)]: 0.05 (10.0%); FDR Analysis tab checked. All identified proteins had an Unused Protscore of ≥1.3 (which corresponds to proteins identified with ≥95% confidence), as calculated by the software and a global false discovery rate (FDR) of ≤1% determined at the protein level by the PSPEP algorithm.

Skyline, a freely available open source software application (http:// proteome.gs.washington.edu/software/skyline), was used for label-free quantification. Skyline provides a label-free quantitation tool called Skyline MS1 filtering that efficiently extracts and processes ion intensity chromatograms from MS1 scans of peptide precursors across multiple experiments. The ProteinPilot searched file (*.group) and the native MS instrument output files (*.wiff,*.wiff.scan) were imported directly into Skyline. Detailed procedures were according to the paper [56]. For GO and KEGG pathway enrichment analysis, a P value less than 0.05 was used as the threshold [57].

S6.2.4 High abundance proteins and different abundance proteins

Totally, we identified 1933 protein groups and 18257 peptides for *L. yunnana*, as well as 2177 protein groups and 19825 peptides for *A. terminalis* (Table S39). Among them, the number of unique peptides is 17766 for *L. yunnana* and 19399 for *A. terminalis*. The characteristics of these identified peptides and protein groups were shown as Figure S24. Filtering zero abundance proteins at both females and males, we acquired 1,279 and 1,516 genes in *L. yunnana* and *A. terminalis*, respectively (Data S12-13), which were enriched mainly in cysteine and methionine metabolism and fatty acid metabolism in *L. yunnana*, and peroxisome, fatty acid degradation and elongation, isoquinoline alkaloid biosynthesis in *A. terminalis* (Data S14). In addition, we chose the first 5% proteins ranked from high to low based on the abundance in luminous organs as high abundance proteins (HAPs). 95 HAPs and 108 HAPs were defined in *L. yunnana* and *A. terminalis*, respectively (Figure S19b). Also, 33 HAPs are highly expressed in both female and male for both species (Figure S19b; Data S15), which are mainly enriched in ATP metabolism, catalytic activity, transport, acetyl-CoA metabolic process and bioluminescence (Table S40).

To ensure comparison reliably in interspecies, we conducted the correlation evaluation of quantitative results using Pearson algorithm (Figures S25-S26), showing that a good correlation for interspecies. The proteins were defined as different abundance proteins (DAPs) with a fold-change (FC) ≥ 2 or ≤ 0.5 and a P value ≤ 0.05 (*t*-test of all comparison groups). We quantified 1279 proteins in *L. yunnana* and 1516 proteins in *A. terminalis* expressed at both females and males (Table S41). Also, we identified 1151 orthologous protein groups of quantified proteins in *L. yunnana* and *A. terminalis* (Data S16). We quantified 981 orthologous protein groups in females and 935 orthologous protein groups in males between *L. yunnana* and *A. terminalis* (Table S41). There are 804 orthologs identified at female and male luminous organs of both

species (Table S41), which are mainly related to cysteine and methionine metabolism, fatty acid metabolism, sulfur metabolism and tyrosine metabolism (Table S42).

In female luminous organs, there were 210 DAPs, with 107 up-regulated in *A. terminalis* and 103 up-regulated in *L. yunnana* (Figure S27). These DAPs were mainly enriched in acyl-CoA biosynthetic and metabolic process, thioester metabolic process, catalytic activity, isomerase activity, cysteine and methionine metabolism, amino acid biosynthesis (phenylalanine, tyrosine and tryptophan), phenylalanine metabolism, sulfur metabolism and isoquinoline alkaloid biosynthesis (Data S17; Table S43).

In male luminous organs, there were 299 DAPs, with 140 up-regulated in *A. terminalis* and 159 up-regulated in *L. yunnana* (Figure S27). The male DAPs were mainly enriched in transporter activity, ATP biosynthetic process, acyl-CoA biosynthetic process, thioester biosynthetic process and biosynthesis of amino acids (Data S17; Table S43).

Combined male and female luminous organs, there are common 111 DAPs between *L. yunnana* and *A. terminalis*, with 46 up-regulated in *L. yunnana* and 61 up-regulated in *A. terminalis* (Figure S28). GO enrichment for 46 up-regulated genes in *L. yunnana* suggested that they are mainly related to cytochrome-c oxidase, oxidoreductase and etc (Figure S28). GO enrichment for 61 up-regulated genes in *A. terminalis* suggested they contributed to sulfotransferase, lysozyme and etc (Figure S28).

S6.3 Combined analyses between protein abundance and transcriptomic level

Integrated analysis of DEGs and DAPs from interspecies comparisons was performed, showing that only 23 and 31 orthologs both belonging to DAPs and DEGs were identified in female luminous organs and male luminous organs, respectively (Figure S29a, b). GO enrichments were showed in Table S44. Also, of that, 17 (9 with increased abundance and 8 with decreased abundance) and 23 (14 with increased abundance and 9 with decreased abundance) were regulated in the same direction as their corresponding DEGs in female luminous organs and male luminous organs, respectively (Figure S29b). To analyze the consistency between transcriptomic and proteomic changes in female luminous organs and male luminous organs, the correlation analysis was conducted using log fold-changes (logFC) for DAPs and DEGs (Figure S29c, d), indicating that logFC in DAPs were significantly (P=0.0052) positively correlated with logFC in corresponding DEGs in male luminous organs

(r=0.46), while no significantly (P=0.063) positively correlation in female luminous organs (r=0.35) (Figure S29c, d).

In addition, we identified 7 genes both belonging to DAPs and DEGs in both female luminous organs and male luminous organs (Table S45). Among them, one ortholog (*LT18522*, *LY04558*: glycogen [starch] synthase) are up-regulated at both transcriptomic and proteomic level in *A. terminalis*. This ortholog both belong to HEG in both female and male luminous organs of *L. yunnana* and *A. terminalis*.

Combined analyses between HAPs and HEGs of luminous organs in *L. yunnana* and *A. terminalis*, 27 pair orthologs were found to be common high expression genes whether at transcriptomic level or proteomic level (Fig. 3c; Data S18). Among them, Luc1 genes (*LT07612*, *LY04946*) were the highest expressed in female and male luminous organs of both species at proteomic level, the next (*LT14917*, *LY00406*) is fatty acid-binding protein (FABP), and the third one (*LT10814*, *LY10071*) is heximerin (also see Note S7). GO enrichment suggested that these orthologs were related to ATP related metabolic process and transport, acetyl-CoA metabolic process, sulfate assimilation, bioluminescence, etc (Table S46).

Supplementary Note 7. The genes related to the pathway of luciferin metabolism

Bioluminescence is dependent on two principal components, i.e. luciferases and its substrate luciferin [58]. Luciferin, the emitter of light, was first isolated in crystalline form from the north American firefly *P. pyralis* [59] and shown to be D-2-(6-hydroxy -2-benzothiazolyl)-2-thiazoline-4-carboxylic acid) [60]. It appears to be conserved in structure among bioluminescent beetles, but is not found in non-luminous insects [58, 61-64], suggesting the key role of luciferin in bioluminescence origin among luminous beetles. Thus, how luciferin is biosynthesized and metabolized in luminous beetle has long been a very important topic in bioluminescence researches. Since the structure of beetle luciferin was dissected, some experiments were carried out to explore the biosynthetic pathway of luciferin [65-68]. Combined the reported experimental evidence [65-68] and transcriptomic data, Vongsangnak et al. proposed the biosynthetic and metabolism pathway of luciferin and identified its related genes [69]. Later, Fallon et al. found that sulfoluciferin could serve as a luciferin storage molecule in fireflies [70]. Here, based on our data of omics (genomics, transcriptomics and proteomics) sequenced in this study and also combining all above reported evidence, we investigated the genomic basis of luciferin origin (Table S47; Data S19-27; Figures S30-S54) and described the details as follows.

S7.1 Acyl-CoA synthetase gene family and luciferase gene

Beetle luciferase has been extensively studied and cloned from more than 40 luminous beetles, and its two paralogues (luciferase 1 and luciferase 2) were identified to possess bioluminescent activity (Data S19). They belong to acyl-CoA synthetase (ACS) superfamily [71]. Based on the type of the carboxylate substrate, enzymes of this superfamily were classified into several families: luciferase (in fact also including other luciferase-like homologs, thus we use luciferase-like (LL) as substitute for reported luciferase clade), 4-coumarate:CoA ligases (4CL), acyl-CoA synthetase short-chain family (ACSS), acyl-CoA synthetase medium-chain family (ACSM), acyl-CoA synthetase bubblegum family (ACSBG), very long-chain acyl-CoA synthetase (ACSVL) (Members of this family were also investigated as fatty acid transport proteins (FATP), acyl-CoA synthetase long-chain family (ACSL) and other families (acyl-CoA synthetase family member 2 (ACSF2); acyl-CoA synthetase family member 3 (ACSF3); aminoadipate-semialdehyde dehydrogenase (AASDH=ACSF4) [72, 73]. In among them, luciferase utilize luciferin as the substrate. 4CL catalyzes the activation of various cinnamic acid derivatives (cinnamate, coumarate, caffeate, sinapate, ferulate etc.) [74]. In order to study the origin and evolution of luciferase gene, we thoroughly investigated all ACS genes in 11 beetle species (Elateroidea: six luminous beetles (five fireflies and one luminous click beetle); non-Elateroidea: five non-luminous beetles) and one fruit fly (Data S20).

S7.1.1 Identification of luciferase and ACS superfamily

A hmm profile was built based on 52 ACS genes from *D. melanogaster* (Dme) (25) (https://flybase.org/reports/FBgg0000835) and *H. sapiens* (human) (27) (https://www.genenames.org/data/genegroup/#!/group/40) using HMMER [75], and then searched by the genomes of queried species (six luminous beetles and six non-luminous species) with hmmsearch (E-value = 1e-5 for full sequence) to identify all candidate ACS genes in target species. Due to the large number of ACS genes, FastTree 2 with maximum likelihood (ML) method [34] was selected to construct the phylogenetic tree of the candidate ACS genes with *Arabidopsis thaliana* 4-coumarate:CoA ligases (4CL) (Ath4CL1) as an outgroup. Our phylogenetic data indicate beetle ACS genes were classified into eight families, i.e. LL, 4CL, ACSS, ACSM, ACSBG, FATP (ACSVL), ACSL and other families (ACSF2; ACSF3; AASDH (ACSF4)) as reported [72] (Data S20-21 and Figure S30). Our phylogenetic tree (Figure S30) shows that 4CL form two clades, one of which is sister to LL clade with both locating at the tree terminal and another of which is at the base of all other ACS; all ACS except LL and 4CL form one clade, which locate at the base of

LL-4CL clade. *CG6178* (DmePACS), which can be converted to have luciferase activity by mutagenesis of a key substitution [76], locates in the LL clade (Figure S30).

To further scrutinize the origin of luciferase, we combined LL clade and 4CL clades in Figure S30 with the cloned beetle luciferase genes (including luciferase 1 (Luc1) and luciferase 2 (Luc2)) and other cloned luciferase paralogues (Data S19) to further construct the phylogenetic tree (Ath4CL1 as an outgroup) using FastTree 2 with ML method [34]. Our result showed that except for *Zophobas morio* luciferase homology (ZopLL), which was verified to possess weak luciferase activity [77], all previously cloned beetle luciferase genes and paralogues were clustered within LL clade (Figure S31). Our data of the phylogenetic analysis and BLASTP against previously cloned beetle luciferase genes identified the luciferase genes in *L. yunnana* and *A. terminalis*, and also confirmed those in published genomes of luminous beetles. Like *A. lateralis* and *P. pyralis* [14], two luciferase genes (one Luc1 and one Luc2) were identified in both *L. yunnana* and *A. terminalis*; two Luc1 (possibly caused by the FALCON assembly) and one Luc2 in *P. pectoralis* were identified; only one Luc was identified in *I. luminosus*. Based on the prediction of PTS (the C-terminal peroxisomal targeting signal 1 (PTS1), or the N-terminal peroxisomal targeting signal 2 (PTS2)), all identified luciferase genes had PTS1 but without PTS2. The remaining LL genes were classified into peroxisomal acyl-COA synthetases (PACS) with PTS and acyl-COA synthetases (ACS) without PTS. In total, 19, 12, 13, 20, 12 and 19 PACS/ACSs were identified in *L. yunnana*, *A. terminalis*, *A. lateralis*, *P. pyralis*, *P. pectoralis,* and *I. luminosus*, respectively (Data S21; Figures S31-S33). Thus, we further analyzed the evolution of LL.

S7.1.2 Phylogeny and evolution of luciferase and other luciferase-like proteins

We further constructed the phylogenetic tree of LL using the method described as follows. Firstly, the protein sequences of all LL were aligned using MAFFT [78] with BLOSUM62 matrix and L-INS-i iterative refinement method. Secondly, the poorly aligned regions were trimmed using trimAl (gt = 0.5). Finally, RAxML [33] based on the maximum likelihood (ML) method with settings "-f a -x 12345 -N 100 -p 12345 -m PROTGAMMAWAG" was used to construct the LL phylogenetic tree with DmeACS (4CL) (*CG9009*) as outgroup. The tree was visualized and modified in iTOL [41].

To analyze the origination of luciferase genes, we estimated the divergent time of LL genes in each of six luminous beetles (Figure S32) using BEAST [79], which was calibrated with the divergence time (~330 mya) of beetle and fruit fly (*D.*

melanoganster: Dme) as substitute for that of beetle LL and DmeACS using BEAUti. The tree files were visualized using FigTree [34]. In addition, we also estimated the divergence time of all LL genes in all investigated species using similar method as above described (Figure S33). To eliminate the possibility of pseudogenes, we also calculated roughly *Ka*, *Ks*, and *Ka*/*Ks* for LL in each of six luminous species using the Codeml program of PAML [35] with the free ratio model for each branch. The *Ka*/*Ks* values of most LL genes (except for 2~3 ACS/PACS among Lyu, Ppy and Ppe) were less than 0.5, suggesting that most LL might be functional genes (Figure S32).

S7.1.3 Gene pattern, structure and their function domain of LL genes

In order to explore gene pattern, we further checked the location of these LL (ACS/PACS) genes in the genomes of six luminous beetles. Our data showed that most of LL genes in luminous beetles are arranged in tandem duplication in one or more contigs (or scaffolds or chromosomes) (Fig. 4b; Data S22). Among them, the luciferase 1 (Luc1) in all luminous beetles locate in a long tandem duplication and neighbors to a PACS at two sides or at one side (Fig. 4b) and has MGST and PRNT1 at two ends of tandem duplication (Fig. 4b). Luciferase 2 (Luc2) are a separated copy in fireflies (Data S22; Figure S32).

We counted the number of exon and intron for LL genes in six luminous species using in-house script, showing that most of LL, including luciferase gene, have 7 exons and 6 introns, while only a few have less than 7 exons or more than 8 exons (Data S23). The visual structure of luciferase genes (Luc1 and Luc2) in six luminous species was showed using Gene Structure Display Server 2.0 (http://gsds.cbi.pku.edu.cn) [80], in which luciferase genes in Lampyridae have short introns, but that of Elateridae has long introns (Figure S34).

We compared amino acid sequences of luciferases with those of the remaining LL in the phylogenetic context of LL gene family. Our data show that all protein with bioluminescent activity possess the patterns of "TSA" in Lampyridae and in Elateridae or CCA/CSA in Phengodidae and CCA in Rhagophthalmidae at the location of 346-348 amino acid of *P. pyralis* luciferase protein (Fig. 4c; Data S24).

S7.1.4 Expression of ACS superfamily

We checked the expression of all ACS genes in eight families of ACS superfamily identified in *L. yunnana* (Lyu) and *A. terminalis* (Ate) at the transcriptomic and proteomic levels. Our data show that Luc1 is the highest expressed gene at both

transcriptomic and translational level (Figure S35), demonstrating its leading role in luminous organs. Three LL (LyuACS8 (*LY08445*), LyuACS9 (*LY08446*), Lyu ACS1 (*LY02722*)) for *L. yunnana* and four LL (AtePACS1(*LT07604*), AteACS1(*LT07605*), AtePACS3(*LT07611*), AtePACS5(*LT07414*)) (LyuACS8 and AtePACS1 at the same clade, LyuACS9 and AteACS1 at the same clade)) for *A. terminalis* also show proteomic expression, suggesting a role played in luminous organs (in peroxisome or outside). Among eight 4CL for *L. yunnana* and nine 4CL for *A. terminalis* identified at proteomic level, some locate at the basal 4CL of whole ACS tree (*LY02022*, *LY02036*, *LY02035*, *LY02032*, *LY02033*, all no PTS; *LT08774* (PTS1)), suggesting they may play a role in peroxisome of luminous organs (Data S21; Figure S35). Other locate at the 4CL sister to LL and all have no PTS (*LY15957*, *LY17881*, *LY17979*; *LT12950*, *LT01985*, *LT10381*, *LT10382*, *LT10385*, *LT04875*, *LT04877*), suggesting they may function outside peroxisome of luminous organs (Data S21; Figure S35).

S7.2 The genes in cysteine metabolism pathway

Cysteine is thought as one of the precursors for luciferin biosynthesis [81, 82]. The mixture of cysteine and benzoquinone produces firefly L-luciferin even without any enzymes [68]. So, we investigated the genes in the cysteine metabolism pathway (Figure S36). Methionine is the initiating amino acid in the synthesis of virtually all eukaryotic proteins and its metabolism provides many important metabolites for a number of other pathways and biological processes [83]. For an example, the primary methyl donor S-adenosylmethionine (SAM) can be generated from methionine through the catalyzation of SAM synthetase (SAM-S), and converted to S-adenosylhomocysteine (SAH) via methyltransferases (MMT) by donating a methyl group to a receptor, such as DNA, RNA, histones, other proteins and smaller metabolites [84]. SAH can be hydrolyzed to homocysteine and adenosine by adenosylhomocysteinase (AHCY) [84]. Homocysteine can be converted to cystathionine via cystathionine-β-synthase (CBS), or remethylated into methionine through methionine synthase (MS) [84]. Cystathionine can be broken down into L-cysteine, a-ketobutyrate, and ammonia by cystathionine gamma-lyase (CGL) [85], which is a member of the Cys/Met metabolism PLP-dependent enzyme family and also a member of the broader aspartate aminotransferase family [85]. L-cysteine can be degraded to be cysteine sulfinic acid (CSA) by the oxidation of cysteine dioxygenase (CDO) and CSA can be decarboxylated into hypotaurine by cysteine sulfinate decarboxylase (CSAD) [86].

We identified candidate genes encoding above mentioned enzymes (SAM-S, MMT, Ahcy, MS, CBS, CGL, CDO, CSAD) in luminous beetles and non-luminous beetles

(Data S25) using the following methods. For SAM-S identification, SAM-S genes from *D. melanogaster* (DmeSAM-S: *CG2674*) and *B. mori* (BmoSAM-S: NP_001040509) were used to BLASTP the genomes of beetles to obtain SAM-S candidate genes after filtering those with less than 70% identity and 50% coverage. For MMT identification, four methyltransferase genes from *D. melanogaster* (*CG10903*; *CG9666*; Mt2: *CG10692*; Set1: *CG40351*) were used to BLASTP the genomes of beetles to obtain MMT candidate genes after filtering those with less than 40% identity and 50% coverage. For Ahcy identification, one gene from *D. melanogaster* (DmeAhcy: *CG11654*) was used to BLASTP the genomes of beetles to obtain the candidate genes after filtering those with less than 50% identity and 60% coverage. For MS identification, one gene from *D. melanogaster* (*CG10623*) was used to BLASTP the genomes of beetles to obtain single-copy MS genes. For CBS identification, one gene from *D. melanogaster* (DmeCBS: *CG1753*) was used to BLAST the genomes of beetles to obtain the CBS candidate genes after filtering those with less than 40% identity and 50% coverage. For CGL identification, one gene (NP_001040113) from *B. mori* [87] was used to BLASTP (E-value = 1e-5) against the beetles genomes, and those genes with more than 45% identity and 75% coverage were regarded as candidate genes. For CDO identification, one gene from *D. melanogaster* (*CG5493*) was used to BLASTP against the beetle genomes. For CSAD identification, two genes from *B. mori* (NP_001296491) and *Halyomorpha halys* (XP_014278487) were used to BLASTP against beetle genomes, and those genes with more than 50% identity and 50% coverage were regarded as candidate genes.

We also analyzed the expression of these candidate genes at transcriptomic and proteomic levels in *L. yunnana* and *A. terminalis*. Our results indicated that the SAM-S, Ahcy, CGL, CDO had higher expression at both transcriptomic and proteomic levels in luminous organs, especially for CGL that the expression in female luminous organs was higher than male luminous organs in both two species, while MS and CBS had higher expression at both transcriptomic and proteomic levels in luminous organs of *A. terminalis* (Figure S37).

S7.3 The genes in tyrosine metabolism pathway

Another precursor of luciferin biosynthesis is thought to be 1,4-benzoquinone (*p*-benzoquinone), which is thought to be from 1,4-hydroquinone under the catalyzation of polyphenol oxidase [66, 68, 88]. 1,4-hydroquinone shows similar structures to homogentisic acid (homogentisate), intermediate of tyrosine degradation, while *p*-benzoquinone is structurally similar to benzoquinone acetic acid, which can be produced from homogentisic acid under the catalyzation of phenoloxidase [89-91].

Besides, synthesis of *p*-benzoquinone involves the utilization of the aromatic ring of tyrosine or phenylalanine in beetles [92]. Thus, aiming to explore the possible contribution of tyrosine to luciferin biosynthesis, we thoroughly investigated the tyrosine metabolism pathway, including the classical degradation pathway and the cuticular tanning pathway (Figure S38).

S7.3.1 The classical degradation pathway

The phenylalanine/tyrosine degradation pathway (Figure S38) is frequently described as a catabolic pathway that funnels aromatic amino acids into citric acid cycle intermediates [93].Tyrosine is a semi-essential amino acid that can be obtained from phenylalanine catalytic by phenylalanine hydroxylase (PAH) or the digestion of food [94]. Tyrosine aminotransferase (TAT) catabolizes tyrosine to 4-hydroxyphenylpyruvate (*p* -hydroxyphenylacetic acid), which in all aerobic forms of life can be catalyzed into to homogentisate by 4-Hydroxyphenylpyruvate dioxygenase (HPPD), an Fe(II)-dependent, non-heme oxygenase [95]. Homogentisate 1,2-dioxygenase (HD) catalyzes the conversion of homogentisate to 4-maleylacetoacetate, which can be converted to 4-fumarylacetoacetate under catalyzation of maleylacetoacetate isomerase (MAAI), also known as glutathione transferase zeta (GSTZ1) [96]. Fumarylacetoacetase (FAH) converts 4-fumarylacetoacetate to acetoacetate and fumarate, which is available for the citric acid cycle.

Tyrosine is also catabolized into octopamine by aromatic L-amino acid decarboxylase (AADC) and tyramine beta-monooxygenase (TβM) [97]. Octopamine is synthesized in the insect nervous system through the hydroxylation of tyramine by the enzyme tyramine β-monooxygenase (TβM), which is similar to dopamine β-monooxygenase (DβM), the mammalian enzyme responsible for the hydroxylation of dopamine to norepinephrine. In vitro, TβM also hydroxylates tyramine to octopamine [98-100].

We identified candidate genes encoding above mentioned enzymes (PAH, TAT, HPPD, HD, MAAI, FAH, AADC, TβM) in luminous beetles and non-luminous beetles. PAH, HD, FAH, and TβM were identified by quickly scanning the functional annotation in gene families clustered in S5.1 (Gene family clusters), and at least one gene in each species were obtained (Data S25). TAT, HPPD, MAAI and AADC genes were identified as the followings: (i) firstly TAT protein sequences of *D. melanogaster* and *Homo sapiens* (human), HPPD sequences of *D. melanogaster*, *B. mori*, *T. castaneum*, *A. planipennis* and *D. ponderosae*, four MAAI genes of *D. melanogaster*, *T. castaneum* and *A. planipennis*, eight AADC genes from *D. simulans*, *D. melanogaster*, *T. castaneum*, and *A. glabripennis*, *Harmonia axyridis* and *Tenebrio*

molitor were download from UniProt database (https://www.uniprot.org/) and used to BLASTP (E-value = 1e-5) against the genome of species (*L. yunnana*, *A. terminalis*, *A. lateralis*, *P. pyralis*, *P. pectoralis*, *I. luminosus*, *A. planipennis*, *A. glabripennis*, *D. ponderosae*, *O. taurus* and *T. castaneum* and *B. mori*) to obtain correspondent candidate genes; (ii) domains corresponding to these genes (TAT: PF00155 (aminotransferase class I and II); HPPD: PF00903 and PF13669 (Glyoxalase/Bleomycin resistance protein/Dioxygenase superfamily) and PF13468 (Glyoxalase-like domain); MAAI: PF14497, PF13417, PF13409 and PF02798 of Glutathione S-transferase I; ADDC: PF00282 (pyridoxal-dependent decarboxylase conserved domain)) were scanned using HMMER [75]; (iii) redundant candidates were removed based on identify and coverage (MAAI: filtering those with less than 50% identity and 90% coverage; AADC: filtering those with less than 40% identity and 40% coverage and with the length less than 400 bp; TAT: single copy; HPPD: no filtering) (Data S25).

S7.3.2 The cuticular tanning pathway

In beetles, the cuticular tanning pathway begins with tyrosine and is responsible for production of a variety of melanin-like and other types of pigments [101]. Tanning metabolism involves quinones and quinone methides, which also act as protein cross-linking agents for cuticle sclerotization [101]. Cuticle tanning (pigmentation and sclerotization) is a complex process, which includes hydroxylation of tyrosine to L-3,4-dihydroxyphenylalanine (L-DOPA) and decarboxylation of L-DOPA to dopamine [101] (Figure S38), the precursor to other catecholamine signaling molecules and sclerotization agents [102]. Catecholamines are monoamine neurotransmitters, including epinephrine (adrenaline), norepinephrine (noradrenaline), dopamine, and catecholamine derivatives, which insects rely heavily on for cuticle hardening and innate immune responses.

We investigated the candidate genes involving cuticular tanning pathways (Figure S38), including tyrosine hydroxylase (TH), aromatic L-amino acid decarboxylase (AADC) (see Note S7), phenoloxidase (PO) (see Note S7), dopachrome conversion enzyme (DCE), arylalkylamine N-acetyltransferase (aaNAT), N-beta-alanyldopamine (NBAD) synthase (*ebony*) and catechol-O-methyltransferase (COMT). TH and COMT were identified by quickly scanning the functional annotation in gene families clustered in S5.1 (Gene family clusters), and in total, 13 TH and 14 COMT genes were obtained, respectively (Data S25). For DCE identification, the *yellow-f* (*CG18550*) and *yellow-f2* (*CG8063*) genes of *D. melanogaster* were used to BLASTP (E-value = 1e-5) the genomes of 13 beetle species (Lyu, Ate, Ala, Ppy, Ppe, Ilu, Apl,

Agl, Tca, Dpo, Ota) and silkworm (Bmo) with more than 35% identity and 60% coverage. For *ebony* identification, the *ebony* genes from *D. melanogaster* and *B. mori* were used to BLASTP (E-value = 1e-5) against the genomes of beetle species (Lyu, Ate, Ala, Ppy, Ppe, Ilu, Apl, Agl, Tca, Dpo and Ota) with more than 40% identity and 60% coverage. To further determine DCEs and *ebony*, the conserved domains (PF03022 (major royal jelly protein) for DCEs; PF00501 (AMP-binding enzyme) for *ebony*)) were scanned using HMMER 3.2 [75] to filter those with lower 80% coverage of domain (Data S25). PSI-BLAST (Position-Specific Iterated BLAST) [103], which is fit to detect distant relationships between proteins, and HMMER 3.2 [75], which is designed to detect remote homologs as sensitively as possible relying on the strength of its underlying probability models, were used to identify aaNAT genes due to its low identity among diverse species according to the followings: (i) eight aaNAT genes from *D. melanogaster* and two aaNAT genes from *B. mori* [104] were used as database to query against the genomes of 13 beetle species (Lyu, Ate, Ala, Ppy, Ppe, Ilu, Apl, Ota, Agl, Dpo, and Tca) by PSI-BLAST (E-value = 1e-5); (ii) these known genes were also used to build a hmm profile with HMMER, which was searched by queried species with hmmsearch (E value = 1e-5 for full sequence and domain length) to identify candidate aaNAT genes; (iii) all possible candidate aaNAT genes were obtained by combining PSI-BLAST with HMMER results (Data S25).

S7.3.3 The expression analysis of genes identified in tyrosine metabolism pathway

We also analyzed the expression of all candidate genes in tyrosine metabolism identified in *L. yunnana* and *A. terminalis* at transcriptomic and proteomic levels. Our results showed that most genes in degradation pathway (PAH, HPPD, HD and FAH) had higher expression at both transcriptomic and proteomic levels in luminous organs, while most genes in cuticular tanning pathway (TH, AADC and TβM) had lower or no expression at transcriptomic and proteomic levels in luminous organs (Figure S39), indicating that tyrosine is mainly degraded via such intermediate as homogentisate.

S7.4 β-glucosidase genes

Firefly luciferin biosynthesis is thought to be produced from 1,4-hydroquinone [82], which is proposed to be stored as arbutin and was immediately oxidized to 1,4-benzoquinone (*p*-benzoquinone) after its releasing from arbutin [69, 82]. The production of 1,4-hydroquinone from arbutin is hydrolyzed by arbutin hydrolysis enzymes, i.e. glucosidases (BGL) [69, 82, 105]. Thus, we analyzed BGL genes in the genomes of luminous beetles and non-luminous beetles.

We used the amino acids of eight cloned β-glucosidase genes (EC: 3.2.1.21) (BGLs) from eight insects (cockcroach *Periplaneta americana*, AIA09348.1; termite *Odontotermes formosanus*, ADD92156.1; termite *Nasutitermes takasagoensis*, BAI50022.1; termite *Anoplotermes schwarzi*, AGP76178.1; termite *Rhynchotermes bulbinasus*, AGP76180.1; termite *Macrotermes barneyi*, AFD33364.1; termite *Macrotermes carbonarius*, AGP76179.1; silkworm *Bombyx mori*, AAP13852.1) [106, 107] to BLASTP (E-value = 1e-20) against the reference genomes of 12 targeted species. After removing redundancy, we identified the genes with more than 44% identity and 60% coverage as primary candidates. Subsequently, these primary candidates were further confirmed by scanning the best hit domain, the glycosyl hydrolases family 1 (GH1) (PF00232), using the Hidden Markov Model (HMM) HMMER 3.2 [75]. In total, 190 BGLs were obtained (Data S26). Our BGL identified in *D. melanogaster* (Data S26) was consistent with the previously reported [107, 108], suggesting our methodology is reliable. Only four genes in four species (*I. luminosus*: BGL21; *A. lateralis*: BGL6; *A. terminalis*: BGL6; *T. castaneum*: BGL3) have PTS1 signal and one gene in *T. castaneum* has PTS2 signal (Figure S40).

191 BGL genes identified above in the genomes of beetles and fruit fly, together with the reported one BGL genes (c11559, c14185 without PF00232) from firefly *Luciola aquatilis* [109], were used to infer phylogenetic tree with DmeBGL1 as outgroup using similar methodology to that described in S7.1.2. In details, their amino acid sequences were aligned using MAFFT [78] with BLOSUM62 matrix and FFT-NS-i iterative refinement method, and the poorly aligned regions and partial gaps in alignment were removed with trimAl (gt=0.5) [110]. Then, the maximum likelihood (ML) tree was constructed using RAxML [33] with defaults parameters. The tree was visualized and modified by iTOL [41]. Our tree (Figure S40) shows that beetle BGLs form three clades (Clade1, Clade2 and Clade3). The Clade1 includes no luminous beetle BGLs, the Clade2 includes BGLs of all beetles, and the Clade3 includes BGL genes of *A. planipennis* and luminous beetles (all belonging the Series Elateriformia), with those of luminous beetles (including c11559) at the Clade3.

We checked the expression of BGL genes at transcriptomic and proteomic levels at the different developmental stages and adult luminous organs of *L. yunnana* and *A. terminalis* (Figure S52-S54). Among BGLs of *L. yunnana*, LyuBGL3 (*LY09398*) and LyuBGL7 (*LY09402*), clustered with c11599 and located at Clade3, show high transcriptional expression in luminous organs. Among them, LyuBGL3 (*LY09398*) is the highest expression in the luminous organs of both female and male, and LyuBGL7 (*LY09402*) is expressed highly in female luminous organ. However, at proteomic level,

only other two genes ((LyuBGL10 (*LY13221*) and LyuBGL9 (*LY12234*)) were identified to be active (Figure S54), both of which is low expression at transcriptional level (Figure S52). Among them, LyuBGL9 (*LY12234*) locate in the Clade3, but LyuBGL10 (*LY13221*) locate at the Clade2. In *A. terminalis*, AteBGL2 (*LT06876*), located at the Clade3, was found the highest expression in luminous organs of female and male at both transcriptional and proteomic levels; AteBGL1 (*LT04765*), located at the Clade2, is highly expressed in female luminous organ at both transcriptional and proteomic levels (Figure S53-S54). From these data, we speculated that LyuBGL10 (*LY13221*), LyuBGL9 (*LY12234*), LyuBGL3 (*LY09398*), LyuBGL7 (*LY09402*), AteBGL2 (*LT06876*) and AteBGL1 (*LT04765*) may play an important role in luciferin synthesis of luminous organs. In addition, we calculated the expression of luminous organs from *A. lateralis*, *P. pyralis* and *I. luminosus* at transcriptome [14], finding that the BGL genes clustered the same two clades with *L. yunnana* and *A. terminalis* genes had high expression slightly (Data S27).

S7.5 Phenoloxidase (tyrosinase) genes and hexamerin genes

Benzoquinone is usually presented in the defensive exudates of arthropod including beetles [111-116]. Polyphenol oxidase were thought to take part in the biosynthesis of *p*-benzoquinone from 1,4-hydroquinone [113, 117, 118], and similarly in the biosynthesis of benzoquinone acetic acid from homogentisic acid [89-91, 119-121]. Insect prophenoloxidae/hexamerin protein family include phenoloxidases (tyrosinases) (PO) and hexamerin (Hex)[122, 123]. Hexamerin are thought to act as storage protein which are used as a source of amino acid an energy [124-126]. Our expression data also indicate that the gene annotated as heximerin (Note S6 and Data S18) is the third highest in female and male luminous organs of both species at proteomic level (the first: Luciferase; the second: atty acid-binding protein (FABP)). So, we investigated the phenoloxidase and hexamerin genes in the genomes of luminous beetles.

Three PO genes from *D. melanogaster* (DmePPO1, NP_476812; DmePPO2, NP_610443; DmePPO3, NP_524760) [127] and two PO genes (c14353 and c14376) from firefly *Luciola aquatilis* [109] were used references to BLASTP (E-value = 1e-20) against the beetle genomes (Lyu, Ate, Ala, Ppy, Ppe, Ilu, Apl, Ota, Agl, Dpo and Tca) with more than 45% identity and 60% coverage. For identifying hexamerins, the known heximerin protein sequences available from the UniProt database (https://www.uniprot.org/) were used as queries to BLASTP (E-value < le-5; identity > 40%; score >100) against the beetle genomes; the identified hexamerin candidates were searched for conserved hexamerin domains in the Pfam database (PF03722: Haemocyanin_N; PF00372: Haemocyanin_M; PF03723: Haemocyanin_C)

using the program HMMER (http://hmmer.janelia.org/); the notably conserved motif "TXX(R)DP" was further checked in all identified hexamerins [128]. The obtained hexamerins were classified based on their amino acid composition following the criteria described elsewhere [129].

In total, 30 PO genes and 36 Hex genes were identified in beetle genomes (Data S26). Only two PO genes in *A. glabripennis* (AglPO1 and AglPO3) and two Hex genes (Lyu, Ate) have PTS1 signal, but five genes (Lyu: LyuPO1, LyuPO2, LyuPO3; Ala: AlaPO1; Ppy: PpyPO2) have PTS2 signal in three firefly species. All beetle Hex genes belonged to the subtype of arylphorin hexamerin, and no methionine-rich or riboflavin-binding hexamerins were identified.

The amino acid sequences of 35 PO genes and 36 Hex genes including above identified and previously known plus two crustacea POs (*Penaeus monodon* (PmoPPO: AF099741) and *Pacifastacus leniusculus* (PlePPO: X83494)) were aligned using MAFFT [78] with BLOSUM62 matrix and L-INS-i iterative refinement method and the spurious sequences and poorly aligned regions were filtered using trimAl (gt = 0.5). The phylogenetice tree was constructed t using RAxML software (--auto-prot=ml -f a -x 12345 -N 100 -p 12345 -m PROTGAMMAWAG) [33] with PmoPPO and PlePPO as outgroups, and visualized and colored using iTOL [41]. Our tree (Figure S41) showed that PO genes and Hex genes of all luminous beetles form two seperated clades (i.e. PO clade and Hex clade) with PO clade at the base.

The expression analysis indicates that Hex (LyuHex: *LY10071*, AteHex: *LT10814*) are higher than PO in the female and male luminous organs of *L. yunnana* (Lyu) and *A. terminalis* (Ate) at both transcriptional and proteomic levels (Figure S52-S54). Hex is the third highest expressed gene in luminous organs ((Data S18). The combined data demonstrated that Hex play an important role, e.g. to store such amino acid as cysteine (precursor of luciferin). For PO genes, LyuPO2 (*LY16904*) and LyuPO3 (*LY12653*) show high expression at all developmental stages and luminous organs with LyuPO2 (*LY16904*) higher than LyuPO3 (*LY12653*) (Figure S52), and AtePO1 (*LT15038*) and AtePO2 (*LT16085*) show high expression (Figure S53). At proteomic level, 3 PO genes (LyuPO1 (*LY16898*), LyuPO2, LyuPO3) and 2 PO genes (AtePO2, AtePO3) were identified to be active in *L. yunnana* and *A. terminalis*, respectively (Figure S54). LyuPO2 is the highest in *L. yunnana*, and AtePO2 is the highest in *A. terminalis*. Combined transcriptomic and proteomic data, we speculate that LyuPO1, LyuPO2 and LyuPO3, and AtePO2 and AtePO1 may play a key role in luciferin biosynthesis. In addition, the expression of Hex genes from *A. lateralis* and *P. pyralis* was extremely high in luminous organs (Data S27).

S7.6 The thiolase gene family

Peroxisomes, the place of light reaction in the luminous organs of luminous beetles, are well-known as one of β-oxidation organelles. The peroxisomal β-oxidation system includes: (1) a classical peroxisome proliferator-inducible pathway capable catalyzing straight-chain acyl-CoAs by fatty acyl-CoA oxidase, L-bifunctional protein, and thiolase; and (2) a second noninducible pathway catalyzing the oxidation of 2-methyl-branched fatty acyl-CoAs by branched-chain acyl-CoA oxidase (pristanoyl-CoA oxidase/thrihydroxycoprostanoyl-CoA oxidase), D-bifunctional proteins, and sterol carrier protein (ScpX) [130]. The last step of β-oxidation is that 3-ketoacyl-CoA is broken into acyl-CoA and acetyl-CoA by the thiolase reaction mechanism [131]. Thiolases, also known as acetyl-coenzyme A acetyltransferase (ACAT), are ubiquitous enzymes that have key roles in many vital biochemical pathways including the beta oxidation pathway of fatty acid degradation (3-ketoacyl-CoA thiolase: thiolase I) and various biosynthetic pathways (acetoacetyl-CoA thiolase: thiolase II) [132]. Sterol carrier protein X (ScpX) is a fused protein of 58 kDa with thiolase activity (3-oxoacyl CoA thiolase) at its N-terminal and sterol carrier protein activity at its C-terminal, playing a central role in branched chain fatty acid β-oxidation in peroxisome [133]. The benzoquinone acetic acid can be thought as alpha-branched fatty acid and thus may be degraded into *p*-benzoquinone if an acetyl group is removed by 3-ketoacyl-CoA thiolase in β-oxidation. Thus, we investigated the thiolase gene family.

Thiolase related genes of *D. melanogaster* from FlyBase (http://flybase.org) (ScpX: *CG17320*; Thiolase: *CG4581*; yip2 (yippee interacting protein 2): *CG4600*; Hmgs (HMG Coenzyme A synthase): *CG4311*; Thiolase-like: *CG9149*; *CG10932*; *CG12170*; *CG17597*) were used a set to BLASTP against the genomes of luminous and non-luminous beetles. After discarding those with less than 40% identity and 50% coverage, we acquired 119 genes of thiolase family (Data S26). The amino acid sequences of 119 genes were aligned using MAFFT [78] with BLOSUM62 matrix and FFT-NS-i iterative refinement method and filtered for spurious sequences and poorly aligned regions using trimAl (gt = 0.5), and then were used to construct the gene tree using RAxML software (--auto-prot=ml -f a -x 12345 -N 100 -p 12345 -m PROTGAMMAWAG) [33]. The tree was visualized and colored using iTOL [41]. Our phylogenetic tree shows (Figure S42) that like those of fruit fly, beetle thiolase genes can be divided into seven clades. By predicting the C-terminal peroxisomal targeting signal 1 (PTS1) and the N-terminal targeting peroxisomal targeting signal 2 (PTS2) for thiolase related genes in 11 beetles and fruit fly, we discovered that at least one

gene for each species in ScpX clade had PTS1 (Figure S42), suggesting that peroxisome is their function place.

The expression analysis showed that the peroxisomal ScpX genes had higher expression at both transcriptomic and proteomic levels at *L. yunnana* and *A. terminalis* (Figure S52-S54), and also at transcriptome of luminous organs in other three luminous beetles [14] (Data S27).

S7.7 Alpha-methyl-acyl-CoA-racemase and acyl-CoA thioesterases genes

Alpha-methyl-acyl-CoA-racemase (AMACR) catalyzes the chiral inversion of a diverse number of 2-methyl acids (as their CoA esters), and regulates the entry of branched-chain lipids into the peroxisomal and mitochondrial β-oxidation [134, 135]. D-luciferin (light reaction substrate) is thought to be converted from L-luciferin via L-luciferyl-CoA to D-luciferyl-CoA. Just like α-branched fatty acyl-CoA, luciferyl-CoA has a proton at α-position, and AMACR from bacterial *Rhodococcus erythropolis* was verified to contribute to an efficient light emission in the combination of the three enzymes, Luc from firefly *Luciola cruciata*, fatty ACOT II from *Escherichia coli* and AMACR [136]. To explore the possible contribution of firefly AMACR and ACOT to D-luciferin biosynthesis, we investigated them in luminous beetles and non-luminous beetles.

S7.7.1 Alpha-methyl-acyl-CoA-Racemase gene

We used the AMACR (*CG9319*) gene from *D. melanogaster* to identify AMACR genes by BLASTP against the genomes of luminous and no-luminous beetles. After removing less than 50% identity, we identified one AMACR gene in each species. Also, each gene in luminous beetles had the PTS1 signal (Figure S43).

The amino acid sequences of 11 AMACR genes including identified and DmeAMACR were aligned using MAFFT [78] with BLOSUM62 matrix and L-INS-i iterative refinement method, which were used to construct the phylogenic tree that DmeAMACR was regarded as the outgroup using RAxML software (--auto-prot=ml -f a -x 12345 -N 100 -p 12345 -m PROTGAMMAWAG) [33]. The tree was visualized and colored using iTOL [41] (Figure S43).

The expression of AMACR in luminous beetles was showed in Fig. 5b-c and Figure S52-S54, indicating that AMACR gene was overexpressed at transcriptomic level in

luminous organs of six luminous beetles, also had high abundance at proteomic level in luminous organs of *L. yunnana* and *A. terminalis* (Data S27).

S7.7.2 Acyl-CoA thioesterases gene

Acyl-CoA thioesterase (ACOTs) are divided into two families, i.e. Type-I and type-II, where the type-I thioesterase, together with the acyl-transferases, show a high degree of sequence similarity and structurally belong to the α/β-hydrolase superfamily, which is one of the largest superfamilies of proteins[137]. The type-II thioesterases show a low degree of sequence similarity to each other with the HotDog domain [137]. The type-I thioesterases are only found in (some) bacteria and in the animal kingdom (not in yeast, insects or plants), while the type-II thioesterases are found in all three branches of life [137].

We collected 10 type-I thioesterases from human (ACOT1-2, ACOT4, ACOT6) [138] and mouse (ACOT1-6) [139], 19 cloned type-II thioesterases from human (ACOT7-9, ACOT11-15) [138], mouse (ACOT7-15) [139] and firefly *Luciola aquatilis* (c9513 and c13177) [109] to BLASTP (E-value = 1e-5) against twelve insect genomes (11 beetles and *D. melanogaster*). No type-I ACOTs were identified even through E-value = 1e-3, consistent with the reported that no type-I in insects [137]. Type-II ACOTs (67 ACOTs) were identified after discarding duplication (Data S26), in which only five genes in four species (*L. yunnana*, *D. ponderosae*, *O. taurus*, *T. castaneum*) have PTS1 signals (Fig. 6a). In addition, the HotDog domain superfamily (IPR029069) of these ACOTs were found by searching against InterPro database (http://www.ebi.ac.uk/interpro/). Based on the above contents, ACOTs of fireflies belong to the type-II thioesterases with a low degree of sequence similarity to each other.

Due to the very divergence among 86 ACOTs sequences (including 9 insects, mouse and human), we aligned ACOTs amino acid sequences using MAFFT with BLOSUM62 matrix and L-INS-i iterative refinement method and trimmed poorly aligned regions and gaps using trimAl (gt = 0.2) [110] to construct the ML rooted tree using FastTree 2 [34] with fatty acyl-CoA thioesterase (ACOT) II from *Escherichia coli* (E.coilACOTII) as the group. The tree was visualized using iTOL [41]. Motifs of all ACOTs of the gene tree were identified using MEME (http://meme-suite.org/index.html) (Fig. 6a). The domains of all ACOTs were analyzed using HMMER [75] to compare with E.coilACOTII confirmed the key role of converting L-luciferin to D-luciferin [136] and were visualized using iTOL [41] (Fig. 6a). Our tree (Fig. 6a) showed that most luminous beetle (LB) ACOTs form two LB-specific clades and the remaining dispersed in the middle of human and mouse. One of LB-specific clade, together with some ACOTs of luminous beetles,

non-luminous beetles and fruit fly ACOTs, is sister to human and mouse mitochondrial ACOT (HomoACTO9, MusACOT9 and ACOT10), and all of them have two 4HBT domains and eight conserved motifs (Fig. 6a). Another of LB-specific clade, together with most of the remaining ACOTs, have mainly two motifs and one 4HBT domain. In addition, AteACOT8 and AteACOT9 (no PTS), locate at the base of two above mentioned clades, with human and mouse peroxisomal ACOT (HomoACOT8 and MusACOT8) further at their base. All four ACOTs (AteACOT8, AteACOT9, HomoACOT8 and MusACOT8) have 4HBT_3 or Abhydrolase domain, instead of 4HBT domain. 4HBT and 4HBT_3 domains belong to CL0050 Hotdog superfamily.

ACOT expression at transcriptional level is shown in Figure S52-S54. LyuACOT1, LyuACOT3 and LyuACOT5 are higher expression than other ACOT genes (Figure S52), and AteACOT1 and AteACOT4 is higher expression than other ACOT genes (Figure S53). At proteomic level, LyuACOT1 is active in both female and male luminous organs, but LyuACOT5 is active in female and LyuACOT2 is active in male. For *A. terminalis*, only AteACOT1 is active in female and male (Figure S54). Combining transcriptomic and proteomic data, we speculate that LyuACOT1, LyuACOT2, LyuACOT5 and AteACOT1 can play important role in luciferin synthesis.

S7.7.3 Functional verification of some key genes in converting L-luciferin to D-luciferin in vitro synthesis experiments

Based on the above analysis, we selected three representatives (AteACOT1, AteACOT4 and AteACOT9) of AteACOTs to verify their role in stereoisomeric inversion of L-luciferin and D-luciferin. AteACOT1 had two 4HBT domains and eight motifs and showed the highest expression in luminous organs at both transcriptomic and proteomic levels (Figure S53-S54). AteACOT4 had one 4HBT domains and two motifs (Fig. 6a) and showed a higher expression at transcriptomic level (Figure S53). Although no expression in luminous organs was identified for AteACOT9, a same complete domain 4HBT_3 similar to that of fatty acyl-CoA thioesterase (ACOT) II from *Escherichia coli* (Fig. 6a) was found for it. Thus, we chose these three ACOTs (AteACOT1, AteACOT4 and AteACOT9) as candidate representatives to verify their combined role in stereoisomeric inversion of L-luciferin to D-luciferin in vitro experiments with luciferase (LUC) and alpha-methyl-acyl-CoA-racemase (AMACR).

S7.7.3.1 Recombinant plasmids construction

The synthesized DNA sequences including the firefly LUC, AMACR and ACOT (AteACOT1, AteACOT4 and AteACOT9) with optimized codons according to the preference of *E. coli* and six His tags were constructed to the pET-28a vector, which were named as pET-28a-LUC, pET-28a-AMACR, pET-28a-ACOT1, pET-28a-ACOT4 and pET-28a-ACOT9, respectively. In addition, the ACOT1, ACOT4 and ACOT9 were further subcloned into the pCold-TF vector using the restriction enzyme digestion (restriction sites: Ndel and Xhol) to get pCold-TF-ACOT1, pCold-TF-ACOT4 and pCold-TF-ACOT9 because they failed to express well in pET-28a vectors. The recombinant plasmids (pET-28a-LUC, pET-28a-AMACR, pCold-TF-ACOT1, pCold-TF-ACOT4, pCold-TF-ACOT9) were transformed into *E. coli* DH5α for amplification and extraction, which having correct sequences of target genes confirmed by PCR and sequencing were transformed into *E. coli* BL21(DE3) to express proteins, respectively.

S7.7.3.2 Protein expression and purification

50 μL *E. coli* BL21 (DE3) cell glycerol stocks containing the recombinant plasmids were inoculated into 5 mL LB medium and cultured overnight (10 to 12 hours) at 37°C and 220 rpm incubator. Then an aliquot of 500 μL bacterial fluid was further inoculated into 50 mL LB medium and cultured to logarithmic growth phase (4 to 6 hours) at 37°C and 220 rpm incubator. 15 mL of the above were further transferred into 800 mL 2 × YT medium and incubated at 37°C and 220 rpm incubator until absorption at 600 nm was approximately 0.6~0.8 (OD_{600}=0.6~0.8) measured using spectrophotometer (UNICO, USA), which were then cultured at 15°C for 30 min. Then, the expression of recombinant protein was induced with 0.5 mM isopropyl-D-thiogalactopyranoside (IPTG) at 15°C and 220 rpm incubator for 16 hours. The cells were collected by centrifugation at 5500 rpm for 15 min, and then resuspended in 50 mM Tris-HCL buffer (pH = 8.0, containing 150 mM NaCl), lysed by ATS AH-1500 cell disruptor within 800 to 1000 bar for 3 min. The bacterial lysate was centrifuged for 45 min (4°C, 8500 rpm). The protein was purified from supernatant by nickel-charged affinity resin using nicke initrilotriacetic acid (Ni^{2+}-NTA) column (Qiagen, Germany) and identified by 10% SDS-PAGE. Protein concentration was determined using Pierce BCA Protein Assay Kit after concentrating eluate with 200 mM imidazole through a concentrator. ACOTs with trigger factor (TF) protein was digested using Factor Xa protease to obtain ACOT protein. The size and purity of the proteins were showed using SDS-PAGE in Figure S44a, consistent with the estimated results (Figure S44b).

S7.7.3.3 The reaction of L-Luciferin converted into D-Luciferin in vitro

The reaction mixture (200 μL) contained 0.1 mM L-luciferin, 8 mM $MgSO_4$, 3 mM ATP-H_2, 0.5 mM COASH, 1μg per protein (LUC, ACOT1, ACOT4, ACOT9, AMACR) in 100 mM Tris-HCl. We first checked the stereoisomeric inversion of L-luciferin to D-luciferin catalyzed by LUC or LUC+ACOT1 during different

reaction time from 15 min to 150 min at pH8.0 and 30 ℃ (Figure S45). Then we checked activity of LUC or LUC+ACOT1 at different pH conditions (6.0, 7.0, 8.0) (Figure S46). Then, we compared the activity of different enzymes to catalyze luciferin stereoisomeric inversion during 45 min at 30 ℃ (Figure S47). The luciferin chirality was monitored by high-performance liquid chromatography (HPLC) system (Alliance HPLC System with 2695 Separation Module, 2475 Multi k Fluorescence Detector, Waters) using a chiral fused silica column (Chiralcel OD-RH, 4.6 × 150 mm; Daicel Chemical Industry, Tokyo, Japan). D-luciferin and L-luciferin were detected with a fluorescence detector (excitation k = 330 nm, emission k = 530 nm). Bioluminescence of the reaction mixture was measured using a Luminescencer Octa AB-2270 (ATTO, Tokyo, Japan). 80 µl substrate solution (100 mM Tris-HCl [pH=8.0], 2 mM ATP, 0.2 mM L-luciferin, 4 mM MgSO4, 125µm CoA) was prepared at 25 °C and left for 30 min. Then, 20 µl enzyme solution (10 µl LUC diluted solution (5ng/µl) and 10 µl double distilled water, or 10 µl the LUC diluted solution (5ng/µl) and 10 ul ACOT1 (5ng/µl)) were added to the substrate solution and incubated for different time (1 min, 10 min, 20 min, 30 min, 40 min, 50 min, 60 min, 90 min, 120 min). Last, luminescence activity of mixtures incubated for different times was measured during 20 second, and the integrated activity was described by relative light unit (RLU) (Figure S45e). The measurement of mixtures for each incubated time was performed with six replicates.

Our results indicated that ACOT1, together with LUC, can promote the high efficient inversion of L-luciferin into D-luciferin, while LUC alone can convert L-luciferin into D-luciferin only with low efficiency (Fig. 5k and Figure S47). ACOT4 and ACOT9 have no promoted role (Figure S47c). AMACR can promote the consumption of L-luciferin (Figure S47d), but no effect on production of D-luciferin (Figure S47c). Our results also indicated that the conversion of L-luciferyl-CoA to D-luciferyl-CoA was most likely non-enzymatic catalysis, consistent with the previous reports [136]. Thus, we concluded that the key enzymes for the conversion of L-luciferin to D-luciferin in fireflies were LUC and one of ACOT (Fig 5k), which were consistent with the proposed luciferin metabolic pathway reported [109] and in Fig. 5a.

S7.8 Sulfotransferase and luciferin sulfotransferase genes

Sulfotransferase (ST) are Phase II detoxifying enzymes that mediate the sulfate conjugation of numerous xenobiotic molecules by catalyzing the transfer of the sulfonate group from the active sulfate, 3′-phosphoadenosine 5′-phosphosulfate (PAPS), to substrate compounds containing hydroxyl or amino group (s) [140, 141]. Sulfoluciferin was observed to be abundant in fireflies as a luciferin storage, and

luciferin sulfotransferase (LST), belonging to the family sulfotransferase, catalyzes its production from firefly luciferin and sulfo-donor PAPS [70]. To explore the contribution of LST to bioluminescence origin and evolution, we thoroughly analyze this gene family in the genomes of luminous beetles and non-luminous beetles.

The sulfotransferase (ST) genes were identified using cloned three ST genes (PpyLST: ANH56803; PpyST2: ANH56804; PpyST3: ANH56805) from *P. pyralis* [70] by a BLASTP similarity search with an e-value cutoff of 1e-20 against five firefly protein databases and other insect genomes. The luciferin sulfotransferase (LST) genes were selected with more than 70% identity and 90% coverage, resulting in identifying one LST in each firefly genome, respectively. Owing to the low identity that about 35% among PpyLST, PpyST2 and PpyST3, we chose the candidate ST genes with remaining more than 30% identity and 60% coverage. The genes were further filtered that the PF00685 domain of ST and LST genes, sulfotransferase, was scanned using the HMMER 3.2 [75] and the short (<267 aa, the length of PF00685 domains was 267 aa) genes were discarded. At the end, we acquired 168 ST genes and 5 LST genes (Data S26). The identified PpyLST, PpyST2 and PpyST3 were consistent with the cloned [70]. Our identification data show that ST gene is expanded greatly among fireflies. PTS1 of LST can be found only in Lampyrinae species (Ppy: PKM; Ppe: PKI).

The protein sequences of LST and ST genes were aligned using MAFFT [78] with BLOSUM62 matrix and L-INS-i iterative refinement method and trimmed unaligned regions using trimAl (gt = 0.5) [110] to construct the unrooted maximum likelihood (ML) tree of LST and ST genes using the substitution model GTRGAMMA of rate heterogeneity of the RAxML software [33] with 100 rapid bootstrap inferences. Our tree showed that five LST genes clustered together, which, together with some of firefly ST genes, form a clade with some of other beetle ST genes, although most ST form another firefly specific clade (Figure S48).

The expression of LST and ST from *L. yunnana* and *A. terminalis* was showed in Figures S52-S54. LST showed high expression at transcriptomic and proteomic levels**.** While AteST5 also showed high expression, especially at the proteomic level that significantly higher than LST in luminous organs of *A. terminalis*.

S7.9 Sulfatase gene

Sulfatases (SULF) hydrolyze sulfate esters from different sulfated substrates such as steroids, carbohydrates, proteoglycans and glycolipids [142]. Together with sulfotransferases, sulfatases form the major catalytic machinery for the synthesis

and breakage of sulfate esters. Fallon et al. speculate that luciferin may be released from sulfoluciferin *in vivo* by uncharacterized sulfatase [70]. To explore the possible contribution of sulfatases to luciferin metabolism, we identified this gene family in beetle genomes.

To identify the SULF genes in the Coleoptera and *D. melanogaster* genomes, a systematic BLASTP (E-value =1e-5) search of these genomes was performed using SULF protein sequences available from National Center for Biotechnology Information (NCBI) (http://www.ncbi.nlm.nih.gov/) and Xiao-Li Ma's article [143] as queries. Candidate SULF sequences were further to examine to identify Sulf domains by searching the conserved protein domain database against Pfam. After removing redundancy, top hits for putative SULF were retained. Candidate genes were further verified using BLASTP versus non-redundant NCBI protein sequences (NR database). We acquired 130 SULF genes in genomes of 11 beetle genomes and fruit fly (Data S26; Table S47).

Multiple alignments of the SULF protein sequences were performed using MAFFT [78] and removed poor aligned regions and partial gaps with trimAl [110]. SULF proteins were then subjected to a phylogenetic analysis using FastTree 2 [34]. The resulting tree was displayed and edited using the iTOL [41]. Our tree showed that all SULF genes form 5 group (A, B, C, D, E) (Figure S49).

The expression of SULF genes at transcriptomic level and proteomic levels was showed in Figures S52-S54. We found one pair of orthologs (*LY08574* and *LT02334*) in group A were high expressed in luminous organs of female and male at both transcriptomic and proteomic levels, and also the orthologs (*PPYR_06806-PA* , *AQULA_010839-PA*) from *A. lateralis* and *P. pyralis* showed similar expression in luminous organs (Data S27), which suggested that they may contribute to the release of luciferin from sulfoluciferin in fireflies.

S7.10 5'-phosphosulfate synthetase gene

We found a pair of ortholog (orth_4964: *LT02179* and *LY11043*) functionally annotated as bifunctional 3'-phosphoadenosine 5'-phosphosulfate synthase in the list of high expression at both transcriptional and proteomic levels. In animal, bifunctional 3'-phosphoadenosine 5'-phosphosulfate synthase (PAPSS) is a fused protein of ATP sulfurylase and adenosine 5'-phosphosulfate kinase, and its function is to catalyze synthesis of universal sulfonate donor, 3'-phosphoadenosine 5'-phosphosulfate (PAPS) [144]. PAPS is also possibly a sulfonate donor to sulfaration

of luciferin to form sulfoluciferin [70]. Therefore, we investigate the PAPSS genes in the genomes of luminous and non-luminous beetles.

The PAPSS genes were identified using DmePAPSS (*CG8363*) of *D. melanogaster* from FlyBase (http://flybase.org/) by a BLASTP similarity search (E-value = 1e-5) against five firefly protein databases and other insects. The genes with more than 60% identity were as candidate genes. We acquired 27 PAPSS genes (Data S26). Six PAPPS genes (AtePAPPS1: *LT02179*; AtePAPSS2: *LT02494*; AlaPAPPS1: *AQULA_110585-PA*; PpyPAPSS2: *PPYR_14372-PA*; PpePAPSS2: *augustus_masked-scaffold418-processed-gene-1.465*; PpePAPSS4: *snap_masked-scaffold383-processed-gene-3.496*) have PTS1 signals.

The amino acid sequences of 27 PAPSS genes were aligned using MAFFT [78] with BLOSUM62 matrix and L-INS-i iterative refinement method. The gene tree was constructed using RAxML software (--auto-prot=ml -f a -x 12345 -N 100 -p 12345 -m PROTGAMMAWAG) [33] with DmePAPSS as an outgroup, and visualized and colored using iTOL [41]. Our tree shows a luminous beetle specific PAPSS clade exist (Figure S50). One pair orthologs (AtePAPSS1 and LyuPAPSS2) have high expression in the luminous organs of *L. yunnana* and *A. terminalis* at both transcriptomic and proteomic levels (Figures S52-S54).

S7.11 Luciferin-regenerating enzyme genes

Preliminary studies have suggested that oxyluciferin could enzymatically recycle to luciferin by luciferin-regenerating enzyme (LRE) in fireflies [145-147]. LRE shows a at most 39 amino acid sequence identity with insect anterior fat protein (AFP) (a parter of heximerin acceptor) and mammalian senescence marker protein-30 (SMP-30) [148 149, 150], and thought to be a novel SMP30-like protein based on its enzymatic function in insects [151]. The recent evidence suggest that LRE may perform other functions in cells and its role in recycling luciferin in vivo requires further clarification [67]. Even still with uncertainty on the role of LRE in the recycle of luciferin, we still investigate the LRE in the genomes of luminous and non-luminous beetles.

To identify genes encoding LRE, four cloned LRE genes of fireflies (*P. pyralis*, A-LRE, BAB60700; *Lampyris turkestanicus*, T-LRE, ADK55065; *Luciola cruciata*, G-LRE, BAB85479; *Luciola lateralis* (=*A. terminalis*), H-LRE, BAB85478) [152-154] were used to search homologs in six luminous beetles and other beetles genomes using BLSTP (E-value = 1e-20). The candidate genes were further filtered by removing less than 50% identity and duplication. We identified 33 LREs (Data S26),

consistent to annotation with NR and UniProt database. No PTS signals were identified for all LRE genes.

The protein sequences from 30 LREs including identified, cloned and three LREs of firefly *Luciola aquatilis* (c10156, c12106 and c8279) [109] were aligned with MAFFT with BLOSUM62 matrix and L-INS-i iterative refinement method and the unaligned regions were trimmed using trimAl (gt = 0.5) [110]. The gene tree was constructed using RAxML software (--auto-prot=ml -f a -x 12345 -N 100 -p 12345 -m PROTGAMMAWAG) [33] with DmeSMP30 (*CG7390*) as an outgroup. Our tree (Figure S51) shows that LRE form two clades with all LRE identified the genomes of each species of luminous Elateroidea in two separated clades (most in terminal clade and a few in basal clade).

LyuLRE1 (terminal clade) and LyuLRE2 (basal clade) in different clades have low transcriptomic expression (Figure S52) and can be identified in proteome of male luminous organ (Figure S54). AteLRE5 (basal clade) have higher transcriptomic expression, and, together with AteLRE2 and AteLRE3 (terminal clade), can be identified in in proteome of male and female luminous organs, while AteLRE1 (terminal clade) can be identified only in the proteome of female luminous organ (Figure S54).

S7.12 Transfer of protein and metabolite across the peroxisomal membrane

All the proteins that function in the peroxisomes (incl. peroxisomal membrane and matrix proteins) are encoded in the nuclear DNA, synthesized on cytosolic ribosomes and directed by sequenced-encoded signals, which can be recognized by homologous sets of organelle biogenesis proteins (i.e.) peroxins) to the organelles [155]. In addition, large soluble metabolites such as acyl-CoAs and coenzymes can be transported into peroxisomes by ABC transporters, whereas smaller metabolites are transported through pores created by proteins [155]. Photocyte peroxisome is thought as the place of light reaction and luciferin biosynthesis [156]. Thus, we investigated the possibility that main enzymes and precursor metabolites related to luciferin biosynthesis are transported into peroxisomes.

S7.12.1 Peroxisomal targeting signals of proteins

Proteins destined for import into the peroxisomal matrix or insertion into the peroxisomal membrane follow distinct pathways [157]. Peroxisomal targeting signals (PTS) have been found in almost all peroxisomal proteins residing either in lumen or

in the limiting membrane of peroxisomes[158]. Soluble matrix proteins can use one of two peroxisomal targeting signals (PTS) (PTS1 at the C-terminus or PTS2 at the N-terminus) conserved throughout evolution to direct them into peroxisomal lumen. By combining PTS1 and PTS2 motifs reportedly from diverse species [159, 160] with those in the Eukaryotic Linear Motif database [161], we summarized the consensus sequences of PTS1 as [A/C/H/K/N/P/S/T]-[H/K/N/Q/R/S]-[A/F/I/L/M/V] or [K|R|H]-[S|A|P|T|C]-[N|T|S]-[L|M|F|I], and PTS2 as R-[^P]-[^P]-[^P]-[L|I|V|H]-[^P]-[^P]-[H|Q]-[L|I|F|A]. Insertion into the peroxisomal membrane proteins (PMP) have been identified to include two routes: from the cytoplasm directly to the peroxisomal membrane (cytoplasm-to-peroxisome pathway) or sorting indirectly to peroxisome by way of ER-derived vesicles or a specialized subdomain of the ER, the peroxisoamle endoplasmic reticulm (pER) (pER-to-peroxisome pathway) [162]. The targeting signals for PMPs (mPTS) requires the presence of at least one transmembrane domain for its functionality [163], and includes at least two different types, mPTS-I and mPTS-II [164]. mPTS-I has been linked to the transport of proteins from the cytosol to peroxisomes and its consensus sequence is X-X-X-[C/F/I/L/T/V/W]-X-X-[A/C/F/I/L/Q/V/W/Y]-[C/I/L/V]-X-X-[A/C/F/I/L/V/W/Y]-[I/L/Q/R/V]-X-X-X) [164]; mPTS-II might correspond to the mPTS for the alternative route via the ER but its consensus sequence is poorly understood [164].

Due to still no identified consensus sequence for mPTS-II, we identified PTS1, PTS2 and mPTS-I motifs based on the above described consensus sequences in protein sequences of all genes of the whole genomes of 11 beetles and fruit fly (Table S48). GO/KEGG enrichment analysis indicated that those proteins with PTS in luminous beetles are mainly related to peroxisome, ABC transporters and fatty acid degradation (Data S28). More importantly, we also scrutinized and summarize the PTS of all above identified genes (gene families) in Note S7 (7.1-7.11), and the results were described there (Figures S41-S43, S48-S50).

S7.12.2 Peroxin

The currently known peroxins (Pex) play a role in the targeting and insertion of peroxisomal membrane proteins (PMPs), the import of matrix proteins into the peroxisomal matrix, as well as the proliferation, division or inheritance of peroxisomes [165]. Of which, Pex5 and Pex7 are the cytosolic receptors for PTS1 containing proteins and PTS2 containing proteins, respectively, which recognize the PTS signals of corresponding matrix proteins imported into peroxisomes [166-168]. Of which, Pex13 and Pex14 are the docking complex which target the cargo-receptor

complex to peroxisomes during the peroxisomal matrix protein import [169]. For the PMP import, only three Pex, Pex3, Pex16 and Pex19, are involved [170-172]. To explore Pex role in firefly, we identified and analyzed the Pex in whole genomes.

15 Pex genes of *D. melanogaster* from FlyBase (http://flybase.org) were used as a set to BLASTP (E-value = 1e-5) against the genomes of luminous and non-luminous beetles. After filtering those with less than 30% identity and 50% coverage, 147 Pex genes were obtained in 11 species (Data S26).

The amino acid sequences of 147 Pex genes along with 15 Pex genes of *D. melanogaster* were aligned using MAFFT [78] with BLOSUM62 matrix and FFT-NS-i iterative refinement method. The unrooted gene tree was constructed using RAxML (--auto-prot=ml -f a -x 12345 -N 100 -p 12345 -m PROTGAMMAWAG) [33], and visualized and colored using iTOL [41]. The classification of Pex genes were performed according to the best hit and phylogenetic relationship (Figure S55).

The expression of Pex genes was showed at Figure S57 that Pex5 and Pex14 presented higher expression in luminous organs of six luminous beetles at both transcriptional proteomic level in luminous organs of *L. yunnana* and *A. terminalis*, corresponding to the function that Pex5 targets the PTS1 proteins to peroxisomal membrane by docking onto a peroxisomal membrane protein (PMP), Pex14 [155]. While Pex7 had no expression, indicating that Pex5-PTS1 import manner is the most important in firefly. In addition, Pex3 and Pex19 involved in the PMP import showed expression at both transcriptiomic and proteomic levels in luminous organs of *L. yunnana* and *A. terminalis*.

S7.12.3 Peroxisomal membrane channel

Pxmp2 (peroxisomal membrane protein 2) is the first peroxisomal membrane channel, which has been confirmed to allow free diffusion across the membrane of compounds with molecular masses below 400 Da [173]. To study the cysteine (molar mass: 121 Da) across the peroxisomal membrane, we identified and analyzed the Pxmp2 genes in beetles and *D. melanogaster*.

To identify Pxmp2 family genes, we searched the domain PF04117 (Mpv17/PMP22 family) against the annotated domain of genes of beetles and *D. melanogaster* genome using Pfam database (E-value = 1e-10). After deleting abnormal genes, 67 Pxmp2 family genes were acquired.

To classify identified 67 Pxmp2 family genes to three subfamilies (Pxmp2, Mpv17 and Mpv17-L), we constructed the gene tree with collected Pxmp2 family genes (*H. sapiens*: NP_061133-HunPxmp2, NP_002428-HunMpv17, NP_001121895-HunMpv17-L; *Mus musculus*: NP_033019-MusPxmp2, AAA39736-MusMpv17, AF305634_1-MusMpv17-L) using RAxML [33] after aligned using MAFFT [78] with BLOSUM62 matrix and FFT-NS-2 iterative refinement method. The Pxmp2 genes were determined that one gene in each luminous species (Figure S56).

The expression of Pxmp2 genes in *L. yunnana* and *A. terminalis* presented higher expression at transcriptional level, and also presented higher abundance at proteomic level in *L. yunnana* (Figure S57).

S7.12.4 ATP-binding cassette (ABC) transporter of the peroxisomal membrane

ABC (ATP-binding cassette) protein family is one of the largest transporter families, and the majority of these ABC proteins function as primary-active transporters to transport substrates across lipid membranes by binding and hydrolysis of ATP [174]. In arthropods, ABC gene family can be classified into 8 subfamilies (A, B, C, D, E, F, G, H) [174]. In humans, ABC-D are involved in the import of long and branched chain acyl-CoA molecules into the peroxisome [175], which is the place of bioluminescence in luminous beetles. Interestingly, we found that one orthologs annotated as ABC-D are positively selected in the ancestor of luminous beetles (*LY01293*, *LT01539*, *AQULA_001865_PA*, *PPYR_12097_PA*, *maker-scaffold384-snap-gene-2.575*, *ILUMI_23200_PA*) (Data S8). *LY14844* (ABC-D) was also positively selected in *L. yunnana*. Thus, we thoroughly identified ABC-D proteins in luminous beetles and other insects.

To identify ABC-D genes, BLASTP searches (E-value = 10^{-10}) were performed on insect genomes using the reported ABC-D protein sequences of human and *Drosophila* [176] as queries, which were retained with more than 50% identity. The conserved nucleotide binding domain (NBD, PF00005.24) and transmembrane domain (TMD, PF00664.20) were scanned by the HMMER 3.2 [75]. After removing redundancy, putative transporter genes were retained. Totally, 29 putative ABC-D genes were obtained in 12 species (Data S29). Of which, the identified ABC-D genes of *T. castaneum*, *B. mori* and *D. melanogaster* are identical to those previously reported [177, 178].

To infer orthologous relationships of ABC-D in insects, we constructed the maximum likelihood (ML) phylogenetic tree using the RAxML [33] with settings "-f a -x 12345 -N 100 -p 12345 -m PROTGAMMAWAG" after multiple alignments by MAFFT [78] and removed poor aligned regions with trimAl (gt=0.5) [110], which was displayed and edited using iTOL [41]. Our tree showed that all of ABC-D genes were perfectly clustered into two clades, of which luminous species were clustered (Figure S58a). The clear orthologous relationships between insect ABC-D and human ABCD1/2 and ABCD3/4 suggest that their function maybe conserved [174].

In addition, we analyzed the expression patterns of ABC-D genes in *A. terminalis* and *L. yunnana* at transcriptomic and proteomic levels (Figure S59). *LY01293* of *L. yunnana* and *LT01539* of *A. terminalis* orthologous with Homo_ABCD3 (Figure S58a), had same expressive patterns that they were high expressed at transcriptomic level in luminous organs and higher expressed at proteomic level in only female luminous organ (Figure S59), which could involve in the import of long and branched chain acyl-CoA molecules into the peroxisome [179]. By analyzing selected sites, we found two positive selected sites (G (S, A) ->C; G (A, F) ->Y) located in 2nd transmembrane (TM) segment of transmembrane domains (Figure S58b). So, we speculate that the variation of these two sites in luminous beetles may promote import of homogentisic acid (2,5-dihydroxyphenylacetic acid) into peroxisome in homogentisic acyl-CoA (i.e. branched chain acyl-CoA), which may be produced by one of 4CL with high expression in luminous organs.

Supplementary Note 8. Genome editing of homeobox gene *Abd-B* in *A. terminalis*

Abdominal-B (*Abd-B*) is a Hox gene that plays a critical role in determining cell fate in the tail end of organisms and expressed in abdominal 8-10 segments (A8-A10) and telson [180]. The transcript depletion of *Abd-B* in *Photuris* fireflies by RNAi resulted in the extensive disruption of adult lantern, suggesting that the evolution of adult lanterns involved the acquisition of a novel regulatory role for the Hox gene [181]. Loss of function of *Abd-B* resulted in the transformation of A10 to an A9 identity [182]. In Lepidoptera, mutation or decreased expression of *Abd-B* causes the development of extra prolegs on all segments posterior to the sixth abdominal segment (A6) in silkworm larvae [183], butterflies *P. xuthus* [184] and *P. machaon* [185]. The homeobox region plays a pivotal role in the morphogenetic and regulatory functions of the *Abd-B* gene [186-189]. Considering that *Abd-B* has mutated phenotype easily observable and also is related to luminous organ development, we selected the homeobox region of the *Abd-B* gene to perform CRISPR/Cas9 gene editing in firefly *A. terminalis* (Figure S60).

S8.1 Target site identification, sgRNA and Cas9 mRNA preparation

Target site selection and sgRNA preparation mainly follow the methodology in our previous studies [185, 190]. In brief, to check the integrity of *Abd-B* gene annotation in the *A. terminalis* reference genome, we aligned *Abd-B* coding sequences of *A. terminalis* against those of firefly *Photuris sp* (KC190488) [181], other beetle *T. castaneum* (*TC000889*) [191], butterflies *P. xuthus* (*Px_03961_Abd-B*) [184], silkworm *B. mori* (AB461857) [183], and fruit fly *D. melanogaster* (*CG11648-PB*) [187]. Among them, the *Abd-B* gene functions and sequences in silkworm and fruit fly are well studied [183, 187]. Our alignment data suggested that the *Abd-B* gene (*LT07795*) of *A. terminalis* was well annotated, and the homeobox region located in the second exon was conserved among beetles.

To identify all targets located completely or partly in the coding region, using the method described by Chen et al [192], we first searched sequences corresponding to N_{20}NGG in the gene of *Abd-B* of the sense and antisense strands of DNA. We then searched the candidate target sequences using BLAST against the *A. terminalis* genome to eliminate those with off-target sites using strict criteria, where the editable candidate sites were defined only when the seed region (12 nucleotides (nt) to protospacer adjacent motif (PAM) NGG) was unique [193]. We further selected those candidate sites in the exons with the first two bases of GG. Totally, eight sgRNAs (2nd exon: T22053, T22120, T22341, T22349, T22355; 3rd exon: T23987, T23999, T24286) (Table S49; Figure S61) were selected to target double-strand break in the second exon and third exon of *Abd-B*.

We used Sanger sequencing to check whether any SNPs or indels differed from the reference genome in the different individuals. Genomic DNA was extracted from male adults. We amplified and sequenced the gene targeting fragment in the second exon, using pair primers LT07795_ex2-F1/R1 (LT07795_ex2-F1: CGATATTCTAAAAGAGATT; LT07795_ex2-R1: GACTAAATGTATCTGGTGTTG), and in the third exon using pair primers LT07795_ex3-F3/R3 (LT07795_ex3-F3: AATTTGTGTTGGTTTGATTCG; LT07795_ex3-R3: ATGGTGATTAACTACATGGT) (Table S49; Figure S61). The Sanger sequencing data showed that all these candidate target sites were identical to those in the reference genome. All these targets were selected for sgRNA synthesis by PCR-based strategies [184, 194]. Preparations of sgRNAs were the same as previously described [184, 185]. Recombinant Cas9 protein (PNA Bio Inc, CA, USA) was used.

S8.2 Gene injection, phenotypic observation and genotyping

Our pilot gene-editing experiment began in 2017, aiming to develop the methodology of firefly gene editing. To test which target site(s) works better, we combined all five sgRNA targets in 2nd (I) and all three targets of 3rd exons (II, III, IV), respectively (Table S50). After larvae were hatched, we first observed abdominal morphology including leg number, segment outline etc. No redundant prolegs were observed in all injected individuals, but three abnormal individuals at the end of the abdomen were observed in I treatment (Figure S62c-h). We picked up two of them for genotyping, which were verified to be knocked out in T22120, T22341-T22349-T22355 (Figure S63), suggesting that T22120, T22341-T22349-T22355 may be ideal targets. 4 mixed larvae in II and 11 mixed larvae in III were picked up for genotyping, all of them without observably morphologically mutation, but no disruption was observed, suggesting that target sites in the third exon may be unsuitable.

Based on the results of 2017, we found that moisturizing is a prerequisite for the success of all experiments from oviposition, injection, to hatch. In 2018, we went deep into the Cas9 experiment with targets of T22120, T22341 and T22355. As expected, we got a high percentage of morphologically mutants (Table S51) with abnormal ventrites of 9th and 10th and no luminous organs in the ventral side of 8th ventrite, or with only three abdominal ventrites in one more serious case (Figure S62i-j). We picked up 3 mutants (abnormal ventrites of 9th and 10th and no luminous organ) to genotype and also found them with 100% mutated ratio in all target sites (Figure S64).

S8.3 The expression pattern of genes in mutants and wild types

In order to check the effect of disruption of target gene *Abd-B* on the expression of itself and the related genes regulated by it at the RNA level, we performed transcriptomic sequencing for mixed 14 mutants and mixed 14 wild type larvae from 2018 experiment. Total RNA was extracted using the guanidinium thiocyanate-phenol-chloroform extraction method (Trizol, Invitrogen) according to manufacturer's protocol. RNA sequencing libraries were generated using Illumina mRNA-Seq Prep Kit. The 350 bp insert size paired-end libraries were sequenced using Illumina HiSeq4000 sequencers with read length of PE150. After removing adapter sequences, about 6 Gb raw reads were generated for each sample. Raw reads were filtered using NGSQCToolkit_v2.3.3 [50] to get high-quality clean reads for further analysis. The filtered clean reads were firstly mapped the *de novo* assembled genome of *A. terminalis* using Tophat [53], and then the program Cufflinks, Cuffmerge, Cuffnorm of Cufflinks software package [24] were used to calculate FPKM values in

mutant and wild type larvae. The differentially expressed genes (DEGs) were analyzed using Cuffdiff of Cufflinks software package.

In total, 259 DEGs were identified with the total FPKM ≥ 1, the absolute value of logFC ≥ 2 and P value ≤ 0.01 from mutant and wild type larvae, of which 96 genes were down-regulated and 163 genes were up-regulated in *A. terminalis* mutant (Figure S65; Data S30).

96 down-regulated genes in *A. terminalis* mutants are enriched in ubiquinone and quinone, bioluminescence, sulfotransferase, beta-galactosidase in GO (Table S52) and phenylalanine and tyrosine metabolism in KEGG (Table S53). 163 up-regulated genes in *A. terminalis* mutants are enriched in L-cysteine metabolic process (Data S31), which suggested alternative metabolism of L-cysteine in the case of luciferin synthesis blockage.

We also noted that the expression of *Abd-B* gene in mutant (FPKM= 15.512) was higher than in wild (FPKM= 5.599). By checking the reads mapping in mutant and wild, we found decreased reads mapping in the region of target sites in mutants, although a higher FPKM in mutant in whole *Abd-B* gene (Figure S66).

Analyzing the expression pattern**,** we found that the expression of main genes related to cysteine metabolism was up-regulated in mutant compared with wild type except for MMT and CGL with almost unchanged or slightly lower, especially for CDO and CASD that degrading L-cysteine increased 6~9 times (Fig. 5; Figure S67a). Also, we found that the expression of main genes related to tyrosine metabolism (PAH, TAT, TH, HPPD, HD, MAAI, FAH) was higher (FPKM >120) and was up-regulated in mutant compared with wild type except HD and MAAI reduced, especially for TH degrading tyrosine increased 68 times (Fig. 5; Figure S67b). The data indicated that increase the degradation of L-cysteine and tyrosine in the case of luciferin synthesis blockage.

Supplementary Note 9. The genes related to flash control

The mysterious process by which fireflies can control their flashing has inspired over a century of careful observation but has remained elusive [195]. Oxygen (O_2) is thought as an essentially controlling element in the photochemical reaction [196, 197], and its access to light-emitting photocytes is permitted while mitochondrial respiration in lantern cells is reversibly inhibited by nitric oxide (NO), which is induced by the

neurotransmitter octopamine (OA) [198-200] and synthesized upon activation of nitric oxide synthase (NOS) by calcium/calmodulin [201]. OA can induce the accumulation of cyclic AMP by stimulating adenylate cyclase in firefly light organ, thus suggesting the importantance of octopamine receptors in flashing control [202]. OA has a crucial role in triggering protein synthesis-dependent physiological adaptations to protect central nerve system (CNS) function during heat stress by activating a cAMP/PKA pathway in locust (*Locusta migratoria*) [203]. An alternative mechanism of oxygen access to photocytes is by modulating the levels of fluid in the tracheoles supplying photocytes, providing a variable barrier to oxygen diffusion [204]. In addition, Ghiradella and Schmidt questioned the drawbacks of oxygen-control theory of firefly flash, and proposed an alternative mechanism of flash control that hydrogen peroxide triggers the flash [195]. Whether in oxygen-control model or hydrogen peroxide-control model, it is accepted that the synthesis of NO in tracheolar end cells induced by octopamine through the pathway of G-protein coupled receptor cAMP/PKA-Ca/Calmodulin signaling cascade is very important.

Calcium ions (Ca^{2+}) are essential in producing the intense, long lasting scintillation response and are therefore possibly implicated in the final stages of flash control in firefly *Photuris versicolor* [205]. It is possible that interaction of Ca^{2+} with the mitochondria may initiate the light reaction [205]. Mitochondria form junction with endo/sarcoplasmic reticulum (SR/ER) and these junctions play a pivotal role in mediating calcium signal propagation to the mitochondria, which is the main place for ATP production and reactive oxygen species [206-208]. Ca^{2+} is a key regulator of mitochondrial function and acts at several levels within the organelle to stimulate ATP synthesis [209]. Intriguingly, we identified a few positive selected genes in the ancestor of luminous beetle related to Ca^{2+} transport and store (i.e. sarcoplasmic/endoplasmic reticulum (SR/ER) calcium transporting ATPase (SERCA), calreticulin-like protein). Thus, we investigated the main components of calcium signaling system to explore the possible roles of Ca^{2+} in flash control (Data S32; Figure S68).

S9.1 Receptor: Octapamine receptor

Insect octopamine receptors are G-protein coupled receptors, and they can be coupled to second messenger pathways to mediate either increases or decreases in intracellular cyclic AMP (cAMP) levels or the generation of intracellular calcium signals [210]. We first identified all biogenic amines receptor, and then separated those octopamine receptors (OAR) by phylogenetic analysis, especially with reference to those of *D. melanogaster* (Dme) and *T. castaneum* (Tca).

The candidate genes encoding biogenetic amine GPCRs were identified preliminarily using 21 biogenetic amine GPCRs from *D. melanogaster* [211-217] and 20 biogenetic amine GPCRs form *T. castaneum* [218] as queries to BLASTP (E-value = 1e-20) against the genomes of six Elateroidea beetles (five fireflies: Lyu, Ate, Ala, Ppy, Ppe; and one luminous click beetle (Ilu)) and other six non-Elateroidea beetles (Ota, Dpo, Apl and Agl). Subsequently, the homologs were selected based on three criteria: (a) more than 35% identity of protein sequences; (b) commonly identified in *T. castaneum* and *D. melanogaster*; (c) 7 transmembrane receptor (7TM) (rhodopsin family) domain (PF00001) was searched using HMMER 3.2 [75] and more than four transmembrane segments were predicted by TMHMM 2.0 (http://www.cbs.dtu.dk/services/TMHMM-2.0).

The protein sequences of biogenetic amine GPCRs candidate were aligned by MAFFT with BLOSUM62 matrix and FFT-NS-i iterative refinement method, and then the maximum likelihood (ML) tree was constructed using RAxML software (--auto-prot=ml -f a -x 12345 -N 100 -p 12345 -m PROTGAMMAWAG) [33] with neuropeptide Phe-Met-Arg-Phe-NH2 (FMRFamide) of *D. melanogaster* (DmeFMRFa, *CG2346*) as an outgroup. The tree was visualized and beautified using iTOL [41]. Combined the phylogenetic data (Figure S69) with referring to reported OAR in *D. melanogaster* [219, 220], total 45 OARs were identified in beetles (Lyu: 3; Ate: 4; Ala: 3; Ppy: 5; Ppe: 4; Ilu: 4; Ota: 6; Dpo: 4; Apl: 4, Agl: 3; Tca: 5) (Data S32). Among them, *L. yunnana* have three OAR (Lyu11 (DmeOctalpha2R clade), Lyu5 (DmeOctbeta2R clade), and Lyu3 (DmeOctbeta1R clade)), while *A. terminalis* have four OAR (Ate4 (DmeOctalpha2R clade), Ate11 (DmeOctbeta2R clade), Ate12 (DmeOctbeta1R clade) and Ate7 (DmeOamb clade)).

S9.2 Transducers

S9.2.1 Guanine nucleotide-binding protein alpha subunit

Heterotrimeric Guanine nucleotide-binding (G) proteins (Gα·GDP/βγ) are intracellular partners of G-protein-coupled receptors (GPCRs), and upon interaction with an activated receptor, its α subunit (Gα) exchanges bound GDP for GTP and then target such effector as adenylyl cyclases (AC) [221, 222]. In the *D. melanogaster* genome, 6 genes have been described to encode for Gα proteins: *Gs*, *Gi*, *Gq*, *Go*, *Gf* and *concertina* (*cta*) with each gene activating different transduction pathways [223]. The associated pathways for *Gs*, *Gi*, *Gq* are well known to increase cAMP (*Gs*), decrease

cAMP (*Gi*), and release IP3 and diacylglycerol (*Gq*) [223, 224]. Here we identified all Gα gene in the genomes of fireflies and other beetles.

The six Gα gene from *D. melanogaster* (DmeGs: *CG2835*; DmeGi: *CG10060*; DmeGq: *CG17759*; DmeGo: *CG2204*; DmeGf: *CG12232*; Dmecta: *CG17678*) [223, 225] were used to BLATSP (E-value = 1e-10) against the genomes of 11 beetles (six Elateroidea beetles (five fireflies: Lyu, Ate, Ala, Ppy, Ppe; and one luminous click beetle (Ilu)) and other six non-Elateroidea beetles (Ota, Dpo, Apl and Agl)) and one silkworm. Those protein sequences with scores higher than 200 and 50% coverages were selected as candidates, which were scanned using HMMER 3.2 [75] to confirm their Gα domain (PF00503) with more than 60% protein sequence identity. Total 84 Gα genes were identified in investigated genomes, which were classified based on the best hit of BLASTP (Data S32). These 84 Gα genes, together with six *D. melanogaster* Gα genes, were aligned using MAFFT [78] with BLOSUM62 matrix and L-INS-i iterative refinement method and the poorly aligned regions and gaps were removed using trimAl with settings "gt=0.5" [110]. The unrooted maximum likelihood (ML) tree was constructed using RAxML [33] with settings "--auto-prot=ml -f a -x 12345 -N 100 -p 12345 -m PROTGAMMAWAG", and visualized and modified using iTOL [41]. Our ML Gα tree shows that different Gα subunits of fireflies clustered with correspondent type of other insects (Figure S70).

S9.2.2 Adenylate cyclase

Adenylyl cyclase (AC) is the prototypical second messenger (adenosine 3'5' monophosphate (cAMP) generator, and signaling through cAMP is known to be important in virtually every cell [226, 227]. Functional and ultrastructural investigations have shown that AC is intimately associated with sites of calcium ion entry into the cell [226].

Ten cloned AC genes (*CG9533*, *CG10564*, *CG1506*, *CG7978*, *CG9210*, *CG17176*, *CG17174*, *CG5983*, *CG5712*, *CG17178*) from *D. melanogaster* [228-232] were used to build a HMM profile using hmmbuild program, which was used to search the genomes of 11 beetles (six Elateroidea beetles (five fireflies: Lyu, Ate, Ala, Ppy, Ppe; and one luminous click beetle (Ilu)) and other six non-Elateroidea beetles (Ota, Dpo, Apl and Agl)) and one silkworm using hmmsearch using HMMER 3.2 [75]. The genes with domain score higher than 300 were firstly chosen as candidates. Because the ACs are were predicted to cross the plasma membrane about 12 times in 2 cassettes of about 6 transmembrane-spanning domains with each cassette followed by a large cytosolic domain [233, 234], we further chose those genes with two cytosolic domains

(PF00211, adenylate and guanylate cyclase catalytic domain) and transmembrane-spanning domains as AC candidate genes. In total, 59 AC genes were acquired in the genomes of 11 beetles, one silkworm and one fruit fly (Data S32).

The protein sequences of all AC genes were aligned using MAFFT [78] with BLOSUM62 matrix and L-INS-I iterative refinement method and were trimmed unaligned regions using trimA1 [110] (gt=0.5). The maximum likelihood (ML) phylogenetic tree was constructed using RAxML [33] with settings "--auto-prot=ml -f a -x 12345 -N 100 -p 12345 -m PROTGAMMAWAG", and visualized and modified using iTOL [41]. Our tree showed that firefly AC clustered with different clades of other beetles and fruit fly (Figure S71).

S9.2.3 cAMP-dependent protein kinase

The classical downstream target of cAMP is the protein kinase A (PKA) family of protein [235]. In the absence of cAMP, PKA is an enzymatically inactive tetrameric holoenzyme consisting of two catalytic subunits (C) bound to a regulatory subunit (R) dimer [236]. There are multiple isoforms of R and C subunits of PKA [236]. The cAMP/PKA has three main actions on Ca^{2+} signaling. First, it stimulates voltage-dependent calcium channel (VDCC) to increase the amount of Ca^{2+}. Second, it phosphorylates phospholamban to reduce its inhibitory effect on the sarco(endo)plasmic Ca^{2+}-ATPase (SERCA) pump, which then can increase the Ca^{2+} concentration so that more Ca^{2+} is released from the SR. Third, cAMP/PKA phosphorylates the ryanodine receptors (RYRs), thereby enhancing their ability to release Ca^{2+} from endo/sarcoplasmic reticulum (ER/SR).

The six PKA genes of *D. melanogaster* (Dme) from FlyBase (http://flybase.org/) [237-240], including four catalytic (C) subunit (*CG12069*; Pka-C1: *CG4379*; Pka-C2: *CG12066*; Pka-C3: *CG6177*) and two regulatory (R) subunit (Pka-R1: *CG4234*1; Pka-R2: *CG15862*), were used to identify PKAs via BLATSP (E-value = 1e-20) against the genomes of 11 beetles (six Elateroidea beetles (five fireflies: Lyu, Ate, Ala, Ppy, Ppe; and one luminous click beetle (Ilu)) and other six non-Elateroidea beetles (Ota, Dpo, Apl and Agl)). The candidate genes were selected with more than 45% identity and 50% coverage in protein sequences. In total, we acquired 65 PKAs in 11 beetles and fruit fly (Data S32).

The protein sequences of candidate genes were aligned using MAFFT with BLOSUM62 matrix and L-INS-I iterative refinement method and were trimmed unaligned regions using trimA1 (gt=0.3). The maximum likelihood (ML) tree of

PKAs was constructed using FastTree 2 [34] with default parameters with *D. melanogaster* PKG (*CG3324*) as an outgroup and was visualized and beautified using iTOL [41]. The tree showed that firefly PKA, together with those of other beetles, form five clades with different *D. melanogaster* PKA subunits clade (Figure S72). Thus, like in *D. melanogaster*, firefly PKAs can be classified into three catalytic (C) subunits and two regulatory (R) subunits.

S9.2.4 Phosphoinositide-specific phospholipase C

Phosphoinositide-specific phospholipase C (PLC) enzymes are common signaling components linked to the activation of most cellular receptors [241]. PLC enzymes found in eukaryotes comprise a related group of proteins that cleave the polar head group from phosphatidylinositol 4,5-bisphosphate (PtdIns(4,5)P2) [242, 243]. Its best documented consequence and also a major cell signaling response, is the generation of two second messengers: inositol 1,4,5-triphosphate (Ins(1,4,5)P3), a universal calcium-mobilizing second messenger, and diacylglycerol (DAG), an activator of several types of effector proteins including protein kinase C isoforms [241]. PLC activated ER/SR to release Ca^{2+} by Ins(1,4,5)P3 combined with Ins(1,4,5)P3 receptor (Ins(1,4,5)P3R).There are six families of mammalian PLC enzymes (PLCβ, γ, δ, ε, ζ and η) consisting of 13 isoforms in humans, all of which hydrolyses PI(4,5)P2 with different kind of activated mechanism [241, 244]. For examples, PLCβ is activated by heterotrimeric G-protein subunits following GPCR-ligand binding, and PLCγ is recruited via its SH2 domain to activated receptor tyrosine kinases at the plasma membrane [244]. The *Drosophila* genome encodes three PLC genes: two highly related to the PLCβ4 subfamily of mammalian enzymes and a single PLCγ (sl) [244]. Here, we identified PLC in firefly genomes.

149 known PLC protein sequences downloaded from UniProt database (https://www.uniprot.org) including *D. melanogaster* and human (*Homo sapiens*) were used to BLASTP (E-value = 1e-5) against the genomes of 11 beetles (six Elateroidea beetles (five fireflies: Lyu, Ate, Ala, Ppy, Ppe; and one luminous click beetle (Ilu)) and other six non-Elateroidea beetles (Ota, Dpo, Apl and Agl)) and one silkworm. The Phosphatidylinositol-specific phospholipase c domains (PI-PLC-X, PF00388 and PI-PLC-Y, PF00387) were scanned using HMMER 3.2 [75]. After removing redundancy, the retained best hits for putative PLC genes were further verified using BLASTP (E-value = 1e-5) against non-redundant NCBI protein sequences. In total, 53 PLC genes were identified, of which 3~6 PLC genes existed in each species (Data S32; Table S54).

To classify the PLC genes into different subfamilies, all amino acid sequences were phylogenetically characterized in each case. For this, protein sequences were aligned using MUSCLE 3.8 [30] and poorly aligned regions and partial gaps were removed with trimAl (gt=0.5) [110]. Phylogenetic tree was constructed using a maximum likelihood method in the program package RAxML [33] and displayed and edited using iTOL [41]. Our phylogenetic analysis indicated that all insect PLC genes were clustered into PLCβ, PLCε and PLCγ, and no PLCδ, PLCη and PLCζ genes were founded in the insects (Figure S73).

S9.3 The calcium channels

Signal Ca^{2+} is derived either from internal stores (usually the endoplasmic reticulum (ER) or its muscle equivalent, the sarcoplasmic reticulum (SR),) or from the external medium. The release of Ca^{2+} from the internal stores is controlled by Ca^{2+} itself, or by an expanding group messengers acting on inositol-1,4,5-triphosphate receptor (Ins(1,4,5)P3R) or ryanodine receptor (RYR), while many different plasma-membrane channel control Ca^{2+} entry from the external medium in response to stimuli that include membrane depolarization, stretch, noxious stimuli, extracellular agonists, intracellular messengers and the depletion of intracellular stores [245, 246]. Here, we identified those genes related to these channels.

S9.3.1 Ryanodine receptors and Inositol-1,4,5-trisphophate receptors

Ryanodine receptors (RYRs) and inositol 1,4,5-trisphosphate receptors (Ins(1,4,5)P3Rs) are Ca^{2+} release channels on the endo/sarcoplasmic reticulum(ER/SR) [246-251]. While these proteins are represented by three different isoforms in vertebrate, the *D. melanogaster* genome contains a single InsP3Rs and RyRs genes [252].

The protein sequences of known 67 insect RYRs and three human RYRs were downloaded from the UniProt database (https://www.uniprot.org) and used to BLASTP (E-value =1e-5) against the genomes of 11 beetles (six Elateroidea beetles (five fireflies: Lyu, Ate, Ala, Ppy, Ppe; and one luminous click beetle (Ilu)) and other six non-Elateroidea beetles (Ota, Dpo, Apl and Agl)) and one silkworm. The conserved domains (Inositol 1,4,5-trisphosphate/ryanodine receptor: PF08709; Ion transport protein: PF00520; MIR domain: PF02815; RyR and IP3R Homology associated: PF08454; Ryanodine Receptor TM4-6: PF06459; RIH domain: PF01365; RyR domain: PF02026; SPRY domain: PF00622) were used to scan the candidates by the HMMER 3.2 [75]. After removing redundancy, the best hit for RYRs genes were retained and further verified using BLASTP versus non-redundant NCBI protein

sequences. Ins(1,4,5)P3Rs were identified using similar method used in RYRs identification. The protein sequences of 56 known insect Ins(1,4,5)P3Rs genes and 3 human RYRs were downloaded from the UniProt database (https://www.uniprot.org) and the scanned conserved domains were inositol 1,4,5-trisphosphate/ryanodine receptor (PF08709), ion transport protein (PF00520), MIR domain (PF02815) RIH domain (PF01365) and RIH domain (PF01365). Our data indicates that at least one RYR gene and one Ins(1,4,5)P3R exist in each investigated insect species, except for *I. luminosus* which could lost Ins(1,4,5)P3R gene, while *I. luminosus* and *B. mori* have two RYRs genes (Data S32).

The protein sequences of all RYRs and Ins(1,4,5)P3Rs were aligned using MUSCLE 3.8 [30] and the poorly aligned regions and partial gaps were removed with trimAl (gt=0.5) [110]. Phylogenetic tree was constructed using maximum likelihood method using RAxML [33], and displayed and edited using iTOL [41]. The phylogenetic analysis indicated that all insect RYRs and Ins(1,4,5)P3Rs genes were clustered into two clades representing RYRs and Ins(1,4,5)P3Rs, respectively, which indicated that the two genes were highly conserved (Figure S74).

S9.3.2 The voltage-dependent calcium channel

Voltage-dependent Ca^{2+} channels (VDCC) serve as one of the important mechanisms for Ca^{2+} influx into the cells, enabling the regulation of intracellular concentration of free Ca^{2+} [253], and can be activated by G protein-cAMP-PKA signaling pathway [254-256]. VDCC form heterooligomeric assemblies that are typically comprised of a pore-forming α1 subunit associated with accessory subunit α2δ, β, γ [257-259]. The *D. melanogaster* genome encodes three α1 genes (Dmca1D, Dmca1A, and Ca-α1T) subunits, which are correspondent to Cav1-, Cav2-, and Cav3- type channels of vertebrate, respectively [252]. Channels formed by Dmca1D are dihydropyridine (DHP) sensitive, similar to L-type vertebrate channels, and are expressed in the embryonic nervous system and adult muscles [260-262]. The Dmca1A channel is insensitive to DHP and widely expressed in the embryonic nervous system [263].

We first extracted all annotated VDCC genes in 13 insect species using the UniProt database [264], and then filtered those with protein length less than 300 aa. The 83 candidate VDCC genes were acquired in 13 insect species (Data S32), of which seven genes in *D. melanogaster* corresponding to Dmca1D, Dmca1A and Ca-α1T [265, 266] , ca-beta encoding a β subunit function of which were verified using relevant experiments [267], stj encoding a α2-δ subunit [268] and two unknown function genes (*CG4587* and *CG42817*), respectively.

The VDCC protein sequences were aligned using MAFFT with BLOSUM62 matrix and FFT-NS-I iterative refinement method, and then the poorly aligned regions and gaps were removed using trimAl with settings “gt=0.5” [110]. The unrooted maximum likelihood (ML) tree of VDCC genes was constructed using RAxML [33] with settings “--auto-prot=ml -f a -x 12345 -N 100 -p 12345 -m PROTGAMMAWAG” and visualized and modified using iTOL [41]. The tree showed that firefly VDCCs, together with those other insects, clustered with different a few clades (Figure S75). It is noted that no homology of Ate was found in Dmca1D clade.

S9.3.3 The cyclic nucleotide-gated ion channel

Cyclic nucleotide-gated ion channel (CNG) are nonselective cation channels that open, or gated, by direct binding of intracellular cyclic nucleotides (guanosine 3_,5_-cyclic monophosphate (cGMP), adenosine 3_,5_-cyclic monophosphate (cAMP) [269-272]. Aiming to explore the role of CNG in calcium signaling, we investigated CNG genes in genomes of beetles.

We collected 33 CNGs including also hyperpolarization-activated cyclic nucleotide-gated (HCN) channel genes of four species (human, rat, mouse and *D. melanogaster*) from UniProtKB (https://www.uniprot.org/uniprot) and FlyBase (http://flybase.org) to identify preliminarily CNGs using BLAST (E-value = 1e-10) with more than 30% identity in Coleoptera species (Lyu, Ate, Ala, Ppy, Ppe, Ilu, Apl, Agl, Tca, Dpo, Ota) and *B. mori*. These preliminary CNGs were scanned using Interproscan [273] to check if they possess cyclic nucleotide-binding domain (CNBD, IPR000595), rmlC-like jelly roll fold (IPR014710) and cyclic nucleotide-binding-like (IPR018490) simultaneously. In total, we identified 101 CNGs in 12 species (Data S32).

The amino acid sequences of CNGs including identified and known were aligned using MAFFT [78] with BLOSUM62 matrix and L-INS-i iterative refinement method and the spurious sequences and poorly aligned regions were filtered using trimAl (gt = 0.5) [110]. The phylogenetic tree was constructed using RAxML (--auto-prot=ml -f a -x 12345 -N 100 -p 12345 -m PROTGAMMAWAG) [33], and visualized and colored using iTOL [41]. Our tree showed that firefly CNGs, together with those of other insects, cluster with different clades (Figure S76).

S9.3.4 The store-operated channel

Store-operated calcium channel (SOC), i.e. Orai, is a major pathway for calcium signaling in virtually all metazoan cells. They are activated by the depletion of Ca^{2+} from the endoplasmic reticulum (ER), triggered physiologically through stimulation of a diverse set of surface receptors [274-276]. Here, we also investigate the SOC in the genomes of beetles.

We used cloned Orai genes from human (HumanOrai1: AAH75831, HumanOrai2: AAH69270, HumanOrai3: AAH06126), mouse (MouseOrai1: AAH23149, MouseOrai2: AAH66070, MouseOrai3: AAH61259) and rat (RatOrai1: AAH88225, RatOrai2: AAI58836, RatOrai3: AAH79355), also from *D. melanogaster* (DmeOrai1: *CG11430*) and *B. mori* (BmoOrai1: BAG69140) as query to identify homologs in insect species (Lyu, Ate, Ala, Ppy, Ppe, Ilu, Apl, Agl, Tca, Dpo, Ota, Bmo) using BLASTP (E-value =1e-10). Subsequently, the Orai-1 domain (PF07856) belonging to the mediator of CRAC channel activity was determined in these genes using HMMER 3.2 [75]. In total, the 22 Orai genes were identified as candidate genes (Data S32).

The amino acid sequences of 27 Orai genes including identified and known were aligned using MAFFT [78] with BLOSUM62 matrix and L-INS-i iterative refinement method. The phylogenetic tree was constructed using FastTree 2 with default settings [34] with those of human, rat and mouse as outgroups, and visualized and colored using iTOL [41]. Our tree showed that Orai1 and Orai2 were clustered independently in insect species and also were clustered specifically in fireflies (Figure S77).

S9.3.5 The transient receptor potential ion-channel

Transient receptor potential channels (TRP) are a group of ion channels located mostly on the plasma membrane of numerous animal cell types and function as intracellular Ca^{2+} release channels [277, 278]. The TRP family is subdivided into seven subfamilies containing TRPC (canonical), TRPV (vanilloid), TRPM (melastatin), TRPP (polycystin), TRPML (mucolipin), TRPA (ankyrin) and TRPN (NOMPC-like), of which the last is found only in invertebrates and fish. Here, we investigated the TRP genes in the beetles.

We collected 72 TRP genes from four species (*D. melanogaster*, *B. mori*, *T. castaneum* and *H. sapiens*) from UniProtKB (https://www.uniprot.org/uniprot) and FlyBase (http://flybase.org) to identify preliminary TRPs using BLASTP (E-value = 1e-5) against 11 beetles (six Elateroidea beetles (five fireflies: Lyu, Ate, Ala, Ppy, Ppe; and one luminous click beetle (Ilu)) and other six non-Elateroidea beetles (Ota, Dpo, Apl and Agl)), one silkworm and one fruit fly. The conserved domains (ion

transport protein: PF00520; and polycystin cation channel: PF08016) were scanned using HMMER 3.2 [75]. After removing redundancy, the best hit for TRP genes were retained. In total, we acquired 184 TRPs in 13 species (Data S32; Table S55).

The protein sequences were aligned using MUSCLE [30] and the poorly aligned regions and partial gaps were removed using trimAl (gt = 0.5) [110]. The phylogenetic tree was constructed using FastTree 2 [34], and displayed and colored using iTOL [41]. Our tree showed that all firefly TRP genes were perfectly clustered into 7 clades corresponding to 7 TRP subfamilies (Figure S78).

S9.3.6 Voltage-dependent anion channel

Voltage-dependent anion channel (VDAC) is traditionally considered as a large, high-conductance, weakly anion-selective channel, fully opened at low potential (<20-30 mV), but switching to cation selectivity and lower conductance at higher potentials [207]. Ca^{2+} import across the outer mitochondrial membrane (OMM) occurs through VDAC, the most abundant protein of the outer mitochondrial membrane [207].

70 known VDAC protein sequences including insects and *H. sapiens* were downloaded from UniProt database (https://www.uniprot.org) and used to BLASTP (E-value = 10^{-5}) against the genomes of 11 beetles (six Elateroidea beetles (five fireflies: Lyu, Ate, Ala, Ppy, Ppe; and one luminous click beetle (Ilu)) and other six non-Elateroidea beetles (Ota, Dpo, Apl and Agl)) and one silkworm. Then the eukaryotic porin domains (PF01459) was scanned using HMMER 3.2 [75]. After removing redundancy, the best hit for VDAC genes were retained. In total, we acquired 18 VDACs in 13 species (Data S32).

The amino acid sequences were aligned using MUSCLE 3.8 [30] and the poorly aligned regions and partial gaps were removed with trimAl (gt=0.5) [110]. The phylogenetic tree were constructed using RAxML with maximum likelihood method [33] and displayed and edited using iTOL [41] (Figure S79).

S9.4 Calcium pumps and exchangers

S9.4.1 Calcium ATPase and P-type ATPase

Calcium ATPase belongs to P-type ATPase family, which establish and maintain steep electrochemical gradients of key cations across membranes and are therefore vital to all eukaryotes and most prokaryotes [279]. P-type ATPase family can be divided

into ten different subtypes or classes in five branches (Types I-V) [280, 281]. The genomes of *D. melanogaster* and *Caenorhabditis elegans* contain 21 and 15 genes, respectively, both in branches I, II, IV and V [282]. In order to explore the possible contribution of calcium ATPase to flash control, we identified all P-type ATPase in firefly genomes.

Full-length established P-type ATPase sequences were retrieved from the Transporter Classification Database (TCDB) [283] and used to screen recognized proteins encoded in the genomes of 11 beetles (six Elateroidea beetles (five fireflies: Lyu, Ate, Ala, Ppy, Ppe; and one luminous click beetle (Ilu)) and other six non-Elateroidea beetles (Ota, Dpo, Apl and Agl)) and one silkworm. Because of the high sequence similarity among all functional P-type ATPases, this could usually be achieved with a single BLAST search without iterations. In order to ensure a complete set of sequences, the P-type ATPase database (http://traplabs.dk/patbase/) was also used as a resource. Next, the amino acid sequence of candidate genes was checked for presence of characteristic P-type ATPases domains via Pfam (http://pfam.xfam.org/). The motif of "DKTG" was scanned. After eliminating redundant and truncating sequences, 194 proteins were obtained for further analyses (Data S32; Table S56).

The protein sequences were aligned using MUSCLE [30] and then the poorly aligned regions and partial gaps were removed with trimAl (gt=0.5) [110]. The phylogenetic tree was constructed using FastTree 2 with maximum likelihood method [34], and displayed and edited using iTOL [41]. Our tree showed that all identified P-type ATPase genes were clustered into six subfamilies: P1B (Cu^{+}, Ag^{+}, Cu^{2+}, Cd^{2+}, Zn^{2+}, Pb^{2+}, Co^{2+}), P2A (Ca^{2+}, Mn^{2+}; including SERCA pumps), P2B (Ca^{2+}; including plasma membrane Ca^{2+} ATPase (PMCA) pumps), P2C (Na^{+}/K^{+}; H^{+}/K^{+}), P4 (phospholipids ?), and P5 (no assigned specifity), similar to the TCDB (Figure S80).

S9.4.2 Sodium-calcium exchanger

The Na^{+}-Ca^{2+} (NCX) exchanger is an electrogenic ion transporter that functions for the efflux of one Ca^{2+} in exchange for three Na^{+} [284]. The NCX is considered one of the most important cellular mechanisms for removing Ca^{2+} [285] and is usually found in the plasma membranes and the mitochondria and endoplasmic reticulum of excitable cells [286, 287]. In animals, Na^{+}/Ca^{2+} exchangers are divided into three groups based on stoichiometry: Na^{+}/Ca^{2+} exchangers (NCX), $Na^{+}/Ca^{2+}/K^{+}$ exchangers (NCKX), and Ca^{2+}/Cation exchangers (CCX/NCLX). In mammals, there are three NCX genes, five NCKX genes and one NCLX gene [288].

28 known Na^+/Ca^{2+} exchanger protein sequences (NCX, NCKX, and NCLX) from the UniProt database (https://www.uniprot.org/) including *H. sapiens*, *C. elegans*, *D. melanogaster* and *T. castaneum* were used to BLASTP (E-value = 1e-5) against the genomes of 11 beetles (six Elateroidea beetles (five fireflies: Lyu, Ate, Ala, Ppy, Ppe; and one luminous click beetle (Ilu)) and other six non-Elateroidea beetles (Ota, Dpo, Apl and Agl)) and one silkworm. Specific structural signatures unique to each sodium calcium exchanger subtype were used to phase matches, which were filtered through InterProscan [289] and organized into be one of NCX, NCKX and NCLX subtypes [288]. In total, we acquired 79 NCX genes, while no genes were found in the subtype of NCLX in insect species (Data S32; Table S57).

The amino acid sequences were aligned using MUSCLE [30] and the poorly aligned regions and partial gaps were removed with trimAl (gt=0.5) [110]. The phylogenetic tree was constructed using RAxML with maximum likelihood method [33], and displayed and edited using iTOL [41]. Our tree indicated that the NCX subtype was well conserved that except *P. pectoralis* and *A. lateralis* (two NCX genes), each firefly species had one NCX gene (Data S32; Figure S81).

S9.4.3 Calcium uniporter

Mitochondrial calcium plays critical roles in diverse cellular process ranging from energy metabolism to cell death. The calcium uptake into mitochondria is mediated by mitochondrial inner membrane calcium uniporter [290, 291], which is finely tuned by Mitochondrial Calcium Uptake 1 (MICU1) and Mitochondrial Calcium Uptake 2 (MICU2) [292, 293]. In *Drosophila*, mitochondrial calcium uniporter (MCU) is responsible for endoplasmic reticulum-to-mitochondrial calcium transfer [294]. The mitochondrial calcium uniporter (UP) complex includes the channel-forming subunit MCU and its regulators MICU1, MICU2, MCUb (Mitochondrial Calcium Uniporter b), EMRE (Essential MCU REgulator) and MCUR1 (Mitochondrial Calcium Uniporter Regulator 1).

The protein sequence of 408 known UP protein sequences from the UniProt database (https://www.uniprot.org/) were used to BLASTP against the genomes of 11 beetles (six Elateroidea beetles (five fireflies: Lyu, Ate, Ala, Ppy, Ppe; and one luminous click beetle (Ilu)) and other six non-Elateroidea beetles (Ota, Dpo, Apl and Agl)) and one silkworm. The candidate genes were further identified with conservative domains using Pfam. After removing redundancy, putative UP genes were retained. In total, we identified 49 UP genes in 13 species (Data S32; Table S58).

Protein sequences were aliged using MUSCLE [30] and the poor aligned regions and partial gaps were removed with trimAl (gt=0.5) [110]. Phylogenetic tree were constructed using RAxML with a maximum likelihood method [33], and displayed and edited using iTOL [41]. Our tree indicated that only MCU and its regulators MICU1 and MICU2 existed in fireflies (Figure S82).

S9.5 Calcium buffer: Calreticulin

We found identified a calreticulin-like protein positively selected in the ancestor of luminous beetles. The endoplasmic reticulum (ER) is one of the largest membrane organelles in eukaryotic cells and is also an important site of Ca^{2+} storage and release. Calreticulin (Crc) is a unique ER luminal resident protein and performs two major functions: chaperoning and regulation of Ca^{2+} homeoostasis [295]. Aiming to explore the role of calreticulin in Ca^{2+} signaling and flash control, we investigated this protein in the genomes of beetles.

Four insect Crc genes (*Halyomorpha halys*: HhaCrc, XP_014277576; *Nilaparvata lugens*: NluCrc, ANJ04674; *Pteromalus puparum*: PupCrc, ACZ68113; *Pieris rapae*: PraCrc, ACJ07154) were used to BLASTP against the genomes of 11 beetles (six Elateroidea beetles (five fireflies: Lyu, Ate, Ala, Ppy, Ppe; and one luminous click beetle (Ilu)) and other six non-Elateroidea beetles (Ota, Dpo, Apl and Agl)) and one silkworm. The 14 Crc genes with more than 50% identity were identified (Data S32).

These protein sequences of Crc genes were aligned using MAFFT [78] with BLOSUM6 matrix. Subsequently, the phylogenetic tree was constructed by the maximum likelihood (ML) method using the RAxML [33] with settings "--auto-prot=ml -f a -x 12345 -N 100 -p 12345 -m PROTGAMMAWAG" with NluCrc as an outgroup, and visualized in iTOL [41] (Figure S83).

S9.6 Calcium effectors: Calmodulin/Nitric oxide synthase

S9.6.1 Calmodulin

Calmodulin (CAM) serves as a Ca^{2+} effector to control a variety of physiological, cellular and molecular processes such as contraction of smooth muscle, cross-talk between signaling pathways, gene transcription, ion channel modulation and metabolism [296]. Calcium binding CAM can activate nitric oxide synthase (NOS) to produce NO [297, 298].

In *D. melanogaster* genome, a single gene (DmeCAM, *CG8472*) encoding CAM was discovered and cloned [299]. So, we used DmeCAM to identified CAM genes using BLASTP [49] (E-value = 1e-20) in fireflies and other insects. Due to high conservation among protein sequences, we chose more than 70% identity and 80% coverage as CAM candidate genes. Moreover, the CAM genes were searched to belong to the EF-hand superfamily using hmmscan in HMMER 3.2 [75]. In total, 20 CAM genes were identified in 12 species (Data S32).

The protein sequences were aligned using MAFFT [78] with BLOSUM62 matrix and L-INS-I iterative refinement method, and unaligned regions were trimmed using trimA1 [110] (gt=0.5). The phylogenetic tree was constructed using RAxML [33] with settings "--auto-prot=ml -f a -x 12345 -N 100 -p 12345 -m PROTGAMMAWAG" , with DmeCAM as the outgroup. The tree was visualized and modified using iTOL [41] (Figure S84).

S9.6.2 Nitric oxide synthase genes

Nitric Oxide (NO) was the first discovered gaseous signaling molecule and exerted physiological functions in the nervous and immune systems, contributing to the regulation of behavior, gastrointestinal motility, and defense mechanisms against infectious disease and tumors [298]. It is mainly produced from L-arginine by nitric oxide synthase (NOS), though other mechanisms of NO production exist [300, 301].

Six cloned NOS genes from two fireflies (Coleoptera, Lampyridae: *A. lateralis* (BAF63160), *L. cruciata* (BAF63161) [302, 303]) and other four insects (Lepidoptera, Bombycidae: *B. mori* (NP_001036963); Hymenoptera, Pteromalidae, *Nasonia vitripennis* (NP_001161704), Apidae, *Apis mellifera* (BAD89803); Diptera: *D. melanogaste*r (AAC46882)) [304-306] were used to BLATP [49] against the genomes of the 11 beetles (six Elateroidea beetles (five fireflies: Lyu, Ate, Ala, Ppy, Ppe; and one luminous click beetle (Ilu)) and other six non-Elateroidea beetles (Ota, Dpo, Apl and Agl)), other five non-coleoptera insects (Lepidoptera: Pxu, Pma; Phthiraptera: Pnu; Hemipera: Api; Isoptera: Zne) and one crustacea (Cladocera: Dpu). These NOS genes were selected with more than 50% identity and 80% coverage. In addition, selected NOS genes were further scanned using HMMER 3.2 [75] to search PF02898 (nitric oxide synthase oxygenase domain).

The protein sequences of NOS genes of 23 NOS genes identified here and previously known from 22 species were aligned using MAFFT [78] with BLOSUM6 matrix and removed poorly aligned regions using trimA1 (gt=0.5) [110]. The phylogenetic tree was

constructed by the maximum likelihood (ML) method using the RAxML [33] with DpuNOS as an outgroup , and visualized in iTOL [41]. Our tree showed that all firefly NOS form a clade (Figure S85).

In addition, we discovered a specific amino acid site (277Q) at PF02898 (nitric oxide synthase oxygenase domain) in flash firefly species by aligning these NOS genes against all NOS genes of NR database (Data S33; Figure S86). Due to the limitation of flash species, we will need to further determine the specific site in virtue of more flash species in the future.

S9.7 The expression of the calcium signaling system

We analyzed the expression of genes of the calcium signaling system at transcriptomic and proteomic levels in luminous organs of *L. yunnana* and *A. terminalis* (Figures S87-S89). The expressed signals were captured in all of above genes at transcriptomic level in both *L. yunnana* and *A. terminalis*. Specially, VDAC (channel), P-type ATPase (pump) (P2B (PMCA) and P2C (Na^+/K^+; H^+/K^+)) and calreticulin (buffer) showed higher expression than other genes. In addition, they showed also high abundance at proteomic level. So, we speculate that these genes may play important role in the calcium balance in luminous insects. Besides, other genes had signals at proteomic level, including Gα and PKA (transducer), TRP (channel) and SERCA (P2A). Interestingly, the expression of Gs, VDAC, P2B and P2C in *A. terminalis* was higher than *L. yunnana* at proteomic level (Data S16), which might be major genes regulating flash control. Thus, we speculate that flash control difference between *L. yunnana* and *A. terminalis* might be induced by modulating the calcium ion concentration which has important role in the synthesis of NO in tracheolar end cells induced by octopamine.

References

1. Jeng, M.L., Lai, J., Yang, P.S. & Sato, M. Notes on the Taxonomy of Lamprigera yunnana (Fairmaire) and the Genus Lamprigera Motschulsky (Coleoptera, Lampyridae). *Japanese Journal of Systematic Entomology* **6**, 7 (2000).
2. Fairmaire, L.M.H. Description de Coléoptères nouveaux de la Malaisie, de l’Inde et de la Chine. . *Notes Leyden Mus.* **19**, 25 (1897).
3. Ballantyne, L. et al. Studies on South-east Asian fireflies: Abscondita, a new genus with details of life history, flashing patterns and behaviour of Abs.

chinensis (L.) and Abs. terminalis (Olivier) (Coleoptera: Lampyridae: Luciolinae). *Zootaxa* **3721**, 1-48 (2013).

4. McDermott, F.A. in Coleopterorum Catalogus Supplementa (9) (editio secunda). (ed. W.O. Steel) 149 (W. Junk,'s-gravenhage, Nethelands; 1966).
5. McDermot, F.A. The taxonomy of the Lampyridae (Coleoptera). *Trans. Amer. Ent. Soc.* **90**, 70 (1964).
6. Li, X.Y., Yang, S., Xie, M. & Liang, X.C. Phylogeny of fireflies (Coleoptera : Lampyridae) inferred from mitochondrial 16S ribosomal DNA, with references to morphological and ethological traits. *Prog Nat Sci* **16**, 817-826 (2006).
7. Martin, G.J., Branham, M.A., Whiting, M.F. & Bybee, S.M. Total evidence phylogeny and the evolution of adult bioluminescence in fireflies (Coleoptera: Lampyridae). *Mol Phylogenet Evol* **107**, 564-575 (2017).
8. Chin, C.S. et al. Phased diploid genome assembly with single-molecule real-time sequencing. *Nat Methods* **13**, 1050-+ (2016).
9. Li, H. Minimap and miniasm: fast mapping and de novo assembly for noisy long sequences. *Bioinformatics* **32**, 2103-2110 (2016).
10. Li, H. & Durbin, R. Fast and accurate short read alignment with Burrows-Wheeler transform. *Bioinformatics* **25**, 1754-1760 (2009).
11. Li, H. et al. The Sequence Alignment/Map format and SAMtools. *Bioinformatics* **25**, 2078-2079 (2009).
12. Simao, F.A., Waterhouse, R.M., Ioannidis, P., Kriventseva, E.V. & Zdobnov, E.M. BUSCO: assessing genome assembly and annotation completeness with single-copy orthologs. *Bioinformatics* **31**, 3210-3212 (2015).
13. Fu, X. et al. Long-read sequence assembly of the firefly Pyrocoelia pectoralis genome. *Gigascience* **6**, 1-7 (2017).
14. Fallon, T.R. et al. Firefly genomes illuminate parallel origins of bioluminescence in beetles. *Elife* **7** (2018).
15. Kim, D. et al. TopHat2: accurate alignment of transcriptomes in the presence of insertions, deletions and gene fusions. *Genome Biology* **14** (2013).
16. Bao, W.D., Kojima, K.K. & Kohany, O. Repbase Update, a database of repetitive elements in eukaryotic genomes. *Mobile DNA-Uk* **6** (2015).
17. M, T.-G. & N, C. Using RepeatMasker to identify repetitive elements in genomic sequences. *Curr Protoc Bioinformatics* **3**, 4–14 (2009).
18. Benson, G. Tandem repeats finder: a program to analyze DNA sequences. *Nucleic Acids Research* **27**, 573-580 (1999).
19. Fallon, T.R. et al. Firefly genomes illuminate parallel origins of bioluminescence in beetles. *Elife* **7** (2018).
20. Fu, X.H. et al. Long-read sequence assembly of the firefly Pyrocoelia pectoralis genome. *Gigascience* **6** (2017).
21. Haas, B.J. et al. Automated eukaryotic gene structure annotation using EVidenceModeler and the program to assemble spliced alignments. *Genome Biology* **9** (2008).

22. Haas, B.J. et al. Improving the Arabidopsis genome annotation using maximal transcript alignment assemblies. *Nucleic Acids Research* **31**, 5654-5666 (2003).
23. Hoff, K.J. & Stanke, M. WebAUGUSTUS-a web service for training AUGUSTUS and predicting genes in eukaryotes. *Nucleic Acids Research* **41**, W123-W128 (2013).
24. Trapnell, C. et al. Differential gene and transcript expression analysis of RNA-seq experiments with TopHat and Cufflinks (vol 7, pg 562, 2012). *Nat Protoc* **9**, 2513-2513 (2014).
25. Tang, S.Y.Y., Lomsadze, A. & Borodovsky, M. Identification of protein coding regions in RNA transcripts. *Nucleic Acids Research* **43** (2015).
26. Kent, W.J. BLAT - The BLAST-like alignment tool. *Genome Res* **12**, 656-664 (2002).
27. Gertz, E.M., Yu, Y.K., Agarwala, R., Schaffer, A.A. & Altschul, S.F. Composition-based statistics and translated nucleotide searches: Improving the TBLASTN module of BLAST. *Bmc Biol* **4** (2006).
28. Birney, E. & Durbin, R. Using GeneWise in the Drosophila annotation experiment. *Genome Res* **10**, 547-548 (2000).
29. Li, L., Stoeckert, C.J., Jr. & Roos, D.S. OrthoMCL: identification of ortholog groups for eukaryotic genomes. *Genome Res* **13**, 2178-2189 (2003).
30. Edgar, R.C. MUSCLE: multiple sequence alignment with high accuracy and high throughput. *Nucleic Acids Res* **32**, 1792-1797 (2004).
31. Suyama, M., Torrents, D. & Bork, P. PAL2NAL: robust conversion of protein sequence alignments into the corresponding codon alignments. *Nucleic Acids Res* **34**, W609-612 (2006).
32. Castresana, J. Selection of conserved blocks from multiple alignments for their use in phylogenetic analysis. *Molecular Biology and Evolution* **17**, 540-552 (2000).
33. Stamatakis, A. RAxML version 8: a tool for phylogenetic analysis and post-analysis of large phylogenies. *Bioinformatics* **30**, 1312-1313 (2014).
34. Price, M.N., Dehal, P.S. & Arkin, A.P. FastTree 2-Approximately Maximum-Likelihood Trees for Large Alignments. *Plos One* **5** (2010).
35. Yang, Z. PAML 4: phylogenetic analysis by maximum likelihood. *Mol Biol Evol* **24**, 1586-1591 (2007).
36. Rambaut, A., Drummond, A.J., Xie, D., Baele, G. & Suchard, M.A. Posterior Summarization in Bayesian Phylogenetics Using Tracer 1.7. *Syst Biol* **67**, 901-904 (2018).
37. Kumar, S., Stecher, G., Li, M., Knyaz, C. & Tamura, K. MEGA X: Molecular Evolutionary Genetics Analysis across Computing Platforms. *Molecular Biology and Evolution* **35**, 1547-1549 (2018).
38. Vaidya, G., Lohman, D.J. & Meier, R. SequenceMatrix: concatenation software for the fast assembly of multi-gene datasets with character set and codon information. *Cladistics* **27**, 171-180 (2011).

39. Lanfear, R., Frandsen, P.B., Wright, A.M., Senfeld, T. & Calcott, B. PartitionFinder 2: New Methods for Selecting Partitioned Models of Evolution for Molecular and Morphological Phylogenetic Analyses. *Molecular Biology and Evolution* **34**, 772-773 (2017).
40. John P. Huelsenbeck & Ronquist, F. MRBAYES: Bayesian inference of phylogenetic trees. *Bioinformatics* **17**, 754-755 (2001).
41. Letunic, I. & Bork, P. Interactive tree of life (iTOL) v3: an online tool for the display and annotation of phylogenetic and other trees. *Nucleic Acids Research* **44**, W242–W245 (2016).
42. Misof, B. et al. Phylogenomics resolves the timing and pattern of insect evolution. *Science* **346**, 763-767 (2014).
43. Mckenna, D.D. et al. The beetle tree of life reveals that Coleoptera survived end-Permian mass extinction to diversify during the Cretaceous terrestrial revolution. *Syst Entomol* **40**, 835-880 (2015).
44. De Bie, T., Cristianini, N., Demuth, J.P. & Hahn, M.W. CAFE: a computational tool for the study of gene family evolution. *Bioinformatics* **22**, 1269-1271 (2006).
45. Cosentino, S. & Iwasaki, W. SonicParanoid: fast, accurate and easy orthology inference. *Bioinformatics* **35**, 149-151 (2019).
46. Loytynoja, A. & Goldman, N. An algorithm for progressive multiple alignment of sequences with insertions. *Proc Natl Acad Sci U S A* **102**, 10557-10562 (2005).
47. Storey, J.D. & Tibshirani, R. Statistical significance for genomewide studies. *Proc Natl Acad Sci U S A* **100**, 9440-9445 (2003).
48. Zhang, J.Z., Nielsen, R. & Yang, Z.H. Evaluation of an improved branch-site likelihood method for detecting positive selection at the molecular level. *Mol Biol Evol* **22**, 2472-2479 (2005).
49. Ye, J., McGinnis, S. & Madden, T.L. BLAST: improvements for better sequence analysis. *Nucleic Acids Res* **34**, W6-9 (2006).
50. Patel, R.K. & Jain, M. NGS QC Toolkit: A Toolkit for Quality Control of Next Generation Sequencing Data. *Plos One* **7** (2012).
51. Chang, Z. et al. Bridger: a new framework for de novo transcriptome assembly using RNA-seq data. *Genome Biology* **16** (2015).
52. Fu, L.M., Niu, B.F., Zhu, Z.W., Wu, S.T. & Li, W.Z. CD-HIT: accelerated for clustering the next-generation sequencing data. *Bioinformatics* **28**, 3150-3152 (2012).
53. Kim, D. et al. TopHat2: accurate alignment of transcriptomes in the presence of insertions, deletions and gene fusions. *Genome Biol* **14**, R36 (2013).
54. Anders, S., Pyl, P.T. & Huber, W. HTSeq--a Python framework to work with high-throughput sequencing data. *BIOINFORMATICS* **31**, 166–169 (2015).
55. Robinson, M.D., McCarthy, D.J. & Smyth, G.K. edgeR: a Bioconductor package for differential expression analysis of digital gene expression data. *Bioinformatics* **26**, 139-140 (2010).

56. Schilling, B. et al. Platform-independent and Label-free Quantitation of Proteomic Data Using MS1 Extracted Ion Chromatograms in Skyline APPLICATION TO PROTEIN ACETYLATION AND PHOSPHORYLATION. *Mol Cell Proteomics* **11**, 202-214 (2012).
57. Smith, S.J., Kroon, J.T.M., Simon, W.J., Slabas, A.R. & Chivasa, S. A Novel Function for Arabidopsis CYCLASE1 in Programmed Cell Death Revealed by Isobaric Tags for Relative and Absolute Quantitation (iTRAQ) Analysis of Extracellular Matrix Proteins. *Mol Cell Proteomics* **14**, 1556-1568 (2015).
58. Day, J.C., Tisi, L.C. & Bailey, M.J. Evolution of beetle bioluminescence: the origin of beetle luciferin. *Luminescence* **19**, 8-20 (2004).
59. Seliger, H.H. in Chemiluminescence and Bioluminescence. (eds. M.J. Cormier, D.M. Hercules & J. Lee) 461-478 (Plenum:, New York; 1973).
60. White, E.H., McCapra, F. & Field, G.F. The Structure and Synthesis of Firefly Luciferin. *J. Am. Chem. Soc.* **85(3)337-343** (1963).
61. White, E.H., Field, G.F., Mcelroy, W.D. & Mccapra, F. Structure and Synthesis of Firefly Luciferin. *J Am Chem Soc* **83**, 2402-& (1961).
62. Colepicoloneto, P., Costa, C. & Bechara, E.J.H. Brazilian Species of Luminescent Elateridae - Luciferin Identification and Bioluminescence Spectra. *Insect Biochem* **16**, 803-810 (1986).
63. HadjMohammadi, M.R. & Chaichi, M.J. Separation, identification and determination of luciferin in the Iranian firefly, Lampyris turkestanicus by HPLC and spectroscopic methods. *Photochem Photobiol* **64**, 821-822 (1996).
64. Seliger, H.H. & McELroy, W., D. Light Physical and Biological Action. (Academic Press, New York; 1965).
65. Niwa, K., Nakamura, M. & Ohmiya, Y. Stereoisomeric bio-inversion key to biosynthesis of firefly D-luciferin. *Febs Lett* **580**, 5283-5287 (2006).
66. Oba, Y., Yoshida, N., Kanie, S. & Inouye, S. Biosynthesis of Firefly Luciferin in Adult Lantern: Decarboxylation of L-Cysteine is a Key Step for Benzothiazole Ring Formation in Firefly Luciferin Synthesis (vol 8, e84023, 2013). *Plos One* **9** (2014).
67. Hemmati, R. et al. Luciferin-Regenerating Enzyme Mediates Firefly Luciferase Activation Through Direct Effects of D-Cysteine on Luciferase Structure and Activity. *Photochem Photobiol* **91**, 828-836 (2015).
68. Kanie, S., Nishikawa, T., Ojika, M. & Oba, Y. One-pot non-enzymatic formation of firefly luciferin in a neutral buffer from p-benzoquinone and cysteine. *Sci Rep-Uk* **6** (2016).
69. Vongsangnak, W., Chumnanpuen, P. & Sriboonlert, A. Transcriptome analysis reveals candidate genes involved in luciferin metabolism in Luciola aquatilis (Coleoptera: Lampyridae). *Peerj* **4** (2016).
70. Fallon, T.R., Li, F.S., Vicent, M.A. & Weng, J.K. Sulfoluciferin is Biosynthesized by a Specialized Luciferin Sulfotransferase in Fireflies. *Biochemistry* **55**, 3341-3344 (2016).
71. Viviani, V.R. The origin, diversity, and structure function relationships of insect luciferases. *Cell Mol Life Sci* **59**, 1833-1850 (2002).

72. Watkins, P.A. & Ellis, J.M. Peroxisomal acyl-CoA synthetases. *Bba-Mol Basis Dis* **1822**, 1411-1420 (2012).
73. Khurana, P., Gokhale, R.S. & Mohanty, D. Genome scale prediction of substrate specificity for acyl adenylate superfamily of enzymes based on active site residue profiles. *Bmc Bioinformatics* **11**, 57 (2010).
74. Stuible, H.P. & Kombrink, E. Identification of the substrate specificity-conferring amino acid residues of 4-coumarate:coenzyme A ligase allows the rational design of mutant enzymes with new catalytic properties. *J Biol Chem* **276**, 26893-26897 (2001).
75. Finn, R.D. et al. HMMER web server: 2015 update. *Nucleic Acids Res* **43**, W30-38 (2015).
76. Oba, Y., Iida, K. & Inouye, S. Functional conversion of fatty acyl-CoA synthetase to firefly luciferase by site-directed mutagenesis: A key substitution responsible for luminescence activity. *Febs Lett* **583**, 2004-2008 (2009).
77. Viviani, V.R., Prado, R.A., Arnoldi, F.C.G. & Abdalla, F.C. An ancestral luciferase in the Malpighi tubules of a non-bioluminescent beetle! *Photoch Photobio Sci* **8**, 57-61 (2009).
78. Katoh, K. & Standley, D.M. MAFFT multiple sequence alignment software version 7: improvements in performance and usability. *Mol Biol Evol* **30**, 772-780 (2013).
79. Bouckaert, R. et al. BEAST 2: A Software Platform for Bayesian Evolutionary Analysis. *Plos Comput Biol* **10** (2014).
80. Hu, B. et al. GSDS 2.0: an upgraded gene feature visualization server. *Bioinformatics* **31**, 1296-1297 (2015).
81. Niwa, K. et al. Biosynthesis of firefly D-luciferin. *Luminescence* **21**, 286-286 (2006).
82. Oba, Y., Yoshida, N., Kanie, S., Ojika, M. & Inouye, S. Biosynthesis of Firefly Luciferin in Adult Lantern: Decarboxylation of L-Cysteine Is a Key Step for Benzothiazole Ring Formation in Firefly Luciferin Synthesis. *Plos One* **8** (2013).
83. Brosnan, J.T. & Brosnan, M.E. The sulfur-containing amino acids: An overview. *J Nutr* **136**, 1636s-1640s (2006).
84. Liu, M., Barnes, V.L. & Pile, L.A. Disruption of Methionine Metabolism in Drosophila melanogaster Impacts Histone Methylation and Results in Loss of Viability. *G3 (Bethesda)* **6**, 121-132 (2015).
85. Messerschmidt, A. et al. Determinants of enzymatic specificity in the Cys-Met-metabolism PLP-dependent enzyme family: Crystal structure of cystathionine gamma-lyase from yeast and intrafamiliar structure comparison. *Biol Chem* **384**, 373-386 (2003).
86. Zhao, X.L., Li, Q., Meng, Q., Yue, C.Y. & Xu, C.X. Identification and expression of cysteine sulfinate decarboxylase, possible regulation of taurine biosynthesis in Crassostrea gigas in response to low salinity. *Sci Rep-Uk* **7** (2017).

87. Suetsugu, Y. et al. Large scale full-length cDNA sequencing reveals a unique genomic landscape in a lepidopteran model insect, Bombyx mori. *G3 (Bethesda)* **3**, 1481-1492 (2013).
88. Okada, K., Iio, H. & Goto, T. Biosynthesis of Firefly Luciferin - Probable Formation of Benzothiazole from Para-Benzoquinone and Cysteine. *J Chem Soc Chem Comm*, 32-32 (1976).
89. Day, J.C., Goodall, T.I. & Bailey, M.J. The evolution of the adenylate-forming protein family in beetles: multiple luciferase gene paralogues in fireflies and glow-worms. *Mol Phylogenet Evol* **50**, 93-101 (2009).
90. Oba, Y., Iida, K. & Inouye, S. Functional conversion of fatty acyl-CoA synthetase to firefly luciferase by site-directed mutagenesis: a key substitution responsible for luminescence activity. *FEBS Lett* **583**, 2004-2008 (2009).
91. Okada, K., Iio, H. & Goto, T. Biosynthesis of firefly luciferin. Probable formation of benzothiazole from p-benzoquinone and cysteine., (1), 32. doi:10.1039/c39760000032 *Journal of the Chemical Society, Chemical Communications* **(1), 32** (1976).
92. Meinwald, J., Koch, K.F., Rogers, J.E. & Eisner, T. Biosynthesis of Arthropod Secretions .3. Synthesis of Simple P-Benzoquinones in a Beetle (Eleodes Longicollis). *J Am Chem Soc* **88**, 1590-& (1966).
93. Sterkel, M. & Oliveira, P.L. Developmental roles of tyrosine metabolism enzymes in the blood-sucking insect Rhodnius prolixus. *P Roy Soc B-Biol Sci* **284** (2017).
94. Sterkel, M. et al. Tyrosine Detoxification Is an Essential Trait in the Life History of Blood-Feeding Arthropods. *Curr Biol* **26**, 2188-2193 (2016).
95. Moran, G.R. 4-Hydroxyphenylpyruvate dioxygenase. *Archives of Biochemistry and Biophysics* **433**, 117-128 (2005).
96. Fernandez-Canon, J.M. et al. Maleylacetoacetate isomerase (MAAI/GSTZ)-deficient mice reveal a glutathione-dependent nonenzymatic bypass in tyrosine catabolism. *Molecular and Cellular Biology* **22**, 4943-4951 (2002).
97. Sterkel, M. & Oliveira, P.L. Developmental roles of tyrosine metabolism enzymes in the blood-sucking insect Rhodnius prolixus. *Proc Biol Sci* **284** (2017).
98. Gray, E.E., Small, S.N. & McGuirl, M.A. Expression and characterization of recombinant tyramine beta-monooxygenase from Drosophila: a monomeric copper-containing hydroxylase. *Protein Expr Purif* **47**, 162-170 (2006).
99. Wallace, B.G. The biosynthesis of octopamine-characterization of lobster tyramine beta-hydroxylase. *Neurochem* **26**, 761–770 (1976).
100. Goldstein, M. & Contrera, J.F. The substrate specificity of phenylamine-beta-hydroxylase. *J Biol Chem* **237**, 1898-1902 (1962).
101. Noh, M.Y., Muthukrishnan, S., Kramer, K.J. & Arakane, Y. Cuticle formation and pigmentation in beetles. *Curr Opin Insect Sci* **17**, 1-9 (2016).
102. Vavricka, C.J. et al. Tyrosine metabolic enzymes from insects and mammals: a comparative perspective. *Insect Sci* **21**, 13-19 (2014).

103. Altschul, S.F. et al. Gapped BLAST and PSI-BLAST: a new generation of protein database search programs. *Nucleic Acids Research* **25**, 3389-3402 (1997).
104. Han, Q., Robinson, H., Ding, H., Christensen, B.M. & Li, J. Evolution of insect arylalkylamine N-acetyltransferases: structural evidence from the yellow fever mosquito, Aedes aegypti. *Proc Natl Acad Sci U S A* **109**, 11669-11674 (2012).
105. Reinhard, J. et al. Hydroquinone: A general phagostimulating pheromone in termites. *J Chem Ecol* **28**, 1-14 (2002).
106. Bujang, N.S., Harrison, N.A. & Su, N.-Y. Molecular Cloning of Five β-Glucosidases From Four Species of Higher Termites (Blattodea: Termitidae). *Annals of the Entomological Society of America* **107**, 251-256 (2014).
107. Byeon, G.M. et al. A digestive beta-glucosidase from the silkworm, Bombyx mori: cDNA cloning, expression and enzymatic characterization. *Comp Biochem Physiol B Biochem Mol Biol* **141**, 418-427 (2005).
108. Singh, G., Verma, A.K. & Kumar, V. Catalytic properties, functional attributes and industrial applications of beta-glucosidases. *3 Biotech* **6**, 3 (2016).
109. Vongsangnak, W., Chumnanpuen, P. & Sriboonlert, A. Transcriptome analysis reveals candidate genes involved in luciferin metabolism in Luciola aquatilis (Coleoptera: Lampyridae). *PeerJ* **4**, e2534 (2016).
110. Capella-Gutierrez, S., Silla-Martinez, J.M. & Gabaldon, T. trimAl: a tool for automated alignment trimming in large-scale phylogenetic analyses. *Bioinformatics* **25**, 1972-1973 (2009).
111. Blum, M.S. Biosynthesis of Arthropod Exocrine Compounds. *Annual Review of Entomology* **32**, 381-413 (1987).
112. Rocha, D.F.O. et al. Harvestman Phenols and Benzoquinones: Characterisation and Biosynthetic Pathway. *Molecules* **18**, 11429-11451 (2013).
113. Roth, L.M. & Stay, B. The Occurrence of Para-Quinones in Some Arthropods, with Emphasis on the Quinone-Secreting Tracheal Glands of Diploptera-Punctata (Blattaria). *J Insect Physiol* **1**, 305-& (1958).
114. Aneshansley, D.J., Eisner, T., Widom, J.M. & Widom, B. Biochemistry at 100 Degrees C - Explosive Secretory Discharge of Bombardier Beetles (Brachinus). *Science* **165**, 61-+ (1969).
115. Weathers.J & Percy, J.E. Studies of Physiologically Active Arthropod Secretions .3. Chemical, Morphological, and Histological Studies of Defence Mechanism of Uroblaniulus Canadensis (Say) (Diplopoda - Julida). *Can J Zoolog* **47**, 1389-& (1969).
116. Conner, W.E., Alley, K.M., Barry, J.R. & Harper, A.E. Has vertebrate chemesthesis been a selective agent in the evolution of arthropod chemical defenses? *Biol Bull* **213**, 267-273 (2007).

117. Happ, G.M. Quinone and Hydrocarbon Production in Defensive Glands of Eleodes Longicollis and Tribolium Castaneum (Coleoptera Tenebrionidae). *J Insect Physiol* **14**, 1821-+ (1968).
118. Li, J.W. et al. Odoriferous Defensive Stink Gland Transcriptome to Identify Novel Genes Necessary for Quinone Synthesis in the Red Flour Beetle, Tribolium castaneum. *Plos Genet* **9** (2013).
119. Taylor, A.M., Kammath, V. & Bleakley, A. Tyrosinase, could it be a missing link in ochronosis in alkaptonuria? *Med Hypotheses* **91**, 77-80 (2016).
120. Zannoni, V.G., Lomtevas, N. & Goldfinger, S. Oxidation of Homogentisic Acid to Ochronotic Pigment in Connective Tissue. *Biochimica Et Biophysica Acta* **177**, 94-+ (1969).
121. Moran, G.R. 4-Hydroxyphenylpyruvate dioxygenase. *Arch Biochem Biophys* **433**, 117-128 (2005).
122. BURMESTER, T. Evolution and function of the insect hexamerins. *Eur. J. Entomol.* **96**, 213-225 (1999).
123. Burmester, T., Massey, H.C., Jr., Zakharkin, S.O. & Benes, H. The evolution of hexamerins and the phylogeny of insects. *J Mol Evol* **47**, 93-108 (1998).
124. Hahn, D.A. & Wheeler, D.E. Presence of a single abundant storage hexamerin in both larvae and adults of the grasshopper, Schistocerca americana. *J Insect Physiol* **49**, 1189-1197 (2003).
125. Burmester, T. Origin and evolution of arthropod hemocyanins and related proteins. *J Comp Physiol B* **172**, 95-107 (2002).
126. Hagner-Holler, S., Pick, C., Girgenrath, S., Marden, J.H. & Burmester, T. Diversity of stonefly hexamerins and implication for the evolution of insect storage proteins. *Insect Biochem Molec* **37**, 1064-1074 (2007).
127. Asano, T. & Takebuchi, K. Identification of the gene encoding pro-phenoloxidase A(3) in the fruitfly, Drosophila melanogaster. *Insect Mol Biol* **18**, 223-232 (2009).
128. Burmester, T., Massey, H.C., Zakharkin, S.O. & Benes, H. The evolution of hexamerins and the phylogeny of insects. *J Mol Evol* **47**, 93-108 (1998).
129. Telfer, W.H. & Kunkel, J.G. The Function and Evolution of Insect Storage Hexamers. *Annu Rev Entomol* **36**, 205-228 (1991).
130. Reddy, J.K. & Hashimoto, T. Peroxisomal beta-oxidation and peroxisome proliferator-activated receptor alpha: An adaptive metabolic system. *Annu Rev Nutr* **21**, 193-230 (2001).
131. Merilainen, G., Poikela, V., Kursula, P. & Wierenga, R.K. The Thiolase Reaction Mechanism: The Importance of Asn316 and His348 for Stabilizing the Enolate Intermediate of the Claisen Condensation. *Biochemistry* **48**, 11011-11025 (2009).
132. Thompson, S. et al. Mechanistic Studies on Beta-Ketoacyl Thiolase from Zoogloea-Ramigera - Identification of the Active-Site Nucleophile as Cys89, Its Mutation to Ser89, and Kinetic and Thermodynamic Characterization of Wild-Type and Mutant Enzymes. *Biochemistry* **28**, 5735-5742 (1989).

133. Seedorf, U., Brysch, P., Engel, T., Schrage, K. & Assmann, G. Sterol Carrier Protein-X Is Peroxisomal 3-Oxoacyl Coenzyme-a Thiolase with Intrinsic Sterol Carrier and Lipid Transfer Activity. *Journal of Biological Chemistry* **269**, 21277-21283 (1994).
134. Faust, J.E., Verma, A., Peng, C. & McNew, J.A. An inventory of peroxisomal proteins and pathways in Drosophila melanogaster. *Traffic* **13**, 1378-1392 (2012).
135. Lloyd, M.D., Darley, D.J., Wierzbicki, A.S. & Threadgill, M.D. alpha-Methylacyl-CoA racemase - an 'obscure' metabolic enzyme takes centre stage. *Febs J* **275**, 1089-1102 (2008).
136. Maeda, J. et al. Biosynthesis-inspired deracemizative production of d-luciferin by combining luciferase and thioesterase. *Biochim Biophys Acta Gen Subj* **1861**, 2112-2118 (2017).
137. Hunt, M.C., Siponen, M.I. & Alexson, S.E. The emerging role of acyl-CoA thioesterases and acyltransferases in regulating peroxisomal lipid metabolism. *Biochim Biophys Acta* **1822**, 1397-1410 (2012).
138. Brocker, C., Carpenter, C., Nebert, D.W. & Vasiliou, V. Evolutionary divergence and functions of the human acyl-CoA thioesterase gene (ACOT) family. *HUMAN GENOMICS* **4**, 411–420 (2010).
139. Tillander, V., Alexson, S.E.H. & Cohen, D.E. Deactivating Fatty Acids: Acyl-CoA Thioesterase-Mediated Control of Lipid Metabolism. *Trends Endocrinol Metab* **28**, 473-484 (2017).
140. Lipmann, F. Biological Sulfate Activation and Transfer. *Science* **128**, 575-580 (1958).
141. Suiko, M., Kurogi, K., Hashiguchi, T., Sakakibara, Y. & Liu, M.C. Updated perspectives on the cytosolic sulfotransferases (SULTs) and SULT-mediated sulfation. *Biosci Biotech Bioch* **81**, 63-72 (2017).
142. Sardiello, M., Annunziata, I., Roma, G. & Ballabio, A. Sulfatases and sulfatase modifying factors: an exclusive and promiscuous relationship. *Hum Mol Genet* **14**, 3203-3217 (2005).
143. Ma, X.L. et al. Structure and expression of sulfatase and sulfatase modifying factor genes in the diamondback moth, Plutella xylostella. *Insect science* (2017).
144. Venkatachalam, K.V., Akita, H. & Strott, C.A. Molecular cloning, expression, and characterization of human bifunctional 3 '-phosphoadenosine 5 '-phosphosulfate synthase and its functional domains. *J Biol Chem* **273**, 19311-19320 (1998).
145. Suzuki, N. & Goto, T. Firefly Bioluminescence .2. Identification of 2-(6'-Hydroxybenzothiazol-2'-Yl)-4-Hydroxthiazole as a Product in Bioluminescence of Firefly Lanterns and as a Product in Chemiluminescence of Firefly Luciferin in Dmso. *Tetrahedron Lett*, 2021-& (1971).
146. Gates, B.J. & Deluca, M. Production of Oxyluciferin during Firefly Luciferase Light Reaction. *Arch Biochem Biophys* **169**, 616-621 (1975).

147. Okada, K., Iio, H., Kubota, I. & Goto, T. Firefly Bioluminescence .3. Conversion of Oxyluciferin to Luciferin in Firefly. *Tetrahedron Lett*, 2771-2774 (1974).
148. Scott, S.H. & Bahnson, B.J. Senescence Marker Protein 30: Functional and Structural Insights to its Unknown Physiological Function. *Biomol Concepts* **2**, 469-480 (2011).
149. Gomi, K., Hirokawa, K. & Kajiyama, N. Molecular cloning and expression of the cDNAs encoding luciferin-regenerating enzyme from Luciola cruciata and Luciola lateralis. *Gene* **294**, 157-166 (2002).
150. Hansen, I.A., Meyer, S.R., Schafer, I. & Scheller, K. Interaction of the anterior fat body protein with the hexamerin receptor in the blowfly Calliphora vicina. *Eur J Biochem* **269**, 954-960 (2002).
151. Day, J.C. & Bailey, M.J. Structure and evolution of the luciferin-regenerating enzyme (LRE) gene from the firefly Photinus pyralis. *Insect Molecular Biology* **12**, 365–372 (2003).
152. Gomi, K. & Kajiyama, N. Oxyluciferin, a luminescence product of firefly luciferase, is enzymatically regenerated into luciferin. *J Biol Chem* **276**, 36508-36513 (2001).
153. Gomi, K., Hirokawa, K. & Kajiyama, N. Molecular cloning and expression of the cDNAs encoding luciferin-regenerating enzyme from Luciola cruciata and Luciola lateralis. *Gene* **294** (2002).
154. Emamzadeh, R., Hosseinkhani, S., Hemati, R. & Sadeghizadeh, M. RACE-based amplification of cDNA and expression of a luciferin-regenerating enzyme (LRE): An attempt towards persistent bioluminescent signal. *Enzyme and Microbial Technology* **47**, 159-165 (2010).
155. Luis A. del Río & Schrader, M. Proteomics Of Peroxisomes. *Subcellular Biochemistry* (2018).
156. Hanna, C.H., Hopkins, T.A. & Buck, J. Peroxisomes of the firefly lantern. *J Ultrastruct Res* **57**, 150-162 (1976).
157. Heiland, I. & Erdmann, R. Biogenesis of peroxisomes. Topogenesis of the peroxisomal membrane and matrix proteins. *Febs J* **272**, 2362-2372 (2005).
158. Subramani, S. Components involved in peroxisome import, biogenesis, proliferation, turnover, and movement. *Physiol Rev* **78**, 171-188 (1998).
159. Emanuelsson, O., Elofsson, A., von Heijne, G. & Cristobal, S. In silico prediction of the peroxisomal proteome in fungi, plants and animals. *Journal of Molecular Biology* **330**, 443-456 (2003).
160. Petriv, O.I., Tang, L., Titorenko, V.I. & Rachubinski, R.A. A new definition for the consensus sequence of the peroxisome targeting signal type 2. *Journal of Molecular Biology* **341**, 119-134 (2004).
161. Dinkel, H. et al. ELM-the database of eukaryotic linear motifs. *Nucleic Acids Research* **40**, D242-D251 (2012).
162. Van Ael, E. & Fransen, M. Targeting signals in peroxisomal membrane proteins. *Biochim Biophys Acta* **1763**, 1629-1638 (2006).

163. Halbach, A. et al. Function of the PEX19-binding site of human adrenoleukodystrophy protein as targeting motif in man and yeast - PMP targeting is evolutionarily conserved. *Journal of Biological Chemistry* **280**, 21176-21182 (2005).
164. Van Ael, E. & Fransen, M. Targeting signals in peroxisomal membrane proteins. *Bba-Mol Cell Res* **1763**, 1629-1638 (2006).
165. Theodoulou, F.L., Bernhardt, K., Linka, N. & Baker, A. Peroxisome membrane proteins: multiple trafficking routes and multiple functions? *Biochem J* **451**, 345-352 (2013).
166. SJ, G., GA, K. & S, S. Identification of Peroxisomal Targeting Signals Located at the Carboxy Terminus of Four Peroxisomal Proteins. *J Cell Biol.* **107(3):897-905** (1988).
167. Faber, K.N. et al. The N-terminus of amine oxidase of Hansenula polymorpha contains a peroxisomal targeting signal. *FEBS Lett* **357**, 115-120 (1995).
168. Baron, M.N., Klinger, C.M., Rachubinski, R.A. & Simmonds, A.J. A Systematic Cell-Based Analysis of Localization of Predicted Drosophila Peroxisomal Proteins. *Traffic* **17**, 536-553 (2016).
169. Urquhart, A.J., Kennedy, D., Gould, S.J. & Crane, D.I. Interaction of Pex5p, the type 1 peroxisome targeting signal receptor, with the peroxisomal membrane proteins Pex14p and Pex13p. *J Biol Chem* **275**, 4127-4136 (2000).
170. Fang, Y., Morrell, J.C., Jones, J.M. & Gould, S.J. PEX3 functions as a PEX19 docking factor in the import of class I peroxisomal membrane proteins. *Journal of Cell Biology* **164**, 863-875 (2004).
171. Hattula, K., Hirschberg, D., Kalkkinen, N., Butcher, S.J. & Ora, A. Association between the Intrinsically Disordered Protein PEX19 and PEX3. *Plos One* **9** (2014).
172. Matsuzaki, T. & Fujiki, Y. The peroxisomal membrane protein import receptor Pex3p is directly transported to peroxisomes by a novel Pex19p-and Pex16p-dependent pathway. *Journal of Cell Biology* **183**, 1275-1286 (2008).
173. Rokka, A. et al. Pxmp2 Is a Channel-Forming Protein in Mammalian Peroxisomal Membrane. *Plos One* **4** (2009).
174. Dermauw, W. & Van Leeuwen, T. The ABC gene family in arthropods: Comparative genomics and role in insecticide transport and resistance. *Insect Biochem Molec* **45**, 89-110 (2014).
175. Morita, M. & Imanaka, T. Peroxisomal ABC transporters: Structure, function and role in disease. *Bba-Mol Basis Dis* **1822**, 1387-1396 (2012).
176. Dean, M., Rzhetsky, A. & Allikmets, R. The human ATP-binding cassette (ABC) transporter superfamily. *Genome Res* **11**, 1156-1166 (2001).
177. Liu, S.M. et al. Genome-wide identification and characterization of ATP-binding cassette transporters in the silkworm, Bombyx mori. *Bmc Genomics* **12** (2011).
178. Broehan, G., Kroeger, T., Lorenzen, M. & Merzendorfer, H. Functional analysis of the ATP-binding cassette (ABC) transporter gene family of Tribolium castaneum. *Bmc Genomics* **14** (2013).

179. Kruh, G.D. & Belinsky, M.G. The MRP family of drug efflux pumps. *Oncogene* **22**, 7537-7552 (2003).
180. Hughes, C.L. & Kaufman, T.C. Hox genes and the evolution of the arthropod body plan. *Evolution & development* **4**, 459-499 (2002).
181. Stansbury, M.S. & Moczek, A.P. The function of Hox and appendage-patterning genes in the development of an evolutionary novelty, the Photuris firefly lantern. *P Roy Soc B-Biol Sci* **281** (2014).
182. Beeman, R.W., Stuart, J.J., Haas, M.S. & Denell, R.E. Genetic-Analysis of the Homeotic Gene-Complex (Hom-C) in the Beetle Tribolium-Castaneum. *Dev Biol* **133**, 196-209 (1989).
183. Tomita, S. & Kikuchi, A. Abd-B suppresses lepidopteran proleg development in posterior abdomen. *Developmental biology* **328**, 403-409 (2009).
184. Li, X. et al. Outbred genome sequencing and CRISPR/Cas9 gene editing in butterflies. *Nat Commun* **6**, 8212 (2015).
185. Li, X.Y. et al. Genome editing in the butterfly type-species Papilio machaon. *Insect Sci* **24**, 708-711 (2017).
186. Casanova, J., Sanchezherrero, E. & Morata, G. Identification and Characterization of a Parasegment Specific Regulatory Element of the Abdominal-B Gene of Drosophila. *Cell* **47**, 627-636 (1986).
187. Zavortink, M. & Sakonju, S. The Morphogenetic and Regulatory Functions of the Drosophila-Abdominal-B Gene Are Encoded in Overlapping Rnas Transcribed from Separate Promoters. *Genes & development* **3**, 1969-1981 (1989).
188. Celniker, S.E., Keelan, D.J. & Lewis, E.B. The Molecular-Genetics of the Bithorax Complex of Drosophila - Characterization of the Products of the Abdominal-B Domain. *Genes & development* **3**, 1424-1436 (1989).
189. Celniker, S.E., Sharma, S., Keelan, D.J. & Lewis, E.B. The Molecular-Genetics of the Bithorax Complex of Drosophila - Cis-Regulation in the Abdominal-B Domain. *Embo Journal* **9**, 4277-4286 (1990).
190. Li, X.Y. et al. Outbred genome sequencing and CRISPR/Cas9 gene editing in butterflies. *Nat Commun* **6** (2015).
191. Richards, S. et al. The genome of the model beetle and pest Tribolium castaneum. *Nature* **452**, 949-955 (2008).
192. Chen, L. et al. Advances in genome editing technology and its promising application in evolutionary and ecological studies. *Giga Science* **3**, 24 (2014).
193. Cong, L. et al. Multiplex Genome Engineering Using CRISPR/Cas Systems. *Science* **339**, 819-823 (2013).
194. Bassett, A.R., Tibbit, C., Ponting, C.P. & Liu, J.L. Highly Efficient Targeted Mutagenesis of Drosophila with the CRISPR/Cas9 System. *Cell reports* **4**, 220-228 (2013).
195. Ghiradella, H. & Schmidt, J.T. Fireflies at one hundred plus: A new look at flash control. *Integrative and comparative biology* **44**, 203-212 (2004).
196. Deluca, M. & Mcelroy, W.D. Kinetics of Firefly Luciferase Catalyzed Reactions. *Biochemistry-Us* **13**, 921-925 (1974).

197. Ghiradella, H. in Microscopic anatomy of invertebrates. (ed. M. Locke) 19 (Wiley-Liss, New York.; 1998).
198. Trimmer, B.A. et al. Nitric oxide and the control of firefly flashing. *Science* **292**, 2486-2488 (2001).
199. Trimmer, B.A., Aprille, J. & Modica-Napolitano, J. Nitric oxide signalling: insect brains and photocytes. *Biochem Soc Symp*, 65-83 (2004).
200. Aprille, J.R., Lagace, C.J., Modica-Napolitano, J. & Trimmer, B.A. Role of nitric oxide and mitochondria in control of firefly flash. *Integrative and comparative biology* **44**, 213-219 (2004).
201. Geller, D.A. et al. Molecular-Cloning and Expression of Inducible Nitric-Oxide Synthase from Human Hepatocytes. *P Natl Acad Sci USA* **90**, 3491-3495 (1993).
202. Nathanson, J.A. Octopamine Receptors, Adenosine-3',5'-Monophosphate, and Neural Control of Firefly Flashing. *Science* **203**, 65-68 (1979).
203. Armstrong, G.A.B., Shoemaker, K.L., Money, T.G.A. & Robertson, R.M. Octopamine mediates thermal preconditioning of the locust ventilatory central pattern generator via a cAMP/protein kinase A signaling pathway. *J Neurosci* **26**, 12118-12126 (2006).
204. Timmins, G.S., Robb, F.J., Wilmot, C.M., Jackson, S.K. & Swartz, H.M. Firefly flashing is controlled by gating oxygen to light-emitting cells. *J Exp Biol* **204**, 2795-2801 (2001).
205. Carlson, A.D. Is the firefly flash regulated by calcium? *Integrative and comparative biology* **44**, 220-224 (2004).
206. Csordas, G. & Hajnoczky, G. SR/ER-mitochondrial local communication: Calcium and ROS. *Bba-Bioenergetics* **1787**, 1352-1362 (2009).
207. Rizzuto, R. et al. Ca2+ transfer from the ER to mitochondria: When, how and why. *Bba-Bioenergetics* **1787**, 1342-1351 (2009).
208. Brookes, P.S., Yoon, Y.S., Robotham, J.L., Anders, M.W. & Sheu, S.S. Calcium, ATP, and ROS: a mitochondrial love-hate triangle. *Am J Physiol-Cell Ph* **287**, C817-C833 (2004).
209. Brookes, P.S., Yoon, Y., Robotham, J.L., Anders, M.W. & Sheu, S.-S. Calcium, ATP, and ROS a mitochondrial love-hate triangle. *Am J Physiol Cell Physiol* **287: C817–C833** (2004).
210. Evans, P.D. & Maqueira, B. Insect octopamine receptors: a new classification scheme based on studies of cloned Drosophila G-protein coupled receptors. *Invertebrate neuroscience : IN* **5**, 111-118 (2005).
211. Maqueira, B., Chatwin, H. & Evans, P.D. Identification and characterization of a novel family of Drosophila beta-adrenergic-like octopamine G-protein coupled receptors. *J Neurochem* **94**, 547-560 (2005).
212. Qi, Y.X. et al. A new Drosophila octopamine receptor responds to serotonin. *Insect Biochem Mol Biol* **90**, 61-70 (2017).
213. Metpally, R.P. & Sowdhamini, R. Cross genome phylogenetic analysis of human and Drosophila G protein-coupled receptors: application to functional annotation of orphan receptors. *BMC Genomics* **6**, 106 (2005).

214. Blenau, W. & Baumann, A. Molecular and Pharmacological Properties of Insect Biogenic Amine Receptors: Lessons From Drosophila melanogaster and Apis mellifera. *Archives of Insect Biochemistry and Physiology* **48**, 13–38 (2001).
215. Balfanz, S., Strünker, T., Frings, S. & Baumann, A. A family of octapamine receptors that specifically induce cyclic AMP production or Ca2+release inDrosophila melanogaster. *Journal of Neurochemistry* **93**, 440-451 (2005).
216. Evans, P.D. & Maqueira, B. Insect octopamine receptors: a new classification scheme based on studies of cloned Drosophila G-protein coupled receptors. *Invertebrate Neuroscience* **5**, 111-118 (2005).
217. Arakawa, S. et al. Cloning, localization, and Permanent of a Drosophila Octopamine Receptor. *Neuron* **2**, 343-354 (1990).
218. Hauser, F. et al. A genome-wide inventory of neurohormone GPCRs in the red flour beetle Tribolium castaneum. *Front Neuroendocrinol* **29**, 142-165 (2008).
219. Maqueira, B., Chatwin, H. & Evans, P.D. Identification and characterization of a novel family of Drosophila beta-adrenergic-like octopamine G-protein coupled receptors. *J Neurochem* **94**, 547-560 (2005).
220. Evans, P.D. & Maqueira, B. Insect octopamine receptors: a new classification scheme based on studies of cloned Drosophila G-protein coupled receptors. *Invertebrate neuroscience : IN* **5**, 9 (2005).
221. Quan, F., Wolfgang, W.J. & Forte, M.A. The Drosophila Gene Coding for the Alpha-Subunit of a Stimulatory G-Protein Is Preferentially Expressed in the Nervous-System. *P Natl Acad Sci USA* **86**, 4321-4325 (1989).
222. McCudden, C.R., Hains, M.D., Kimple, R.J., Siderovski, D.P. & Willard, F.S. G-protein signaling: back to the future. *Cell Mol Life Sci* **62**, 551-577 (2005).
223. Boto, T., Gomez-Diaz, C. & Alcorta, E. Expression Analysis of the 3 G-Protein Subunits, G alpha, G beta, and G gamma, in the Olfactory Receptor Organs of Adult Drosophila melanogaster. *Chem Senses* **35**, 183-193 (2010).
224. Quan, F. & Forte, M.A. 2 Forms of Drosophila-Melanogaster Gs-Alpha Are Produced by Alternate Splicing Involving an Unusual Splice Site. *Mol Cell Biol* **10**, 910-917 (1990).
225. Deng, Y. et al. The Stimulatory G alpha(s) Protein Is Involved in Olfactory Signal Transduction in Drosophila. *Plos One* **6** (2011).
226. Cooper, D.M.F., Mons, N. & Karpen, J.W. Adenylyl Cyclases and the Interaction between Calcium and Camp Signaling. *Nature* **374**, 421-424 (1995).
227. Antoni, F.A. New paradigms in cAMP signalling. *Mol Cell Endocrinol* **353**, 3-9 (2012).
228. Livingstone, M.S., Sziber, P.P. & QuinrQ, W.G. Loss of Calcium/Calmodulin Responsiveness in Adenylate Cyclase of rutabaga, a Drosophila Learning Mutant. *Cell* **37**, 205-215 (1994).
229. Levin, L.R. et al. The Drosophila Learning and Memory Gene rutabaga Encodes a Ca2+/Calmodulin-Responsive Adenylyl Cyclase. *Cell* **68**, 479-489 (1992).

230. Iourgenko, V., Kliot, B., Cann, M.J. & Levin, L.R. Cloning and characterization of aDrosophilaadenylyl cyclase homologous to mammalian type IX. *FEBS Letters* **413**, 104-108 (1997).
231. Iourgenko, V. & Levin, L.R. A calcium-inhibited Drosophila adenylyl cyclase. *Biochimica et Biophysica Acta* **1495**, 125-139 (2000).
232. Cann, M.J., Chung, E. & Levin, L.R. A new family of adenylyl cyclase genes in the male germline of Drosophila melanogaster. *Dev Genes Evol* **210**, 200–206 (2000).
233. Kamenetsky, M. et al. Molecular details of cAMP generation in mammalian cells: a tale of two systems. *J Mol Biol* **362**, 623-639 (2006).
234. Steegborn, C. Structure, mechanism, and regulation of soluble adenylyl cyclases —similarities and differences to transmembrane adenylyl cyclases. *Biochimica et Biophysica Acta* **1842**, 2535–2547 (2014).
235. Taylor, S.S. et al. PKA: a portrait of protein kinase dynamics. *Bba-Proteins Proteom* **1697**, 259-269 (2004).
236. Skalhegg, B.S. & Tasken, K. Specificity in the cAMP/PKA signaling pathway. Differential expression, regulation, and subcellular localization of subunits of PKA. *Front Biosci* **5**, D678-D693 (2000).
237. Melkndez, A. & Kalderon, W.L.a.D. Activity, Expression and Function of a Second Drosophila Protein Kinase A Catalytic Subunit Gene. *Genetics* **s141**, 1507-1520 (1995).
238. Foster, J.L., Jesse Jose GuttmanS, HallTII, L. & RosenS, O.M. Drosophila CAMP-dependent Protein Kinase. *THE JOURNAL OF BIOLOGICAL CHEMISTRY* **259**, 13049-13055 (1984).
239. Foster, J.L., Higgins, G.C. & Jacksonnll, F.R. Cloning, Sequence, and Expression of the Drosophila CAMP-dependent Protein Kinase Catalytic Subunit Gene. *THE JOURNAL OF BIOLOGICAL CHEMISTRY* **263**, 1676-1681 (1988).
240. I, D.K. & Rubin, G.M. Isolation and characterization of Drosophila cAMP-dependent protein kinase genes. *GENES & DEVELOPMENT* **2:1539-1556** (1988).
241. Bunney, T.D. & Katan, M. PLC regulation: emerging pictures for molecular mechanisms. *Trends Biochem Sci* **36**, 88-96 (2011).
242. Suh, P.G. et al. Multiple roles of phosphoinositide-specific phospholipase C isozymes. *Bmb Rep* **41**, 415-434 (2008).
243. Katan, M. New insights into the families of PLC enzymes: looking back and going forward. *Biochemical Journal* **391**, 3 (2005).
244. Balakrishnan, S.S., Basu, U. & Raghu, P. Phosphoinositide signalling in Drosophila. *Bba-Mol Cell Biol L* **1851**, 770-784 (2015).
245. Bootman, M.D., Berridge, M.J. & Roderick, H.L. Calcium signalling: More messengers, more channels, more complexity. *Curr Biol* **12**, R563-R565 (2002).
246. Berridge, M.J., Lipp, P. & Bootman, M.D. The versatility and universality of calcium signalling. *Nat Rev Mol Cell Bio* **1**, 11-21 (2000).

247. Santulli, G., Nakashima, R., Yuan, Q. & Marks, A.R. Intracellular calcium release channels: an update. *J Physiol-London* **595**, 3041-3051 (2017).
248. Berridge, M.J. Inositol Trisphosphate and Calcium Signaling. *Nature* **361**, 315-325 (1993).
249. Yoshida, Y. & Imai, S. Structure and function of inositol 1,4,5-trisphosphate receptor. *Jpn J Pharmacol* **74**, 125-137 (1997).
250. Berridge, M.J. Inositol trisphosphate and calcium signalling mechanisms. *Bba-Mol Cell Res* **1793**, 933-940 (2009).
251. Meissner, G. Ryanodine Receptor Ca2+ Release Channels and Their Regulation by Endogenous Effectors. *Annu Rev Physiol* **56**, 485-508 (1994).
252. Chorna, T. & Hasan, G. The genetics of calcium signaling in Drosophila melanogaster. *Bba-Gen Subjects* **1820**, 1269-1282 (2012).
253. Yamakage, M. & Namiki, A. Calcium channels - basic aspects of their structure, function and gene encoding; anesthetic action on the channels - a review. *Can J Anaesth* **49**, 151-164 (2002).
254. Ruiz-Velasco, V., Zhong, J.M., Hume, J.R. & Keef, K.D. Modulation of Ca2+ channels by cyclic nucleotide cross activation of opposing protein kinases in rabbit portal vein. *Circ Res* **82**, 557-565 (1998).
255. Nishiyama, M. et al. Cyclic AMP/GMP-dependent modulation of Ca2+ channels sets the polarity of nerve growth-cone turning. *Nature* **423**, 990-995 (2003).
256. Trautwein, W. & Hescheler, J. Regulation of Cardiac L-Type Calcium Current by Phosphorylation and G-Proteins. *Annu Rev Physiol* **52**, 257-274 (1990).
257. Takahashi, M., Seagar, M.J., Jones, J.F., Reber, B.F.X. & Catterall, W.A. Subunit Structure of Dihydropyridine-Sensitive Calcium Channels from Skeletal-Muscle. *P Natl Acad Sci USA* **84**, 5478-5482 (1987).
258. Tanabe, T. et al. Primary Structure of the Receptor for Calcium-Channel Blockers from Skeletal-Muscle. *Nature* **328**, 313-318 (1987).
259. Ly, C.V., Yao, C.K., Verstreken, P., Ohyama, T. & Bellen, H.J. Straightjacket is required for the synaptic stabilization of cacophony, a voltage-gated calcium channel alpha(1) subunit. *J Cell Biol* **181**, 157-170 (2008).
260. Ren, D.J., Xu, H.J., Eberl, D.F., Chopra, M. & Hall, L.M. A mutation affecting dihydropyridine-sensitive current levels and activation kinetics in Drosophila muscle and mammalian heart calcium channels. *J Neurosci* **18**, 2335-2341 (1998).
261. Eberl, D.F. et al. Genetic and developmental characterization of Dmca1D, a calcium channel alpha(1) subunit gene in Drosophila melanogaster. *Genetics* **148**, 1159-1169 (1998).
262. Zheng, W. et al. Cloning and Characterization of a Calcium-Channel Subunit from Drosophila-Melanogaster with Similarity to the Rat-Brain Alpha(1)-Type-D Isoform. *J Neurosci* **15**, 1132-1143 (1995).
263. Smith, L.A. et al. A Drosophila calcium channel alpha1 subunit gene maps to a genetic locus associated with behavioral and visual defects. *J Neurosci* **16**, 12 (1996).

264. UniProt, C. UniProt: a hub for protein information. *Nucleic Acids Res* **43**, D204-212 (2015).
265. Smith, L.A. et al. A Drosophila Calcium Channel a1 Subunit Gene Maps to a Genetic Locus Associated with Behavioral and Visual Defects. *The Journal of Neuroscience* **16(24):7868–7879** (1996).
266. Quintavalle, A. Voltage-Gated Calcium Channels in Honey Bees: Physiological Roles and Potential Targets for Insecticides. *BioSciences Master Reviews* (2013).
267. Kanamori, T. et al. Compartmentalized Calcium Transients Trigger Dendrite Pruning in Drosophila Sensory Neurons. *SCIENCE* **340** (2013).
268. Ly, C.V., Yao, C.K., Verstreken, P., Ohyama, T. & Bellen, H.J. straightjacket is required for the synaptic stabilization of cacophony, a voltage-gated calcium channel alpha1 subunit. *J Cell Biol* **181**, 157-170 (2008).
269. Fesenko, E.E., Kolesnikov, S.S. & Lyubarsky, A.L. Induction by Cyclic-Gmp of Cationic Conductance in Plasma-Membrane of Retinal Rod Outer Segment. *Nature* **313**, 310-313 (1985).
270. Kaupp, U.B. & Seifert, R. Cyclic nucleotide-gated ion channels. *Physiol Rev* **82**, 769-824 (2002).
271. Matulef, K. & Zagotta, W.N. Cyclic nucleotide-gated ion channels. *Annu Rev Cell Dev Bi* **19**, 23-44 (2003).
272. Craven, K.B. & Zagotta, W.N. CNG and HCN channels: Two peas, one pod. *Annu Rev Physiol* **68**, 375-401 (2006).
273. Jones, P. et al. InterProScan 5: genome-scale protein function classification. *Bioinformatics* **30**, 1236-1240 (2014).
274. Prakriya, M. & Lewis, R.S. Store-Operated Calcium Channels. *Physiol Rev* **95**, 1383-1436 (2015).
275. Putney, J.W. Forms and functions of store-operated calcium entry mediators, STIM and Orai. *Adv Biol Regul* **68**, 88-96 (2018).
276. Wang, J. et al. Orai1, 2, 3 and STIM1 promote store-operated calcium entry in pulmonary arterial smooth muscle cells. *Cell Death Discov* **3**, 17074 (2017).
277. Fowler, M.A. & Montell, C. Drosophila TRP channels and animal behavior. *Life Sci* **92**, 394-403 (2013).
278. Nilius, B. & Owsianik, G. The transient receptor potential family of ion channels. *Genome Biol* **12**, 218 (2011).
279. Bublitz, M., Poulsen, H., Morth, J.P. & Nissen, P. In and out of the cation pumps: P-Type ATPase structure revisited. *Curr Opin Struc Biol* **20**, 431-439 (2010).
280. Axelsen, K.B. & Palmgren, M.G. Evolution of substrate specificities in the P-type ATPase superfamily. *J Mol Evol* **46**, 84-101 (1998).
281. Palmgren, M.G. & Axelsen, K.B. Evolution of P-type ATPases. *Bba-Bioenergetics* **1365**, 37-45 (1998).
282. Okamura, H., Yasuhara, J.C., Fambrough, D.M. & Takeyasu, K. P-type ATPases in Caenorhabditis and Drosophila: Implications for evolution of the

P-type ATPase subunit families with special reference to the Na,K-ATPase and H,K-ATPase subgroup. *J Membrane Biol* **191**, 13-24 (2003).
283. Saier, M.H., Jr. et al. The Transporter Classification Database (TCDB): recent advances. *Nucleic Acids Res* **44**, D372-379 (2016).
284. Blaustein, M.P. & Lederer, W.J. Sodium calcium exchange: Its physiological implications. *Physiol Rev* **79**, 763-854 (1999).
285. Dipolo, R. & Beauge, L. Sodium/calcium exchanger: Influence of metabolic regulation on ion carrier interactions. *Physiol Rev* **86**, 155-203 (2006).
286. Kiedrowski, L., Brooker, C., Costa, E. & Wroblewski, J.T. Glutamate Impairs Neuronal Calcium Extrusion While Reducing Sodium-Gradient. *Neuron* **12**, 295-300 (1994).
287. Patterson, M., Sneyd, J. & Friel, D.D. Depolarization-induced calcium responses in sympathetic neurons: Relative contributions from Ca2+ entry, extrusion, ER/mitochondrial Ca2+ uptake and release, and Ca2+ buffering. *J Gen Physiol* **129**, 29-56 (2007).
288. He, C. & O'Halloran, D.M. Analysis of the Na+/Ca2+ Exchanger Gene Family within the Phylum Nematoda. *Plos One* **9** (2014).
289. Jones, P., Binns, D., McMenamin, C., McAnulla, C. & Hunter, S. The InterPro BioMart: federated query and web service access to the InterPro Resource. *Database-Oxford* (2011).
290. De Stefani, D., Raffaello, A., Teardo, E., Szabo, I. & Rizzuto, R. A forty-kilodalton protein of the inner membrane is the mitochondrial calcium uniporter. *Nature* **476**, 336-U104 (2011).
291. Baughman, J.M. et al. Integrative genomics identifies MCU as an essential component of the mitochondrial calcium uniporter. *Nature* **476**, 341-U111 (2011).
292. Patron, M. et al. MICU1 and MICU2 Finely Tune the Mitochondrial Ca2+ Uniporter by Exerting Opposite Effects on MCU Activity. *Mol Cell* **53**, 726-737 (2014).
293. Matesanz-Isabel, J. et al. Functional roles of MICU1 and MICU2 in mitochondrial Ca2+ uptake. *Bba-Biomembranes* **1858**, 1110-1117 (2016).
294. Choi, S. et al. Mitochondrial calcium uniporter in Drosophila transfers calcium between the endoplasmic reticulum and mitochondria in oxidative stress-induced cell death. *J Biol Chem* **292**, 14473-14485 (2017).
295. Michalak, M., Corbett, E.F., Mesaeli, N., Nakamura, K. & Opas, M. Calreticulin: one protein, one gene, many functions. *Biochem J* **344**, 281-292 (1999).
296. Berridge, M.J., Lipp, P. & Bootman, M.D. The versatility and universality of calcium signalling. *Nat. Rev. Mol. Cell Biol.* **1** (2000).
297. Zhao, Y.Z., Vanhoutte, P.M. & Leung, S.W.S. Vascular nitric oxide: Beyond eNOS. *J Pharmacol Sci* **129**, 83-94 (2015).
298. StevensTruss, R., Beckingham, K. & Marletta, M.A. Calcium binding sites of calmodulin and electron transfer by neuronal nitric oxide synthase. *Biochemistry-Us* **36**, 12337-12345 (1997).

299. K.Yamanaka, M., A.Saugstad, J., Hanson-Painton, O., J.McCarthy, B. & L.Tobin, S. Structure and expression of the Drosophila calmodulin gene. *Nucleic Acids Research* **15**, 3335-3348 (1987).
300. Zhao, Y.Z., Vanhoutte, P.M. & Leung, S.W.S. Endothelial Nitric Oxide Synthase-Independent Release of Nitric Oxide in the Aorta of the Spontaneously Hypertensive Rat. *J Pharmacol Exp Ther* **344**, 15-22 (2013).
301. Elphick, M.R., Green, I.C. & Oshea, M. Nitric-Oxide Synthesis and Action in an Invertebrate Brain. *Brain Res* **619**, 344-346 (1993).
302. Ohtsuki, H., Yokoyama, J., Ohba, N., Ohmiya, Y. & Kawata, M. Nitric oxide synthase (NOS) in the Japanese fireflies Luciola lateralis and Luciola cruciata. *Arch Insect Biochem Physiol* **69**, 176-188 (2008).
303. Ohtsuki, H., Yokoyama, J., Ohba, N., Ohmiya, Y. & Kawata, M. Expression of the nos gene and firefly flashing: A test of the nitric-oxide-mediated flash control model. *Insect Science* **14** (2014).
304. Imamura, M., Yang, J. & Yamakawa, M. cDNA cloning, characterization and gene expression of nitric oxide synthase from the silkworm, Bombyx mori. *Insect Molecular Biology* **11**, 257–265 (2002).
305. REGULSKI, M. & TULLY, T. Molecular and biochemical characterization of dNOS: A Drosophila Ca2+/calmodulin-dependent nitric oxide synthase. *Proc. Natl. Acad. Sci.* **92** (1995).
306. Mu¨ller, U. Inhibition of Nitric Oxide Synthase Impairs a Distinct Form of Long-Term Memory in the Honeybee, Apis mellifera. *Neuron* **16**, 541–549 (1996).

Supplementary Figures

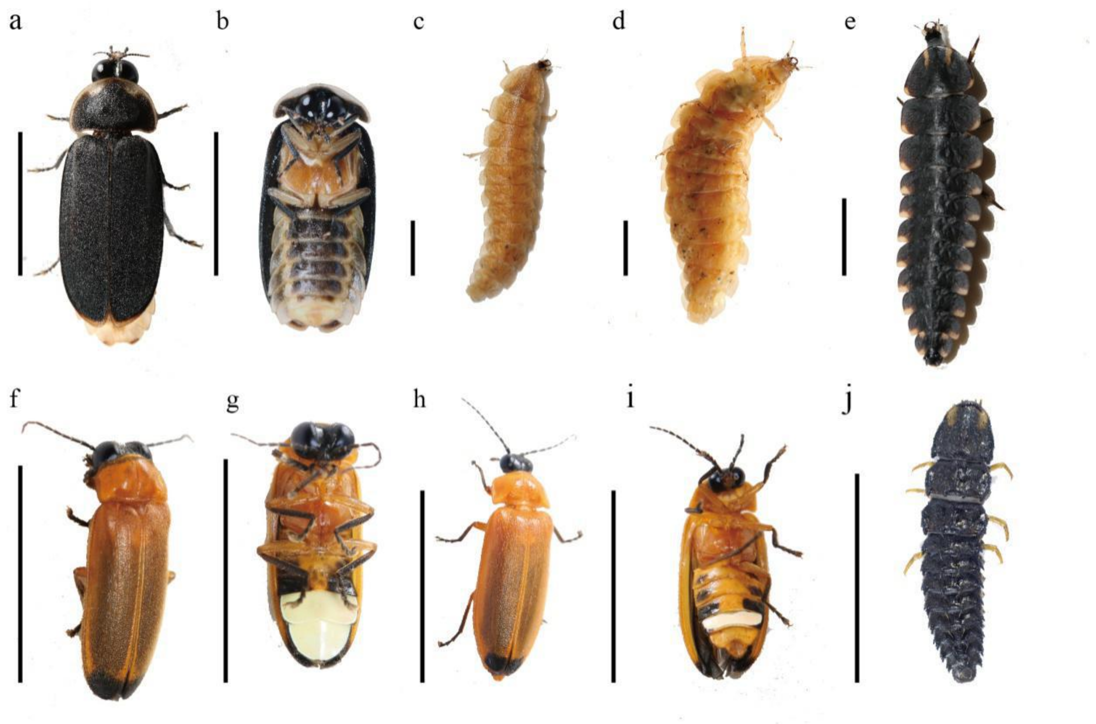

Figure S1. Outer morphology of *L. yunnana* (a-e) and *A. terminalis* (f-j). **a** and **f**, dorsal view of male adults. **b** and **g**, ventral view of male adults. **c** and **h**, dorsal view of female adults. **d** and **i**, ventral view of female adults. **e** and **j**, dorsal view of last instar larva. Scale bars: 1 mm.

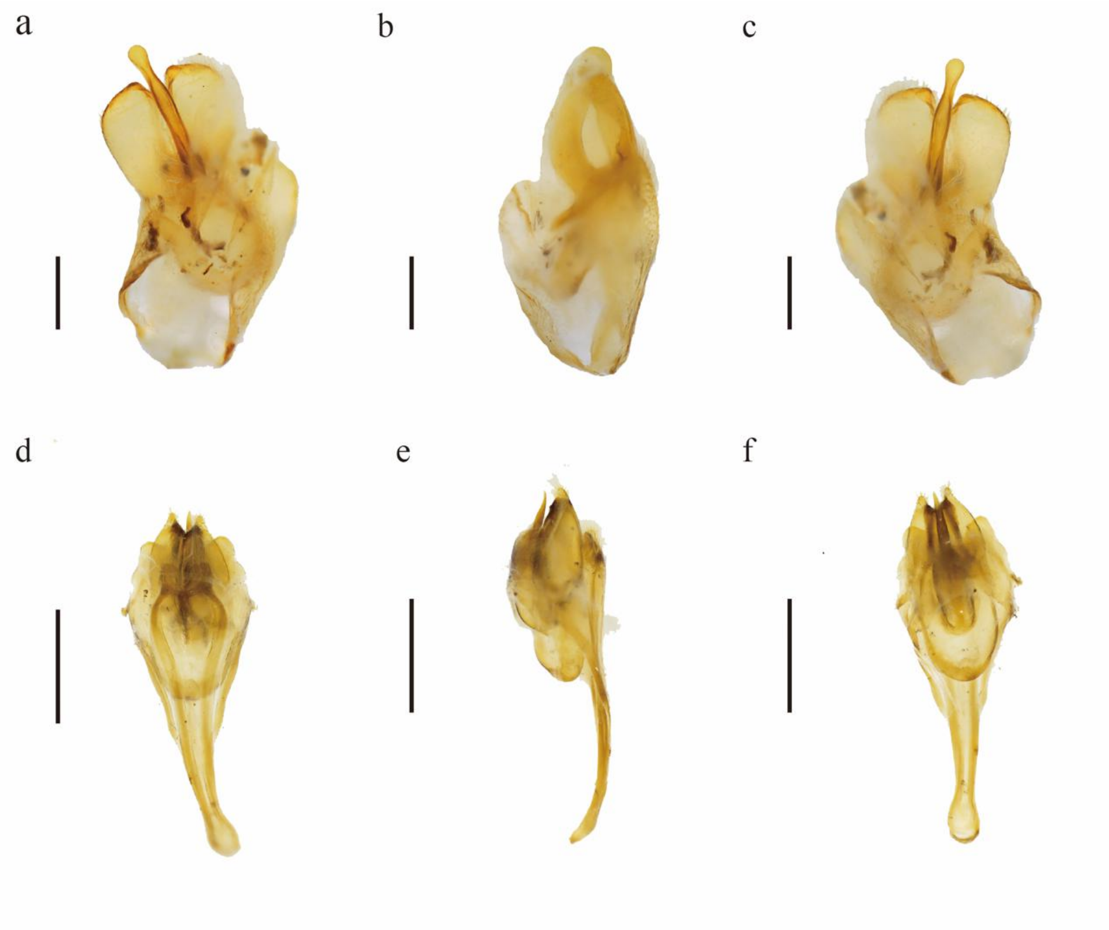

Figure S2. Male genetalia of *L. yunnana* (a-c) and *A. terminalis* (d-f). a and **d**, dorsal view. **b** and **e**, lateral view. **c** and **f**, ventral view. Scale bars: 1 mm.

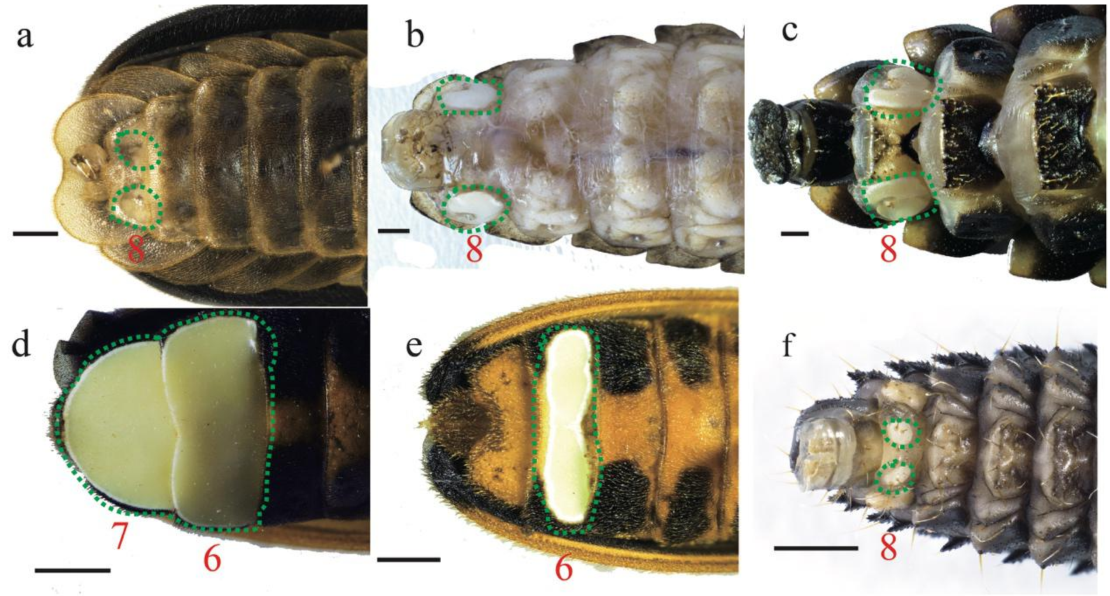

Figure S3. Luminous organs (dotted green regions) of *L. yunnana* (a-c) and *A. terminalis* (d-f). a and **d**, male adults. **b** and **e**, female adults. **c** and **f**, last instar larva. Numbers show the position of the sternites occupied by luminous organs. Scale bars: 1 mm.

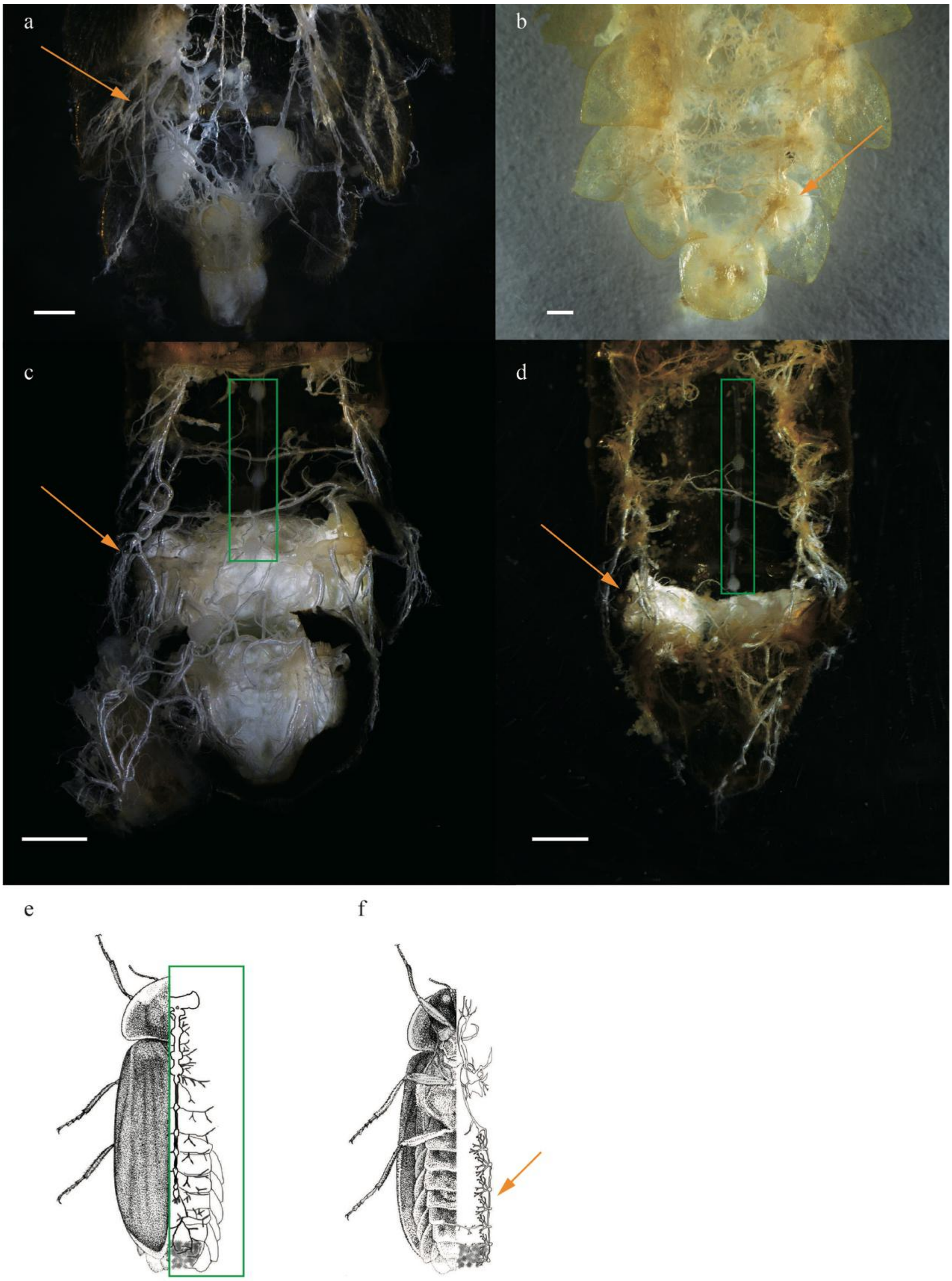

Figure S4. Inner structures of luminous organs, tracheae and nerves of *L. yunnana* (a, b, e, f) and *A. terminalis* (c, d). Orange arrows show the tracheae and green squares show the nerves. **a, c**, **e and f** represent male adults, of which **e** and **f** were hand-drawn based on observing nerves and tracheae of *L. yunnana* male adult. **b** and **d** represent female adult. Scale bars: 5 mm.

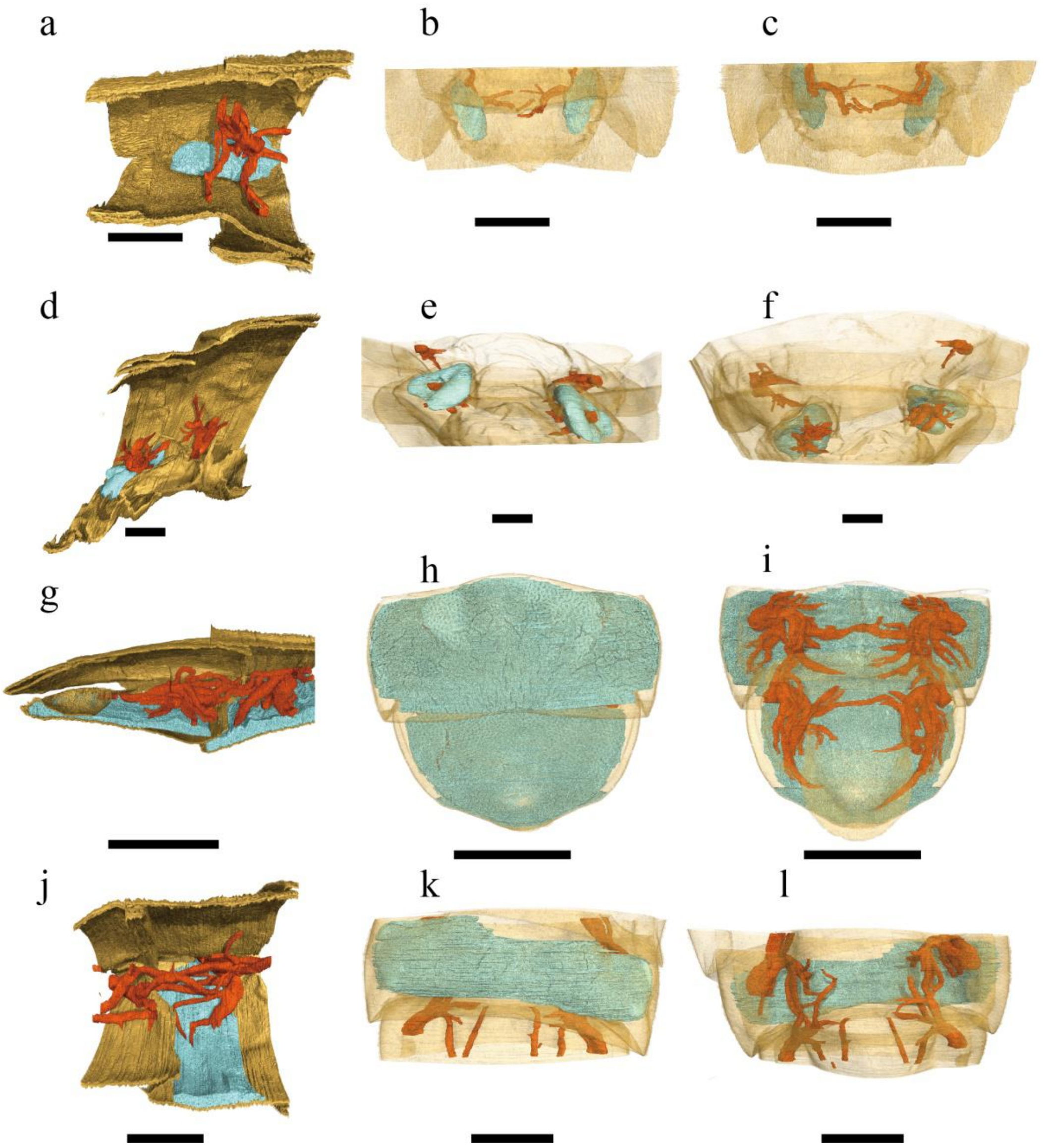

Figure S5. Three-dimension reconstruction of luminous organs of *L. yunnana* (a-f) and *A. terminalis* (g-l). Blue and orange regions denote luminous organs and tracheae, respectively. **a** and **g**, male adults lateral view. **d** and **j**, female adult lateral view. **b** and **h**, male adults ventral view. **e** and **k**, female adults ventral view. **c** and **i**, male adults dorsal view. **f** and **l**, female adults dorsal view. Scale bars: 1 mm.

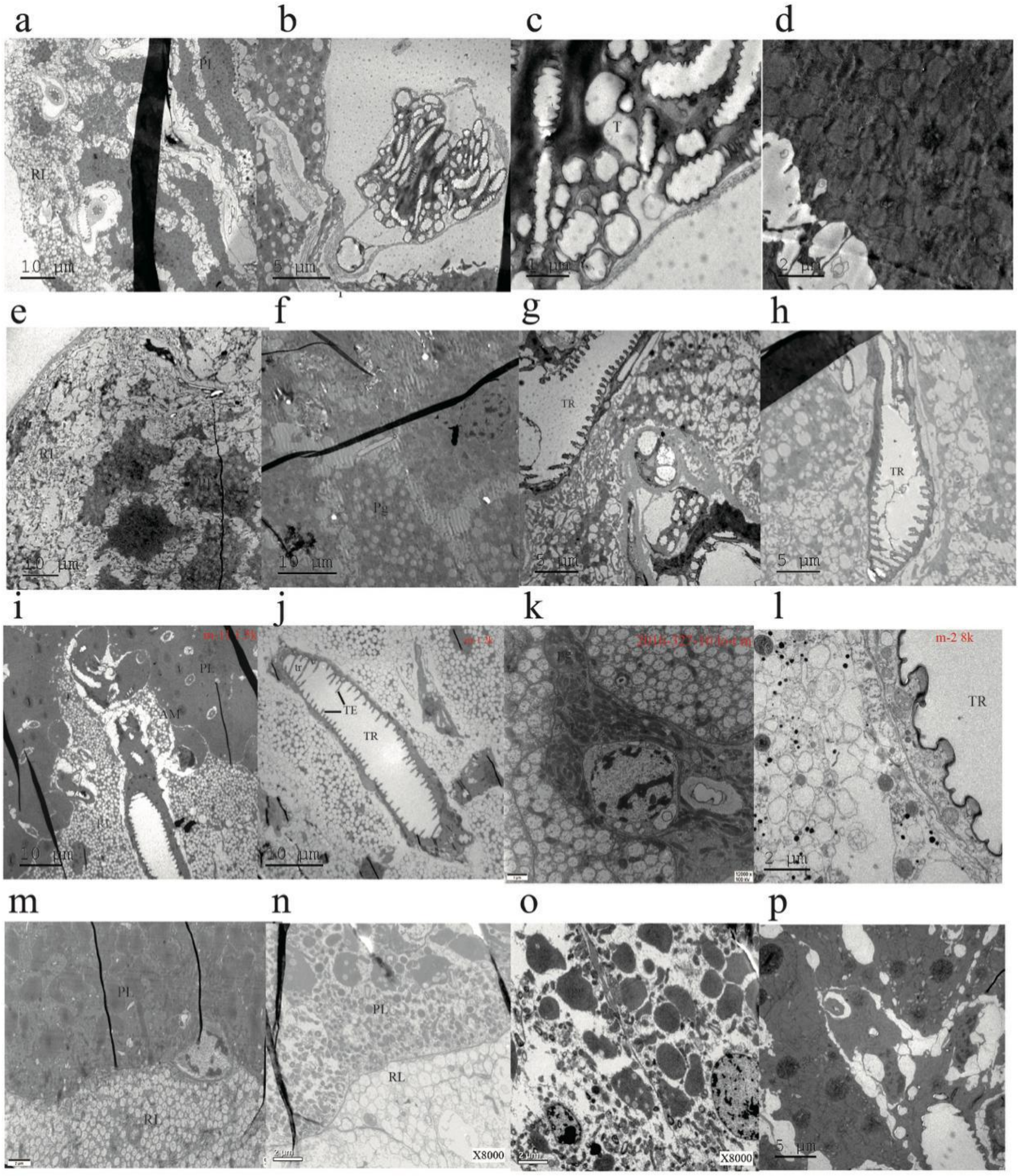

Figure S6. The structure of luminous organs of *L. yunnana* (a-h) and *A. terminalis* (i-p) observed by Transmission Electron Microscrope (TEM). a-d and **i-l**, male adults. **e-h** and **m-p**, female adult. RL: reflecting layer; PL: photogenic layer; pg: photocyte granule; TR: trachea, tr: tracheole.

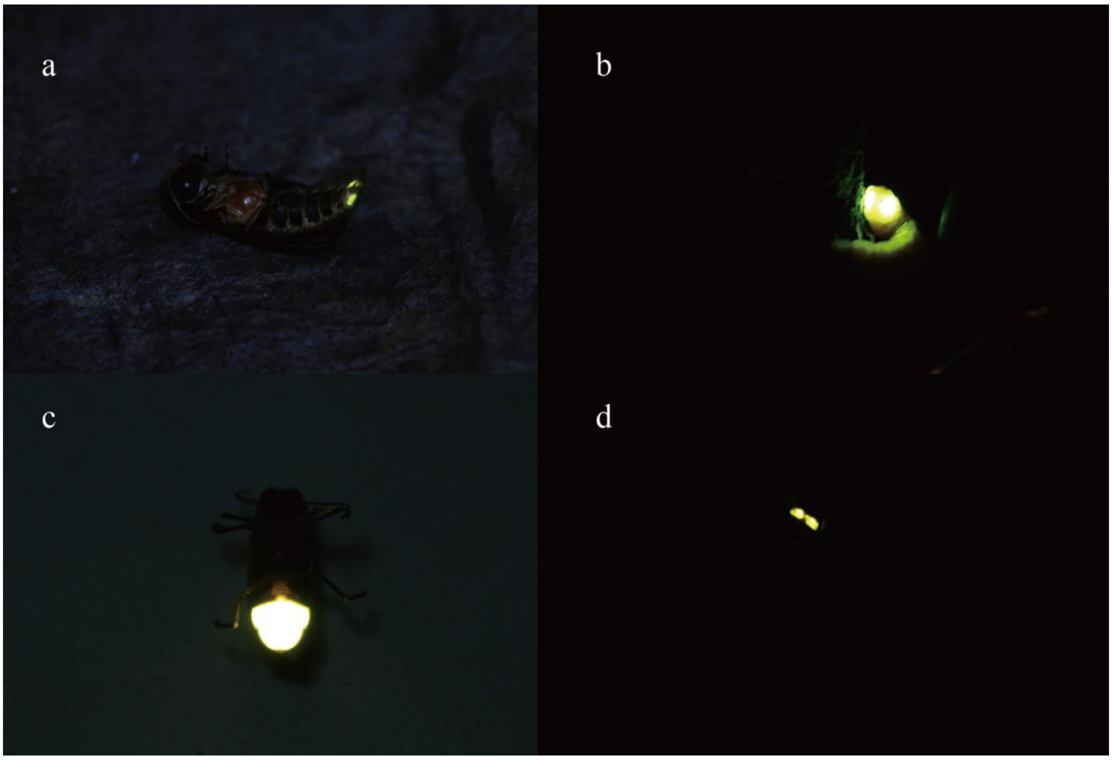

Figure S7. Bioluminescence of *L. yunnana* (a, b) and *A. terminalis* (c, d). a and **c**, male adults. **b** and **d**, female adult. (photo by Canon EOS 70D, 100mm, F6, T:1/30-1s)

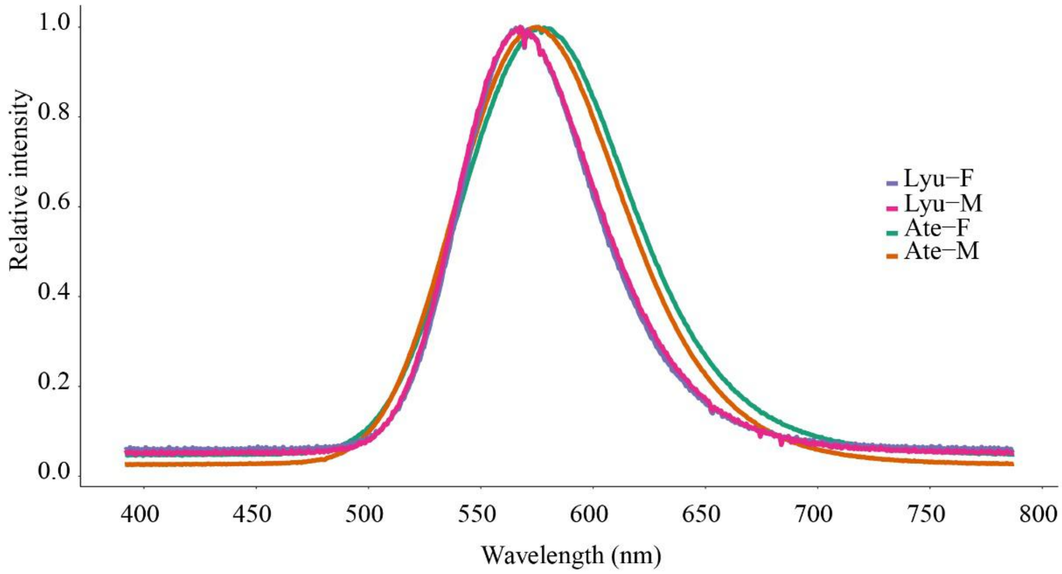

Figure S8. Emission spectrum of live *L. yunnana* (Lyu) and *A. terminalis* (Ate) in vivo. Spectrums were measured using LumiFL spectrocapture AB1850 (ATTO, Japan). F: female, M: male. R package was used for the visualization of the image.

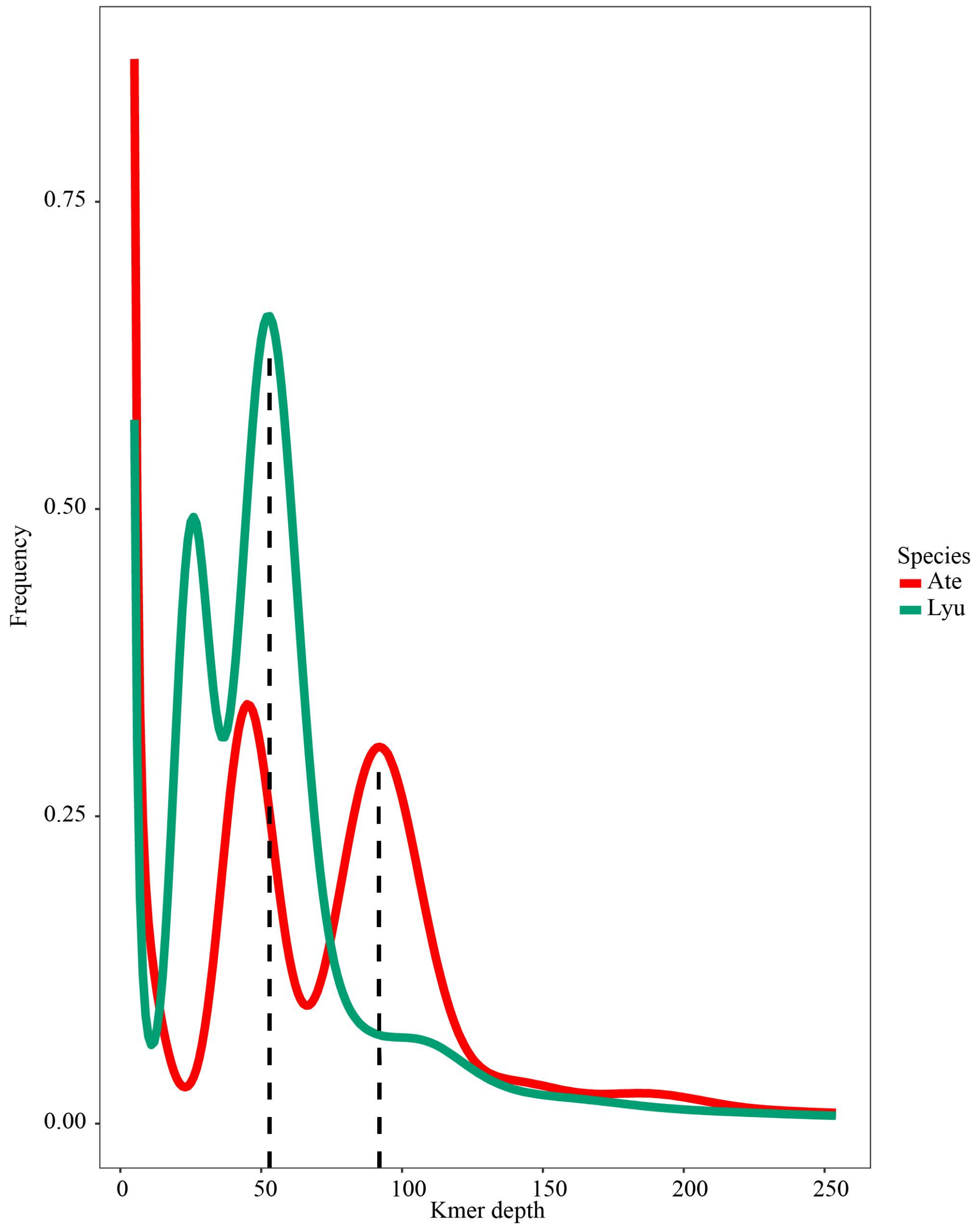

Figure S9. *K*-mer frequency distribution curves of the genomes of *L. yunnana* (Lyu) and *A. terminalis* (Ate). The distribution curves for each species are estimated using Illumina short reads from 350 bp libraries. The bimodality of the curves and the depth of the first peak that is half of the second peak indicate that the genomes have obvious signature of heterozygosis. The black dotted lines indicate peak depth. R package was used for the visualization of the image.

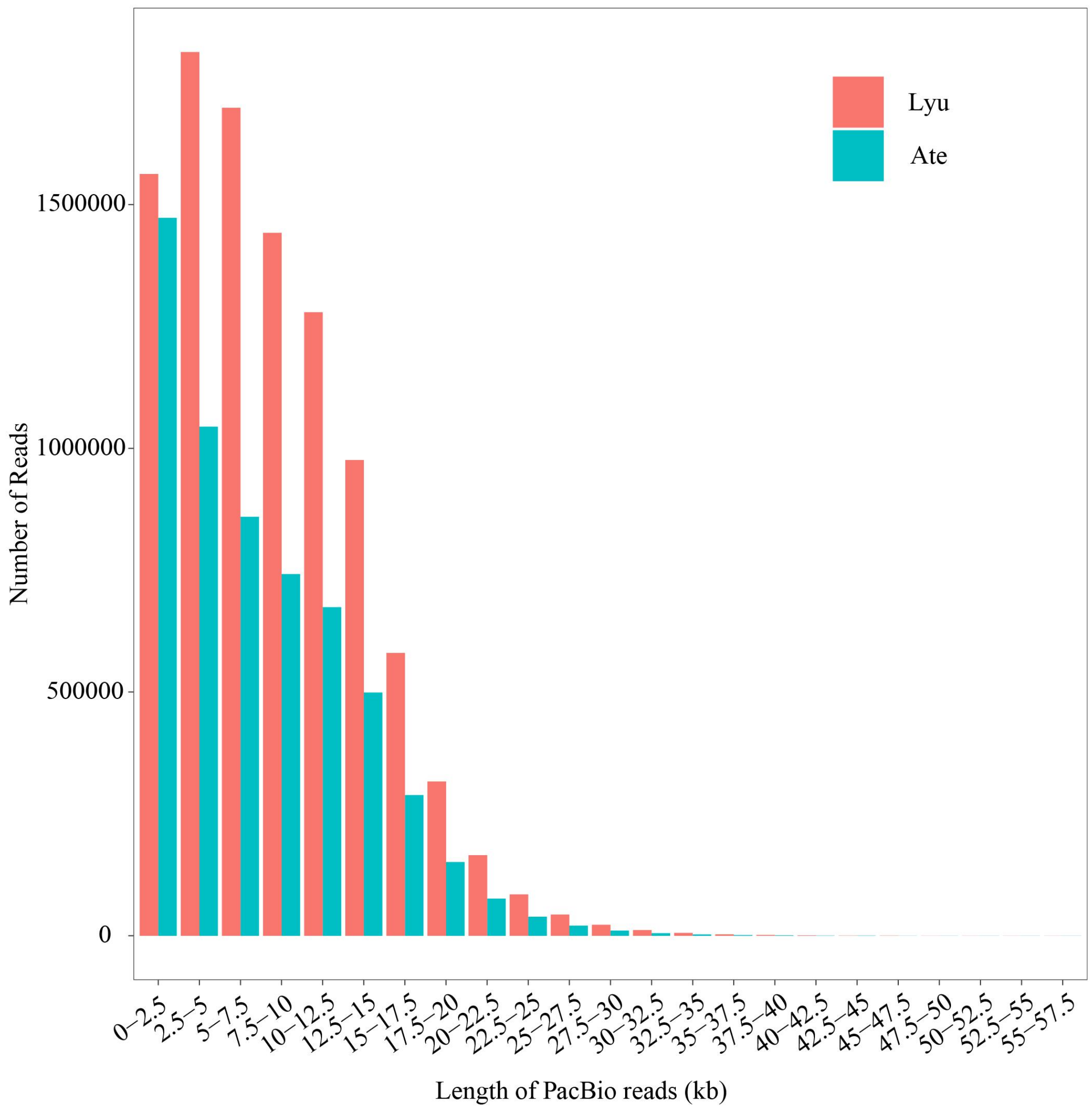

Figure S10. The length distribution of SMRT (Single-molecule real-time) reads sequenced using PacBio RS II platform for *L. yunnana* (Lyu) and *A. terminalis* (Ate). R package was used for the visualization of the image.

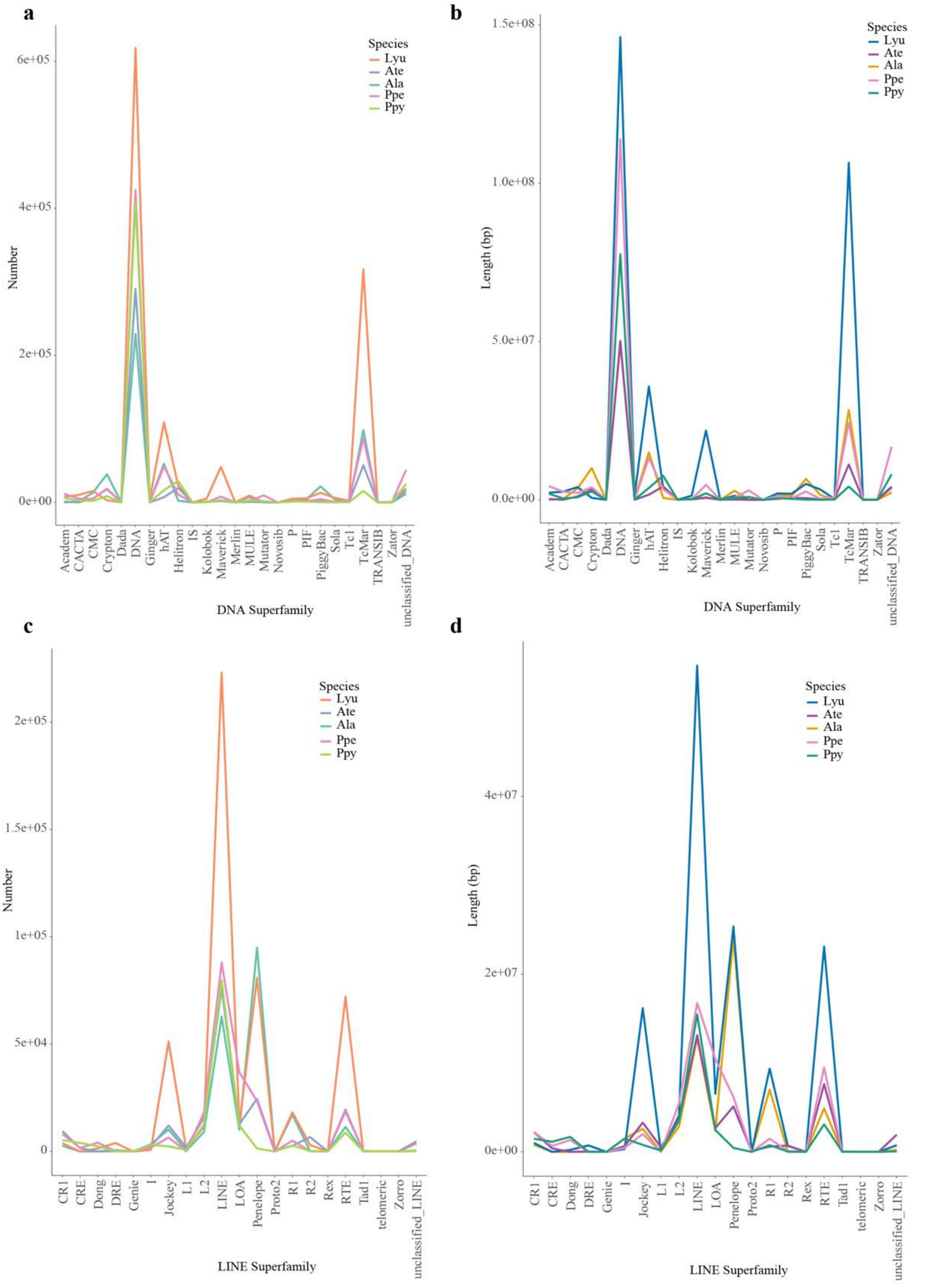

Figure S11. The statistics of the number and length of the superfamilies of DNA transposons and long interspersed nuclear elements (LINEs) among five firefly species. *Lamprigera yunnana* (Lyu), *Abscondita terminalis* (Ate), *Aquatica lateralis* (Ala), *Photinus pyralis* (Ppy), *Pyrocoelia pectoralis* (Ppe). R package was used for the visualization of the images.

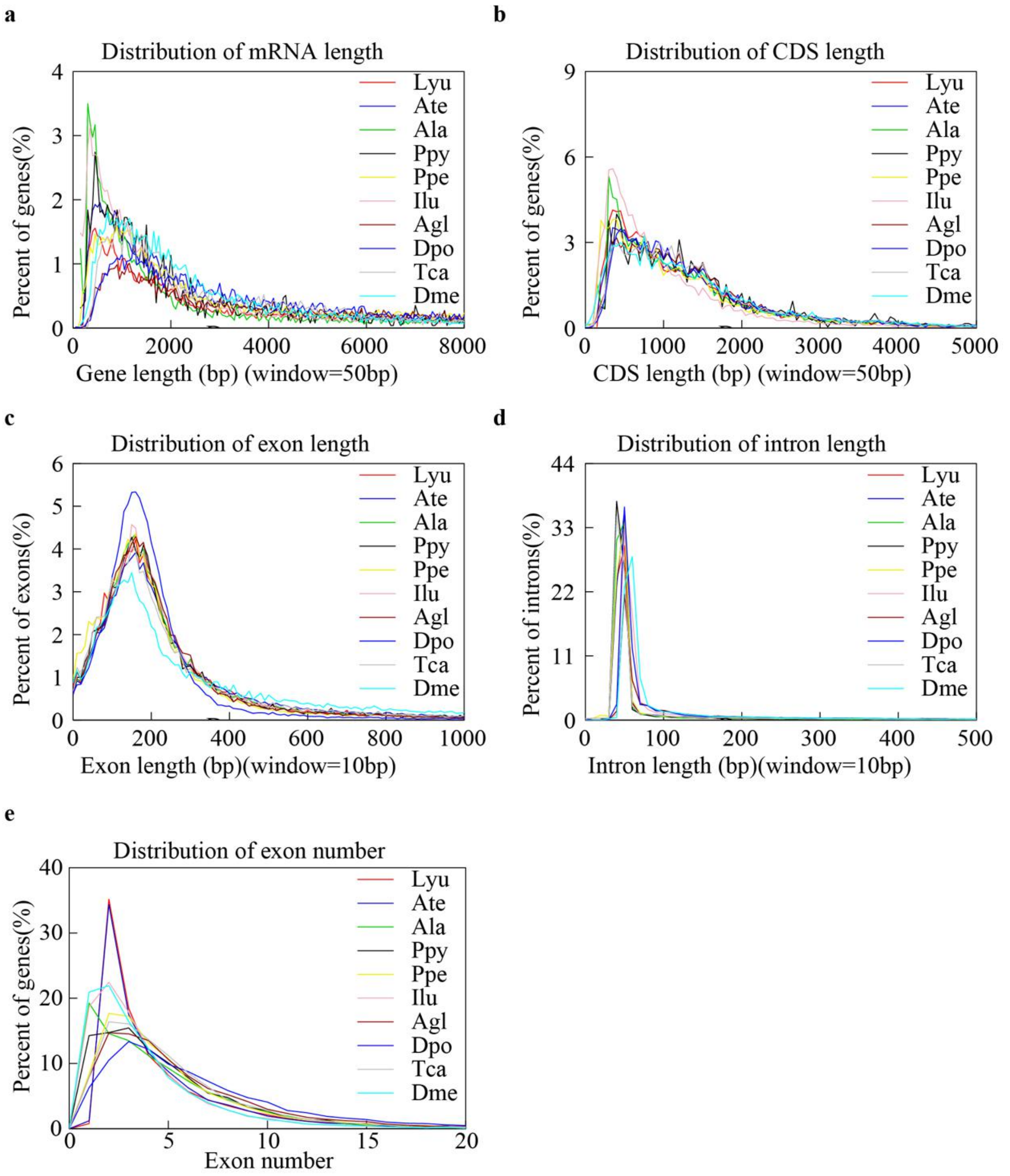

Figure S12. Comparisons of gene features among the genomes of beetles (luminous and non-luminous) and fruit fly. **a** represents the distribution of mRNA length. **b** represents the distribution of CDS length. **c** represents the distribution of exon length. **d** represents the distribution of intron length. **e** represents the distribution of exon number. Luminous beetles: *Lamprigera yunnana* (Lyu), *Abscondita terminalis* (Ate), *Aquatica lateralis* (Ala), *Photinus pyralis* (Ppy), *Pyrocoelia pectoralis* (Ppe), *Ignelater luminosus* (Ilu); non-luminous beetles: *Anoplophora glabripennis* (Agl), *Dendroctonus ponderosae* (Dpo), *Tribolium castaneum* (Tca); fruit fly: *Drosophila melanogaster* (Dme). R package was used for the visualization of the images.

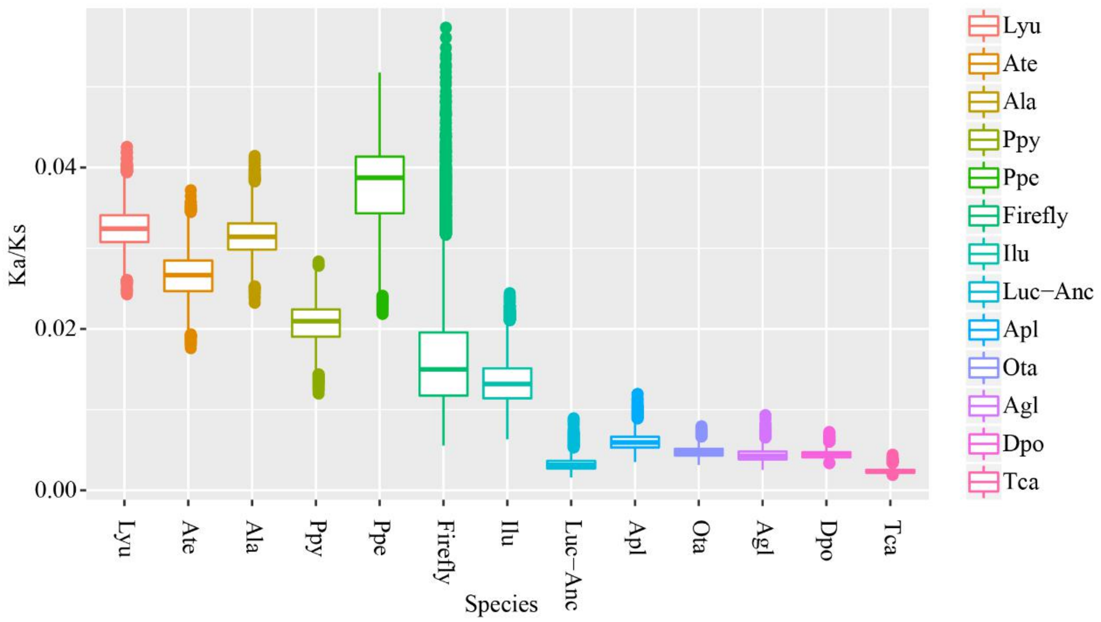

Figure S13. The average ω (*Ka/Ks*) value of 11 coleoptera species (6 luminous beetles and 5 non-luminous beetles), and most recent common ancestor (MRCA) of fireflies (Lampyridae) and Lampyridae-Elateridae beetles. Lampyridae: *Lamprigera yunnana* (Lyu), *Abscondita terminalis* (Ate), *Aquatica lateralis* (Ala), *Photinus pyralis* (Ppy), *Pyrocoelia pectoralis* (Ppe); Firefly: the ancestor of Lampyridae; Elateridae: *Ignelater luminosus* (Ilu); Luc-Anc: the ancestor of Lampyridae-Elateridae; non-luminous beetles: *Agrilus planipennis* (Apl), *Onthophagus taurus* (Ota), *Anoplophora glabripennis* (Agl), *Dendroctonus ponderosae* (Dpo), *Tribolium castaneum* (Tca). R package was used for the visualization of the image.

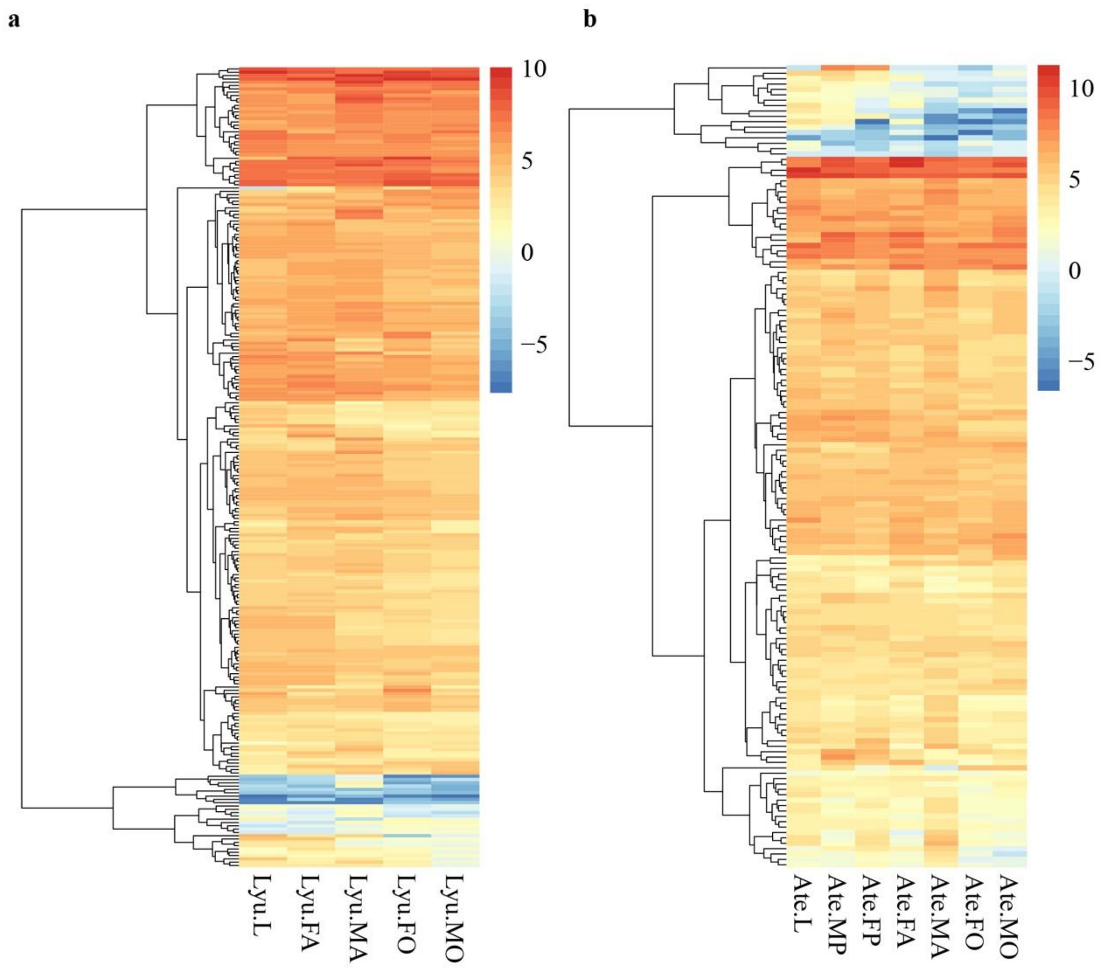

Figure S14. The expression of rapidly evolving genes (REGs) in *L. yunnana* (Lyu) (a) and *A. terminalis* (Ate) (b) at transcriptomic level. Heat map shows log2-scaled fragments per kilobase of transcript per million fragments mapped (FPKM). MO: luminous organ of male adult; FO: luminous organ of female adult; MA: male adult; FA: female adult; MP: male pupa; FP: female pupa; L: larva. R package was used for the visualization of the images.

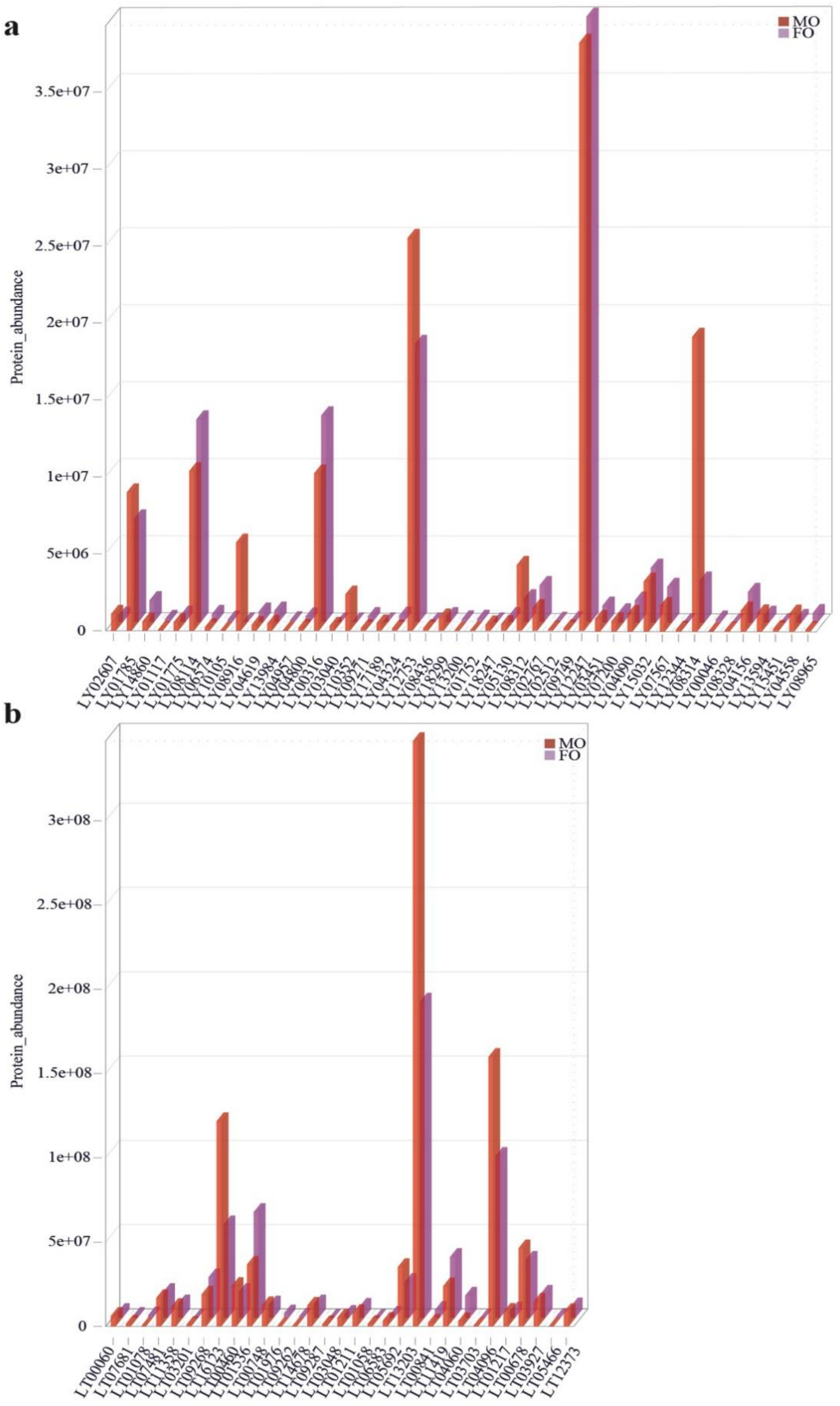

Figure S15. The expression of rapidly evolving genes (REGs) in *L. yunnana* (a) and *A. terminalis* (b) at proteomic level. MO: luminous organ of male adult; FO: luminous organ of female adult. R package was used for the visualization of the images.

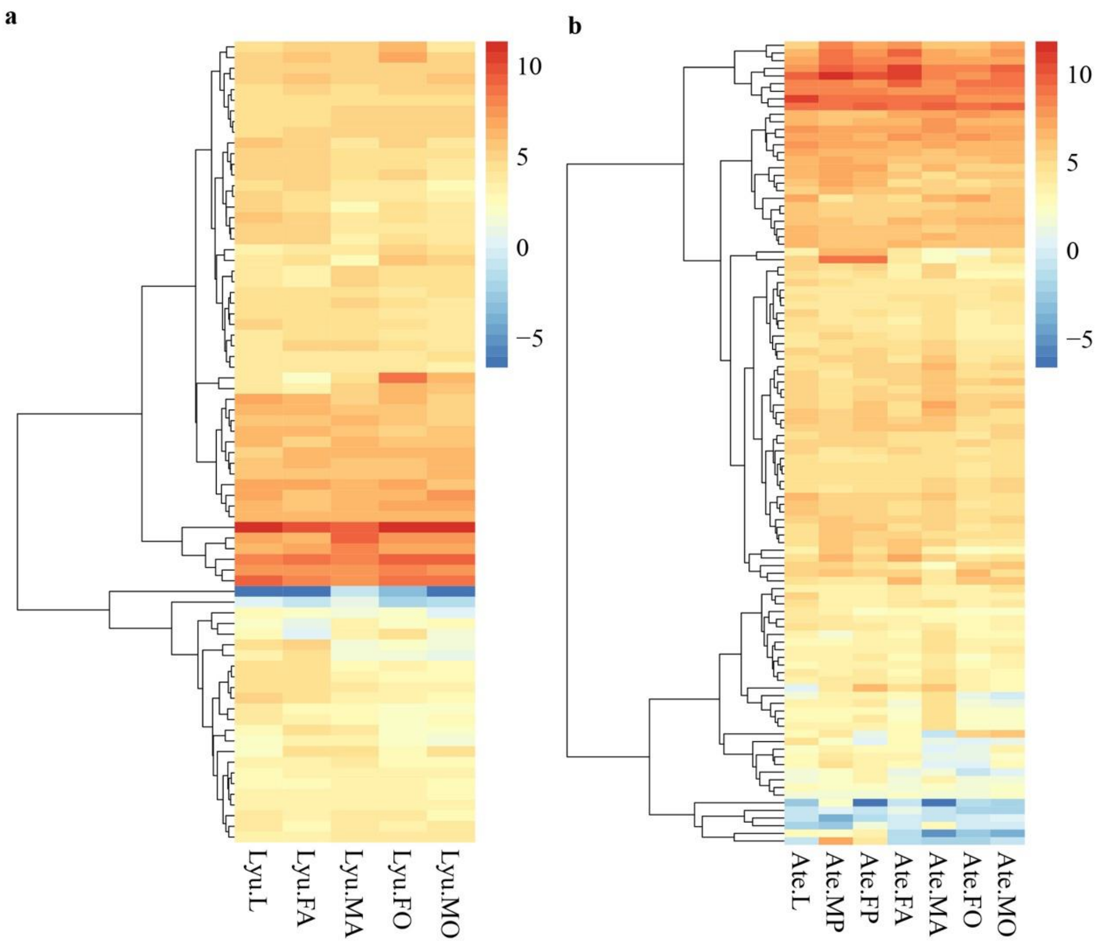

Figure S16. The expression of positive selected genes (PSGs) in *L. yunnana* (Lyu) (a) and *A. terminalis* (Ate) (b) at transcriptomic level. Heat map shows log2-scaled fragments per kilobase of transcript per million fragments mapped (FPKM). MO: luminous organ of male adult; FO: luminous organ of female adult; MA: male adult; FA: female adult; MP: male pupa; FP: female pupa; L: larva. R package was used for the visualization of the images.

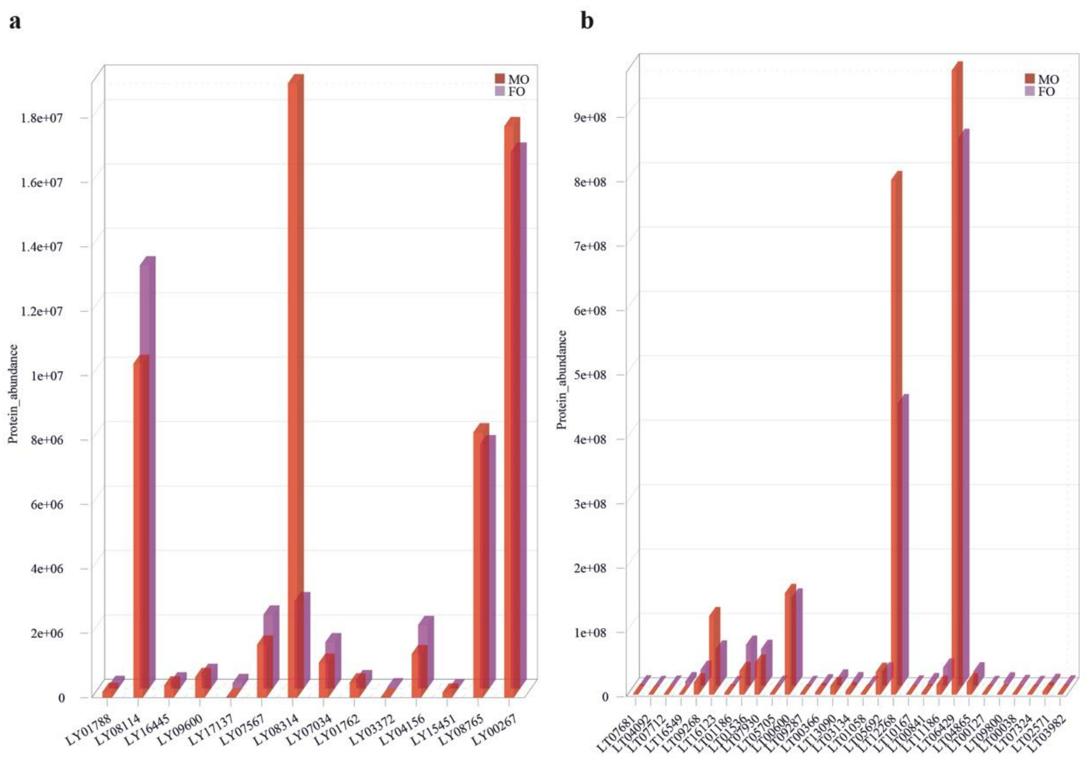

Figure S17. The expression of positive selected genes (PSGs) in *L. yunnana* (a) and *A. terminalis* (b) at proteomic level. MO: luminous organ of male adult; FO: luminous organ of female adult. R package was used for the visualization of the images.

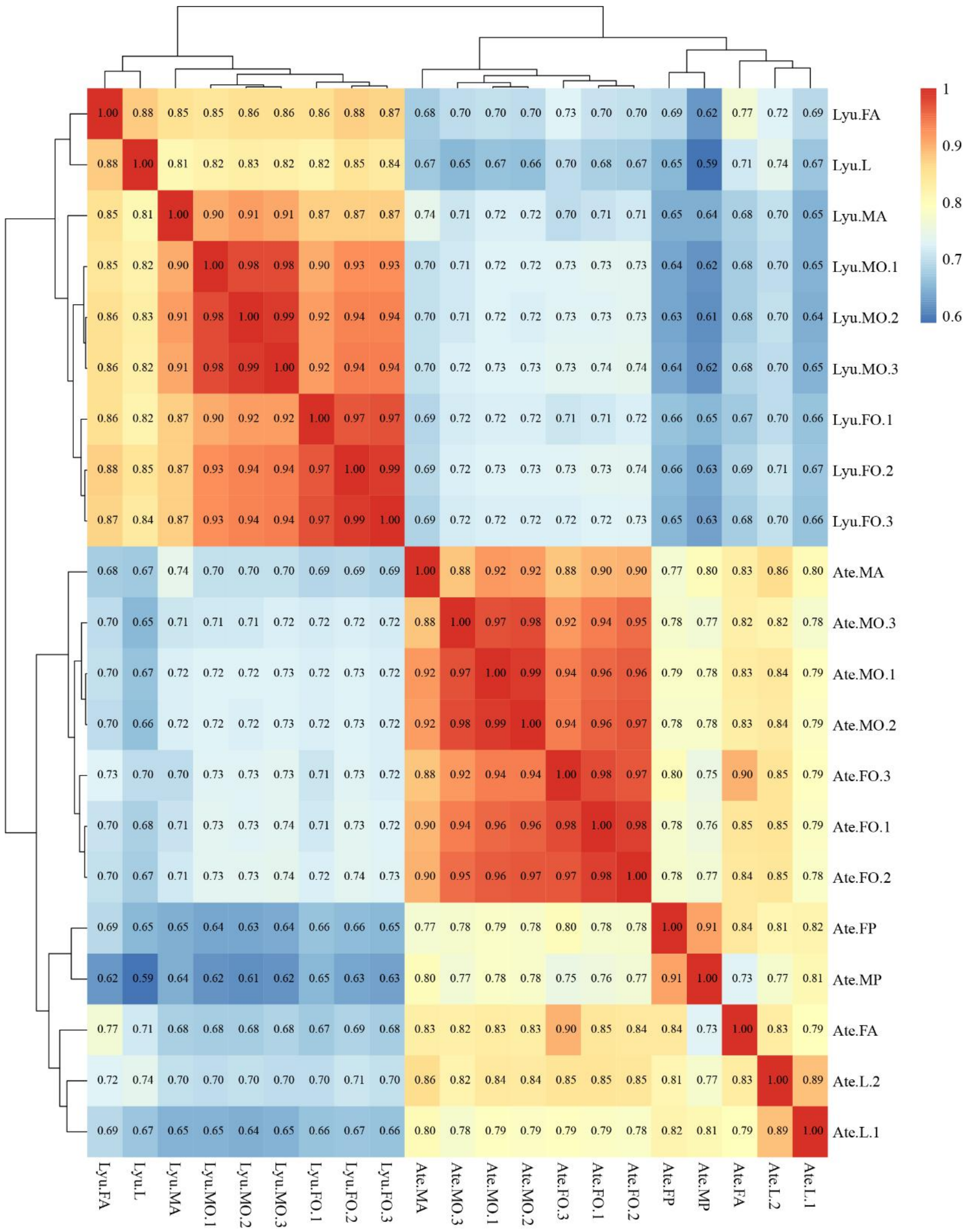

Figure S18. Expression pattern of orthologous genes in *L. yunnana* (Lyu) and *A. terminalis* (Ate). MO: luminous organ of male adult; FO: luminous organ of female adult; MA: male adult; FA: female adult; MP: male pupa; FP: female pupa; L: larva. The number after each sample represents sample duplication. R package was used for the visualization of the image.

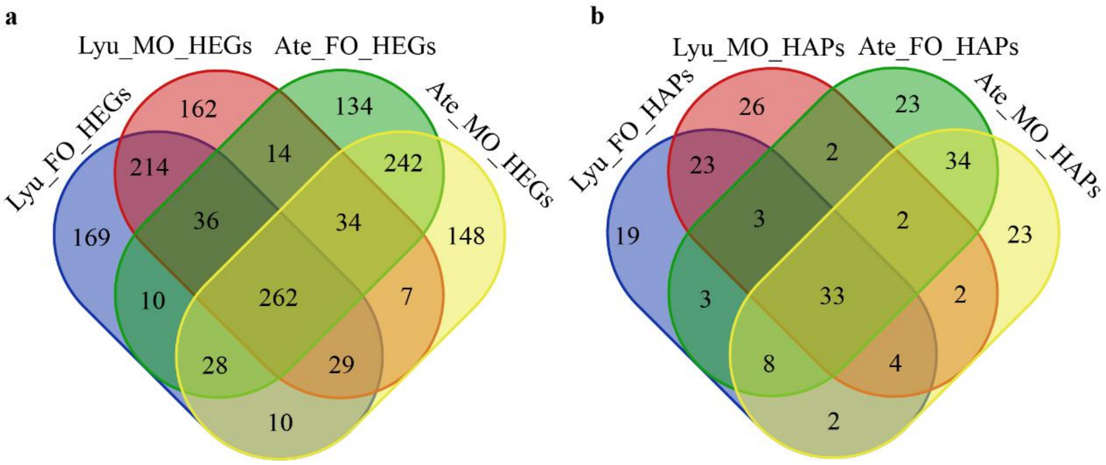

Figure S19. Venn map of high expression genes (HEGs) (a) and high abundance proteins (HAPs) (b) in luminous organs of *L. yunnana* (Lyu) and *A. terminalis* (Ate). MO: luminous organ of male adult; FO: luminous organ of female adult. The Venn maps were created using Calculate and draw custom Venn diagrams (http://bioinformatics.psb.ugent.be/webtools/Venn/).

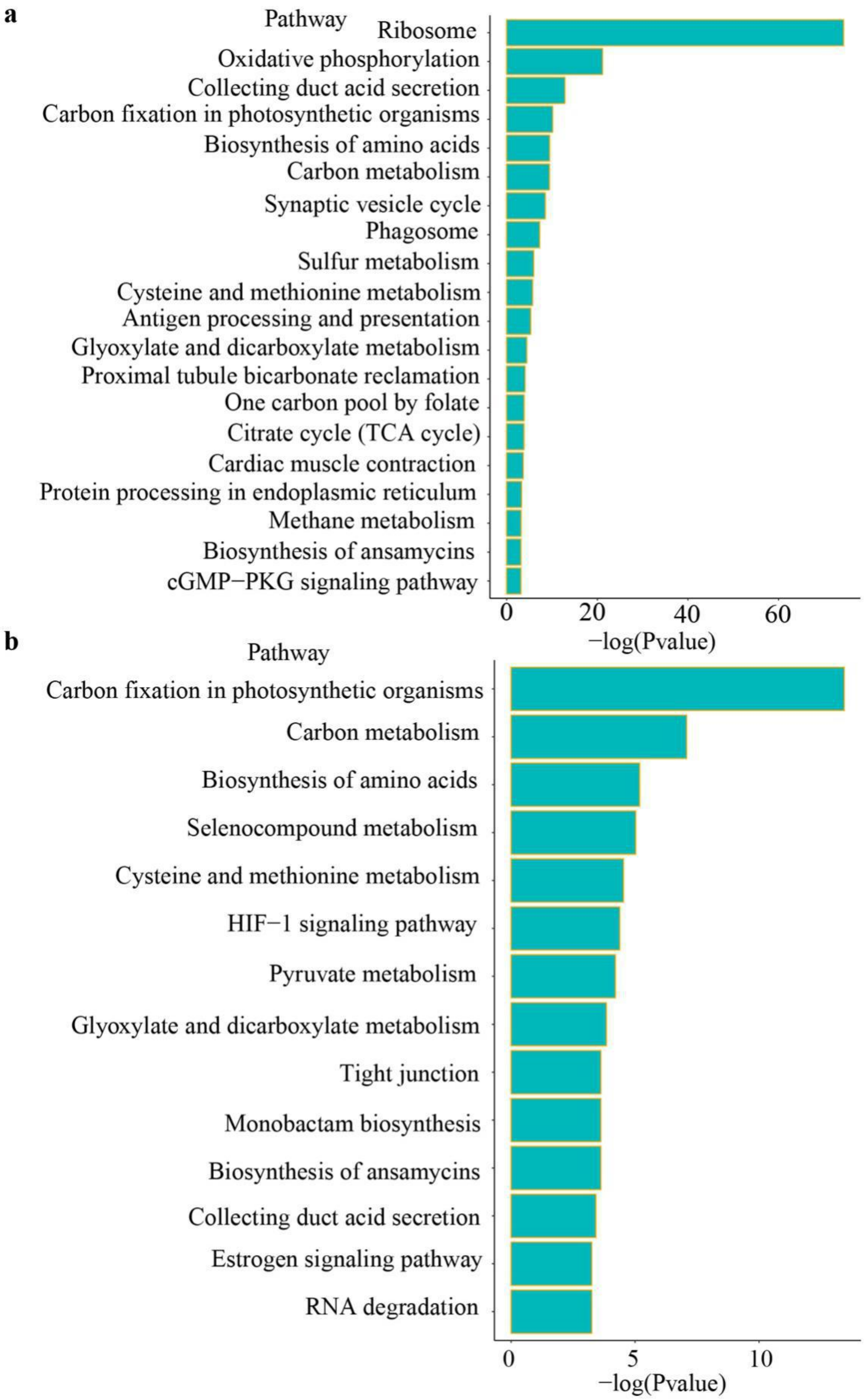

Figure S20. The KEGG enrichment of 263 high expression genes (HEGs) (a) and 33 high abundance proteins (HAPs) (b) in female and male luminous organs of both *L. yunnana* and *A. terminalis* (P < 0.05)**.** R package was used for the visualization of the images.

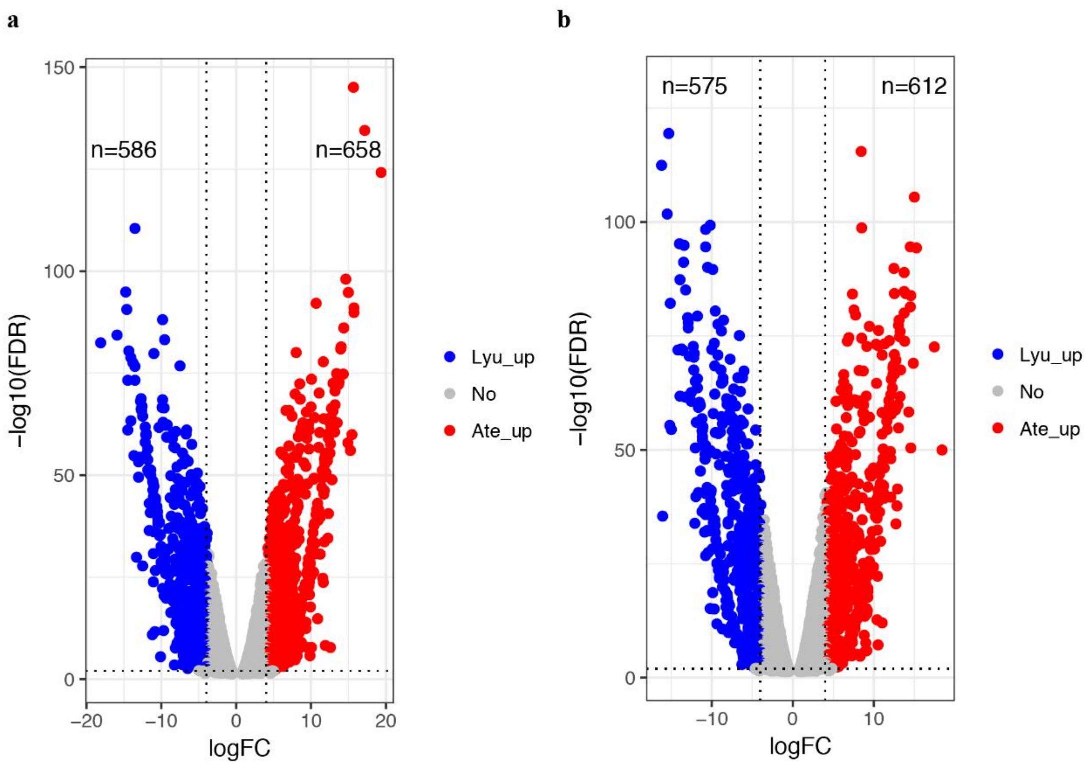

Figure S21. The differentially expressed genes (DEGs) in luminous organs between *L. yunnana* (Lyu) and *A. terminalis* (Ate). a shows comparison of female luminous organs (FO). **b** shows comparison of male luminous organs (MO). The broken lines indicate that the absolute value of log-fold change (logFC) more than 4 and false discovery rate (FDR) less than 0.01. Blue blots indicate up-regulated genes in *L. yunnana* and red blots indicate up-regulated genes in *A. terminalis*. n shows gene numbers. R package was used for the visualization of the images.

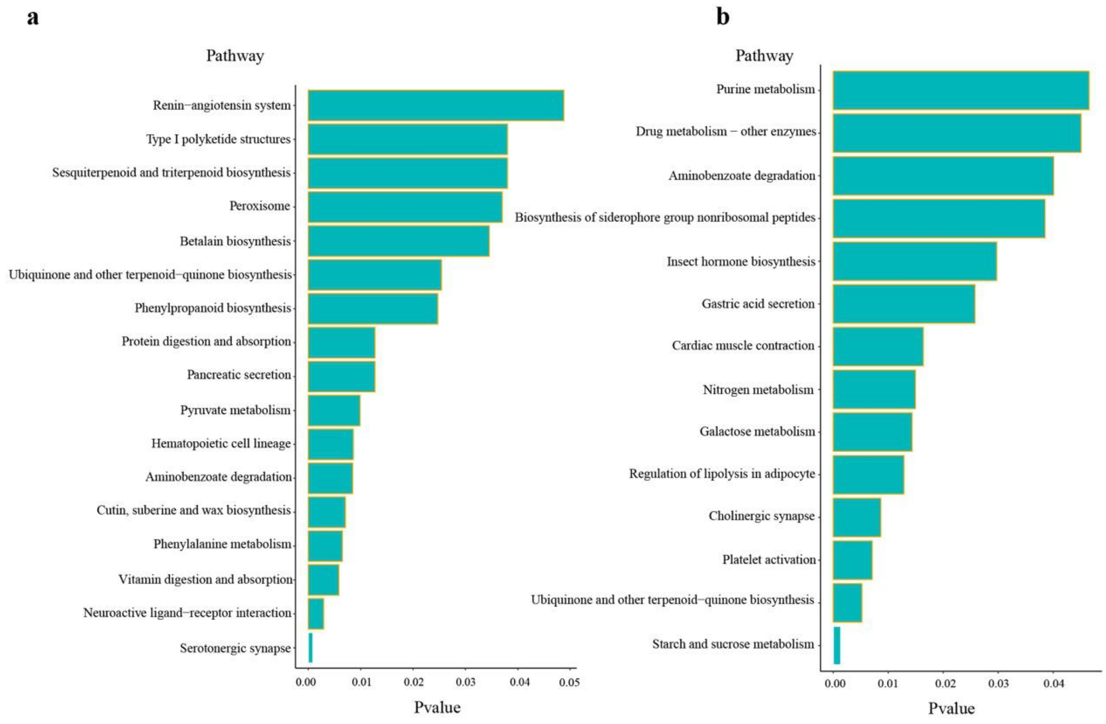

Figure S22. The KEGG enrichment of female luminous organs (FO) and male luminous organs (MO) overlapped genes from interspecies differentially expressed genes (DEGs) between *L. yunnana* and *A. terminalis* (P<0.05). a represents 388 up-regulated genes in *L. yunnana*. **b** represents 413 up regulated genes in *A. terminalis*. R package was used for the visualization of the images.

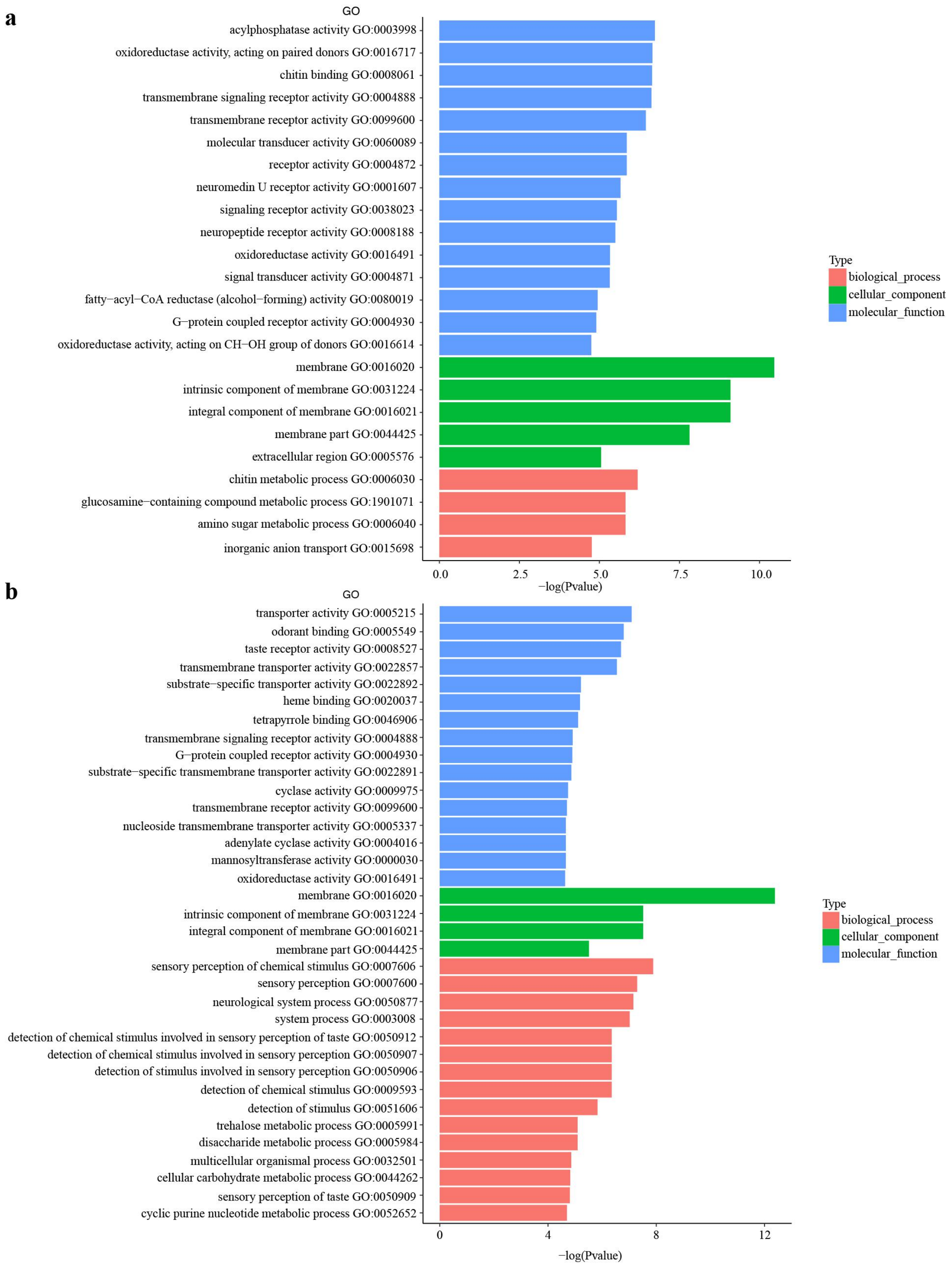

Figure S23. The GO enrichment of female luminous organs (FO) and male luminous organs (MO) overlapped genes from interspecies differentially expressed genes (DEGs) between *L. yunnana* and *A. terminalis* (P<0.01). **a** represents 388 up-regulated genes in *L. yunnana*. **b** represents 413 up regulated genes in *A. terminalis*. R package was used for the visualization of the images.

a

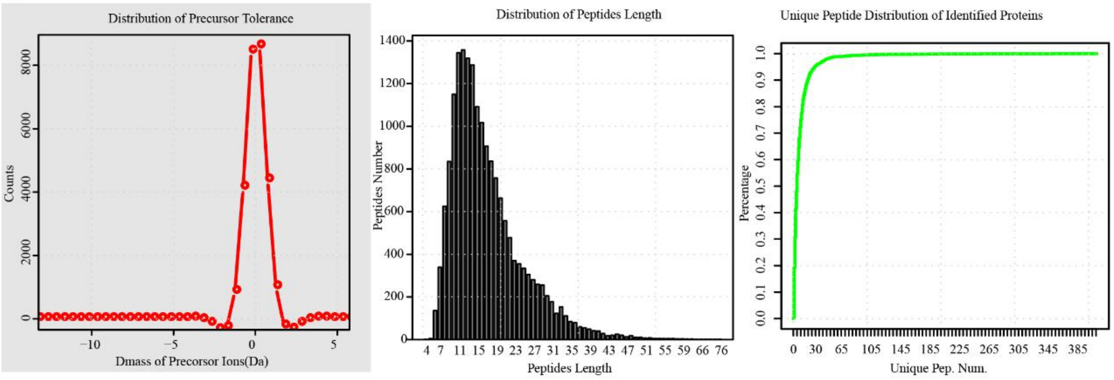

b

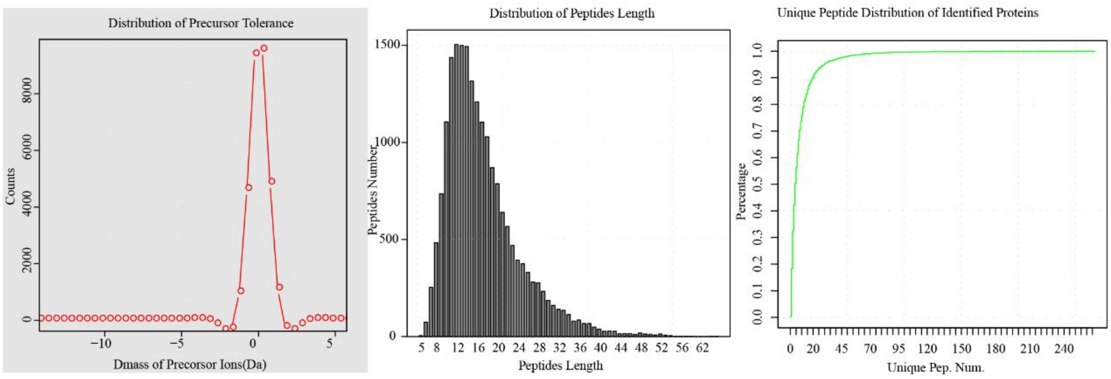

Figure S24. The characteristics of the identified peptides and protein groups in *L. yunnana* (a) and *A. terminalis* (b).

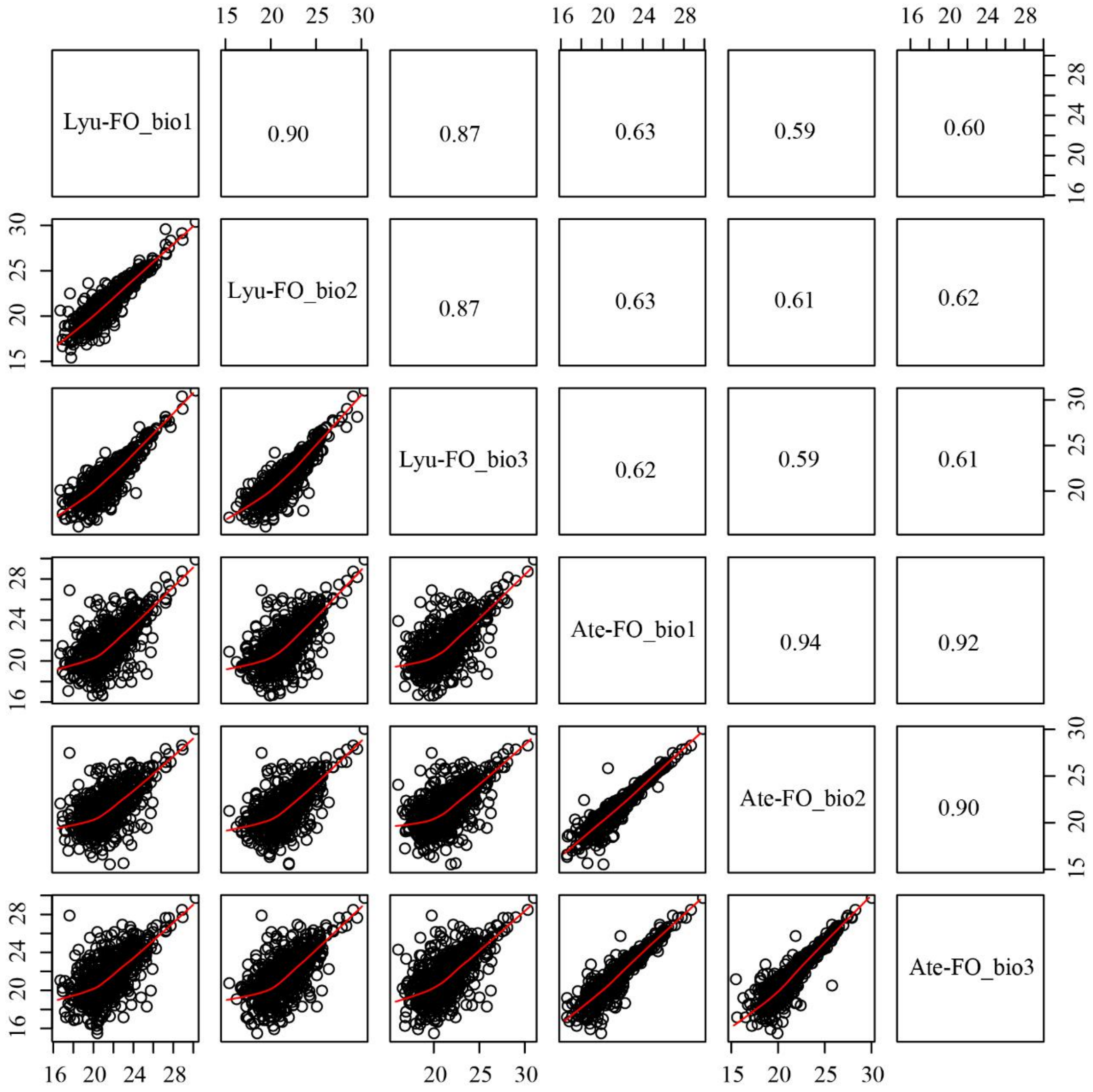

Figure S25. The correlation of quantified proteins between female luminous organs (FO) of *L. yunnana* (Lyu) and female luminous organs (FO) of *A. terminalis* (Ate). The numbers in square show correlation coefficient.

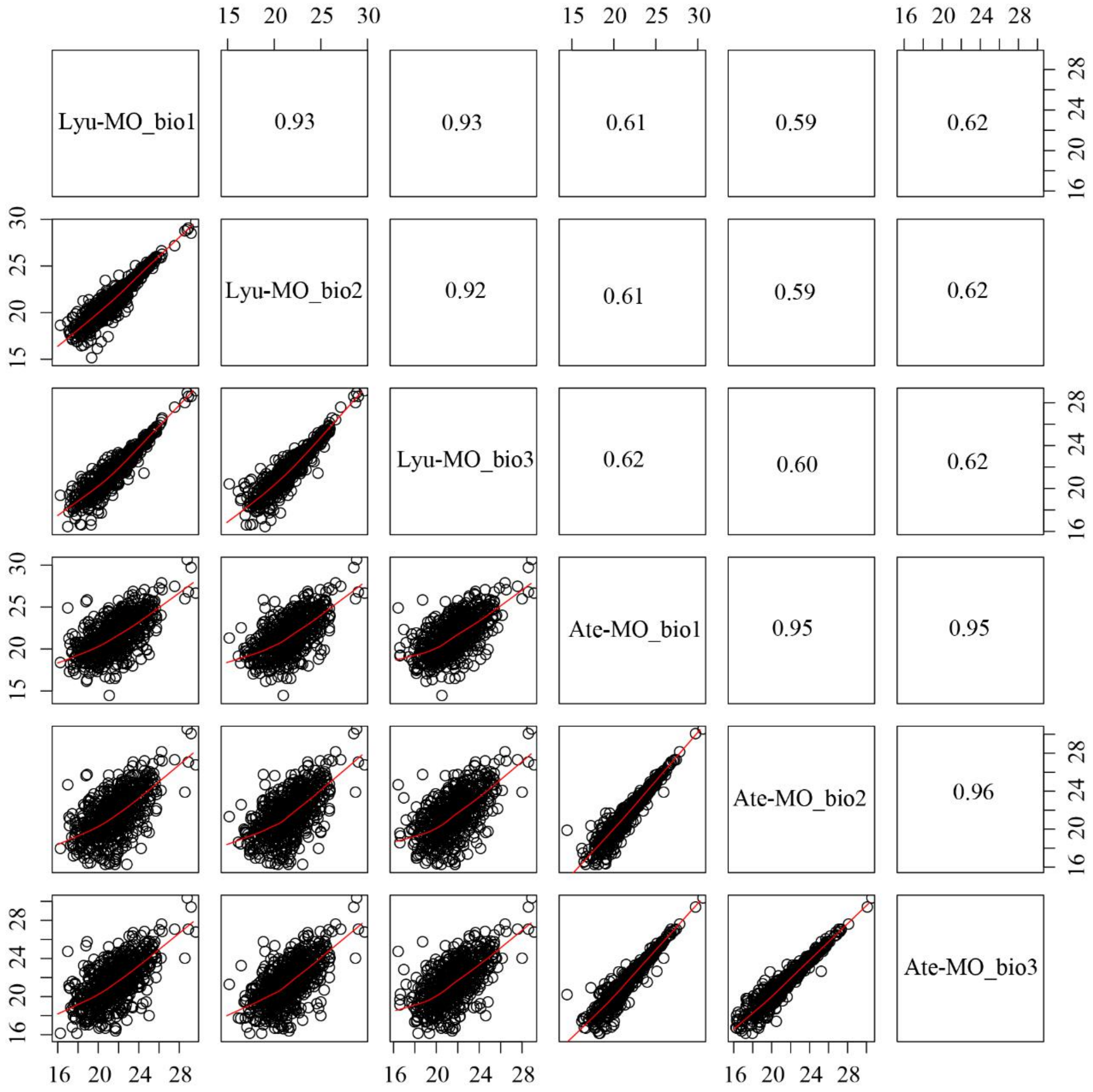

Figure S26. The correlation of quantified proteins between male luminous organs (MO) of *L. yunnana* (Lyu) and female luminous organs (MO) of *A. terminalis* (Ate). The numbers in square show correlation coefficient.

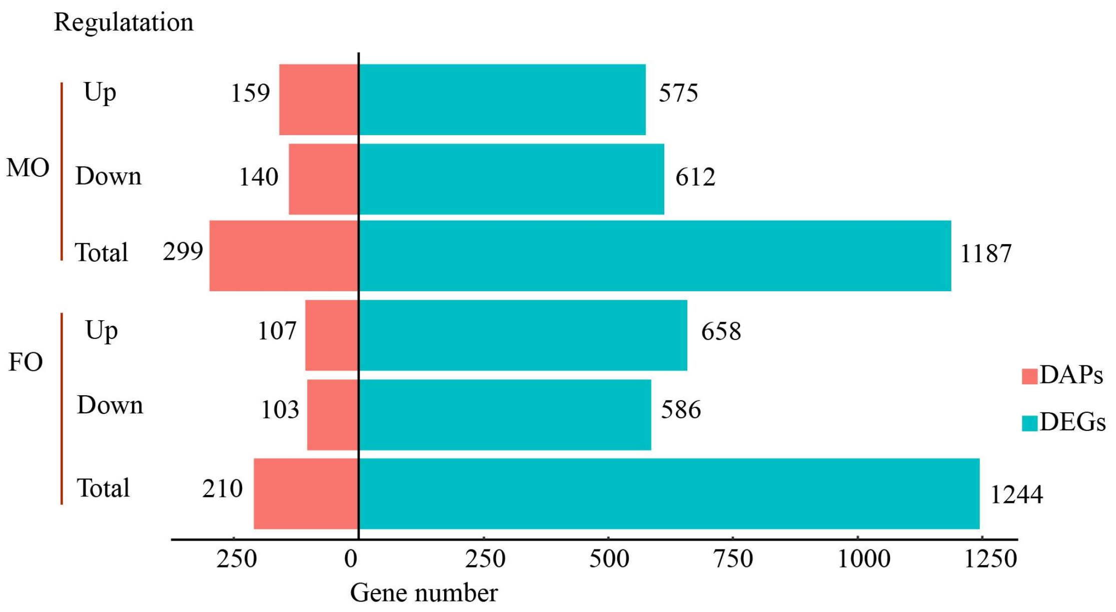

Figure S27. Differentially expressed genes (DEGs) and differentially abundant proteins (DAPs) identified in interspecies comparison. FO shows comparison of female luminous organs between *A. terminalis* and *L. yunnana*. MO shows comparison of male luminous organs between *A. terminalis* and *L. yunnana*. The up and down were referred to *A. terminalis*. R package was used for the visualization of the images.

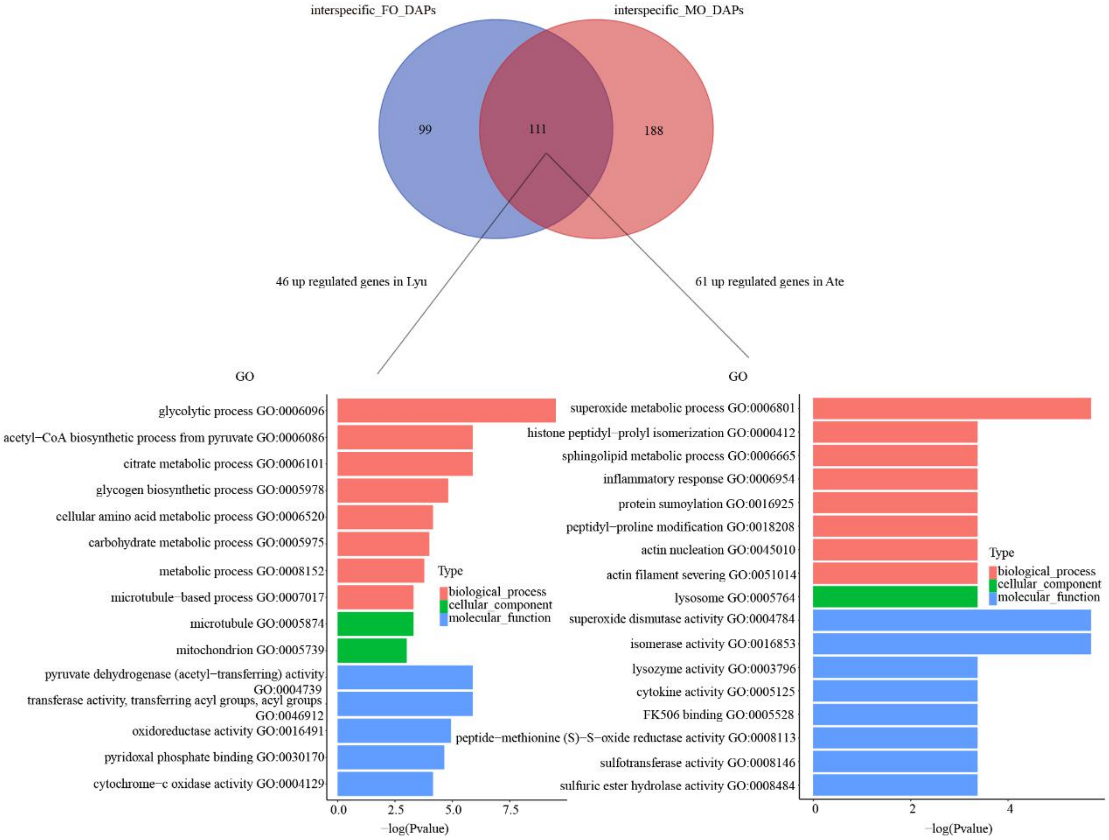

Figure S28. The GO enrichment of female luminous organ (FO) and male luminous organ (MO) overlapping genes from interspecies differentially abundant proteins (DAPs) between *L. yunnana* (Lyu) and *A. terminalis* (Ate) ($P < 0.05$). Left represents *L. yunnana* and right represents *A. terminalis*. R package was used for the visualization of the images.

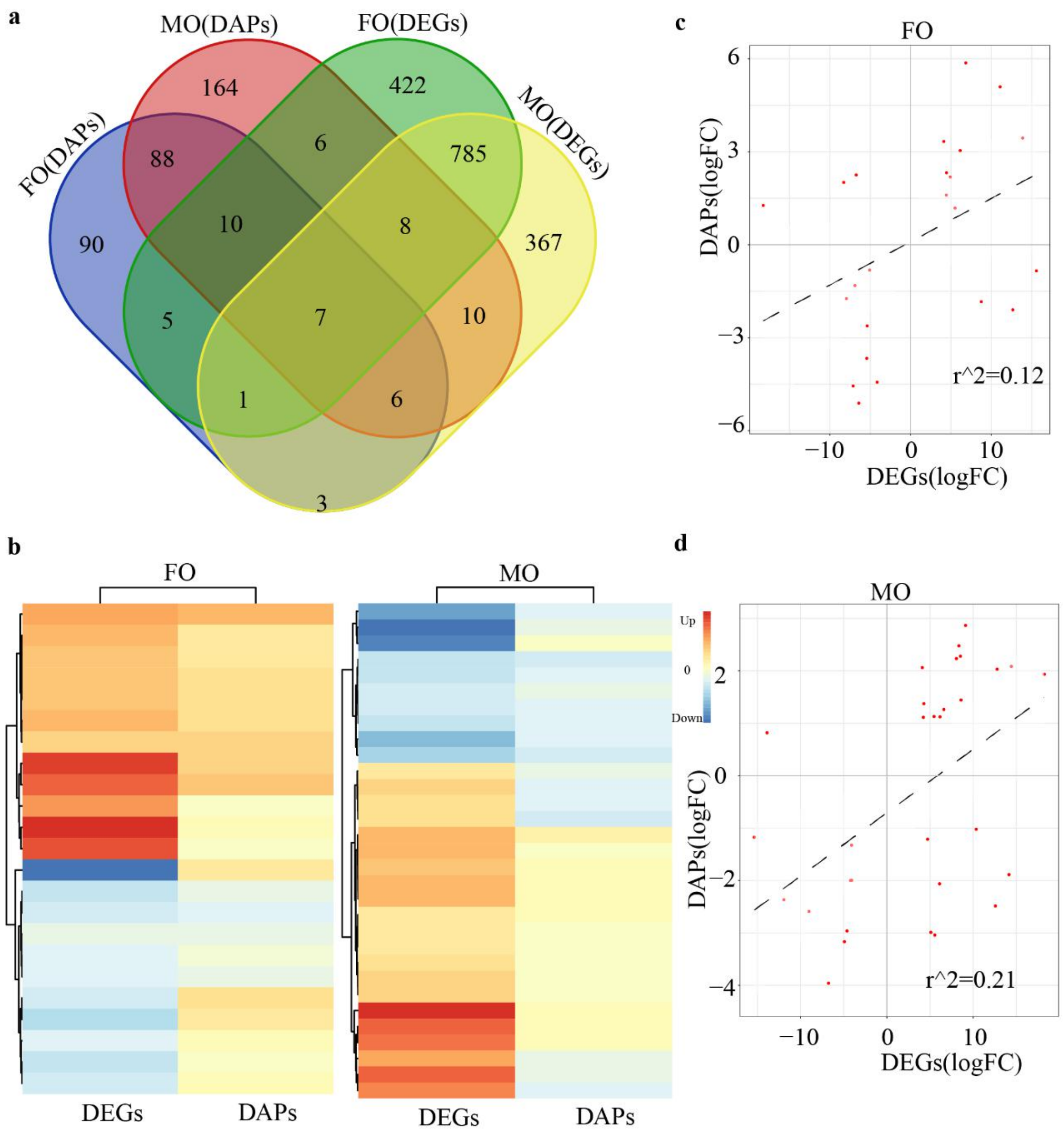

Figure S29. Protein abundance and transcriptomic level in different interspecies comparisons. a represents venn diagram of differentially expressed genes (DEGs) and differentially abundant proteins (DAPs). **b** represents comparison of log FC-change (logFC) in transcriptomic and proteins levels of DEGs and DAPs. **c** represents correlations between transcriptomic level and protein abundance of DEGs and DAPs in female luminous organs (FO). **d** represents correlations between transcriptomic level and protein abundance of DEGs and DAPs in male luminous organs (MO). The Calculate and draw custom Venn diagrams (http://bioinformatics.psb.ugent.be/webtools/Venn/) and R package were used for the visualization of the images.

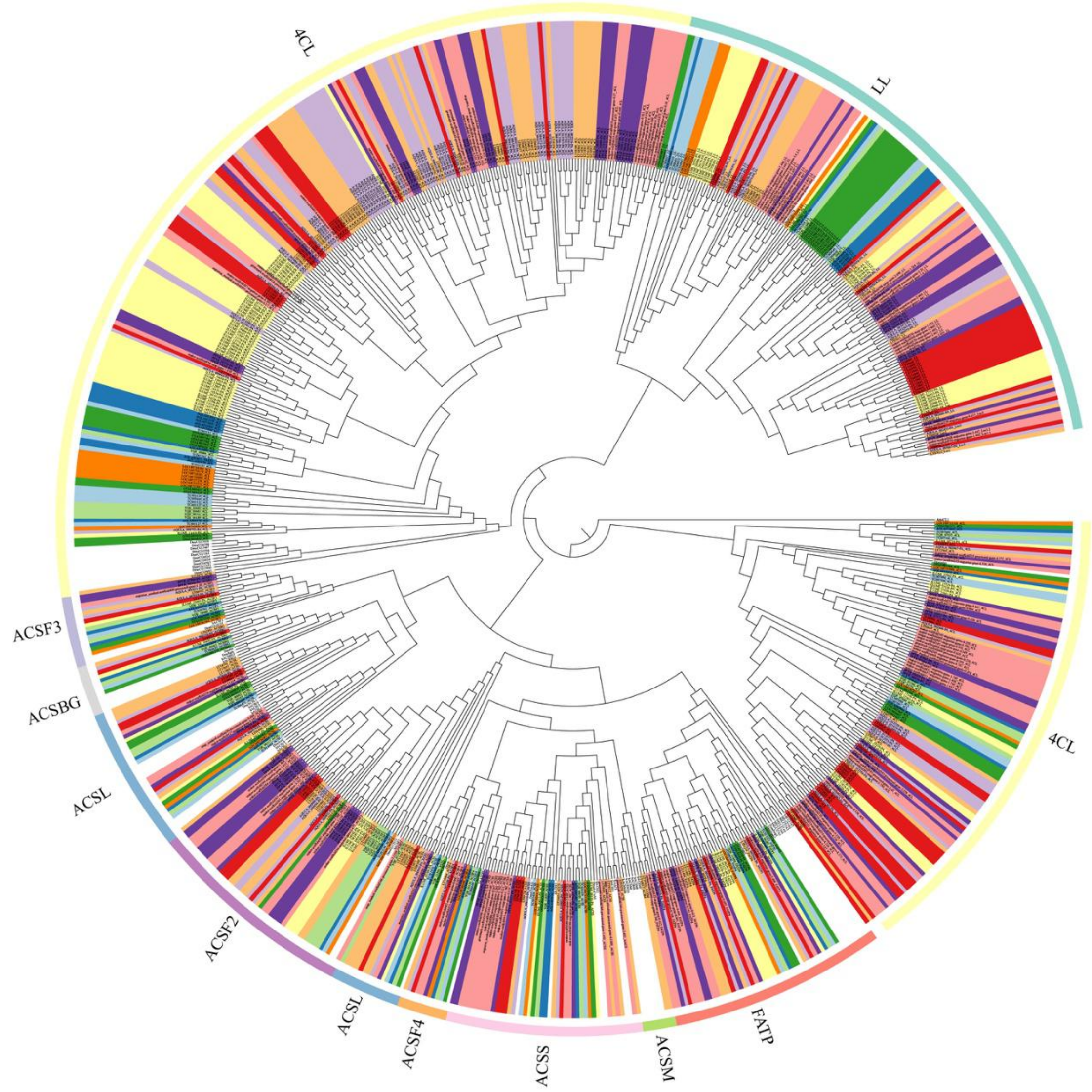

Figure S30. The maximum likelihood (ML) tree and classification of acyl-CoA synthetase (ACS) superfamily in beetles and fruit fly with *Arabidopsis thaliana* 4-coumarate: CoA ligase (Ath4CL1) as an outgroup. LL (luciferase-like), 4CL (4-coumarate:CoA ligases), ACSS (ACS short-chain family), ACSM (ACS medium-chain family), ACSBG (ACS bubblegum family), FATP (ACSVL, very long-chain ACS), ACSL (ACS long-chain family) and other families (ACSF2, ACS family member 2; ACSF3, ACS family member 3; AASDH (ACSF4)-aminoadipate-semialdehyde dehydrogenase). Lyu: *Lamprigera yunnana*; Ate: *Abscondita terminalis*; Ala: *Aquatica lateralis*; Ppy: *Photinus pyralis*; Ppe: *Pyrocoelia pectoralis*; Ilu: *Ignelater luminosus*; Apl: *Agrilus planipennis*; Ota: *Onthophagus taurus*; Agl: *Anoplophora glabripennis*; Dpo: *Dendroctonus ponderosae*; Tca: *Tribolium castaneum*; Dme: *Drosophila melanogaster*.

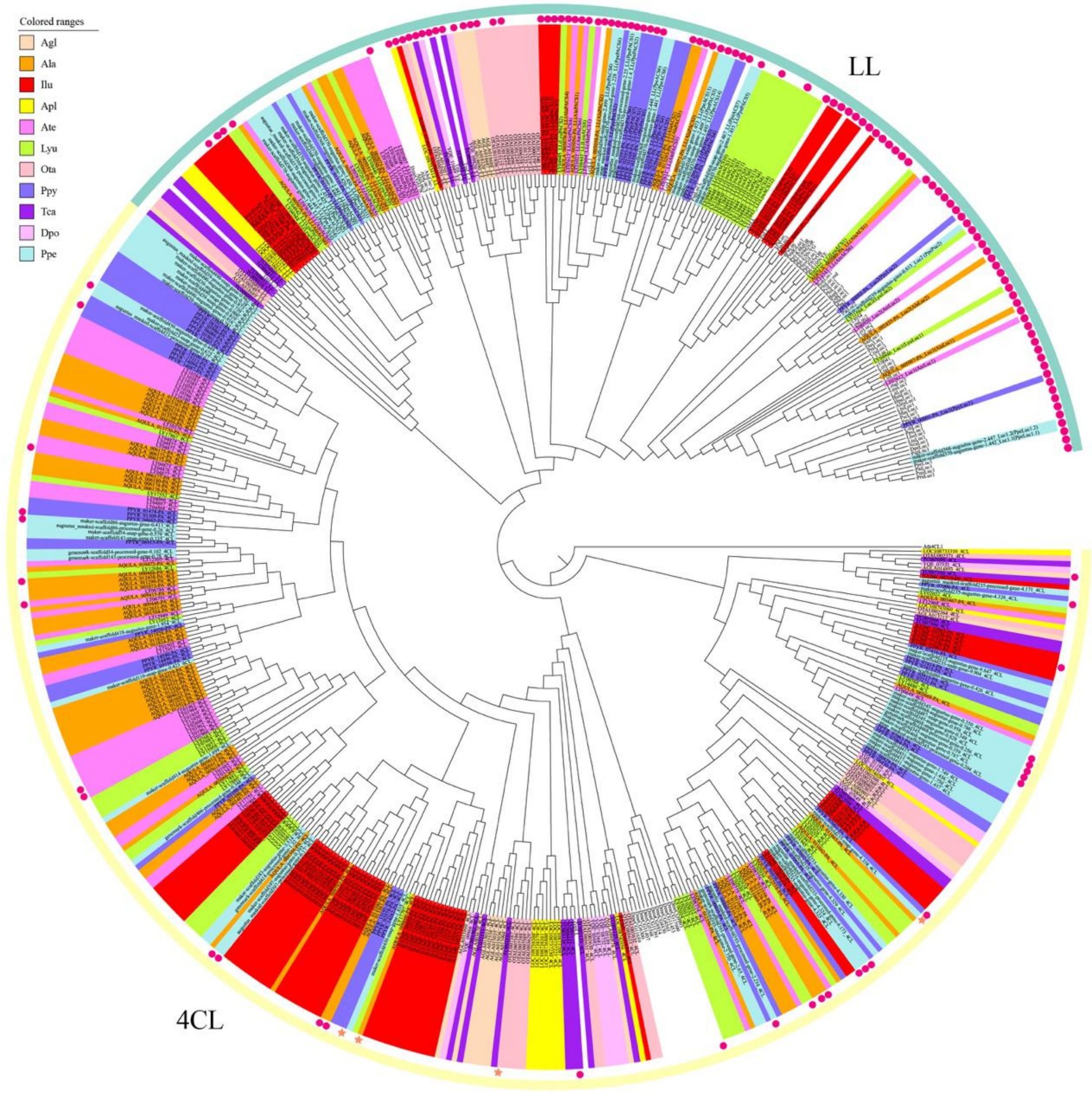

Figure S31. The maximum likelihood (ML) tree of 4CL (4-coumarate: CoA ligases) and LL (luciferase-like) in beetles and fruit fly and cloned luciferase genes with *Arabidopsis thaliana* 4-coumarate: CoA ligase (Ath4CL1) as the outgroup. Red circles represent PTS1 (the C-terminal peroxisomal targeting signal 1) and stars represent PTS2 (the N-terminal peroxisomal targeting signal 2). The cloned luciferase genes see Supplementary Data 21. Lyu: *Lamprigera yunnana*; Ate: *Abscondita terminalis*; Ala: *Aquatica lateralis*; Ppy: *Photinus pyralis*; Ppe: *Pyrocoelia pectoralis*; Ilu: *Ignelater luminosus*; Apl: *Agrilus planipennis*; Ota: *Onthophagus taurus*; Agl: *Anoplophora glabripennis*; Dpo: *Dendroctonus ponderosae*; Tca: *Tribolium castaneum*; Dme: *Drosophila melanogaster*.

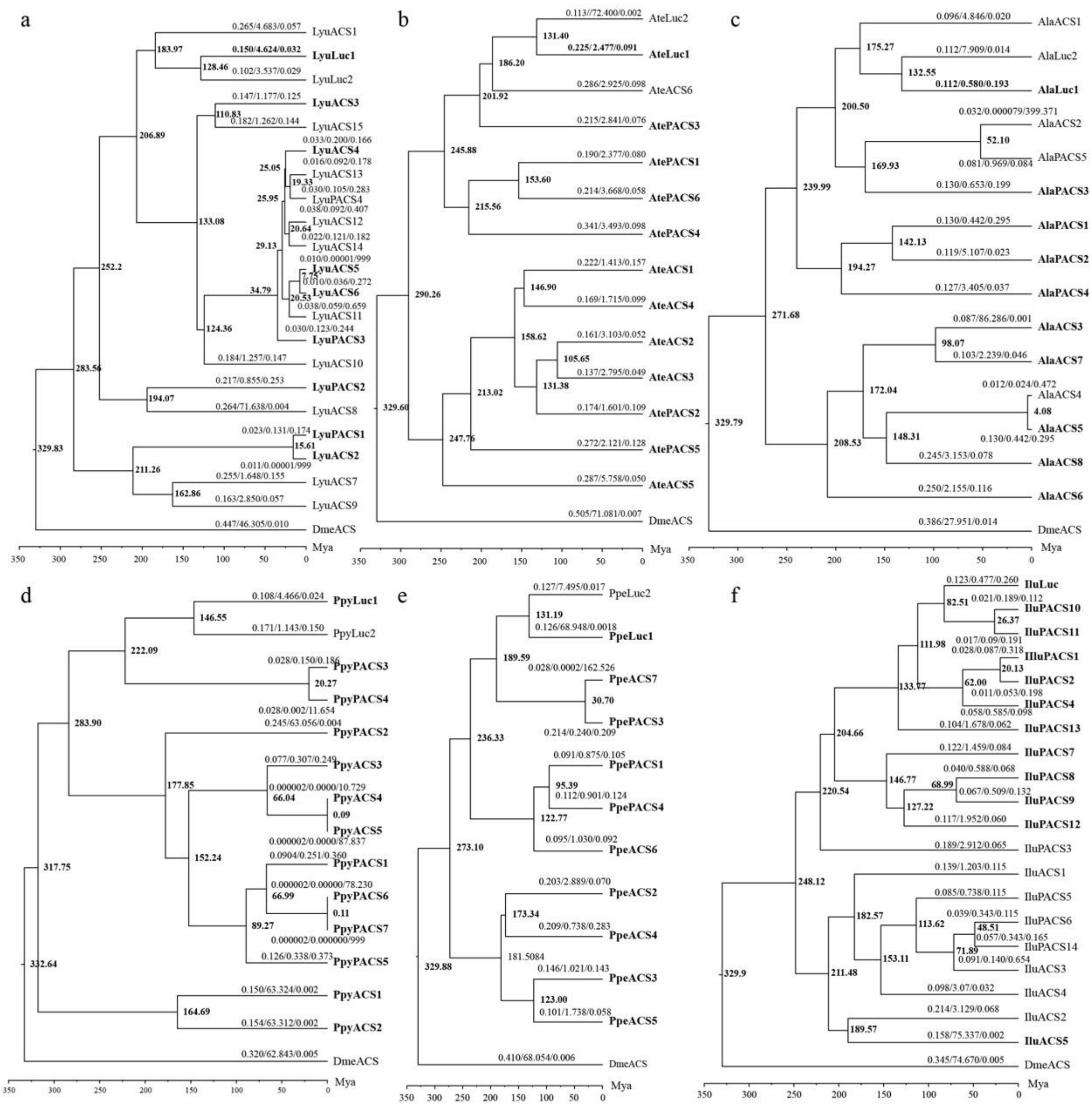

Figure S32. The divergence time, *Ka*, *Ks*, and *Ka/Ks* of LL (luciferase-like) family genes in intraspecies of six luminous species. a-f represent *L. yunnana* (Lyu) (**a**), *A. terminalis* (Ate) (**b**), *A. lateralis* (Ala) (**c**), *P. pyralis* (Ppy) (**d**), *P. pectoralis* (Ppe) (**e**) and *I. luminosus* (Ilu) (**f**), respectively. The divergence time was calculated using BEAST and calibrated with the divergence time (~330 mya) of beetle and fruit fly (*D. melanoganster*: Dme) as substitute for that of beetle LL and DmeACS. The *Ka*, *Ks*, and *Ka*/*Ks* were calculated roughly in six luminous species using the Codeml program of PAML with the free ratio model for each branch. The block highlighted genes from the tandem duplication (Luc1 and ACS/PACS). The abbreviations for the LL see Supplementary Data 23.

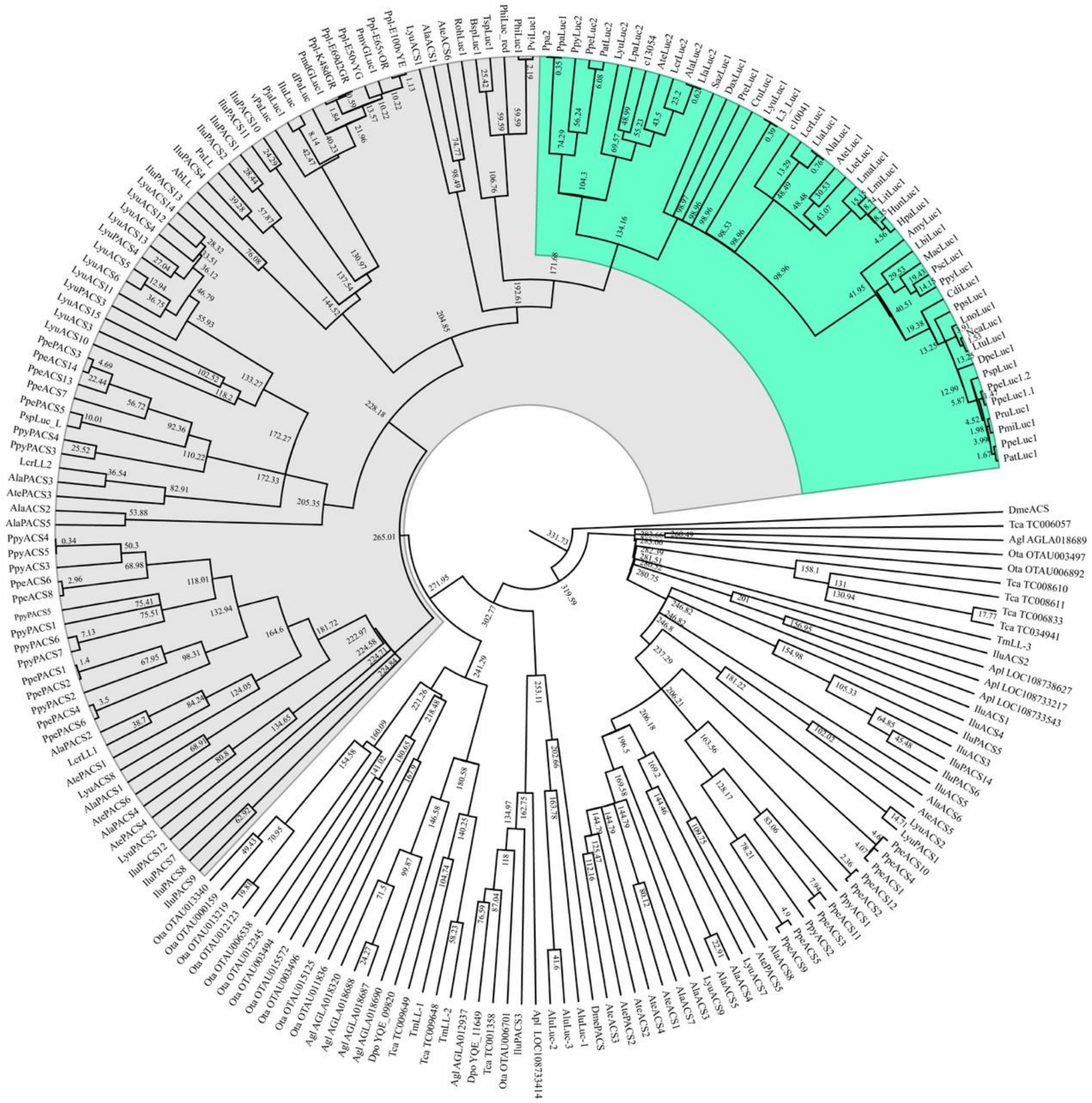

Figure S33. The divergence time of LL (luciferase-like) family genes in 11 beetle species and fruit fly (unit: million years (mya)). The divergence time was calculated using BEAST and calibrated with the divergence time (~330 mya) of beetle and fruit fly (*D. melanoganster*: Dme) as substitute for that of beetle LL and DmeACS. Lyu: *L. yunnana*; Ate: *A. terminalis*; Ala: *A. lateralis*, Ppy: *P. pyralis*; Ppe: *P. pectoralis*; Ilu: *I. luminosus*; Apl: *A. planipennis*; Ota: *O. taurus*; Agl: *A. glabripennis*; Dpo: *D. ponderosae*; Tca: *T. castaneum*; Dme: *D. melanogaster*. The abbreviations for the LL see Supplementary Data 23.

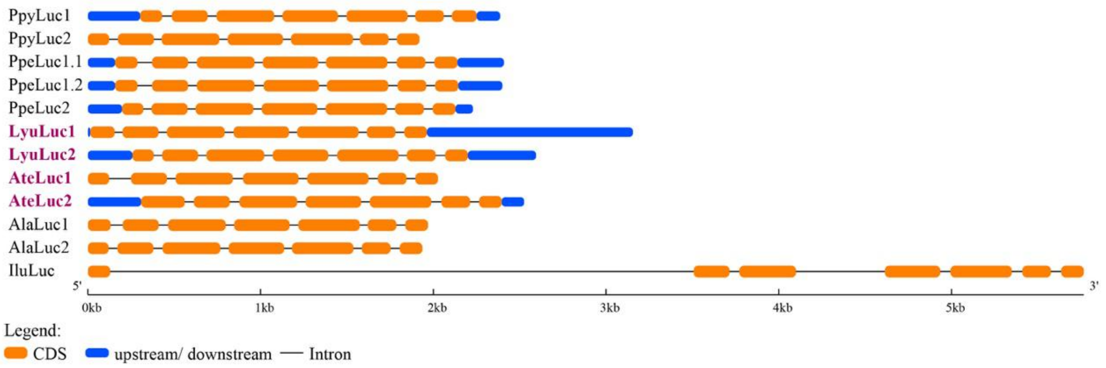

Figure S34. Gene structure of luciferase genes (Luc1 and Luc2). Lyu: *L. yunnanna*; *A. terminalis*; Ala: *A. lateralis*; Ppy: *P. pyralis*; Ppe: *P. pectoralis*; Ilu: *I. luminosus*. The gene structure was created using the Gene Structure Display Server 2.0 (http://gsds.cbi.pku.edu.cn)

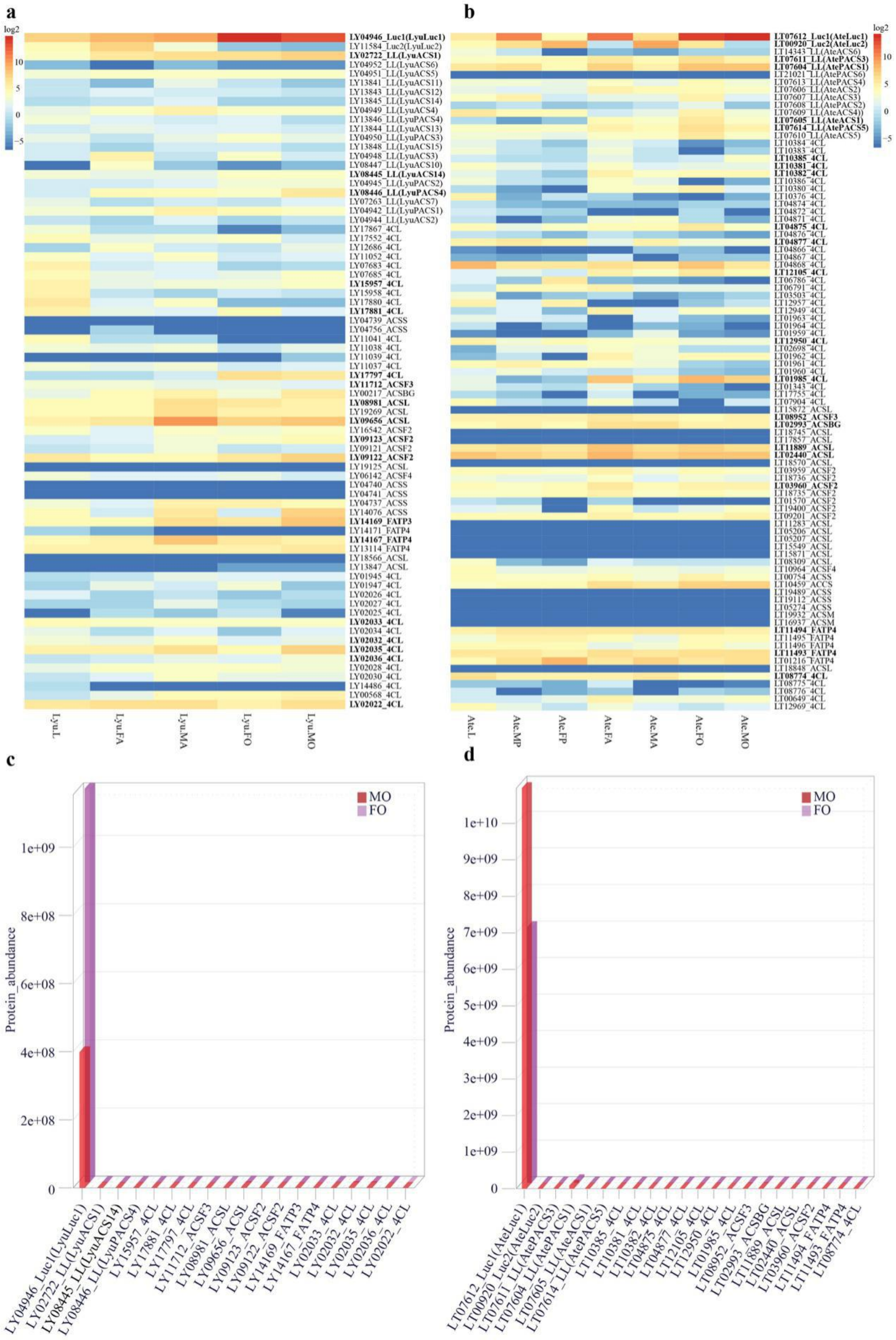

Figure S35. The expression of acyl-CoA synthetase (ACS) superfamily in the luminous organs of *L. yunnanna* (Lyu, LY) and *A. terminalis* (Ate, LT) at transcriptomic and proteomic levels. a and **b** show the heat map of log2-scaled

fragments per kilobase of transcript per million fragments mapped (FPKM) in *L. yunnanna* and *A. terminalis*, respectively. Those genes with abundance at proteomic level were shown as bold. **c** and **d** showed the expression at the proteomic level in *L. yunnanna* and *A. terminalis*, respectively. MO: luminous organ of male adult; FO: luminous organ of female adult; MA: male adult; FA: female adult; MP: male pupa; FP: female pupa; L: larva. R package was used for the visualization of the images.

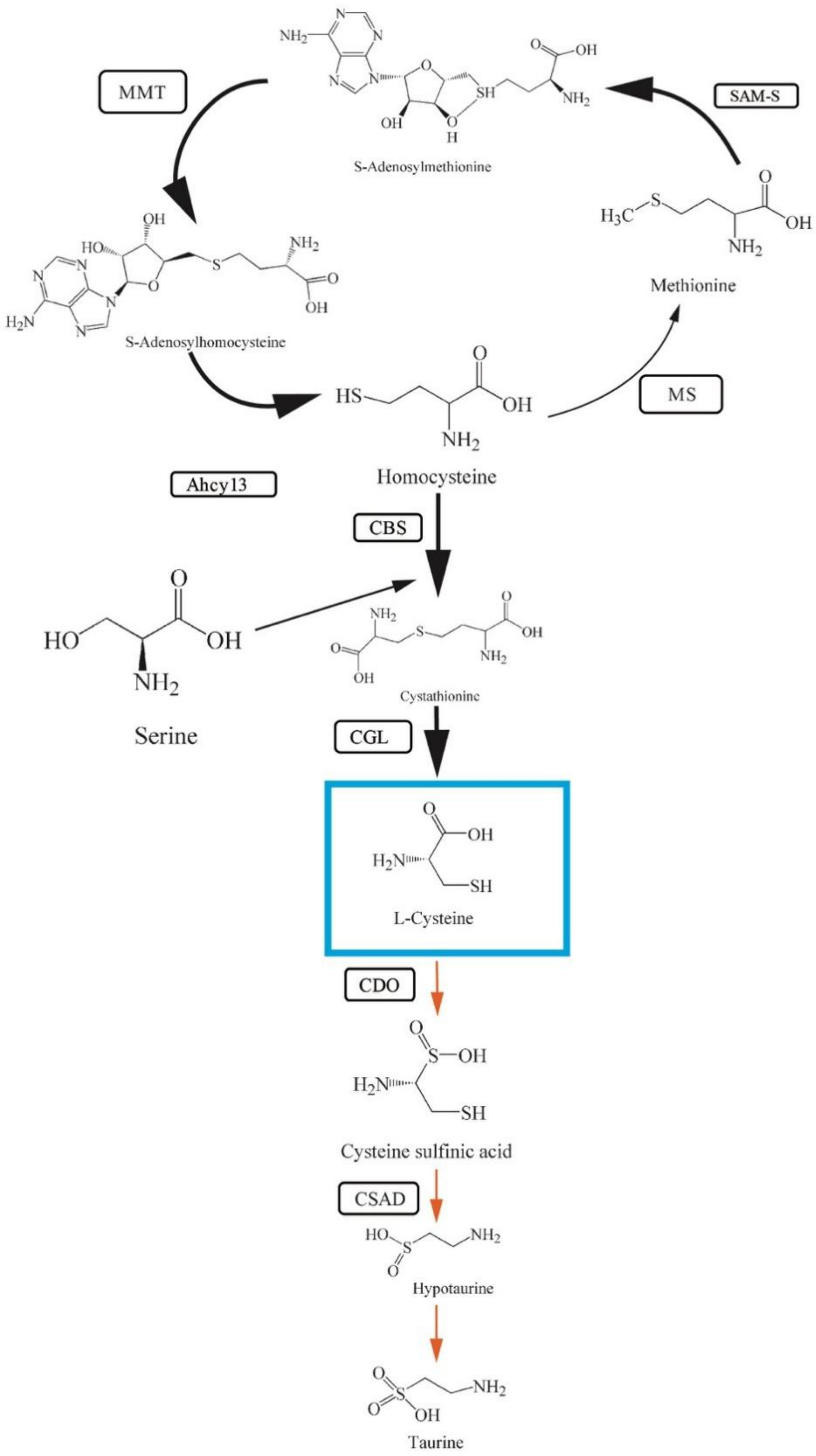

Figure S36. The cysteine metabolism pathway from methionine. Bold black arrows denote anabolism and red arrows denote catabolism. L-cysteine marked using the blue box is the precursor of luciferin biosynthesis. SAM-S: S-adenosylmethionine; MMT: methyltransferases; Ahcy13: adenosylhomocysteinase; MS: methionine synthase; CBS: cystathionine-b-synthase; CGL: cystathionine gamma-lyase; CDO: cysteine dioxygenase; CSAD: cysteine sulfinate decarboxylase.

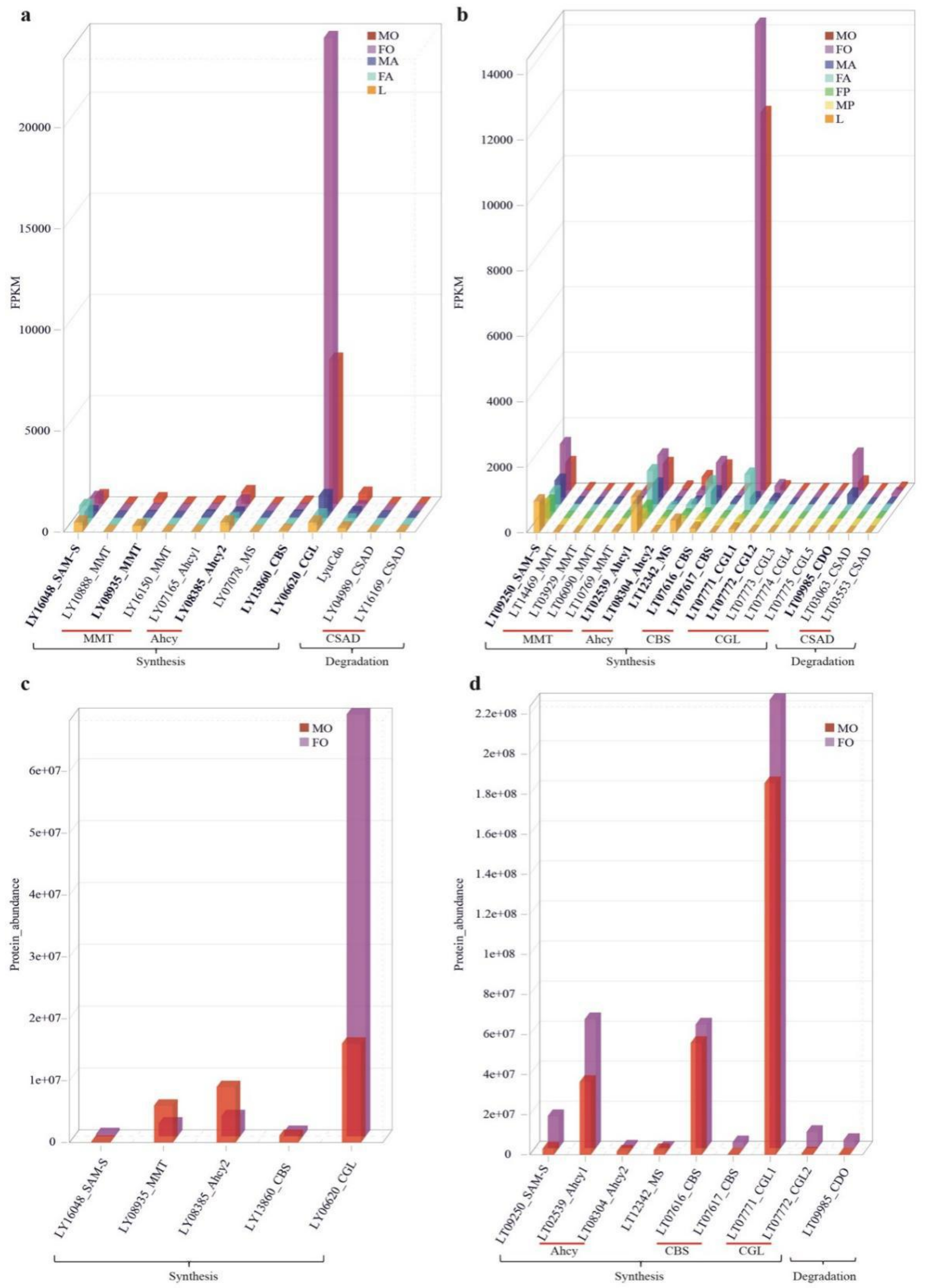

Figure S37. The expression of genes of cysteine metabolism pathway in *L. yunnana* and *A. terminalis* at transcriptomic and proteomic levels. a and **b** showed the expression of the genes at transcriptomic level in *L. yunnana* and *A. terminalis*, respectively. Those genes with abundance at proteomic level were shown as bold. **c** and **d** showed the expression of the genes identified at the proteomic level in *L. yunnana* and *A. terminalis*, respectively. MO: luminous organ of male adult; FO: luminous organ of female adult; MA: male adult; FA: female adult; MP: male pupa; FP: female pupa; L: larva. R package was used for the visualization of the images.

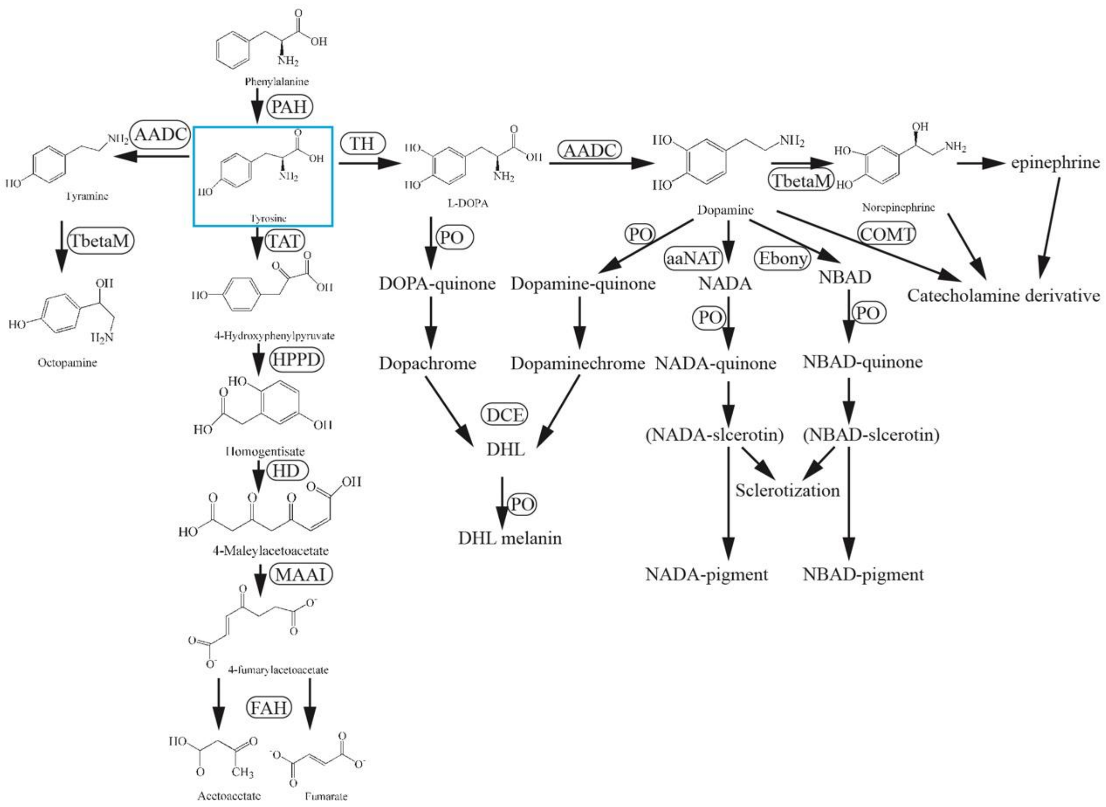

Figure S38. The tyrosine metabolism pathway. Tyrosine marked using the blue box is the candidate precursor of luciferin biosynthesis. PAH: phenylalanine hydroxylase; TAT: tyrosine aminotransferase; HPPD: 4-hydroxyphenylpyruvate dioxygenase; HD: homogentisate 1,2-dioxygenase; MAAI: maleylacetoacetate isomerase; FAH: fumarylacetoacetase; ADDC: aromatic L-amino acid decarboxylase; TβM: tyramine beta-monooxygenase; TH: tyrosine hydroxylase; DCE: dopachrome conversion enzyme; aaNAT: arylalkylamine N-acetyltransferase; Ebony: N-beta-alanyldopamine synthase; COMT: catechol-O-methyltransferase; PO: phenoloxidases.

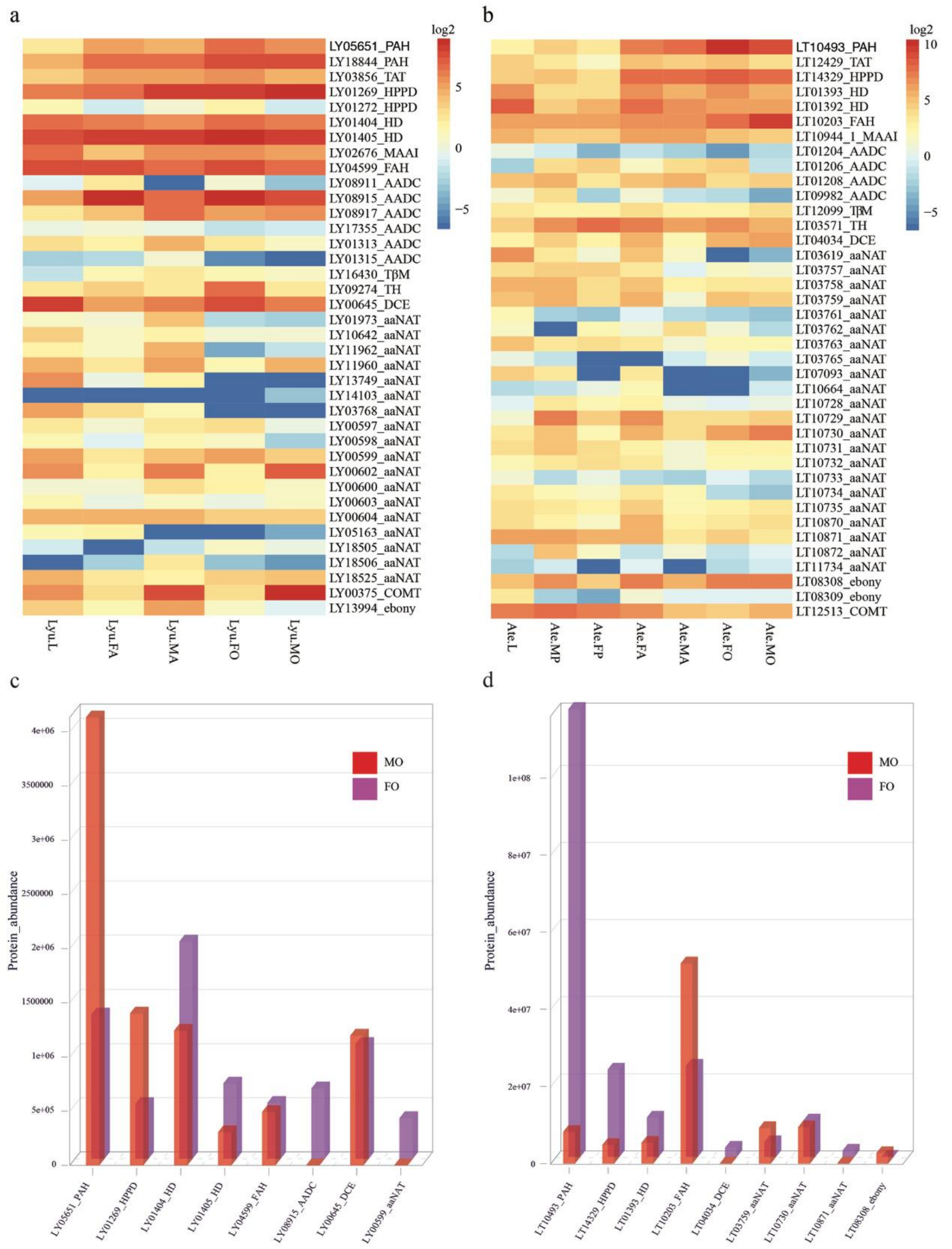

Figure S39. The expression of genes of tyrosine metabolism pathway in *L. yunnana* and *A. terminalis* at transcriptomic and proteomic levels. a and **b** showed log2-scaled fragments per kilobase of transcript per million fragments mapped (FPKM) at transcriptomic level in *L. yunnana* and *A. terminalis*, respectively. **c** and **d** showed the expression of the genes identified at the proteomic level in *L. yunnana* and *A. terminalis*, respectively. MO: luminous organ of male adult; FO: luminous organ of female adult; MA: male adult; FA: female adult; MP: male pupa; FP: female pupa; L: larva. R package was used for the visualization of the images.

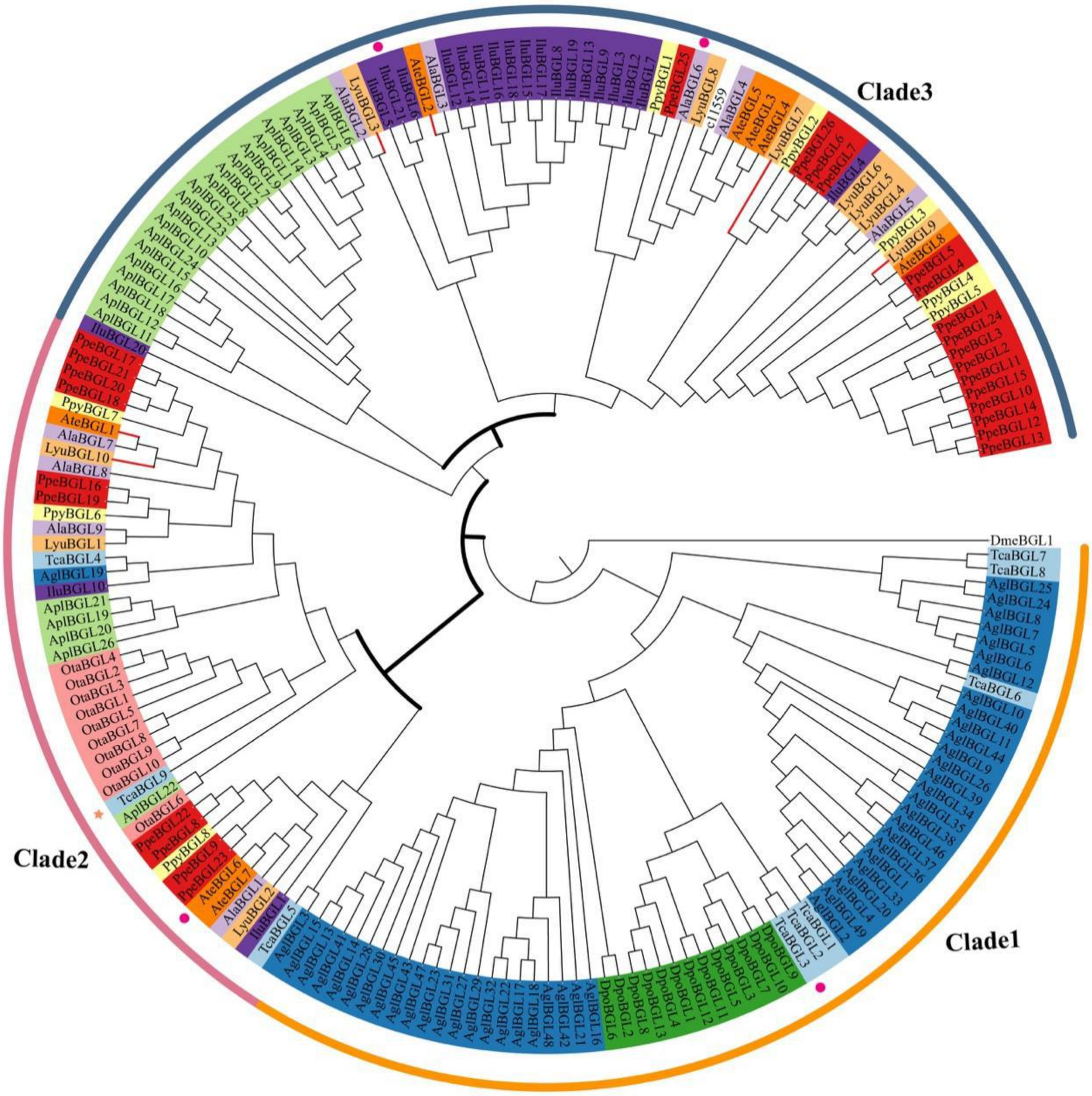

Figure S40. The phylogenetic tree of β-glucosidase (BGL) genes in beetles with fruit fly BGL1 as an outgroup. The beetle BGLs cluster into three clades (Clade1, Clade2 and Clade3). The red circles show those genes with the C-terminal peroxisomal targeting signal 1 (PTS1) and the pink stars show those gene with the N-terminal peroxisomal targeting signal 2 (PTS2). The lineages including potential candidates in luciferin biosynthesis are shown as bold and the candidates are shown as red clade. Lyu: *Lamprigera yunnana*; Ate: *Abscondita terminalis*; Ala: *Aquatica lateralis*; Ppy: *Photinus pyralis*; Ppe: *Pyrocoelia pectoralis*; Ilu: *Ignelater luminosus*; Apl: *Agrilus planipennis*; Ota: *Onthophagus taurus*; Agl: *Anoplophora glabripennis*; Dpo: *Dendroctonus ponderosae*; Tca: *Tribolium castaneum*; Dme: *Drosophila melanogaster*.

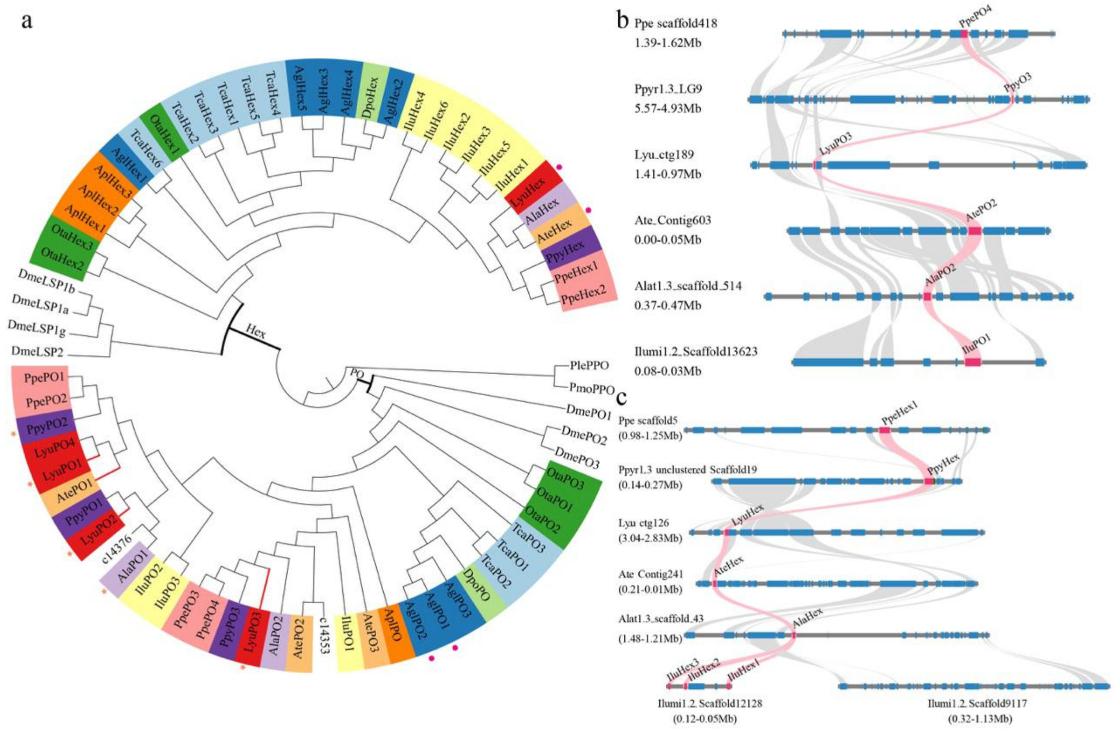

Figure S41. The evolution of hemocyanin superfamily (phenoloxidases (tyrosinase) (PO), hexamerin (Hex)) genes in beetles and fruit fly. a shows the phylogenetic tree of PO genes with POs of *Penaeus monodon* (PmoPPO) and *Pacifastacus leniusculus* (PlePPO) as outgroups. The red circles show those genes with the C-terminal peroxisomal targeting signal 1 (PTS1) and the pink stars show those gene with the N-terminal peroxisomal targeting signal 2 (PTS2). The candidates in luciferin biosynthesis are shown as red clade. **b** and **c** show the syntenic relationships of PO genes (**b**, highlighted in pink) and Hex genes (**c**, highlighted in pink) and their flanking genes surrounding up-200 kb and down-100 kb genomic regions among six luminous beetles, respectively. Lyu: *Lamprigera yunnana*; Ate: *Abscondita terminalis*; Ala: *Aquatica lateralis*; Ppy: *Photinus pyralis*; Ppe: *Pyrocoelia pectoralis*; Ilu: *Ignelater luminosus*; Apl: *Agrilus planipennis*; Ota: *Onthophagus taurus*; Agl: *Anoplophora glabripennis*; Dpo: *Dendroctonus ponderosae*; Tca: *Tribolium castaneum*; Dme: *Drosophila melanogaster*.

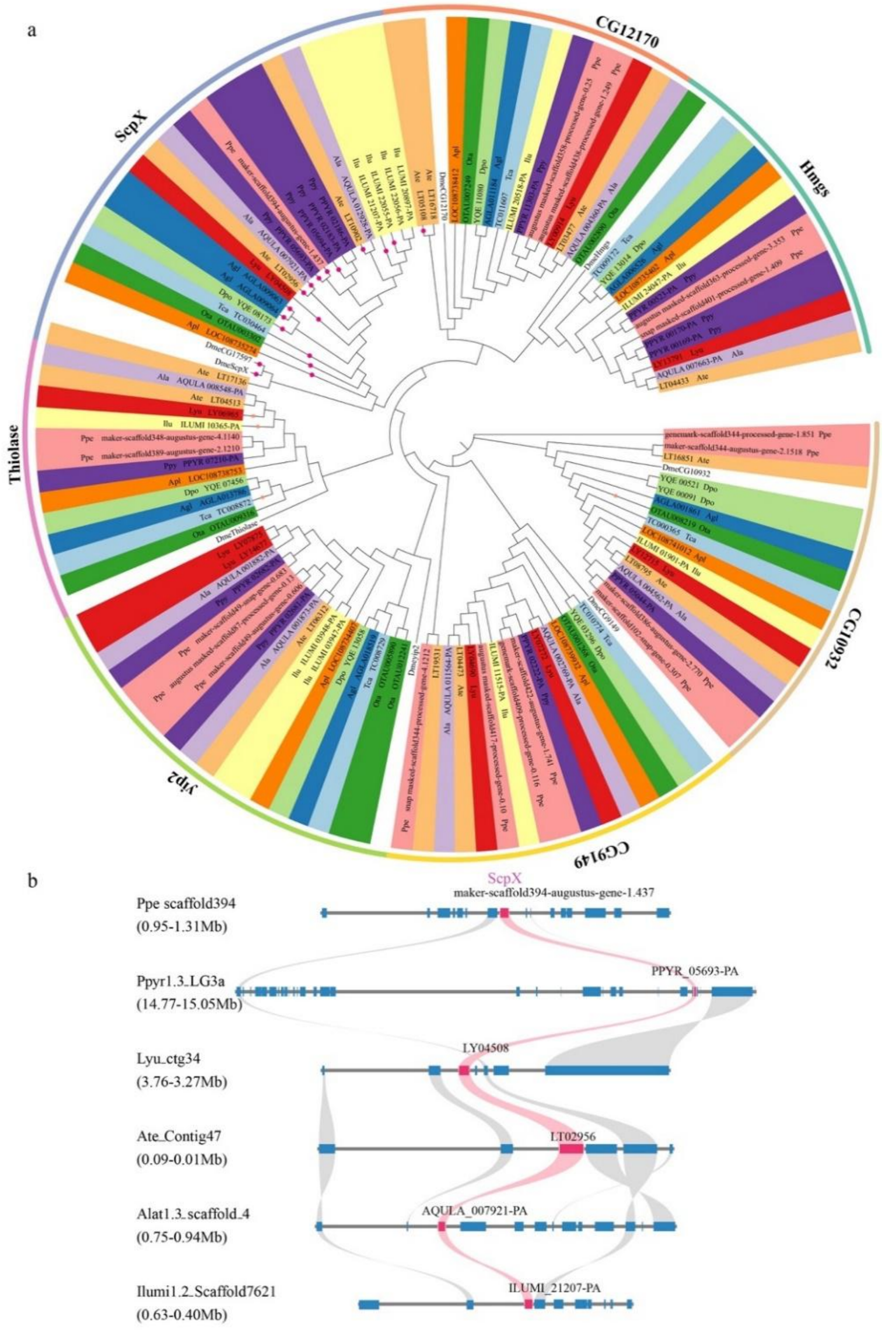

Figure S42. The evolution of thiolase gene family in beetles and fruit fly. a shows the phylogenetic tree. The red circles show those genes with the C-terminal peroxisomal targeting signal 1 (PTS1) and the pink stars show those gene with the N-terminal peroxisomal targeting signal 2 (PTS2). **b** shows the syntenic relationships of sterol carrier protein (ScpX) gene (**b**, highlighted in pink) and its flanking genes surrounding up-200kb and down-200kb genomic regions among six luminous beetles. Lyu: *Lamprigera yunnana*; Ate: *Abscondita terminalis*; Ala: *Aquatica lateralis*; Ppy: *Photinus pyralis*; Ppe: *Pyrocoelia pectoralis*; Ilu: *Ignelater luminosus*; Apl: *Agrilus planipennis*; Ota: *Onthophagus taurus*; Agl: *Anoplophora glabripennis*; Dpo: *Dendroctonus ponderosae*; Tca: *Tribolium castaneum*; Dme: *Drosophila melanogaster*.

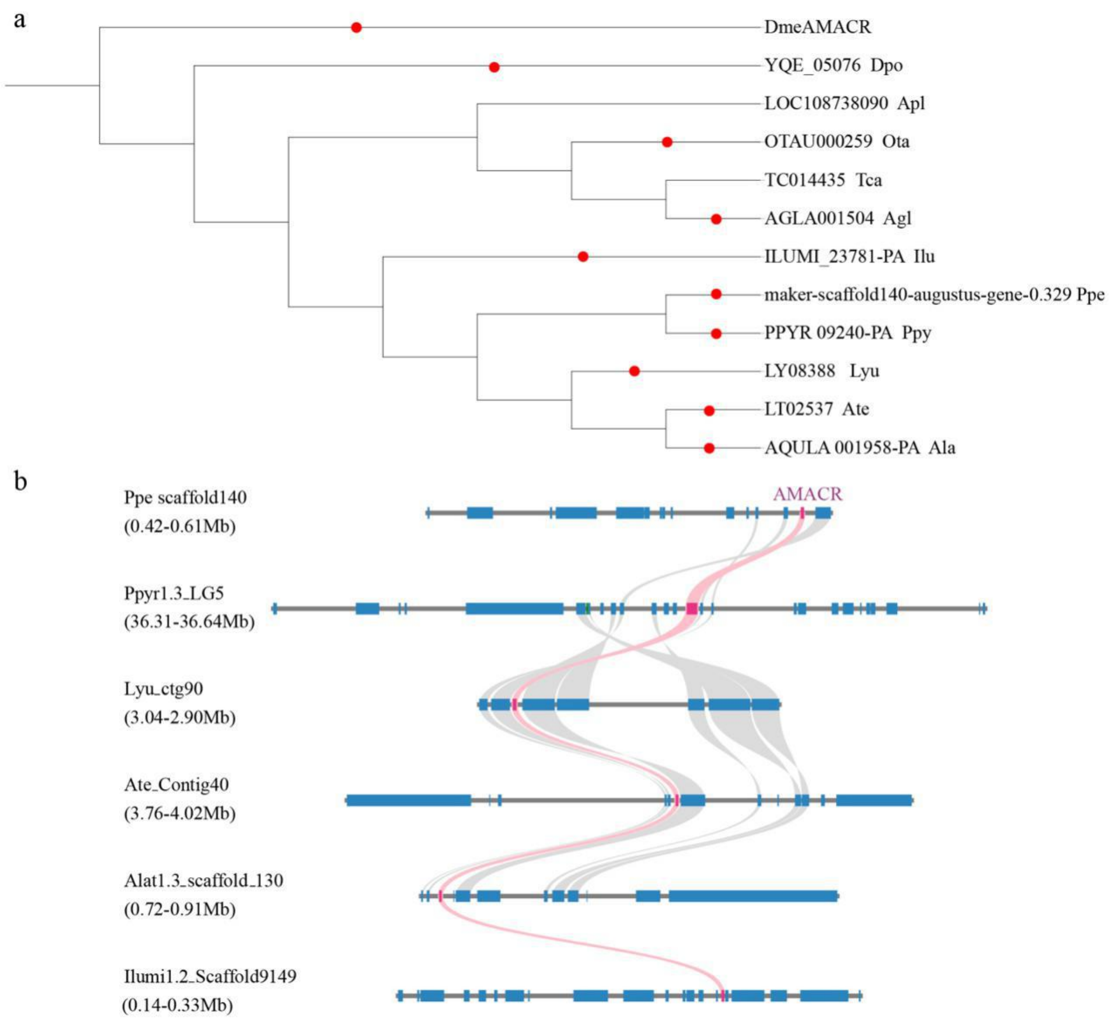

Figure S43. The evolution of alpha-methyl-acyl-CoA racemase (AMACR) family in beetles and fruit fly. a shows the phylogenetic tree. The red circles show those genes with the C-terminal peroxisomal targeting signal 1 (PTS1). **b** shows the syntenic relationships of AMACR gene (**b**, highlighted in pink) and its flanking genes surrounding up-200kb and down-200kb genomic regions among six luminous beetles. Lyu: *Lamprigera yunnana*; Ate: *Abscondita terminalis*; Ala: *Aquatica lateralis*; Ppy: *Photinus pyralis*; Ppe: *Pyrocoelia pectoralis*; Ilu: *Ignelater luminosus*; Apl: *Agrilus planipennis*; Ota: *Onthophagus taurus*; Agl: *Anoplophora glabripennis*; Dpo: *Dendroctonus ponderosae*; Tca: *Tribolium castaneum*; Dme: *Drosophila melanogaster*.

a

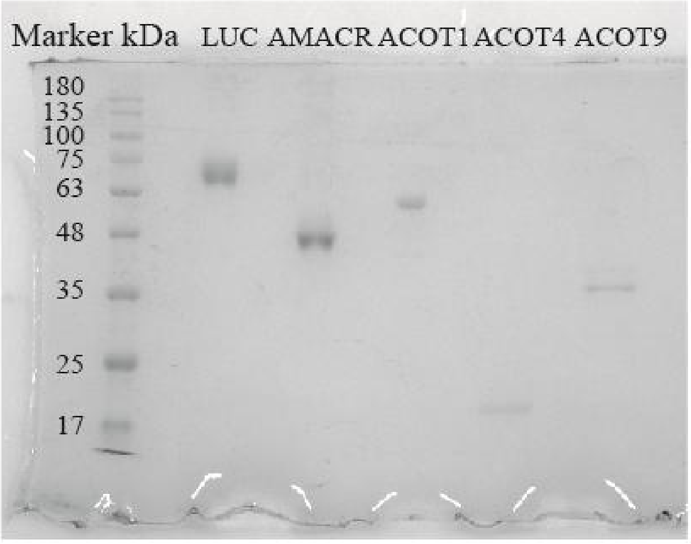

b

Protein	kDa
LUC	59.71
AMACR	42.07
ACOT1	52.50
ACOT4	17.65
ACOT9	30.05

Figure S44. The size and purity of five recombinant proteins *in vitro*. a, The SDS-PAGE image of the synthesized proteins *in vitro*. PR1910 was used as the Marker. **b**, The estimated molecule size of the synthesized proteins using Peptide and Protein Molecular Weight Calculator (https://www.aatbio.com/tools/calculate-peptide-and-protein-molecular-weight-mw).

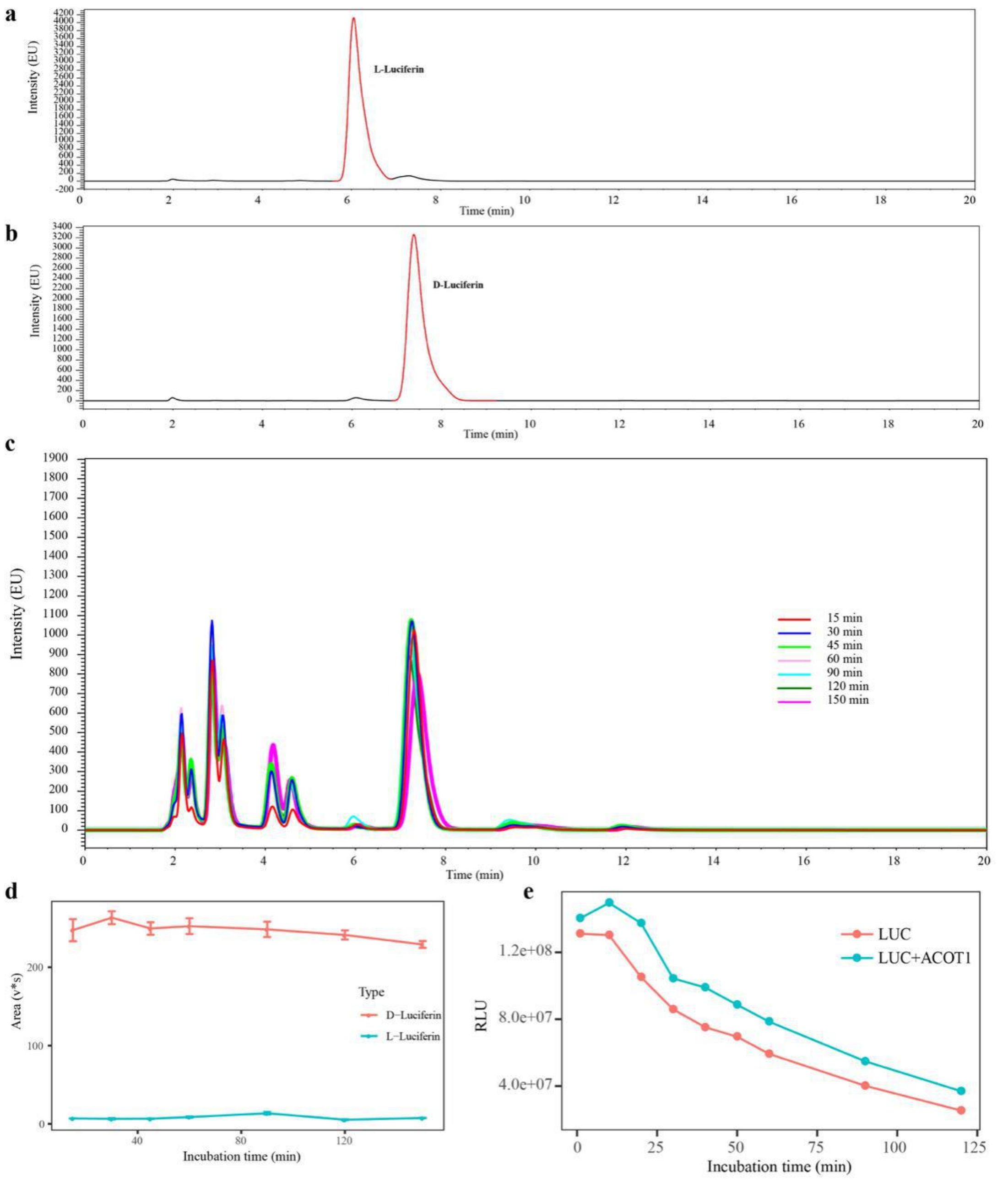

Figure S45. The stereoisomeric inversion of L-luciferin to D-luciferin *in vitro* and their content during different reaction times. a, High performance liquid chromatography (HPLC) chromatogram of L-luciferin standard. **b**, HPLC chromatogram of D-luciferin standard. **c**, HPLC chromatogram of stereoisomeric inversion of L-luciferin to D-luciferin *in vitro* by luciferase (LUC) and acyl-CoA thioesterase 1 (ACOT1) in the 100 mM Tris-HCl (pH=8.0) mixtures of ATP, Mg^{2+} and CoA at 30°C during different reaction time from 15 min to 150 min. **d,** the peak area (unit: v*s) of L-luciferin and D-luciferin during the time points same as those in panel **c**, with three replicates of incubation reaction. **e**, consumption of newly produced D-luciferin from L-luciferin catalyzed by luciferase (LUC) only or LUC and acyl-CoA thioesterase 1 (ACOT1) in the 100 mM Tris-HCl (pH=8.0) mixtures of

ATP, Mg^{2+} and CoA at 25 ℃ during different incubation time. RLU: relative light units. The **d** and **e** were visualized using R package.

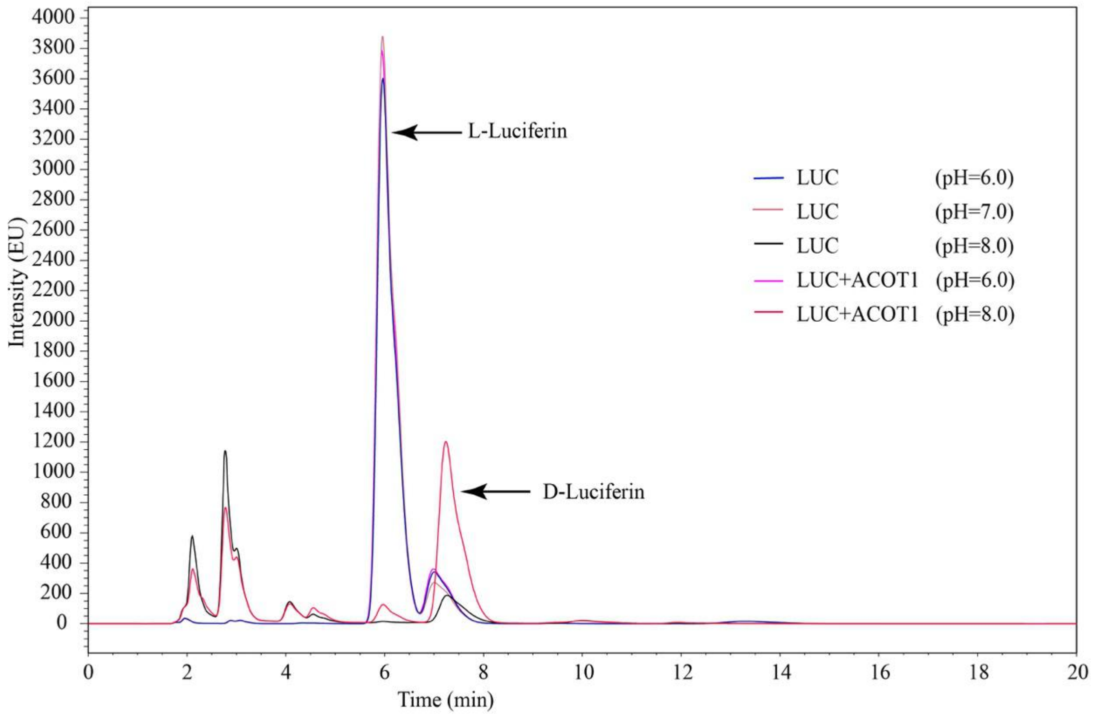

Figure S46. Comparison of stereoisomeric inversion of L-luciferin to D-luciferin by luciferase (LUC) only or LUC and acyl-CoA thioesterase (ACOT) under three different pH conditions (pH=6.0, pH=7.0 and pH=8.0) *in vitro*. The reaction mixtures composed of L-luciferin, LUC or LUC+ACOT1, 100 mM Tris-HCl, ATP, Mg^{2+} and CoA, and were incubated for 45 min at 30 °C. Considering that LUC showed low activity in pH=7.0 in our pilot experiment, we tested two pH (6.0 and 8.0) in the case of LUC+ACOT1.

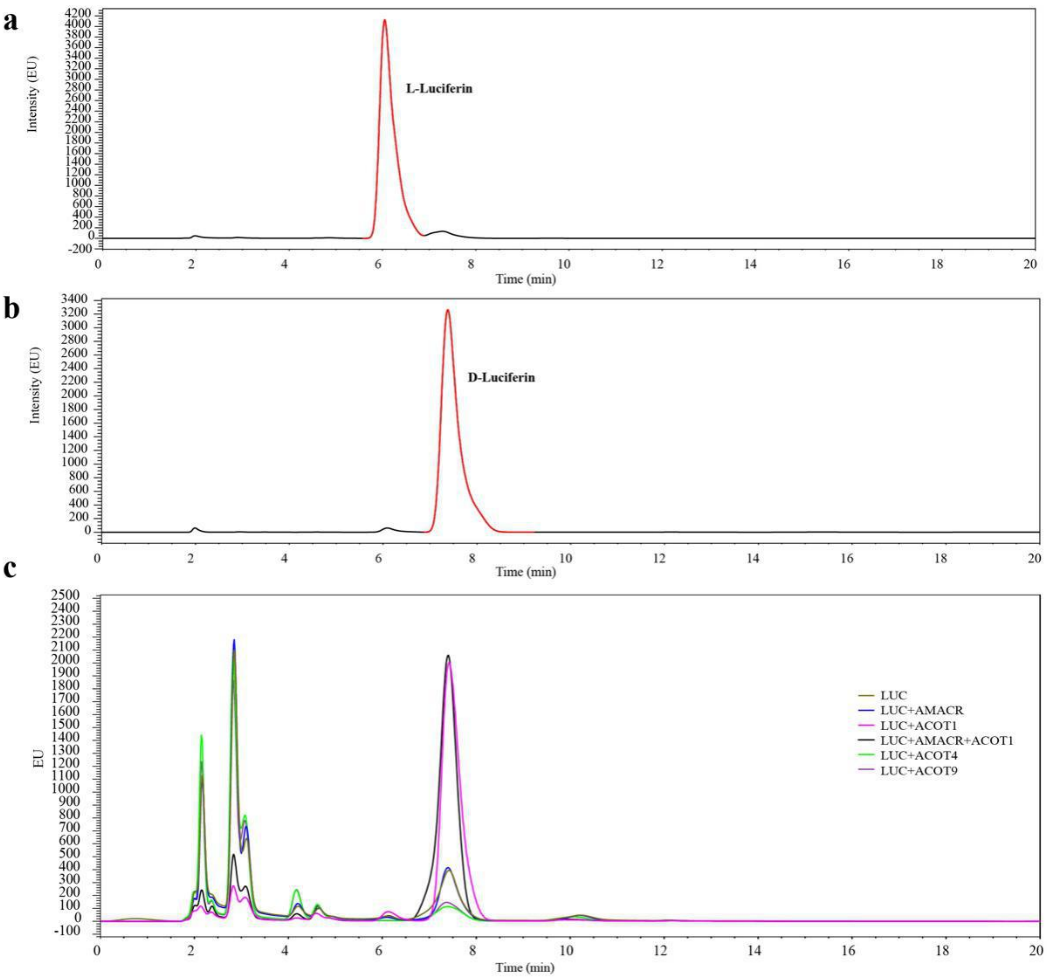

d

	Samples		P value
	LUC	LUC+AMACR	
L-luciferin	84.159±0.148	43.910±0.066	0.027

Figure S47. The stereoisomeric inversion of L-luciferin to D-luciferin *in vitro*. a, High performance liquid chromatography (HPLC) chromatogram of L-luciferin standard. **b**, HPLC chromatogram of D-luciferin standard. **c**, HPLC chromatogram of stereoisomeric inversion of L-luciferin to D-luciferin *in vitro* by luciferase (LUC), acyl-CoA thioesterases (ACOT) and alpha-methyl-acyl-CoA-racemase (AMACR) in the 100 mM Tris-HCl (pH=8.0) mixtures of ATP, Mg^{2+} and CoA at 30°C for 45 min. **d**, Comparison of remaining L-luciferin in the mixtures of LUC or LUC+AMACR showing the promotion role of AMACR in consuming L-luciferin. Values correspond to the average ± standard deviation of three replicates with the data of peak area (unit: v*s). P value was calculated using *t-test*.

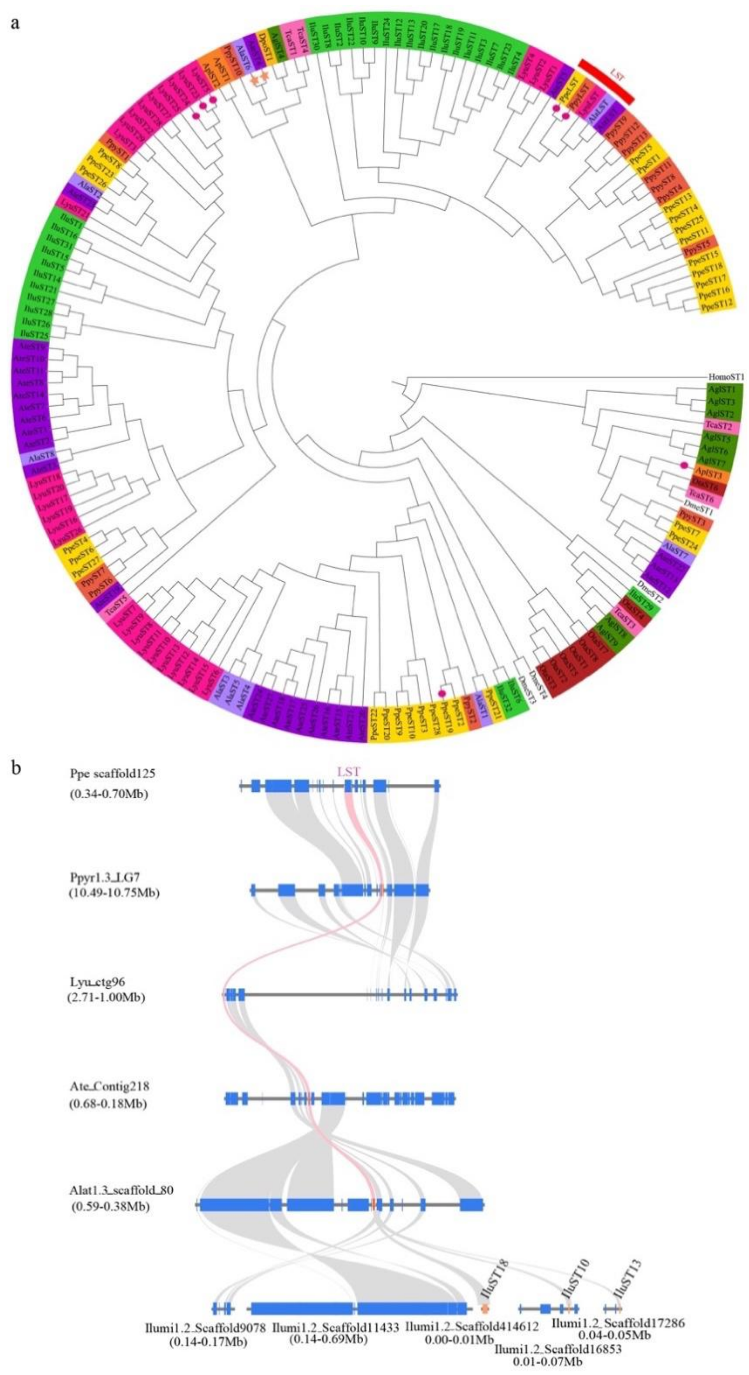

Figure S48. The evolution of luciferin sulfotransferase (LST) and other sulfotransferase (ST) genes in beetles and fruit fly. a shows the phylogenetic tree with ST1 of *Homo sapiens* (HomoST1) as the outgroup. The red circles show those genes with the C-terminal peroxisomal targeting signal 1 (PTS1). The candidates in luciferin biosynthesis are shown as bold clade. **b** shows the syntenic relationships of LST gene (b, highlighted in pink) and its flanking genes surrounding up-200kb and down-200kb genomic regions among six luminous beetles. Lyu: *Lamprigera yunnana*; Ate: *Abscondita terminalis*; Ala: *Aquatica lateralis*; Ppy: *Photinus pyralis*; Ppe: *Pyrocoelia pectoralis*; Ilu: *Ignelater luminosus*; Apl: *Agrilus planipennis*; Ota: *Onthophagus taurus*; Agl: *Anoplophora glabripennis*; Dpo: *Dendroctonus ponderosae*; Tca: *Tribolium castaneum*; Dme: *Drosophila melanogaster.*

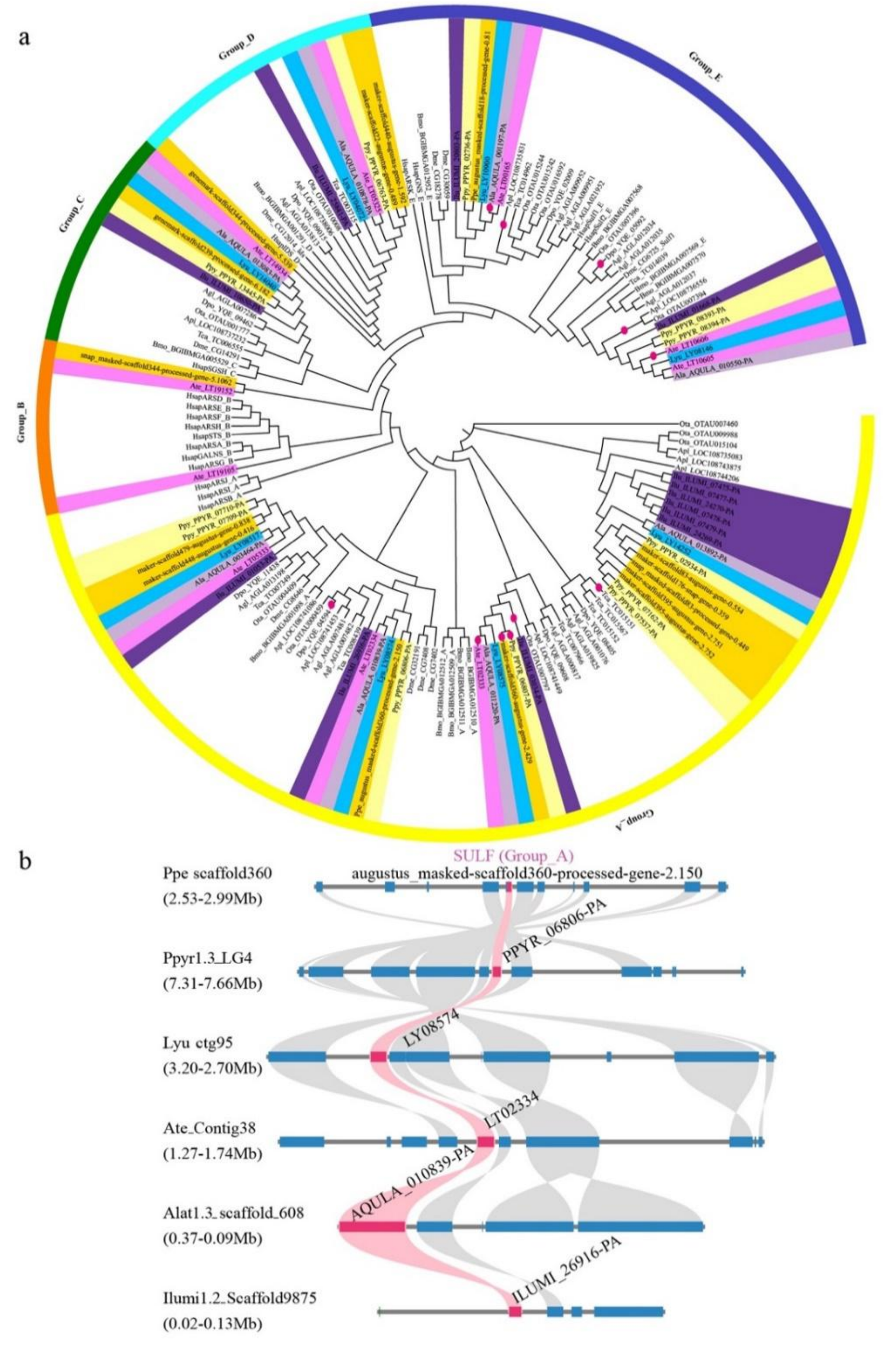

Figure S49. The evolution of sulfatase (SULF) genes in beetles and fruit fly. a shows the phylogenetic tree. The red circles showed those genes with the C-terminal peroxisomal targeting signal 1 (PTS1). **b** shows the syntenic relationships of candidate SULF gene (**b**, highlighted in pink) and its flanking genes surrounding up-200kb and down-200kb genomic regions among six luminous beetles. Lyu: *Lamprigera yunnana*; Ate: *Abscondita terminalis*; Ala: *Aquatica lateralis*; Ppy: *Photinus pyralis*; Ppe: *Pyrocoelia pectoralis*; Ilu: *Ignelater luminosus*; Apl: *Agrilus planipennis*; Ota: *Onthophagus taurus*; Agl: *Anoplophora glabripennis*; Dpo: *Dendroctonus ponderosae*; Tca: *Tribolium castaneum*; Dme: *Drosophila melanogaster*.

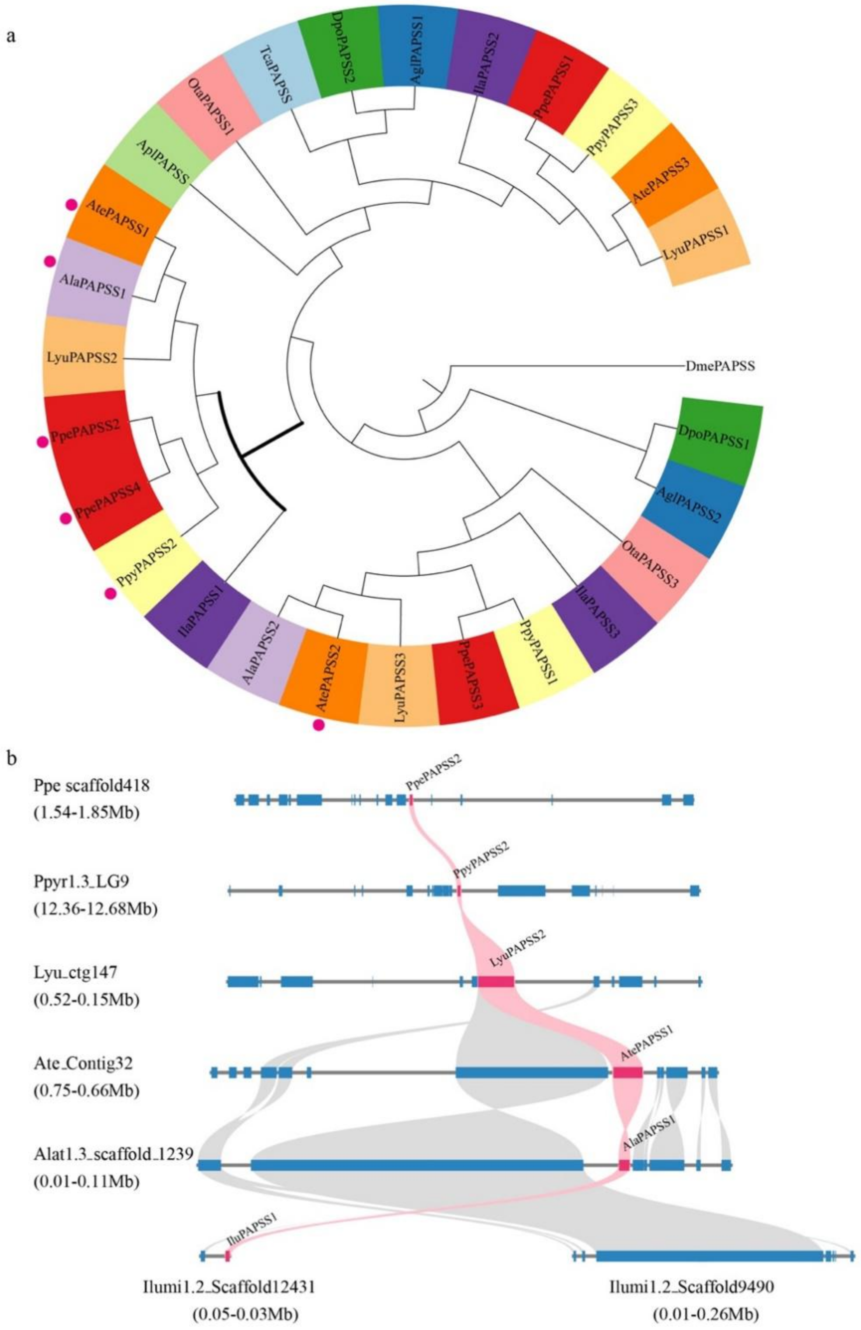

Figure S50. The phylogenetic tree of 5'-phosphosulfate synthetase (PAPSS) genes in beetles and fruit fly. a shows the phylogenetic tree. The red circles show those genes with the C-terminal peroxisomal targeting signal 1 (PTS1). The candidates in luciferin biosynthesis are shown as bold clade. **b** shows the syntenic relationships of PAPSS gene (**b**, highlighted in pink) and its flanking genes surrounding up-200kb and down-100kb genomic regions among six luminous beetles. Lyu: *Lamprigera yunnana*; Ate: *Abscondita terminalis*; Ala: *Aquatica lateralis*; Ppy: *Photinus pyralis*; Ppe: *Pyrocoelia pectoralis*; Ilu: *Ignelater luminosus*; Apl: *Agrilus planipennis*; Ota: *Onthophagus taurus*; Agl: *Anoplophora glabripennis*; Dpo: *Dendroctonus ponderosae*; Tca: *Tribolium castaneum*; Dme: *Drosophila melanogaster*.

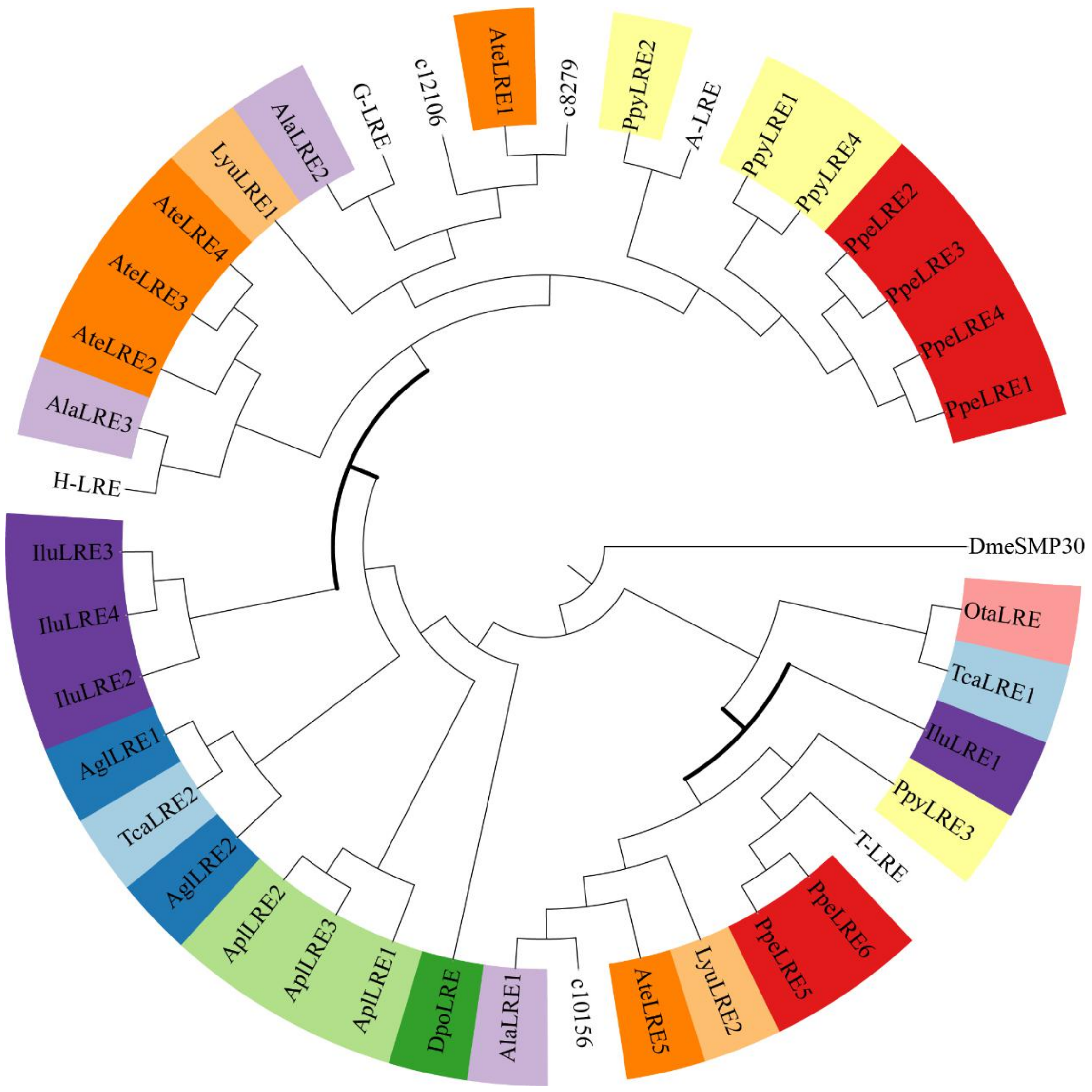

Figure S51. The maximum likelihood (ML) phylogenetic tree of luciferin-regenerating enzyme (LRE) genes in beetles and fruit fly. The candidates in luciferin biosynthesis are shown as bold clade. Lyu: *Lamprigera yunnana*; Ate: *Abscondita terminalis*; Ala: *Aquatica lateralis*; Ppy: *Photinus pyralis*; Ppe: *Pyrocoelia pectoralis*; Ilu: *Ignelater luminosus*; Apl: *Agrilus planipennis*; Ota: *Onthophagus taurus*; Agl: *Anoplophora glabripennis*; Dpo: *Dendroctonus ponderosae*; Tca: *Tribolium castaneum*; Dme: *Drosophila melanogaster*.

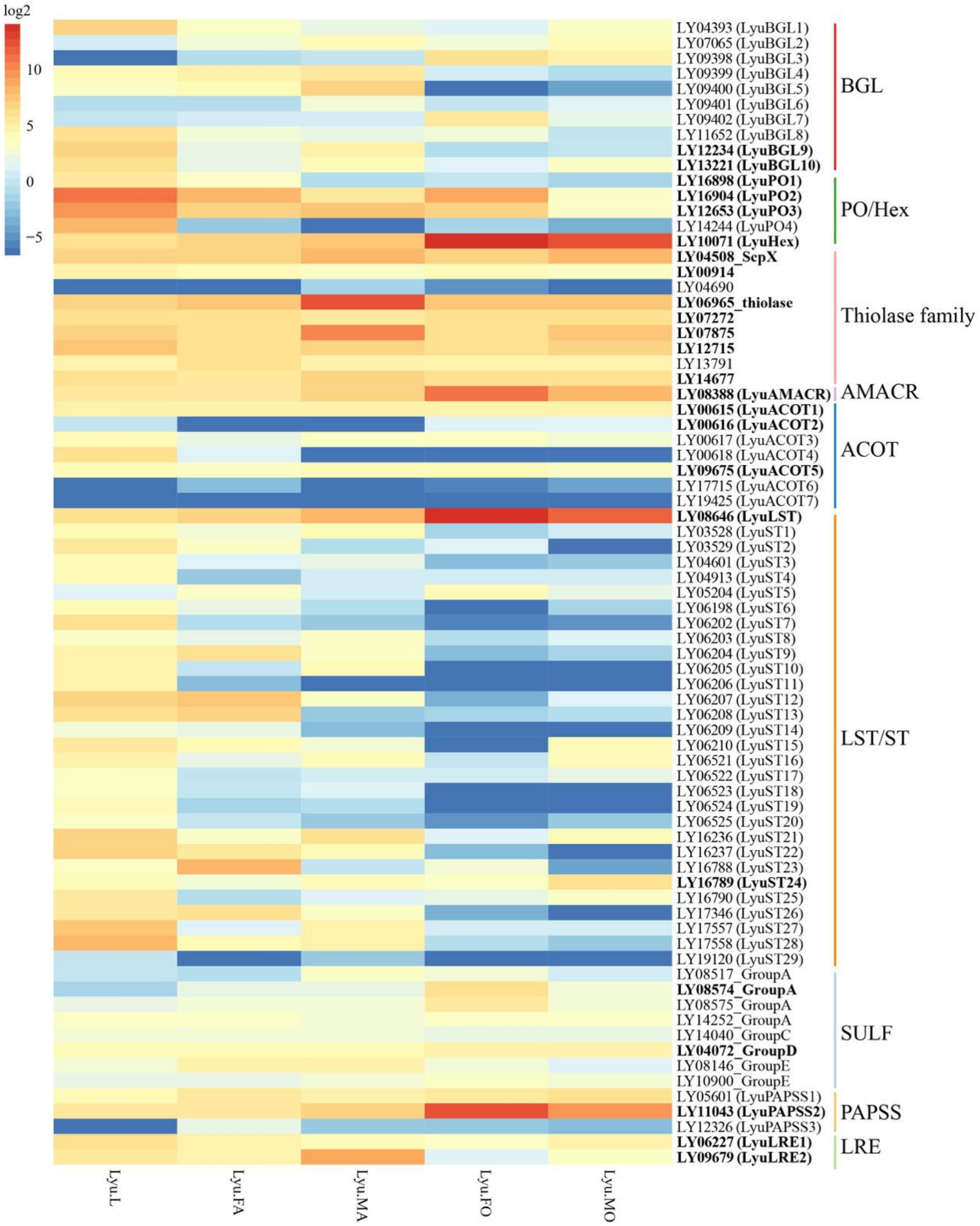

Figure S52. The expression of candidate genes in luciferin synthesis pathway at the transcriptomic level in *L. yunnana* (Lyu). The heat map shows log2-scaled fragments per kilobase of transcript per million fragments (FPKM). MO: luminous organ of male adult; FO: luminous organ of female adult; MA: male adult; FA: female adult; L: larva. Those genes with abundance at proteomic level were shown as bold. R package was used for the visualization of the image.

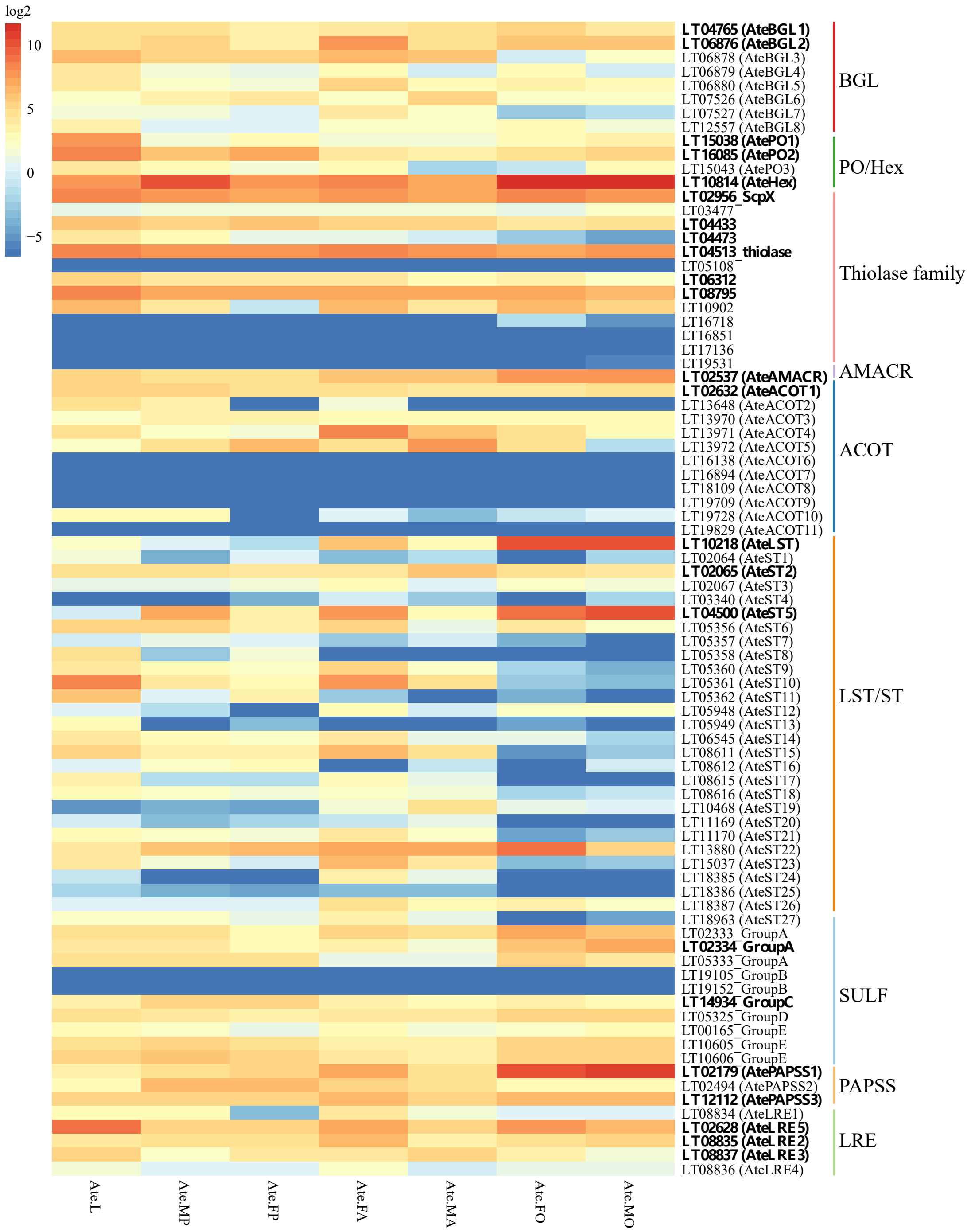

Figure S53. The expression of candidate genes in luciferin synthesis pathway at the transcriptomic level in *A. terminalis* (Ate). The heat map shows log2-scaled fragments per kilobase of transcript per million fragments (FPKM). MO: luminous organ of male adult; FO: luminous organ of female adult; MA: male adult; FA: female adult; MP: male pupa; FP: female pupa; L: larva. Those genes with abundance at proteomic level were shown as bold. R package was used for the visualization of the image.

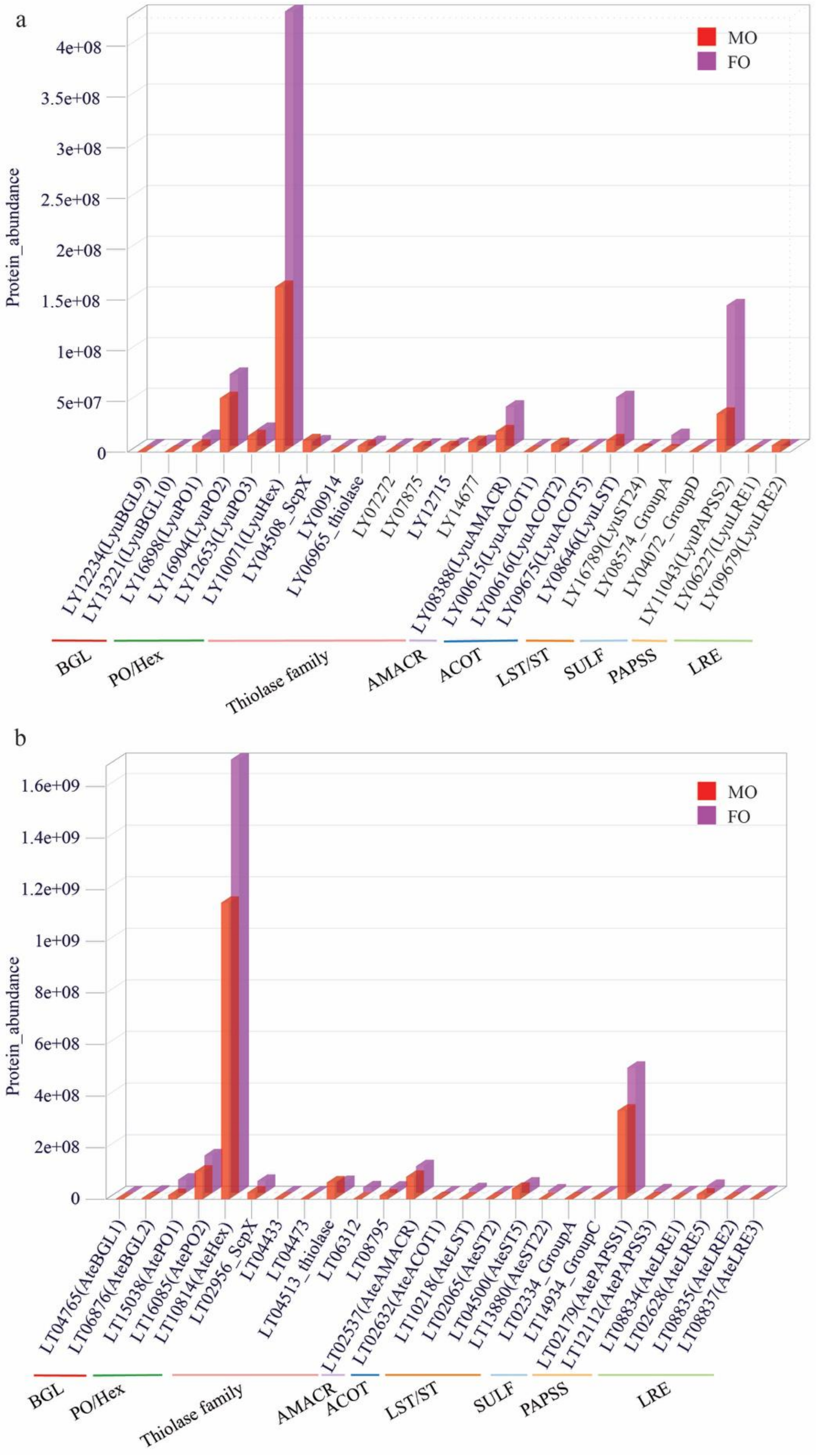

Figure S54. The expression of candidate genes in luciferin synthesis pathway at the proteomic level in *L. yunnana* (Lyu) (a) and *A. terminalis* (Ate) (b). MO: luminous organ of male adult; FO: luminous organ of female adult. R package was used for the visualization of the images.

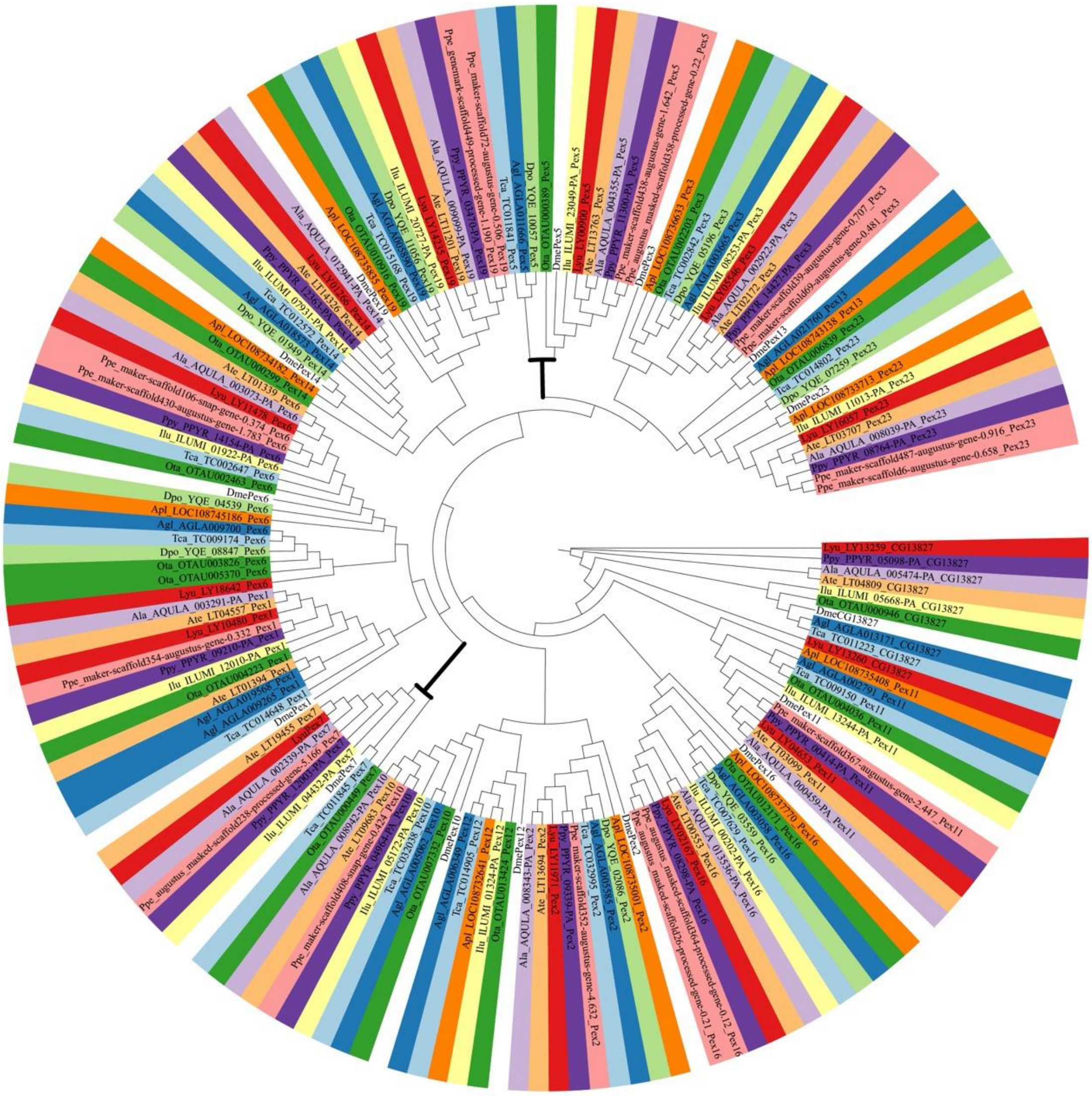

Figure S55. Phylogenetic tree of peroxin (Pex) genes in beetles and fruit fly. The candidate Pex5 and Pex7 are cytosolic receptors, which recognize PTS1 (the C-terminal peroxisomal targeting signal 1) and PTS2 (the N-terminal peroxisomal targeting signal 2) containing proteins, respectively. The candidates related to luciferin biosynthesis are shown as bold clade. Lyu: *Lamprigera yunnana*; Ate: *Abscondita terminalis*; Ala: *Aquatica lateralis*; Ppy: *Photinus pyralis*; Ppe: *Pyrocoelia pectoralis*; Ilu: *Ignelater luminosus*; Apl: *Agrilus planipennis*; Ota: *Onthophagus taurus*; Agl: *Anoplophora glabripennis*; Dpo: *Dendroctonus ponderosae*; Tca: *Tribolium castaneum*; Dme: *Drosophila melanogaster*.

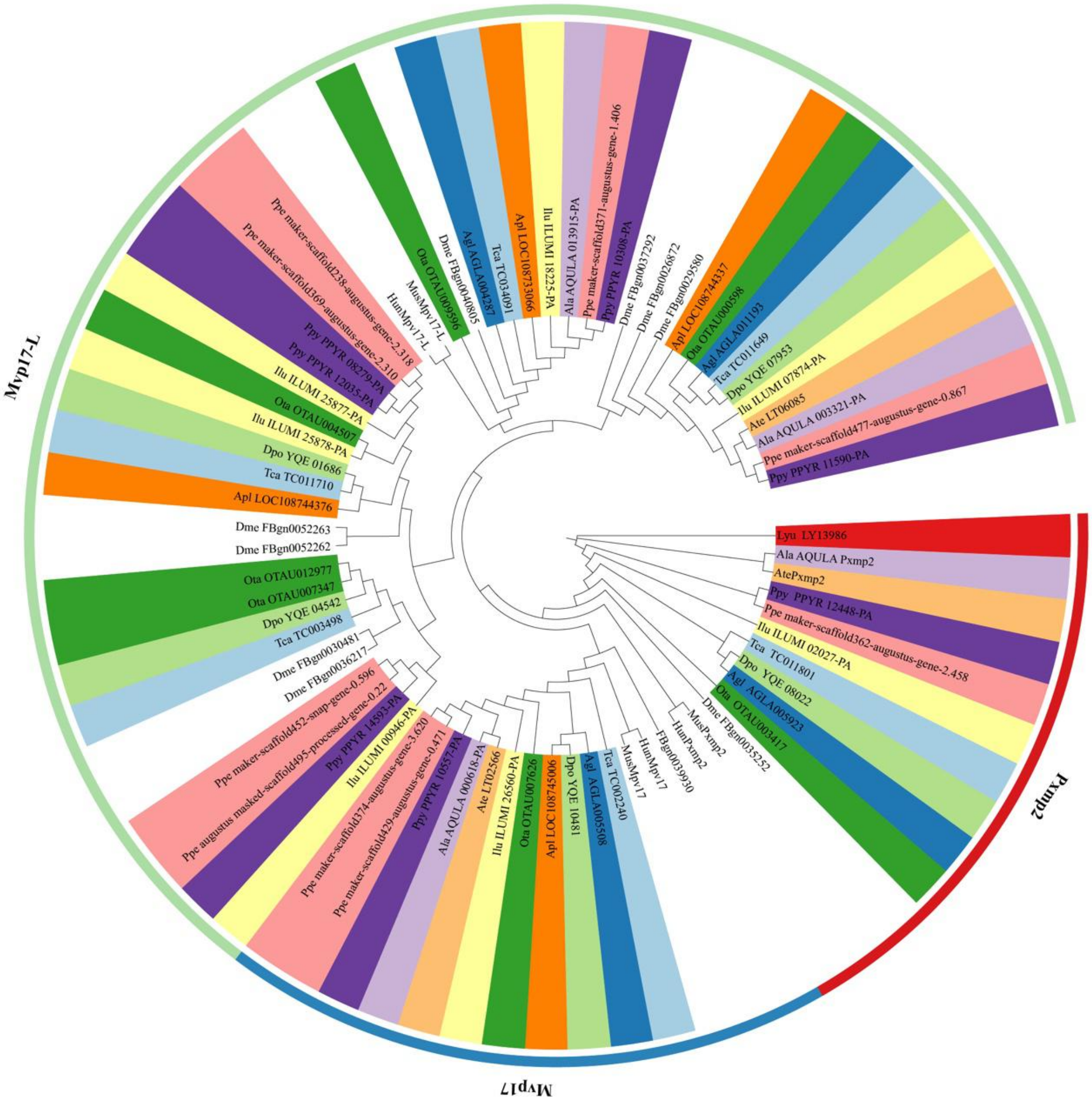

Figure S56. Phylogenetic tree of peroxisomal membrane protein 2 (Pxmp2) family genes. The three subfamilies: Pxmp2, Mpv17 and Mpv17-L. Lyu: *Lamprigera yunnana*; Ate: *Abscondita terminalis*; Ala: *Aquatica lateralis*; Ppy: *Photinus pyralis*; Ppe: *Pyrocoelia pectoralis*; Ilu: *Ignelater luminosus*; Apl: *Agrilus planipennis*; Ota: *Onthophagus taurus*; Agl: *Anoplophora glabripennis*; Dpo: *Dendroctonus ponderosae*; Tca: *Tribolium castaneum*; Dme: *Drosophila melanogaster*.

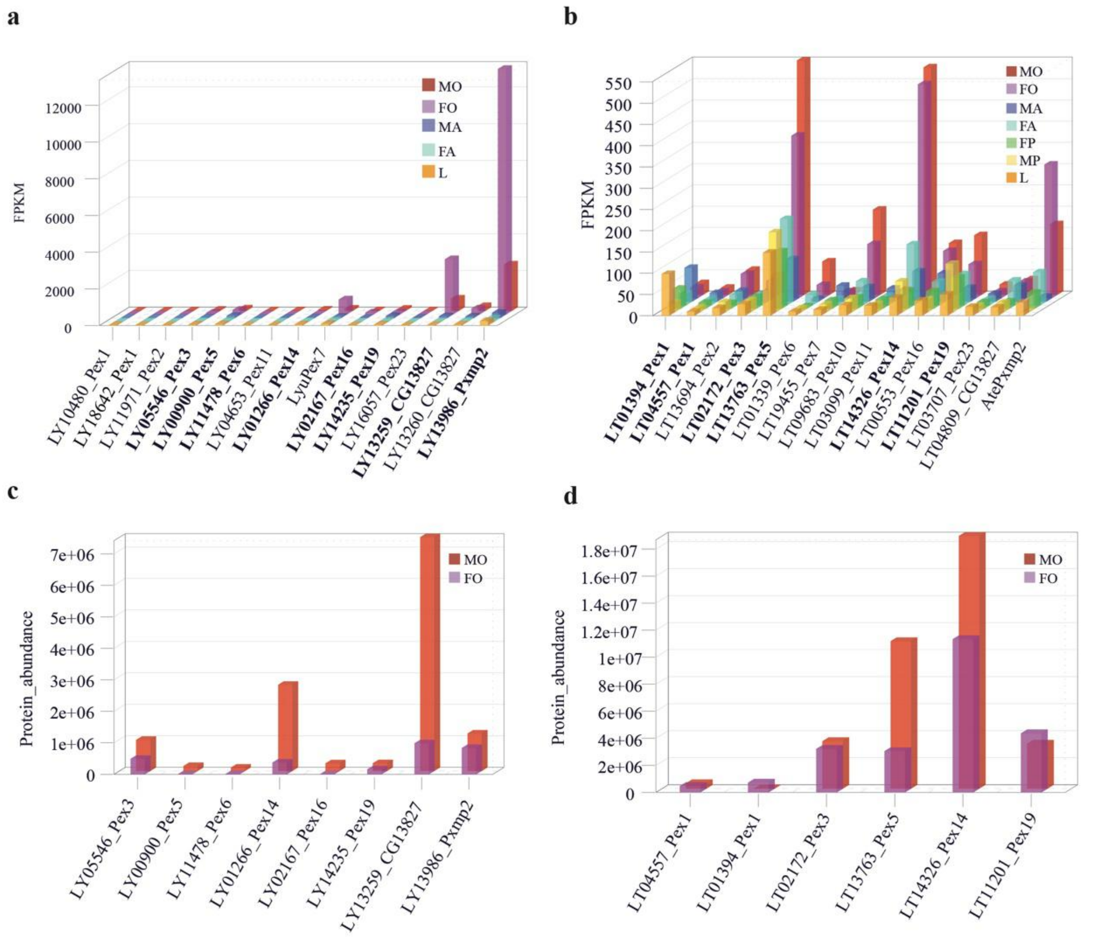

Figure S57. The expression of peroxin (Pex) and peroxisomal membrane protein 2 (Pxmp2) genes in *L. yunnana* (Lyu) and *A. terminalis* (Ate). a and **b** show the expression of the genes at transcriptomic level in *L. yunnana* and *A. terminalis*, respectively. LyuPex7 and AtePxmp2 were manually identified. Those genes with abundance at proteomic level were shown as bold. **c** and **d** show the expression of the genes identified at the proteomic level in *L. yunnana* and *A. terminalis*, respectively. MO: luminous organ of male adult; FO: luminous organ of female adult; MA: male adult; FA: female adult; MP: male pupa; FP: female pupa; L: larva. R package was used for the visualization of the image.

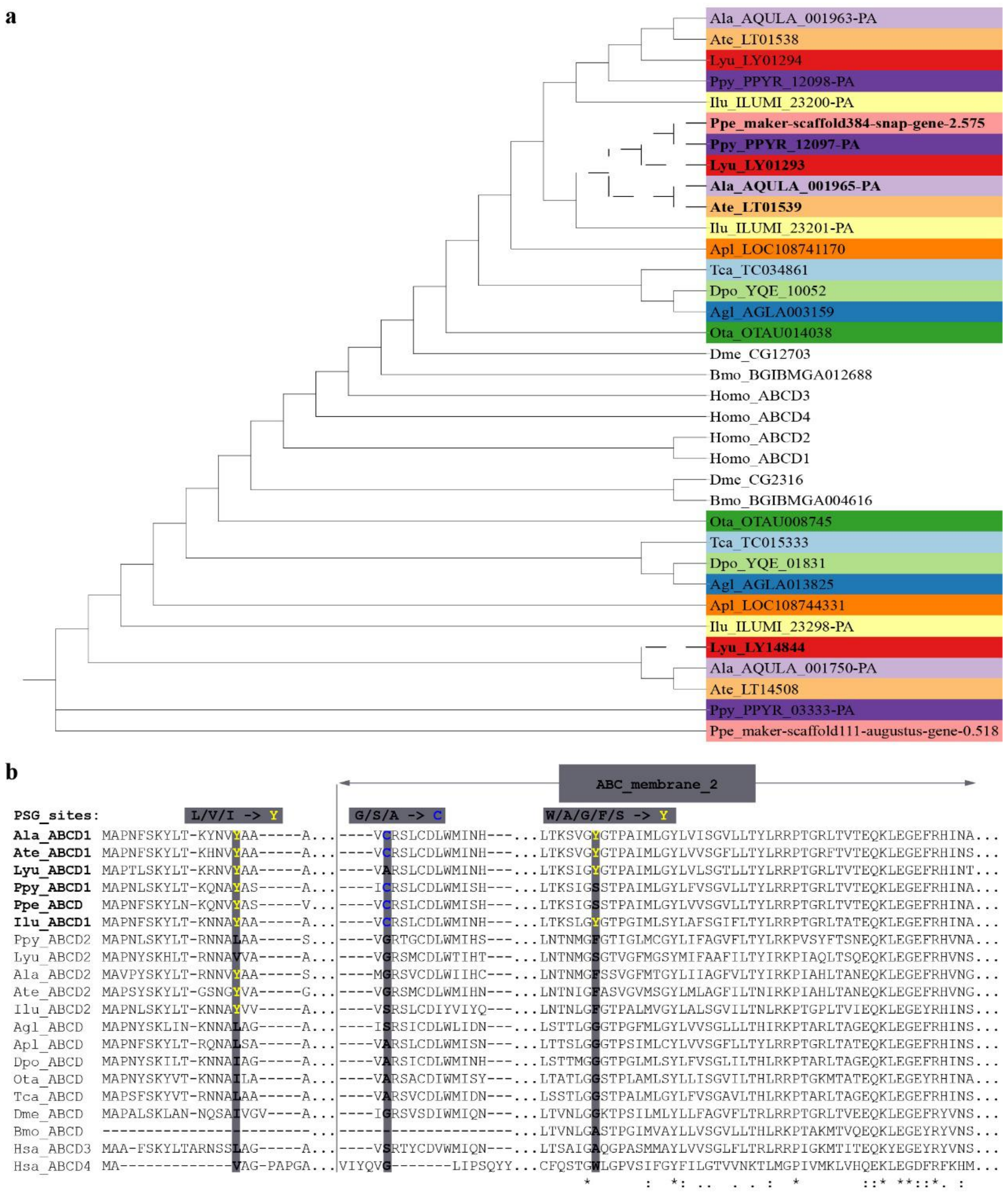

Figure S58. Evolution analysis of ATP-binding cassette protein D (ABCD) transporter subfamilies in 11 beetles, fruit fly, silkworm and human. a shows phylogenetic tree of ABCD genes. **b** shows the positive selected sites in ancestor of luminous beetles. Shadow denotes the positive selected amino acids. Lyu: *Lamprigera yunnana*; Ate: *Abscondita terminalis*; Ala: *Aquatica lateralis*; Ppy: *Photinus pyralis*; Ppe: *Pyrocoelia pectoralis*; Ilu: *Ignelater luminosus*; Apl: *Agrilus planipennis*; Ota: *Onthophagus taurus*; Agl: *Anoplophora glabripennis*; Dpo: *Dendroctonus ponderosae*; Tca: *Tribolium castaneum*; Dme: *Drosophila melanogaster*; Bmo: *Bombyx mori*; Homo: *Homo sapiens*.

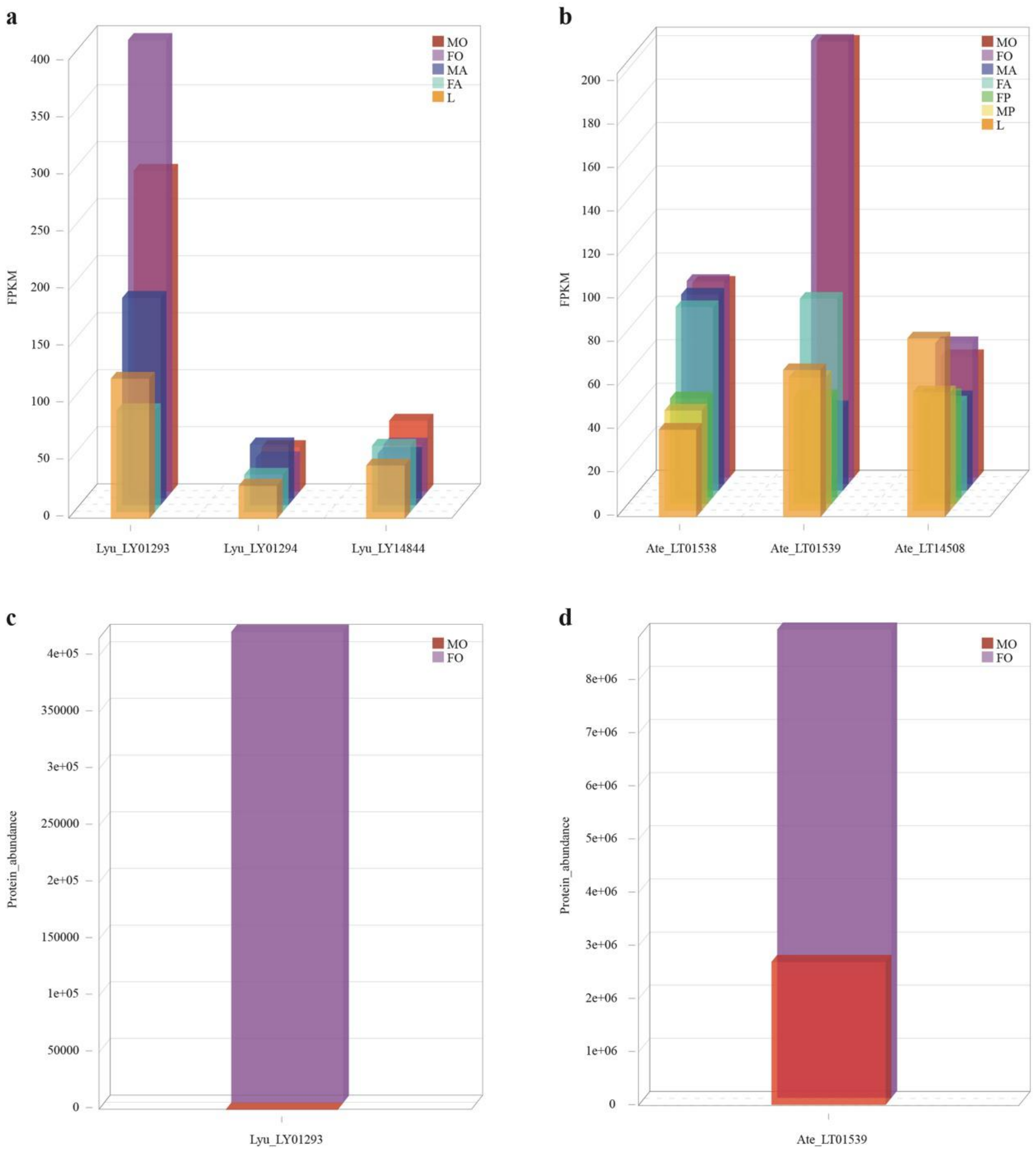

Figure S59. The expression of ATP-binding cassette subfamily D (ABCD) genes in *L. yunnana* (Lyu)and *A. terminalis* (Ate). a and **b** show the expression of ABCD genes at transcriptomic level in *L. yunnana* and *A. terminalis*, respectively. **c** and **d** show the expression of ABCD genes at proteomic level in *L. yunnana* and *A. terminalis*, respectively. MO: luminous organ of male adult; FO: luminous organ of female adult; MA: male adult; FA: female adult; MP: male pupa; FP: female pupa; L: larva. R package was used for the visualization of the images.

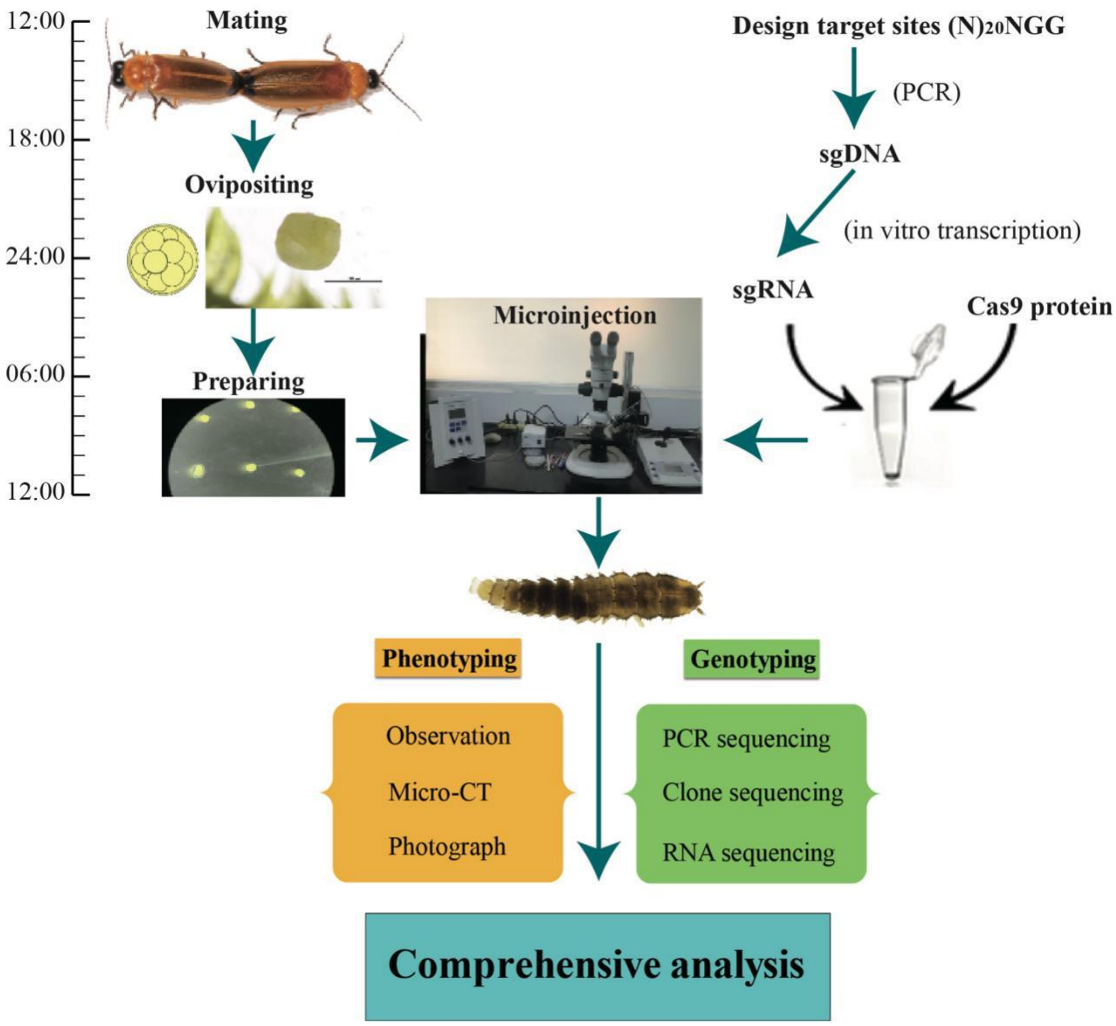

Figure S60. Flowchart of CRISPR/Cas9 technology in firefly.

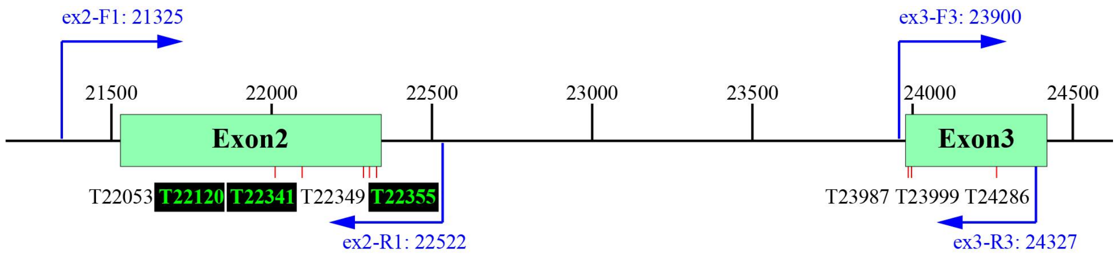

Figure S61. The schematic diagram of target sites (2nd exon: T22053, T22120, T22341, T22349, T22355; 3rd exon: T23987, T23999, T24286) and the primers (ex2-F1/R1, ex3-F3/R3) used in *Abd-B* gene editing.

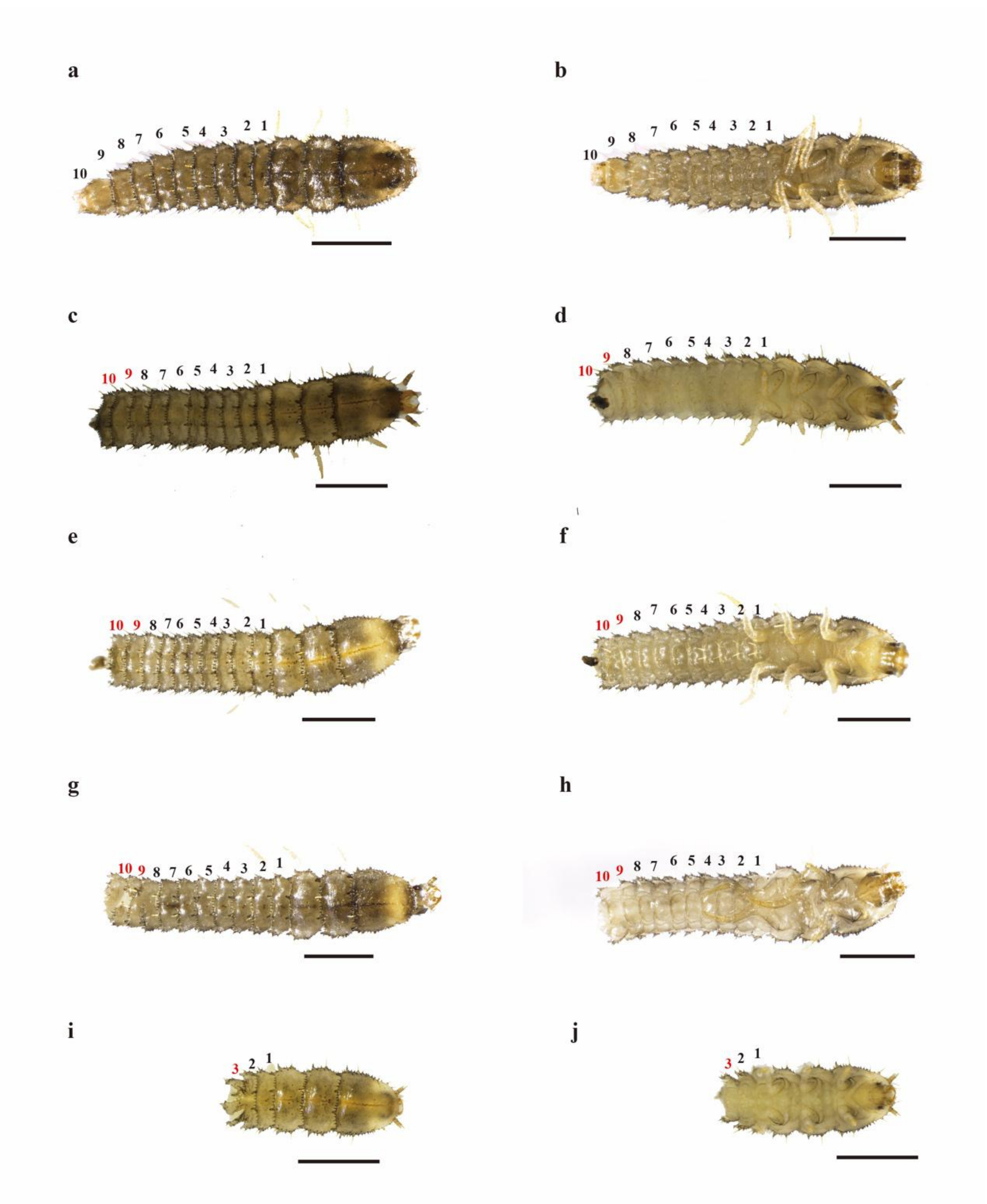

Figure S62. Mutants with abnormal abdomen induced by Cas9-sgRNA injection of *Abd-B* gene in *A. terminalis*. (**a-b**) The first instar larva of wild type in dorsal (**a**) and ventral (**b**) views. (**c-j**) The first instar larva of mutation types in dorsal (**c**, **e**, **g** and **i**) and ventral (**d**, **f**, **h** and **j**) views, respectively. The numbers denote the abdominal segments, with red ones showing abnormal segments in abdomen. Scale bars: 1 mm.

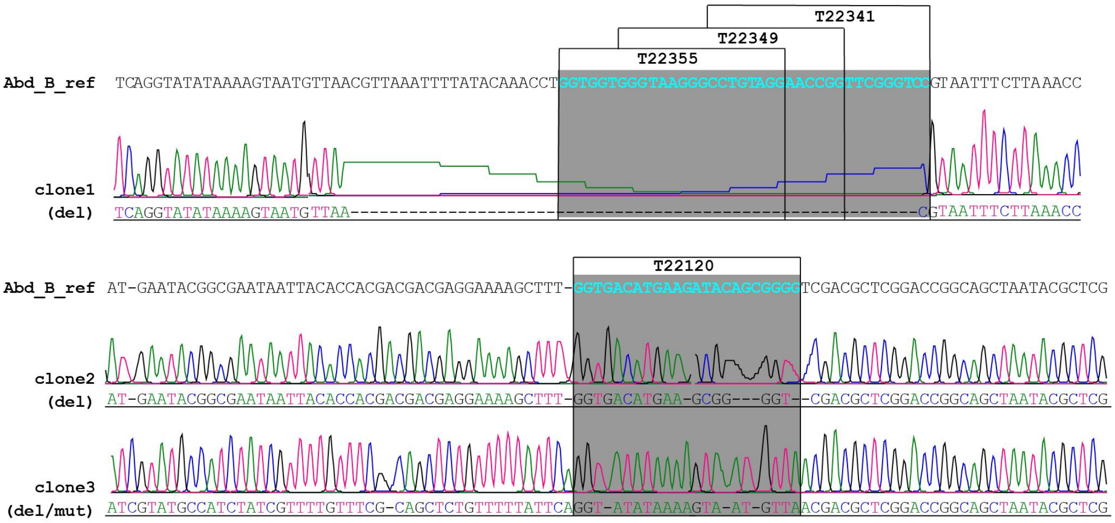

Figure S63. Sequencing of four candidate target sites (T22120, T22341-T22349-T22355) of *Abd-B* mutants in *A. terminalis* injected with mixed sgRNAs and Cas9 protein in 2017. Abd_B_ref represents wild-type sequence, and clones represent the mutated clones. Gray shadow showed the region of target sites. del: deletion. mut: mutation.

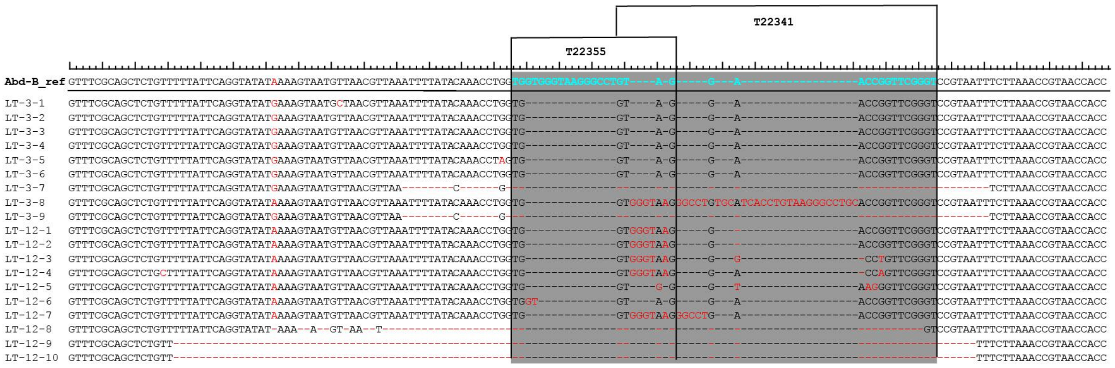

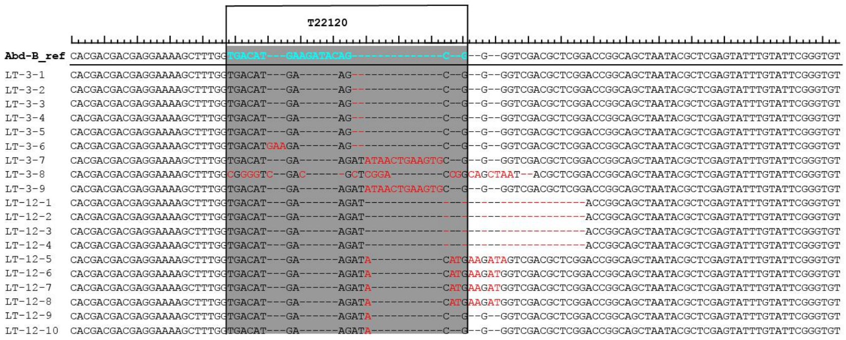

Figure S64. Sequencing the target sites of *Abd-B* mutants in *A. terminalis* injected with sgRNAs of three target sites (T22120, T22341-22355) and Cas9 protein in 2018. Abd-B_ref represents wild-type sequence, LT-3 (1-9) and LT-12 (1-10) represent two abnormal individuals (mutated clones of each mutant individual). Gray shadows show mutation base in target sites.

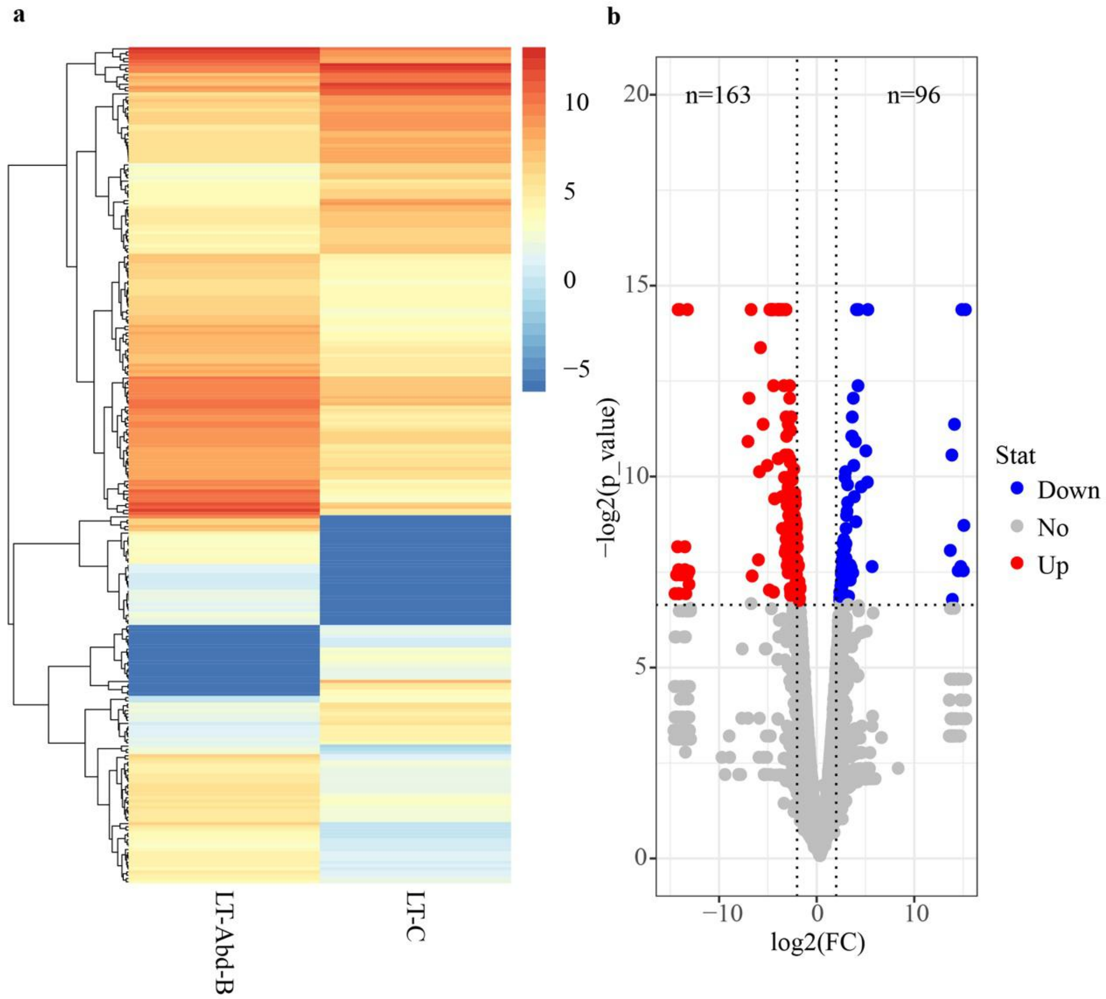

Figure S65. The difference expression genes (DEGs) between CRISPR/Cas9-induced mutant and wild type of *A. terminalis*. a shows the expression heatmap of 259 DEGs at transcriptional level in *A. terminalis* mutant. LT-Abd-B represents the mutant and LT-C represents the wild type. **b** shows the up-regulation and down-regulation of DEGs between mutant and wild in *A. terminalis*. The broken lines indicate that the absolute value of log2-fold change (log2(FC)) is equal to 2 and P-value is equal to 0.01. Blue and red blots indicate down-regulated genes and up-regulated genes in *A. terminalis* mutants, respectively. “n” shows down-regulated and up-regulated gene numbers. R package was used for the visualization of the images.

a

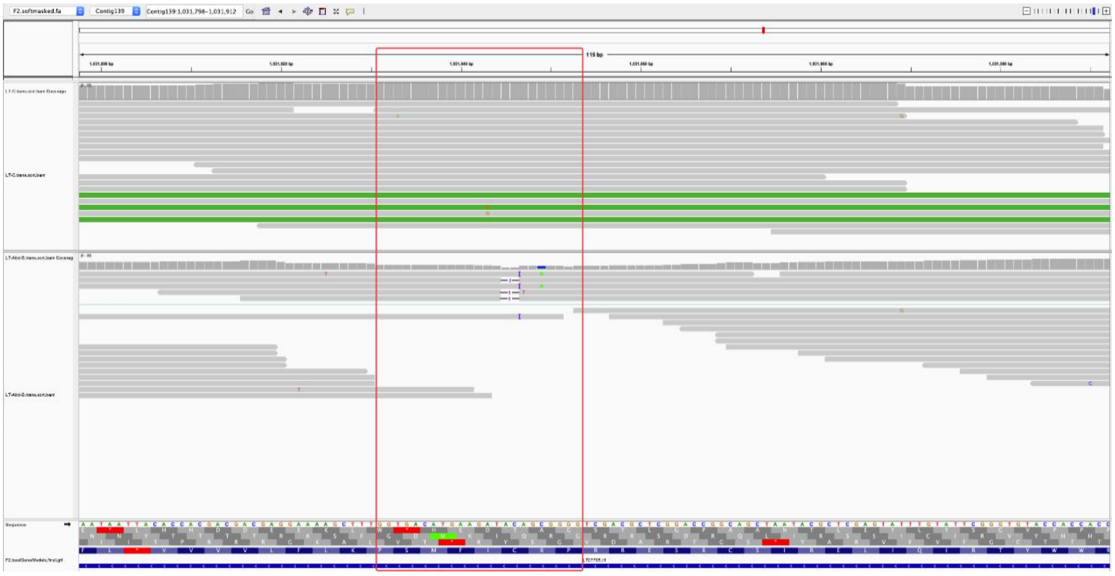

b

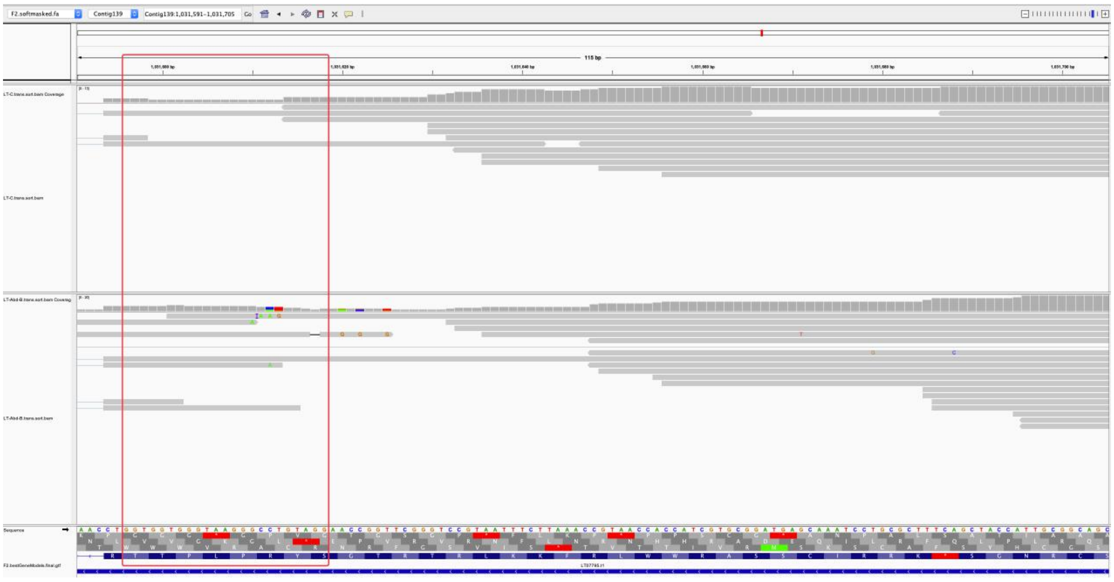

Figure S66. The reads mapping (up: transcriptomic reads of wild type, below: transcriptomic reads of mutant) between *A. terminalis* mutant (LT-Abd-B) and wild type (LT-C) in target sites. Red boxes show target regions. **a** shows the T22120 target sequence (TGACATGAAGATACAGCG) and **b** shows the T22355 target sequence (TGGTGGGTAAGGGCCTGT). The **a** and **b** were showed using the Integrative Genomics Viewer (http://software.broadinstitute.org/software/igv/).

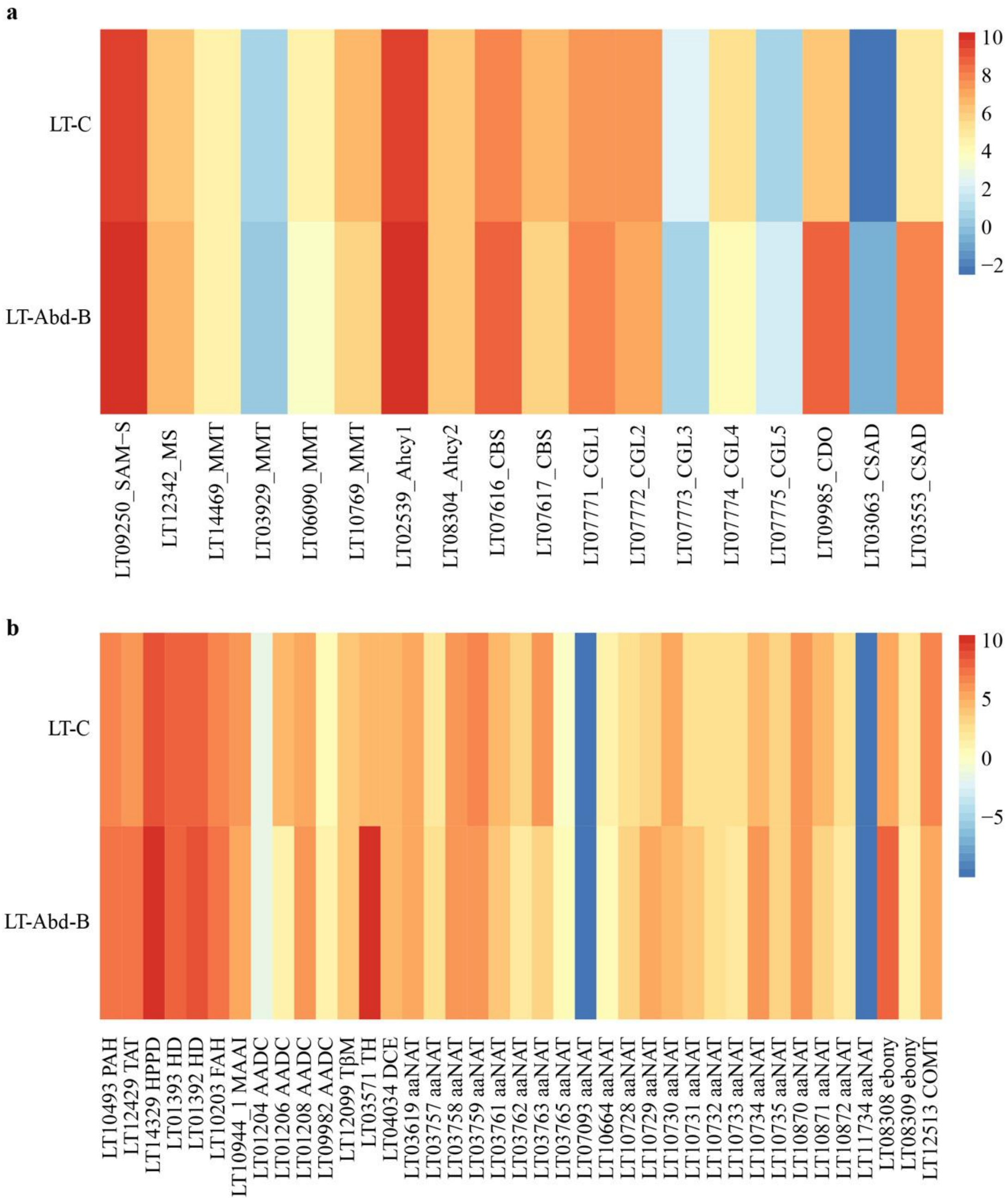

Figure S67. The expression of genes in cysteine metabolism pathway (a) and in tyrosine metabolism pathway (b) between *A. terminalis* mutants and wild. The heat map shows log2-scaled fragments per kilobase of transcript per million fragments (FPKM). LT-C: wild; LT-Abd-B: mutant. R package was used for the visualization of the images.

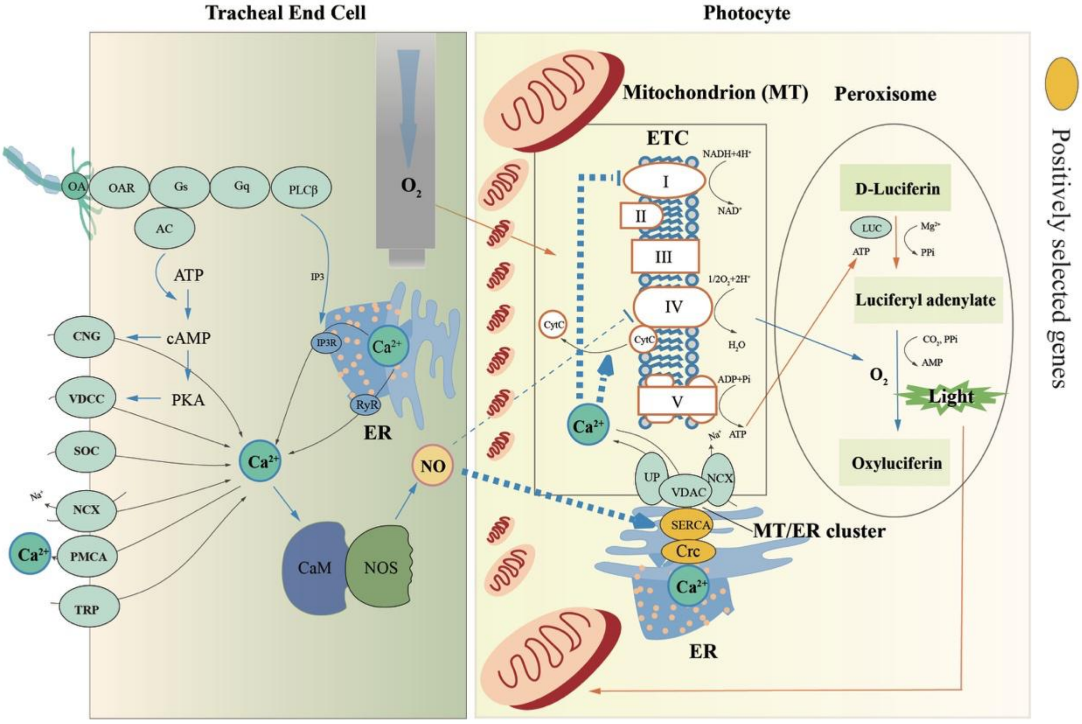

Figure S68. The proposed model of flash control. Orange lines, active during light "off"; blue lines, active during light "on"; thin lines, previously reported [1, 2]; thick dashed lines, currently proposed; lines with arrows, promotion; lines with vertical bars, prohibition. Octopamine (OA), Octopamine receptors (OAR), One member (s) of α subunits of guanine nucleotide-binding (G) proteins (Gs), One member (s) of α subunits of guanine nucleotide-binding (G) proteins (Gq), Adenylate cyclase (AC), Adenosine triphosphate (ATP), circle adenosine 3'5' monophosphate (cAMP), protein kinase A (PKA), A family of phosphoinositide-specific phospholipase C (PLC) enzymes (PLCβ), Inositol-1,4,5-trisphophate (IP3) receptor (Ins(1,4,5)P3R, IP3R), Ryanodine receptor (RyR), Voltage-dependent calcium channel (VDCC), Cyclic nucleotide-gated ion channel (CNG), plasma membrane Ca^{2+}-ATPase (PMCA), Store-operated channel (SOC), Transient receptor potential ion-channel (TRP), Voltage-dependent anion channel (VDAC), Calmodulin (CAM), Nitric Oxide (NO), Nitric oxide synthase (NOS), Sarco (endo)-plasmic reticulum Ca^{2+}-ATPase (SERCA), Calreticutin (Crc), Sodium-calcium exchanger (NCX), Calcium uniporter (UP), Luciferase (LUC).

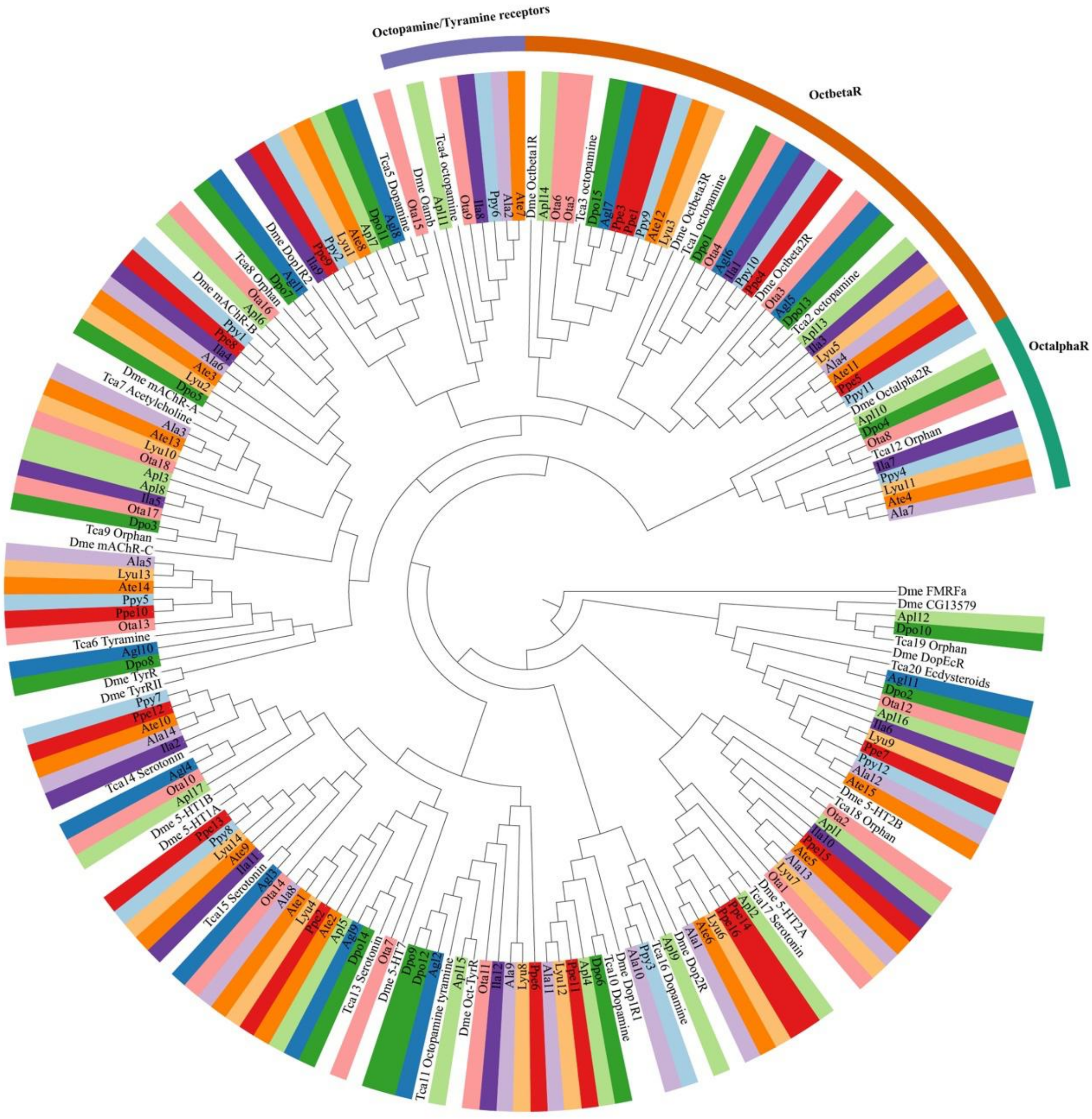

Figure S69. The maximum likelihood (ML) tree of biogenetic amine G protein-coupled receptors (GPCRs) genes in 11 beetles with neuropeptide Phe-Met-Arg-Phe-NH2 (FMRFamide) of fruit fly *D. melanogaster* (DmeFMRFa, *CG2346*) as an outgroup. The outside circle showed the clade of octopamine receptors (OAR). Lyu: *Lamprigera yunnana*; Ate: *Abscondita terminalis*; Ala: *Aquatica lateralis*; Ppy: *Photinus pyralis*; Ppe: *Pyrocoelia pectoralis*; Ilu: *Ignelater luminosus*; Apl: *Agrilus planipennis*; Ota: *Onthophagus taurus*; Agl: *Anoplophora glabripennis*; Dpo: *Dendroctonus ponderosae*; Tca: *Tribolium castaneum*; Dme: *Drosophila melanogaster*.

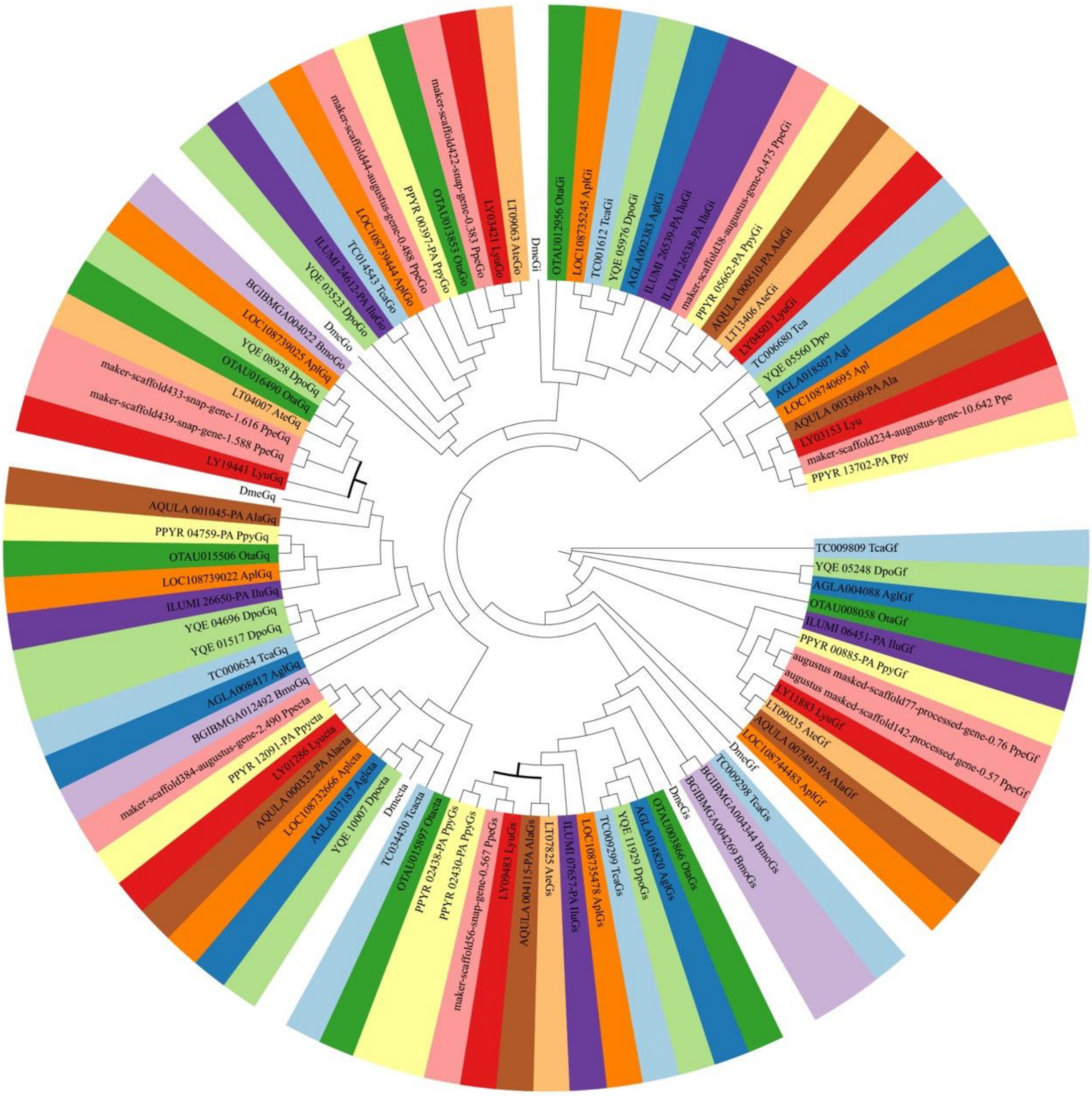

Figure S70. The maximum likelihood (ML) tree of stimulatory guanine nucleotide-binding (G) protein alpha subunit (Gα) genes in 11 beetles, silkworm and fruit fly. Lyu: *Lamprigera yunnana*; Ate: *Abscondita terminalis*; Ala: *Aquatica lateralis*; Ppy: *Photinus pyralis*; Ppe: *Pyrocoelia pectoralis*; Ilu: *Ignelater luminosus*; Apl: *Agrilus planipennis*; Ota: *Onthophagus taurus*; Agl: *Anoplophora glabripennis*; Dpo: *Dendroctonus ponderosae*; Tca: *Tribolium castaneum*; Bmo: *Bombyx mori*; Dme: *Drosophila melanogaster*.

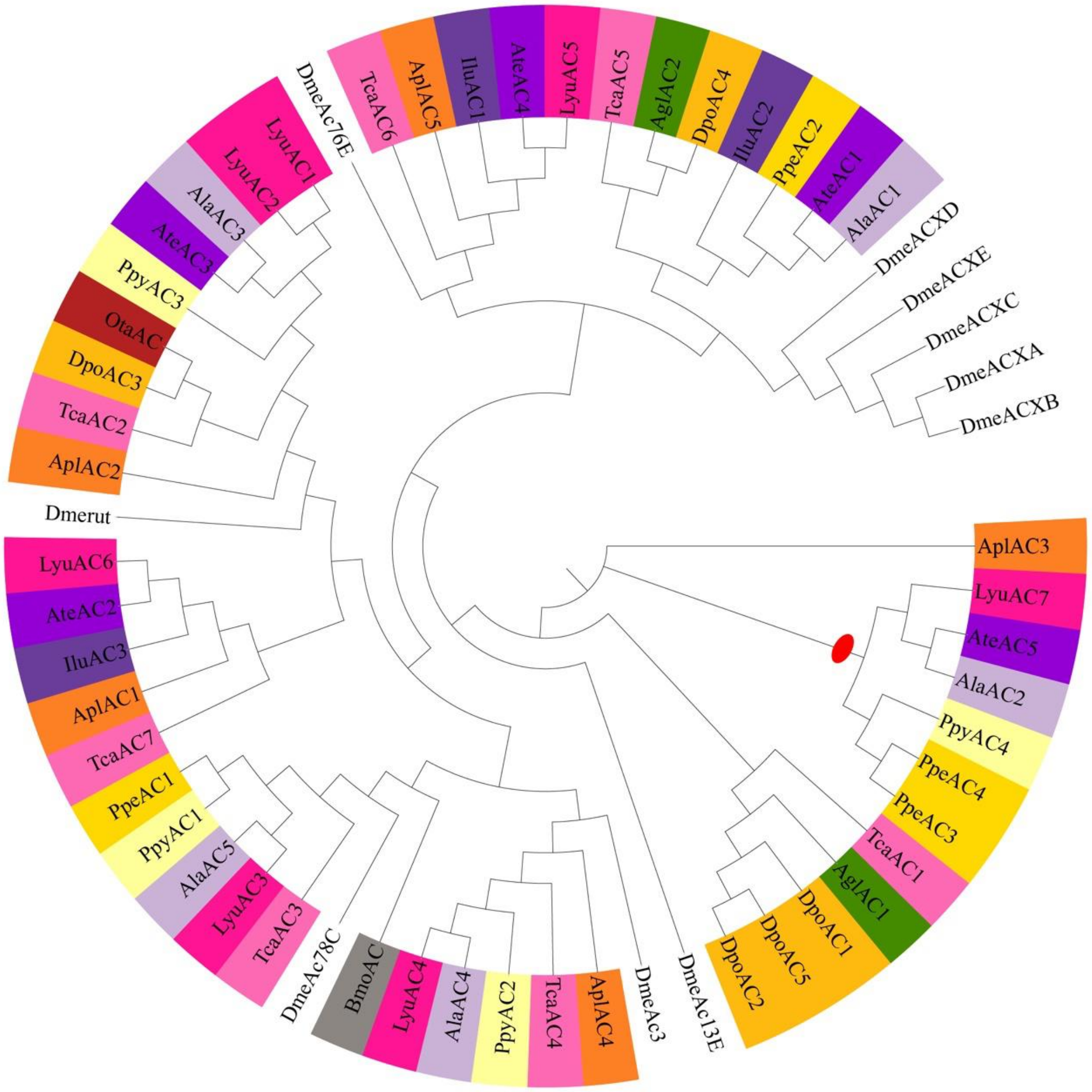

Figure S71. The maximum likelihood (ML) tree of adenylyl cyclase (AC) genes in 11 beetles, silkworm and fruit fly. Filled red circle indicates candidate AC possibly related to flash control. Lyu: *Lamprigera yunnana*; Ate: *Abscondita terminalis*; Ala: *Aquatica lateralis*; Ppy: *Photinus pyralis*; Ppe: *Pyrocoelia pectoralis*; Ilu: *Ignelater luminosus*; Apl: *Agrilus planipennis*; Ota: *Onthophagus taurus*; Agl: *Anoplophora glabripennis*; Dpo: *Dendroctonus ponderosae*; Tca: *Tribolium castaneum*; Bmo: *Bombyx mori*; Dme: *Drosophila melanogaster*.

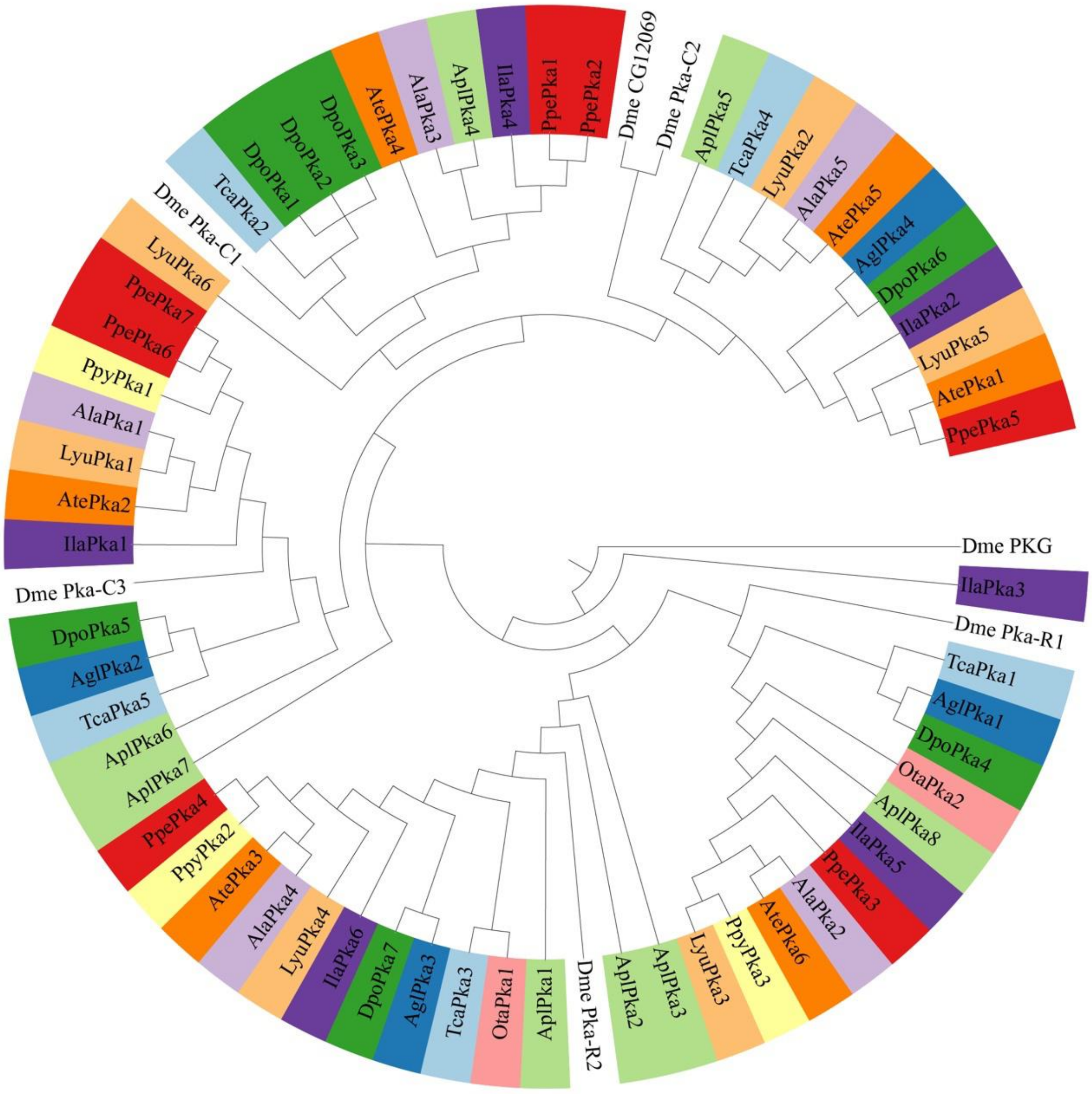

Figure S72. The maximum likelihood (ML) tree of cAMP-dependent protein kinase (PKA) genes in 11 beetles with fruit fly protein kinase G (*CG3324*) (DmePKG) as an outgroup. Lyu: *Lamprigera yunnana*; Ate: *Abscondita terminalis*; Ala: *Aquatica lateralis*; Ppy: *Photinus pyralis*; Ppe: *Pyrocoelia pectoralis*; Ilu: *Ignelater luminosus*; Apl: *Agrilus planipennis*; Ota: *Onthophagus taurus*; Agl: *Anoplophora glabripennis*; Dpo: *Dendroctonus ponderosae*; Tca: *Tribolium castaneum*; Dme: *Drosophila melanogaster*.

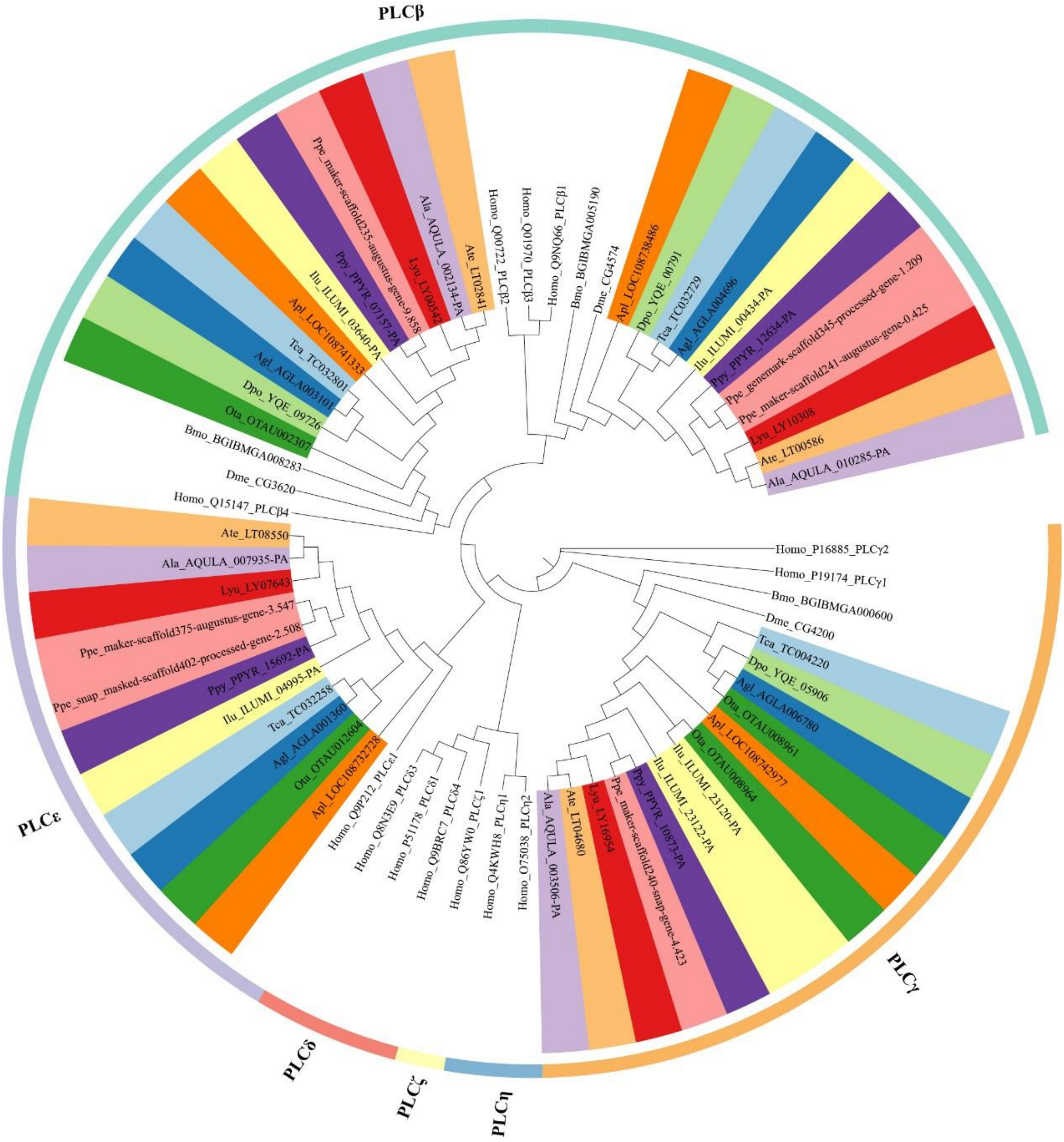

Figure S73. The maximum likelihood (ML) tree of phosphoinositide-specific phospholipase C (PLC) genes in 11 beetles and human. The outer ring showed subfamilies. Lyu: *Lamprigera yunnana*; Ate: *Abscondita terminalis*; Ala: *Aquatica lateralis*; Ppy: *Photinus pyralis*; Ppe: *Pyrocoelia pectoralis*; Ilu: *Ignelater luminosus*; Apl: *Agrilus planipennis*; Ota: *Onthophagus taurus*; Agl: *Anoplophora glabripennis*; Dpo: *Dendroctonus ponderosae*; Tca: *Tribolium castaneum*; Homo: *Homo sapiens.*

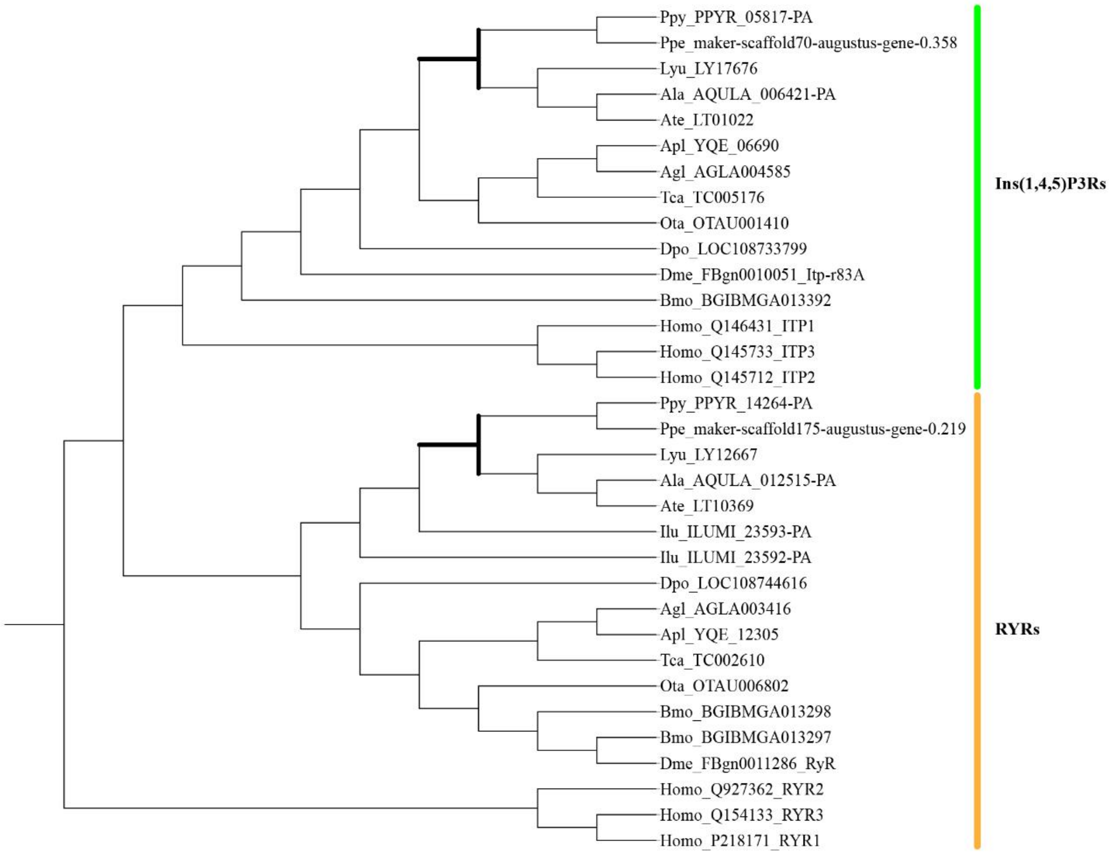

Figure S74. The maximum likelihood (ML) tree of ryanodine receptors (RYRs) and inositol-1,4,5-trisphophate receptors (Ins(1,4,5)P3Rs) genes in 11 beetle species and human. Lyu: *Lamprigera yunnana*; Ate: *Abscondita terminalis*; Ala: *Aquatica lateralis*; Ppy: *Photinus pyralis*; Ppe: *Pyrocoelia pectoralis*; Ilu: *Ignelater luminosus*; Apl: *Agrilus planipennis*; Ota: *Onthophagus taurus*; Agl: *Anoplophora glabripennis*; Dpo: *Dendroctonus ponderosae*; Tca: *Tribolium castaneum*; Homo: *Homo sapiens.*

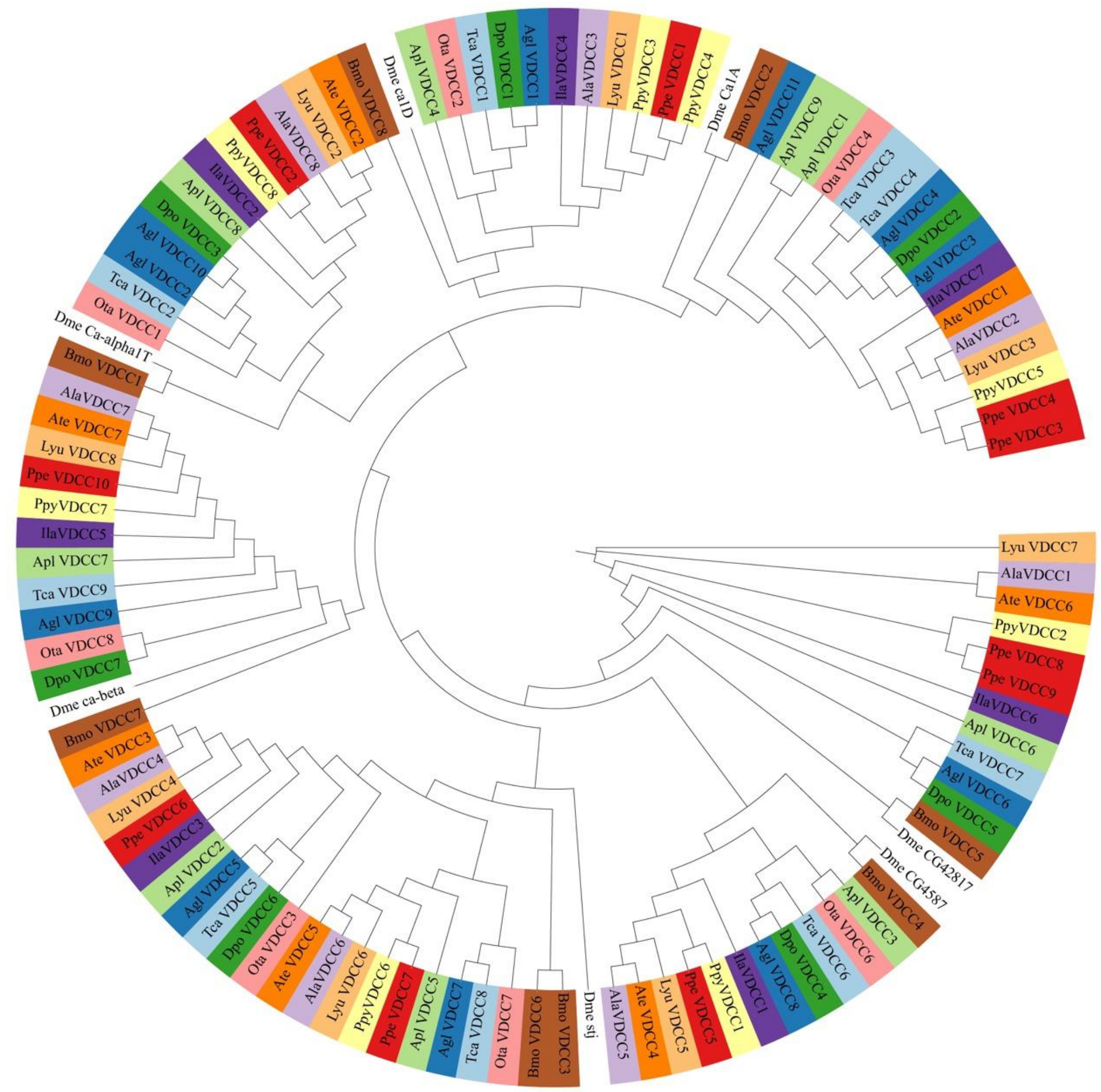

Figure S75. The maximum likelihood (ML) tree of voltage-dependent calcium channel (VDCC) genes in 11 beetles, silkworm and fruit fly. Lyu: *Lamprigera yunnana*; Ate: *Abscondita terminalis*; Ala: *Aquatica lateralis*; Ppy: *Photinus pyralis*; Ppe: *Pyrocoelia pectoralis*; Ilu: *Ignelater luminosus*; Apl: *Agrilus planipennis*; Ota: *Onthophagus taurus*; Agl: *Anoplophora glabripennis*; Dpo: *Dendroctonus ponderosae*; Tca: *Tribolium castaneum*; Bmo: *Bombyx mori*; Dme: *Drosophila melanogaster*.

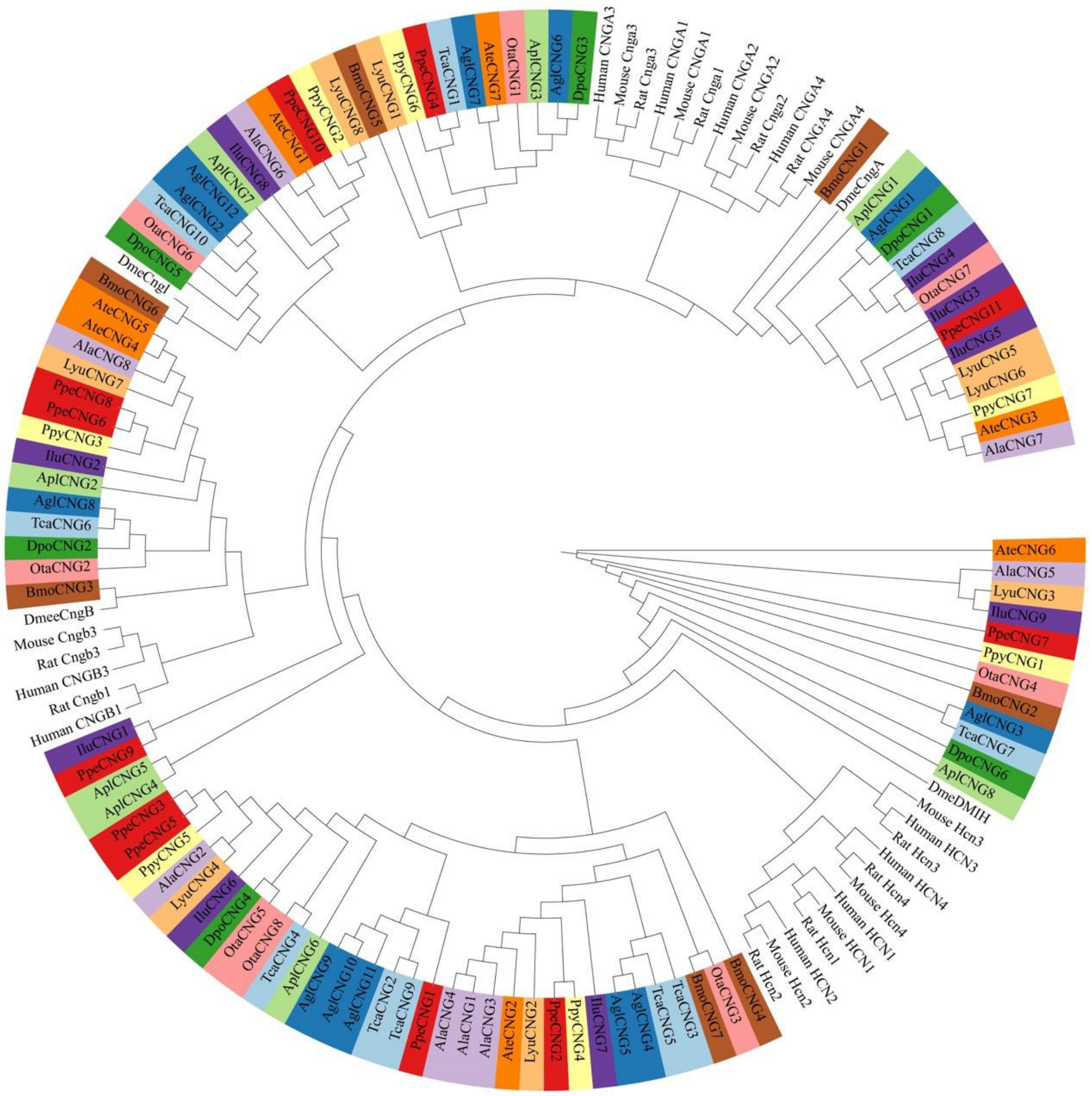

Figure S76. The maximum likelihood (ML) tree of cyclic nucleotide-gated ion channel (CNG) genes in 11 beetles, silkworm and fruit fly. Lyu: *Lamprigera yunnana*; Ate: *Abscondita terminalis*; Ala: *Aquatica lateralis*; Ppy: *Photinus pyralis*; Ppe: *Pyrocoelia pectoralis*; Ilu: *Ignelater luminosus*; Apl: *Agrilus planipennis*; Ota: *Onthophagus taurus*; Agl: *Anoplophora glabripennis*; Dpo: *Dendroctonus ponderosae*; Tca: *Tribolium castaneum*; Bmo: *Bombyx mori*; Dme: *Drosophila melanogaster*.

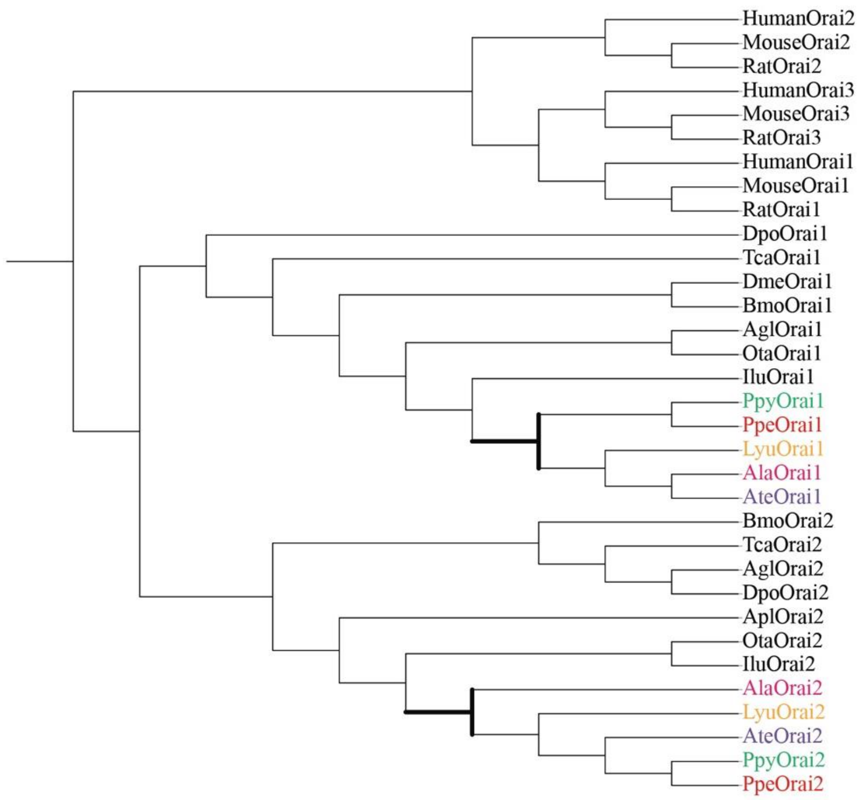

Figure S77. The maximum likelihood (ML) tree of store-operated calcium channel Orai (SOC) genes in 11 beetles, silkworm and fruit fly with the Orai genes of human, rat and mouse as outgroups. Lyu: *Lamprigera yunnana*; Ate: *Abscondita terminalis*; Ala: *Aquatica lateralis*; Ppy: *Photinus pyralis*; Ppe: *Pyrocoelia pectoralis*; Ilu: *Ignelater luminosus*; Apl: *Agrilus planipennis*; Ota: *Onthophagus taurus*; Agl: *Anoplophora glabripennis*; Dpo: *Dendroctonus ponderosae*; Tca: *Tribolium castaneum*; Bmo: *Bombyx mori*; Dme: *Drosophila melanogaster*.

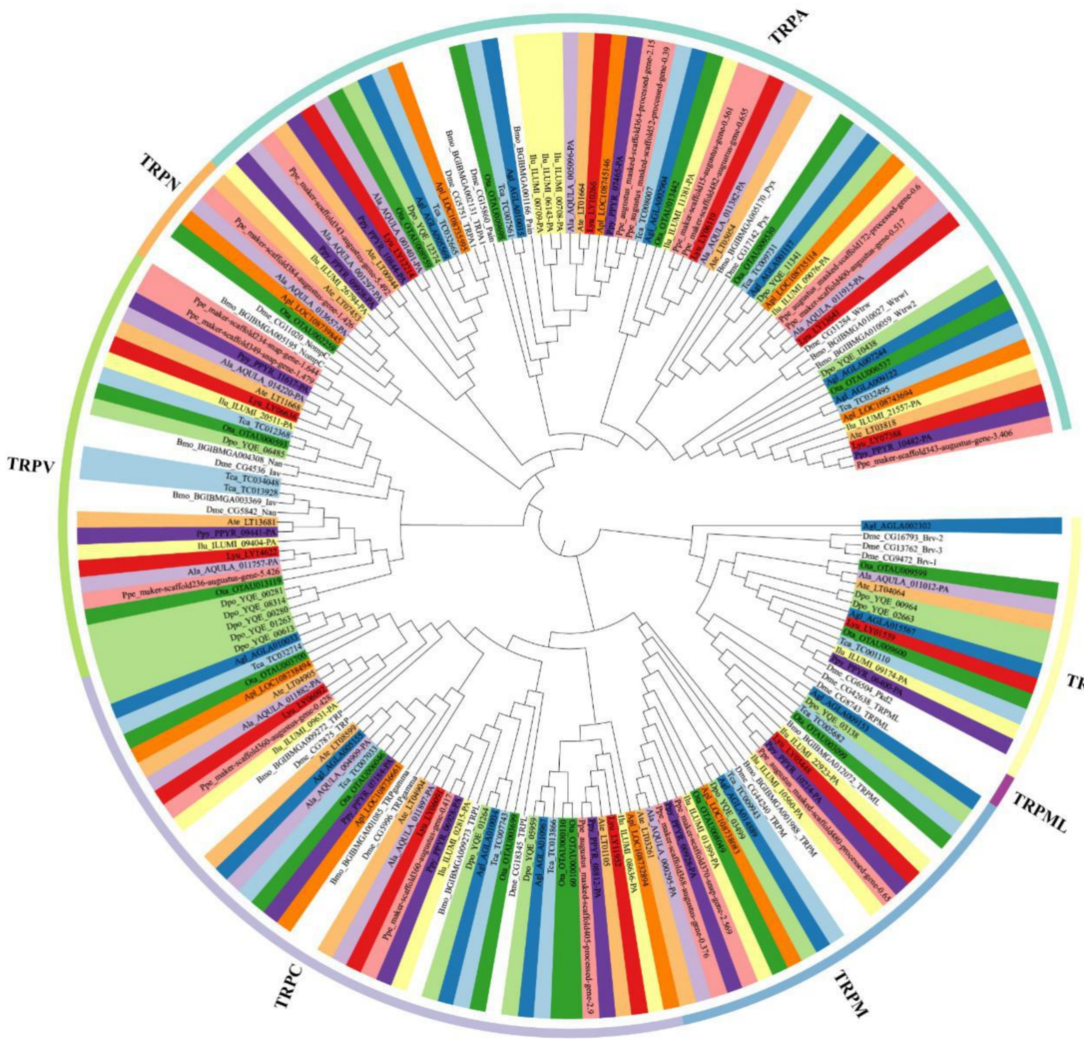

Figure S78. The maximum likelihood (ML) tree of transient receptor potential ion-channel (TRP) genes in 11 beetles, silkworm and fruit fly. The outer ring showed subfamilies (TRPC (canonical), TRPV (vanilloid), TRPM (melastatin), TRPP (polycystin), TRPML (mucolipin), TRPA (ankyrin) and TRPN (NOMPC-like)). Lyu: *Lamprigera yunnana*; Ate: *Abscondita terminalis*; Ala: *Aquatica lateralis*; Ppy: *Photinus pyralis*; Ppe: *Pyrocoelia pectoralis*; Ilu: *Ignelater luminosus*; Apl: *Agrilus planipennis*; Ota: *Onthophagus taurus*; Agl: *Anoplophora glabripennis*; Dpo: *Dendroctonus ponderosae*; Tca: *Tribolium castaneum*; Bmo: *Bombyx mori*; Dme: *Drosophila melanogaster*.

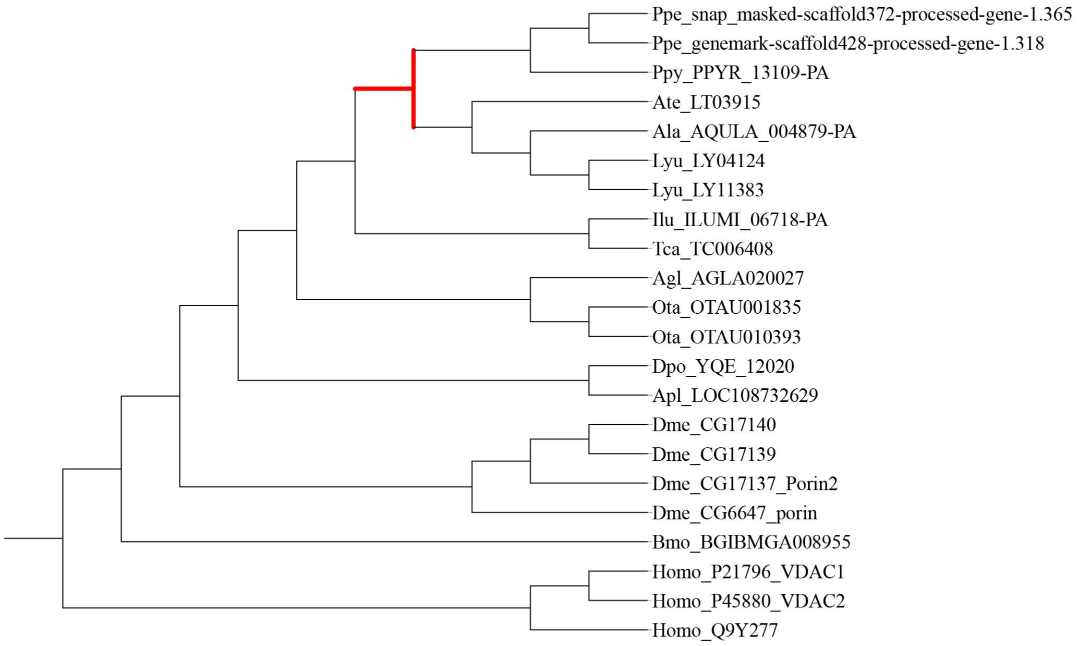

Figure S79. The maximum likelihood (ML) tree of voltage-dependent anion channel (VDAC) genes in in 11 beetles, silkworm and fruit fly with human VDAC genes as the outgroup. Lyu: *Lamprigera yunnana*; Ate: *Abscondita terminalis*; Ala: *Aquatica lateralis*; Ppy: *Photinus pyralis*; Ppe: *Pyrocoelia pectoralis*; Ilu: *Ignelater luminosus*; Apl: *Agrilus planipennis*; Ota: *Onthophagus taurus*; Agl: *Anoplophora glabripennis*; Dpo: *Dendroctonus ponderosae*; Tca: *Tribolium castaneum*; Bmo: *Bombyx mori*; Dme: *Drosophila melanogaster*; Homo: *Homo sapiens.*

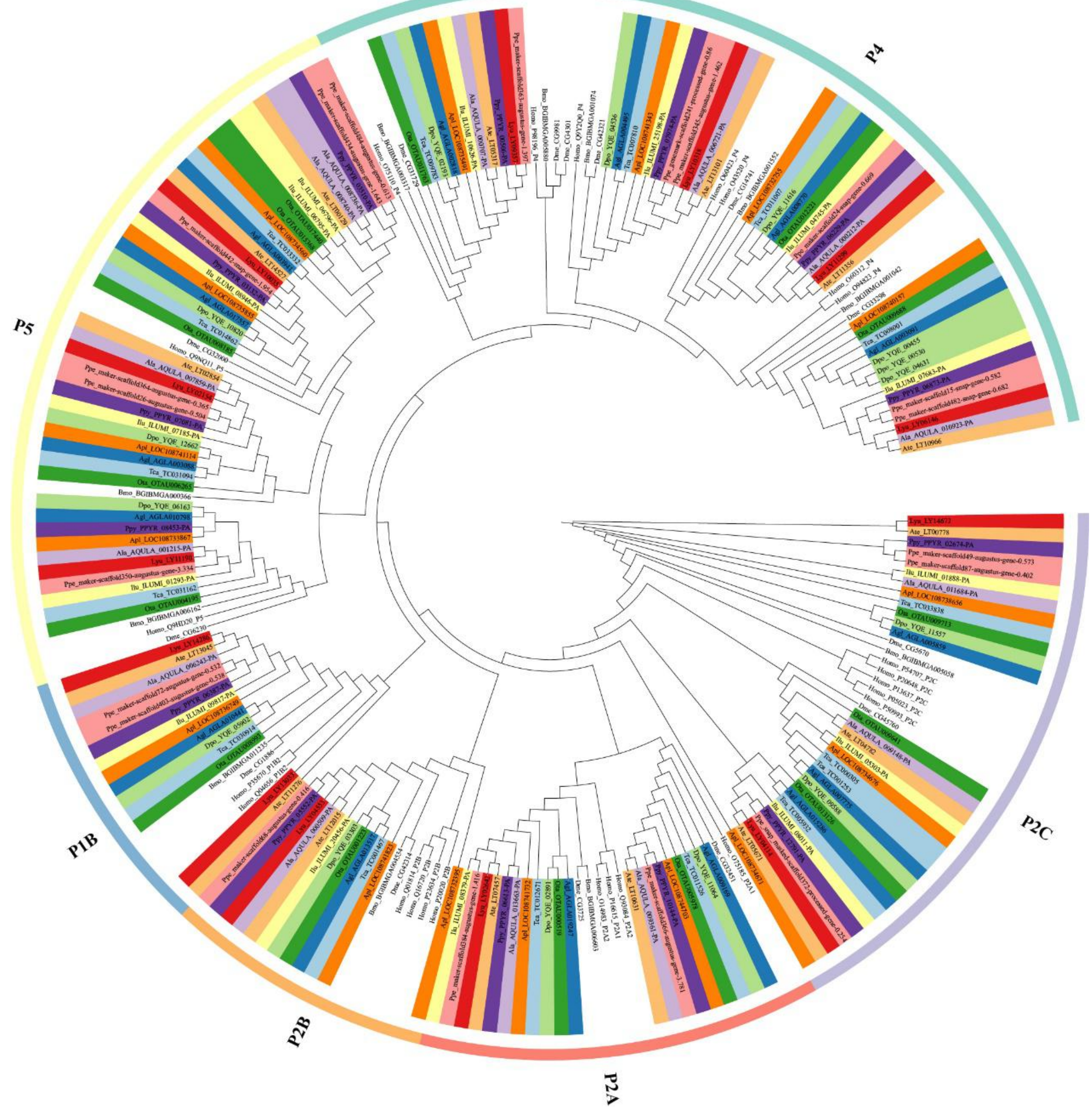

Figure S80. The maximum likelihood (ML) tree of P-type ATPase genes in 11 beetles, silkworm, fruit fly and human. The outer ring showed subfamilies (P1B (Cu^{+}, Ag^{+}, Cu^{2+}, Cd^{2+}, Zn^{2+}, Pb^{2+}, Co^{2+}), P2A (Ca^{2+}, Mn^{2+}; including SERCA pumps), P2B (Ca^{2+}; including plasma membrane Ca^{2+} ATPase (PMCA) pumps), P2C (Na^{+}/K^{+}; H^{+}/K^{+}), P4 (phospholipids ?), and P5 (no assigned specifity)). Lyu: *Lamprigera yunnana*; Ate: *Abscondita terminalis*; Ala: *Aquatica lateralis*; Ppy: *Photinus pyralis*; Ppe: *Pyrocoelia pectoralis*; Ilu: *Ignelater luminosus*; Apl: *Agrilus planipennis*; Ota: *Onthophagus taurus*; Agl: *Anoplophora glabripennis*; Dpo: *Dendroctonus ponderosae*; Tca: *Tribolium castaneum*; Bmo: *Bombyx mori*; Dme: *Drosophila melanogaster*; Homo: *Homo sapiens.*

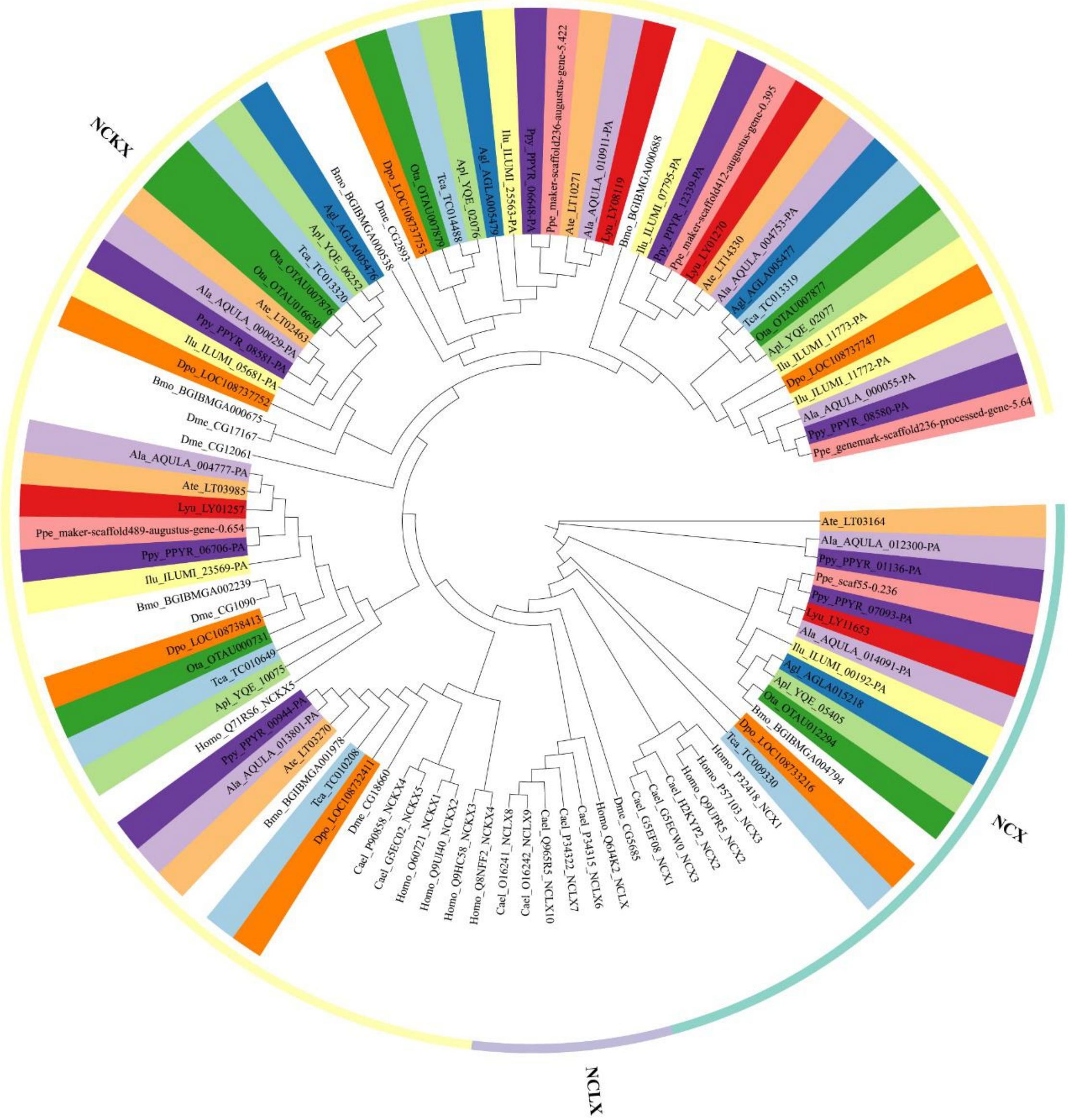

Figure S81. The maximum likelihood (ML) tree of sodium-calcium exchanger (NCX) genes in 11 beetles, silkworm, fruit fly, nematode and human. NCX: Na^{+}/Ca^{2+} exchangers, NCKX: $Na^{+}/Ca^{2+}/K^{+}$ exchangers, NCLX: Ca^{2+}/Cation exchangers. Lyu: *Lamprigera yunnana*; Ate: *Abscondita terminalis*; Ala: *Aquatica lateralis*; Ppy: *Photinus pyralis*; Ppe: *Pyrocoelia pectoralis*; Ilu: *Ignelater luminosus*; Apl: *Agrilus planipennis*; Ota: *Onthophagus taurus*; Agl: *Anoplophora glabripennis*; Dpo: *Dendroctonus ponderosae*; Tca: *Tribolium castaneum*; Bmo: *Bombyx mori*; Dme: *Drosophila melanogaster*; Cael: *Caenorhabditis elegans*; Homo: *Homo sapiens*.

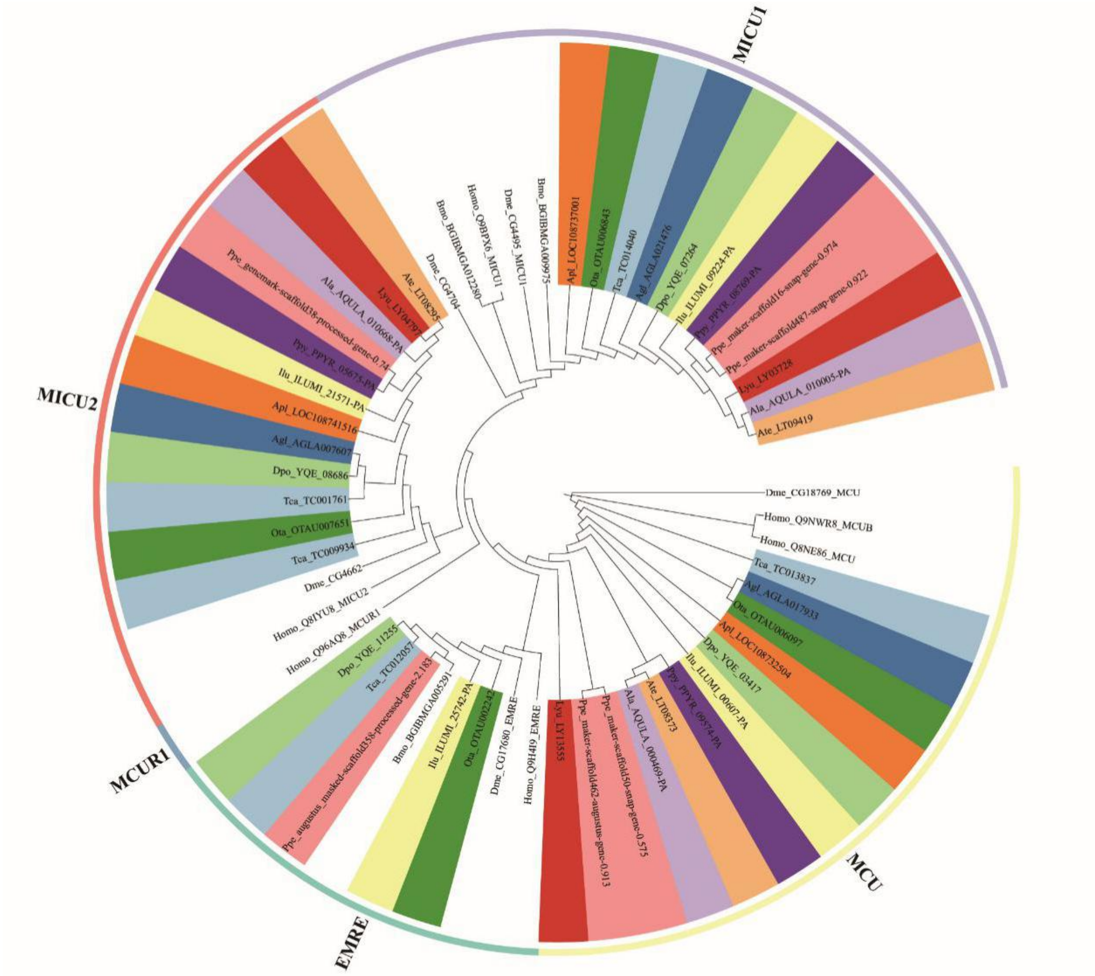

Figure S82. The maximum likelihood (ML) tree of calcium uniporter (UP) genes in in 11 beetles, silkworm, fruit fly, and human. MCU: mitochondrial calcium uniporter, MICU1: Calcium Uptake 1, MICU2: Mitochondrial Calcium Uptake 2, EMRE: Essential MCU Regulator, MCUR1: Mitochondrial Calcium Uniporter Regulator 1. Lyu: *Lamprigera yunnana*; Ate: *Abscondita terminalis*; Ala: *Aquatica lateralis*; Ppy: *Photinus pyralis*; Ppe: *Pyrocoelia pectoralis*; Ilu: *Ignelater luminosus*; Apl: *Agrilus planipennis*; Ota: *Onthophagus taurus*; Agl: *Anoplophora glabripennis*; Dpo: *Dendroctonus ponderosae*; Tca: *Tribolium castaneum*; Bmo: *Bombyx mori*; Dme: *Drosophila melanogaster*; Homo: *Homo sapiens.*

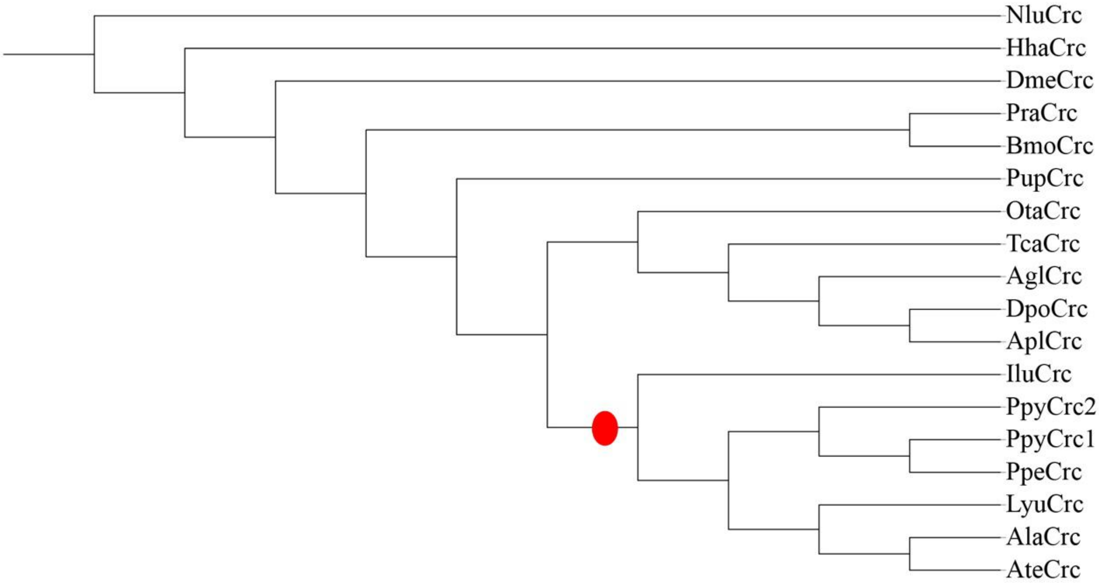

Figure S83. The maximum likelihood (ML) tree of calreticulin (Crc) genes in insect species with that of *Nilaparvata lugens* (NluCrc) as an outgroup. The red circle shows the clade of luminous species. Lyu: *Lamprigera yunnana*; Ate: *Abscondita terminalis*; Ala: *Aquatica lateralis*; Ppy: *Photinus pyralis*; Ppe: *Pyrocoelia pectoralis*; Ilu: *Ignelater luminosus*; Apl: *Agrilus planipennis*; Dpo: *Dendroctonus ponderosae*; Agl: *Anoplophora glabripennis*; Tca: *Tribolium castaneum*; Ota: *Onthophagus taurus*; Bmo: *Bombyx mori*; Pra: *Pieris rapae*; Pup: *Pteromalus puparum*; Dme: *Drosophila melanogaster*; Hha: *Halyomorpha halys*; Nlu: *Nilaparvata lugens*.

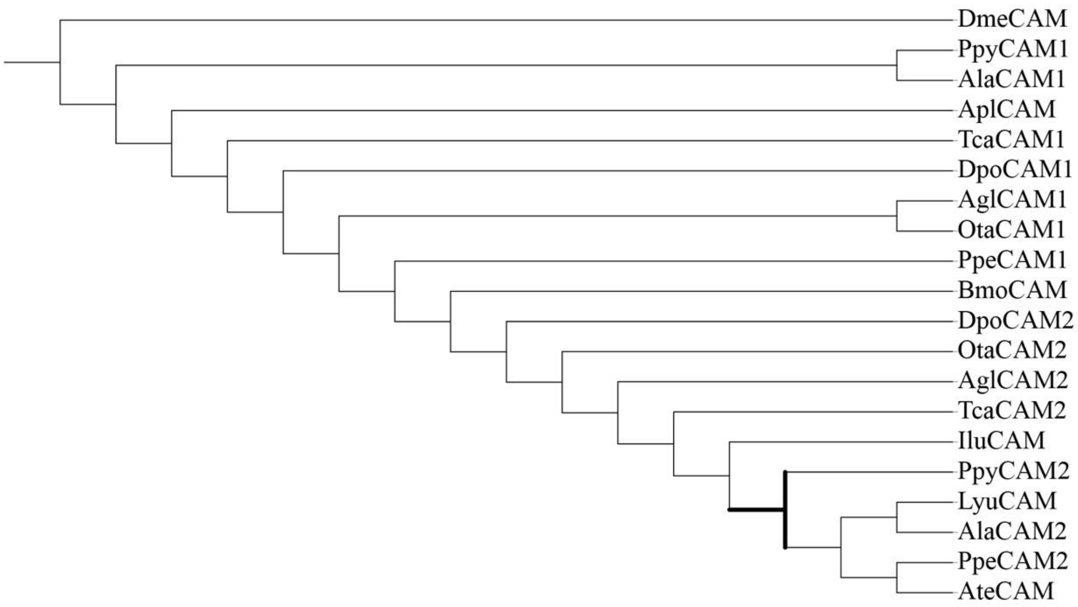

Figure S84. The maximum likelihood (ML) tree of calmodulin (CAM) genes in insect species with that of fruit fly (DmeCAM) as an outgroup. The black highlighted shows the clade of Lampyridae. Lyu: *Lamprigera yunnana*; Ate: *Abscondita terminalis*; Ala: *Aquatica lateralis*; Ppy: *Photinus pyralis*; Ppe: *Pyrocoelia pectoralis*; Ilu: *Ignelater luminosus*; Apl: *Agrilus planipennis*; Ota: *Onthophagus taurus*; Agl: *Anoplophora glabripennis*; Dpo: *Dendroctonus ponderosae*; Tca: *Tribolium castaneum*; Bmo: *Bombyx mori*; Dme: *Drosophila melanogaster*.

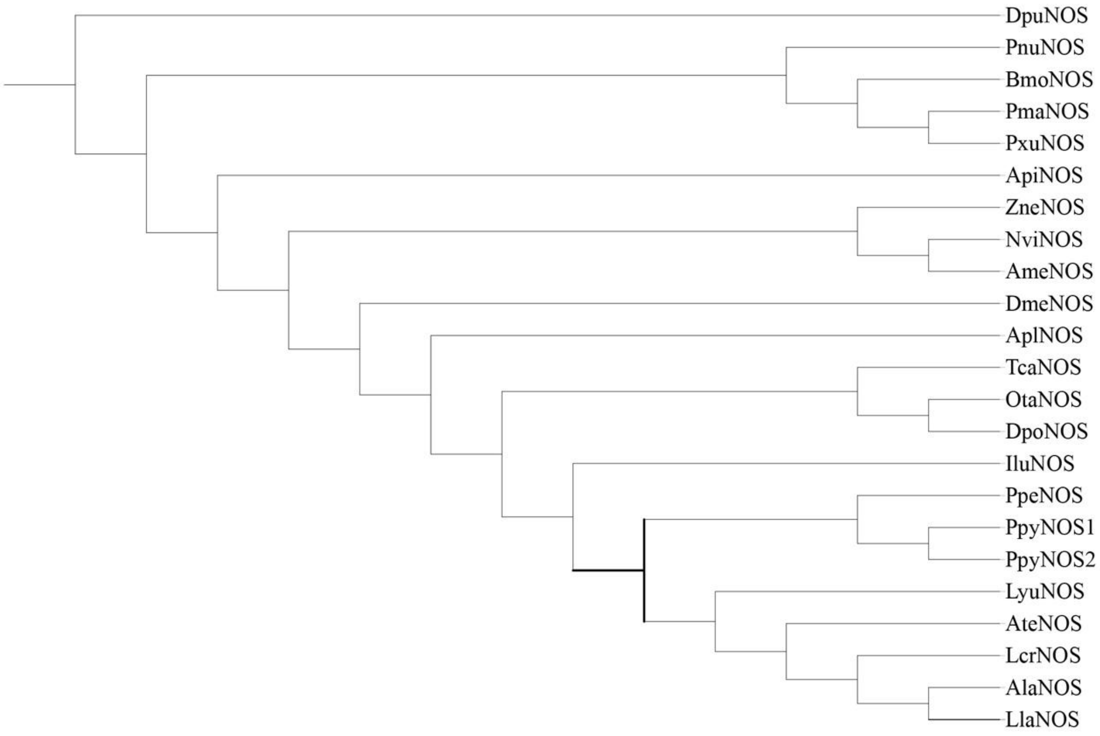

Figure S85. The maximum likelihood (ML) tree of nitric oxide synthase (NOS) genes in 22 insect species with that of one crustacea (DpuNOS) as an outgroup. The black highlighted shows the clade of Lampyridae. Lyu: *Lamprigera yunnana*; Ate: *Abscondita terminalis*; Ala: *Aquatica lateralis*; Ppy: *Photinus pyralis*; Ppe: *Pyrocoelia pectoralis*; Ilu: *Ignelater luminosus*; Apl: *Agrilus planipennis*; Ota: *Onthophagus taurus*; Agl: *Anoplophora glabripennis*; Dpo: *Dendroctonus ponderosae*; Tca: *Tribolium castaneum*; Dme: *Drosophila melanogaster*; Bmo: *Bombyx mori*; Pxu: *Papilio xuthus*; Pma: *Papilio machaon*; Ame: *Apis mellifera*; Nvi: *Nasonia vitripennis*; Api: *Acyrthosiphon pisum*; Phu: *Pediculus humanus;* Zne: *Zootermopsis nevadensis*; Dpu: *Daphnia pulex*.

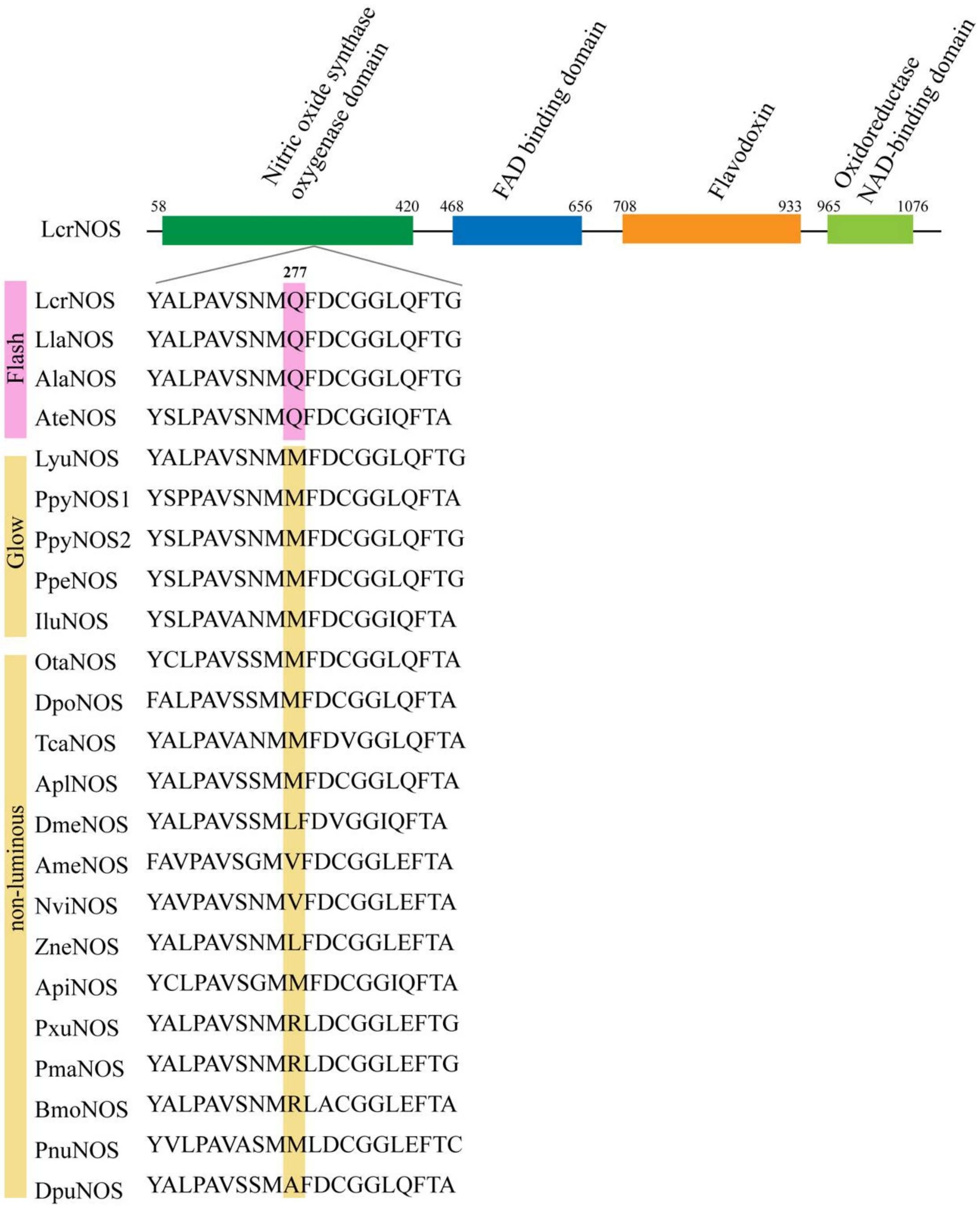

Figure S86. The specific amino acid of nitric oxide synthase (NOS) in the flash firefly species. The amino acid mutation (277Q) were shared at PF02898 (Nitric oxide synthase oxygenase domain) in flash firefly species, while no Q at same position in glow and non-luminous species. And also, we compare all NOS sequences of NR database and don’t find Q in same position (showed in Supplementary Data 35). The positions of the substituted amino acid are numbered based on the cloned NOS protein sequence from *Luciola cruciata* (Lcr).

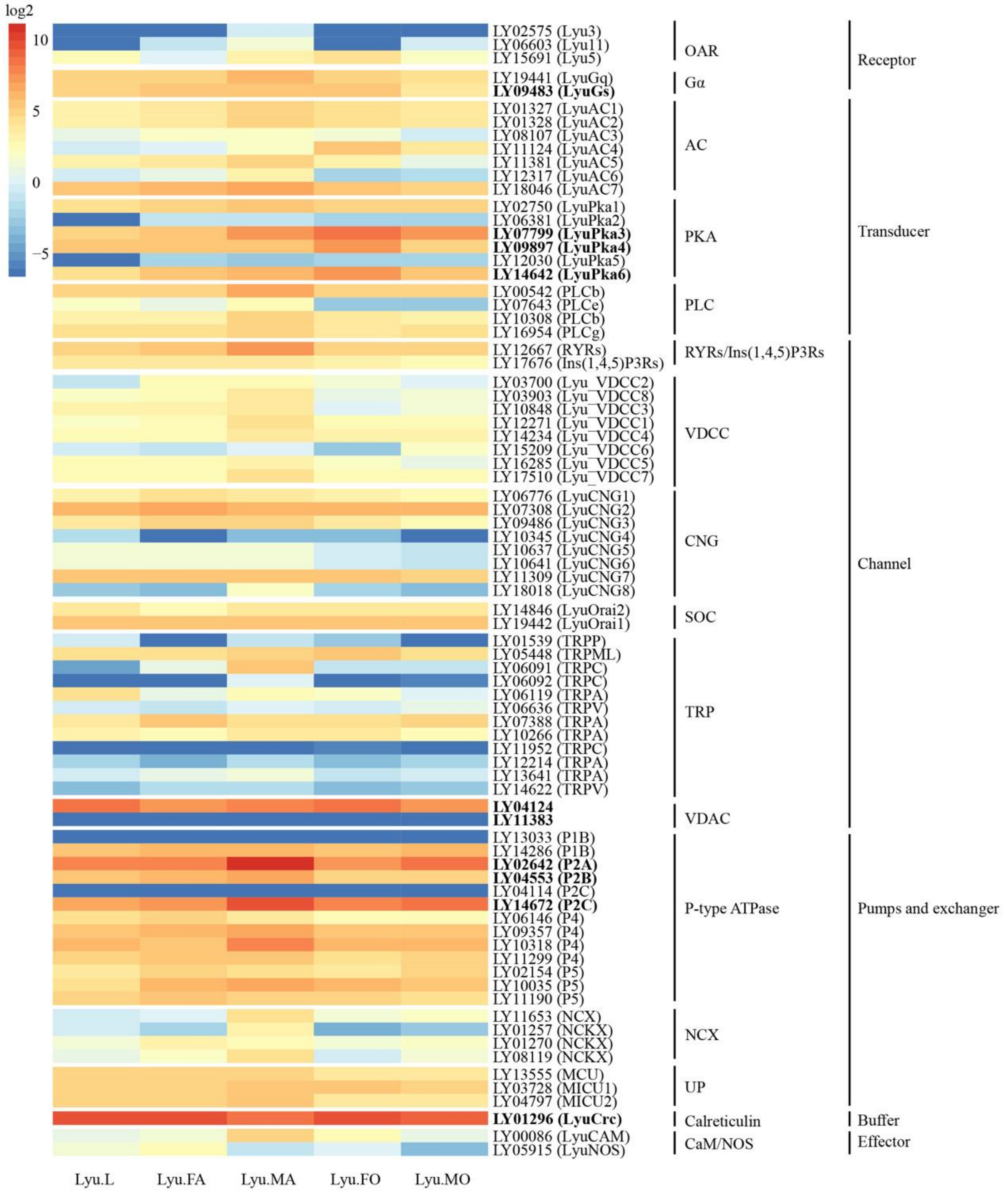

Figure S87. The expression of genes in the Ca^{2+} signal system in *L. yunnan* (Lyu) at transcriptome level. The heat map shows log2-scaled fragments per kilobase of transcript per million fragments (FPKM). MO: luminous organ of male adult; FO: luminous organ of female adult; MA: male adult; FA: female adult; L: larva. Those genes with abundance at proteomic level were marked as bold. R package was used for the visualization of the image.

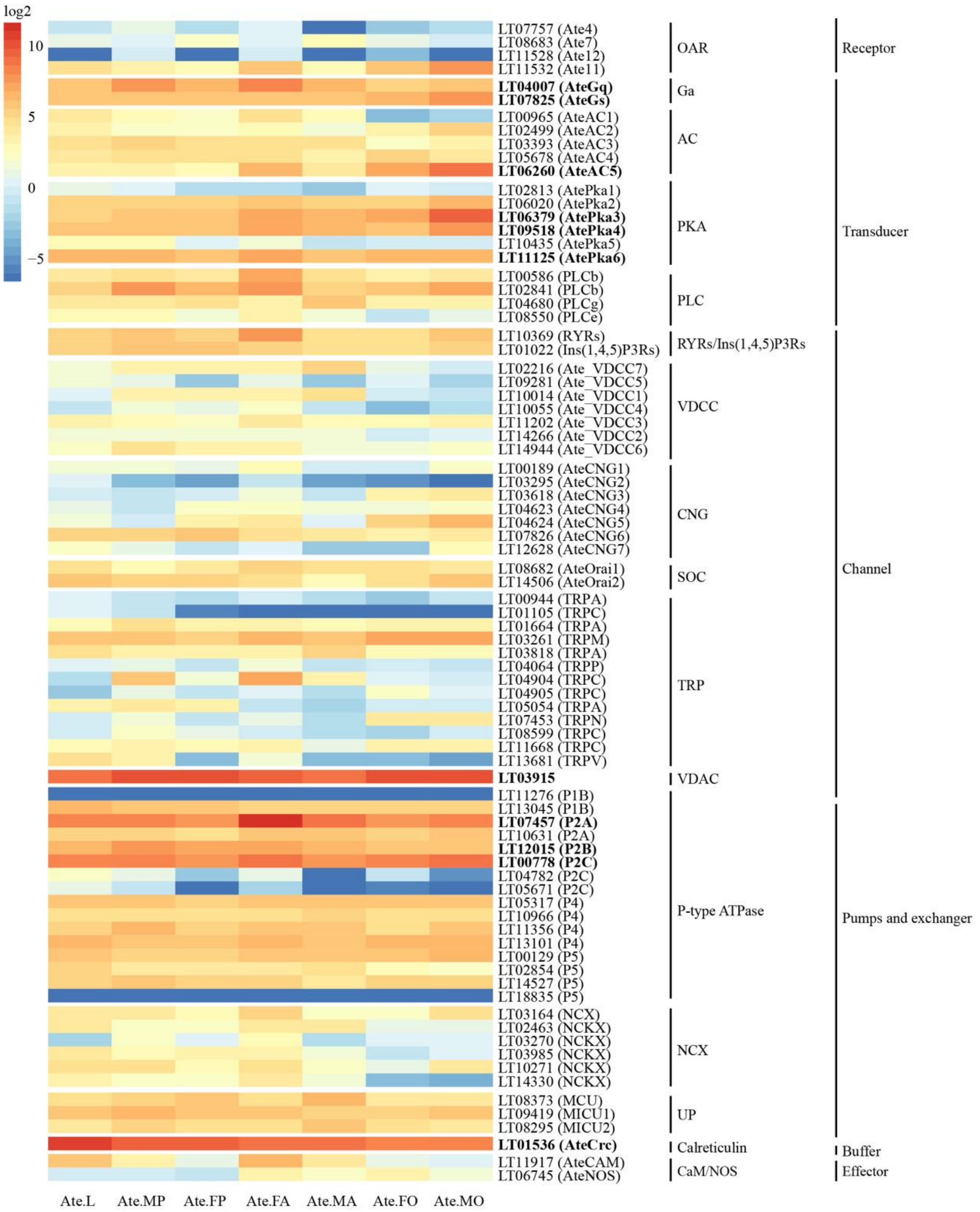

Figure S88. The expression of genes in the Ca^{2+} signal system in *A. terminalis* (Ate) at transcriptome level. The heat map shows log2-scaled fragments per kilobase of transcript per million fragments (FPKM). Those genes with abundance at proteomic level were marked as bold. MO: luminous organ of male adult; FO: luminous organ of female adult; MA: male adult; FA: female adult; MP: male pupa; FP: female pupa; L: larva. R package was used for the visualization of the image.

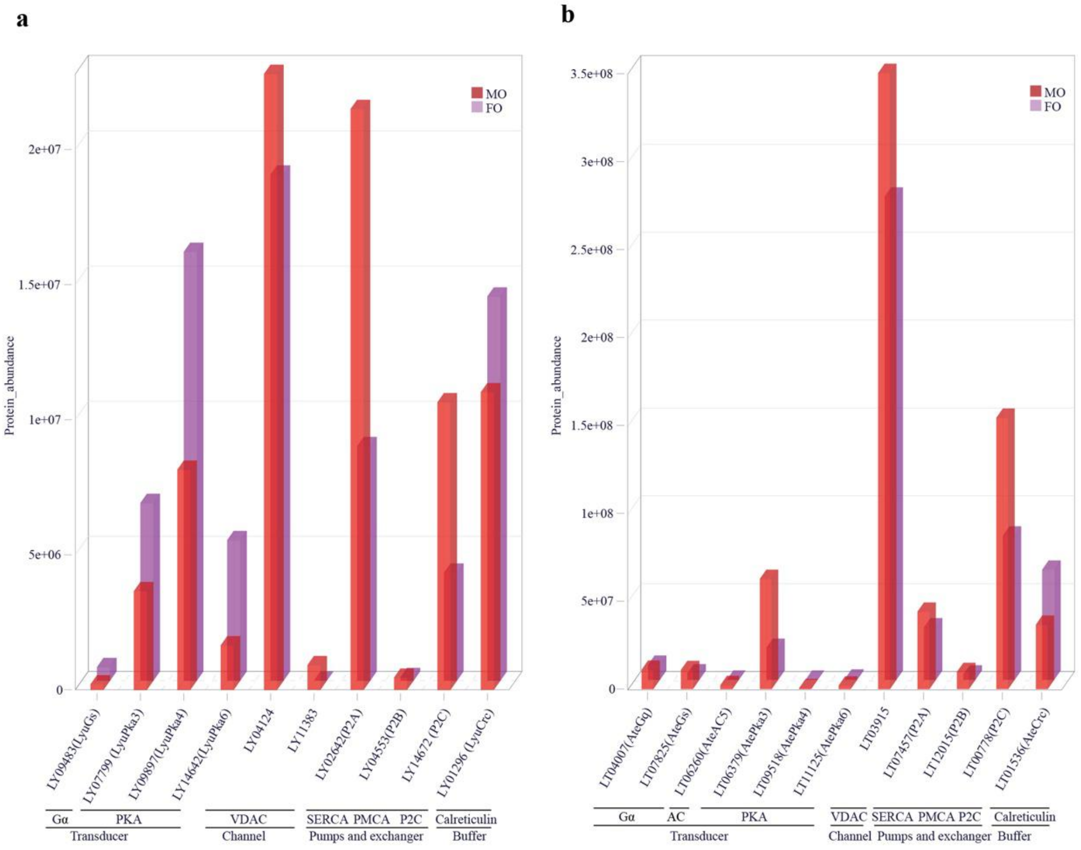

Figure S89. The expression of genes in the Ca^{2+} signal system in the luminous organs of *L. yunnan* (Lyu) and *A. terminalis* (Ate) at proteomic level. a represents *L. yunnan*. **b** represents *A. terminalis*. Transducer: Gα (guanine nucleotide-binding protein alpha subunit), PKA (cAMP-dependent protein kinase); Channel: VDAC (voltage-dependent anion channel); Pumps and exchanger: SERCA (sarcoplasmic/endoplasmic reticulum (SR/ER) calcium transporting ATPase), PMCA (plasma membrane Ca^{2+}-ATPase), P2C (Na^+/K^+; H^+/K^+); Buffer: Calreticulin. MO: luminous organ of male adult; FO: luminous organ of female adult. R package was used for the visualization of the images.

Supplementary Tables

Table S1. Summary of sequencing data from Illumina platform for two fireflies *L. yunnana* (Lyu) and *A. terminalis* (Ate).

Species	Raw reads	Raw base (Gb)	Clean reads	Clean base (Gb)	Sequence coverage (×) *
Lyu	351,877,392	105.56	350,512,995	105.15	96.02
Ate	349,610,777	104.88	320,734,911	91.74	146.46

* Sequence coverage was calculated based on the genome size of *k-mer* frequency analyses.

Table S2. *K*-mer (17-mer) frequency and genome size evaluation based on Illumina short reads from 350 bp library for two fireflies *L. yunnana* (Lyu) and *A. terminalis* (Ate).

Species	Kmer_num	Peak depth	Genome size (Mb)	Used base (bp)	Used read	Sequence coverage (×)	Heterozygosity (%)
Lyu	58,033,958,724	53	1094.98	69,088,046,100	690,880,461	63.10	1.11
Ate	57,627,093,804	92	626.38	68,603,683,100	686,036,831	109.52	1.52

Table S3. Statistics of PacBio RS II sequencing data for two fireflies *L. yunnana* (Lyu) and *A. terminalis* (Ate).

Species	Total read number	Raw reads N50 (kb)	Raw bases (Gb)	Average length (kb)	Max length (bp)	Sequence coverage (×) *
Lyu	9,983,653	11.45	81.08	8.12	56,012	74.05
Ate	5,872,725	11.11	42.21	7.19	54,388	67.39

* Sequence coverage was calculated based on the genome size of *k-mer* frequency analyses.

Table S4. Summary of *de novo* assembly using PacBio data for two fireflies *L. yunnana* (Lyu) and *A. terminalis* (Ate).

Stastitics	Lyu		Ate	
	**Size (Mb)**	**Number**	**Size (Mb)**	**Number**
N90	0.56	344	0.06	672
N80	1.28	224	0.31	338
N70	2.04	158	0.62	221
N60	2.70	113	0.90	155
N50	3.51	79	1.21	106
Longest	16.46	-	5.99	-
Average length	0.39	-	0.16	-
Total size	1053	-	501	-
Total number (≥1 kb)	-	2,685	-	3,192
Total number (≥2 kb)	-	2,673	-	3,191

Table S5. Statistics of Illumina short reads mapped to the assembled genomes of *L. yunnana* (Lyu) and *A. terminalis* (Ate). PE mapped reads represent reads mapped to the genome as read pairs.

Libraries	Insert size (bp)	Total reads	Mapped reads	Mapped ratio (%)	PE mapped reads	PE mapped ratio (%)
Lyu	350	712,000,530	702,929,056	98.58	645,312,946	92.06
Ate	350	706,128,409	679,420,013	98.51	621,356,958	89.95

Table S6. Evaluation on the completeness of assemblies of *L. yunnana* (Lyu), *A. terminalis* (Ate) and *Pyrocoelia pectorolis* (Ppe) by Benchmarking Universal Sing-Copy Orthologs (BUSCO) with Insecta_odb9 Dataset.

	Lyu		Ate		Ppe	
	Numbers	Ratio (%)	Numbers	Ratio (%)	Numbers	Ratio (%)
Complete BUSCOs (C)	1636	98.6	1622	97.8	1637	98.7
Complete and single-copy BUSCOs (S)	1612	97.2	1579	95.2	1028	62.0
Complete and duplicated BUSCOs (D)	24	1.4	43	2.6	609	36.7
Fragmented BUSCOs (F)	6	0.4	20	1.2	12	0.7
Missing BUSCOs (M)	16	1	16	1	9	0.6
Total BUSCO groups searched	1658	-	1658	-	1658	-

Table S7. Statistics of transcriptomic reads mapped to the assembled genomes of of *L. yunnana* (Lyu) and *A. terminalis* (Ate).

Species	Stages	Tissue	Libraries	Reads mapping rate (%)
Lyu	Male adult	whole body	LgY23	91.8
	Female adult	whole body	LgY10	92.0
	Larva	whole body	Lg-YL7	91.6
Ate	Male adult	whole body	Lo-TM37	83.0
	Female adult	whole body	Lo-TM46	88.8
	Female pupa	whole body	Lo-TPF	82.5
	Male pupa	whole body	Lo-TPM	81.6
	Larva	whole body	Lo-TL1-1	83.4
	Larva	whole body	Lo-TL1-2	84.4

Table S8. Statistics of repeats in the assembled genomes of *L. yunnana* (Lyu), *A. terminalis* (Ate) and other three fireflies with the reference genomes published (*Aquatica lateralis* (Ala), *Photinus pyralis* (Ppy), *Pyrocoelia pectoralis* (Ppe)).

**Type**	**Lyu**		**Ate**		**Ala**		**Ppy**		**Ppe**	
	Repeat size (bp)	% of genome	Repeat size (bp)	% of genome	Repeat size (bp)	% of genome	Repeat size (bp)	% of genome	Repeat size (bp)	% of genome
De novo	623,799,611	59.24	139,908,597	27.91	230,841,230	27.86	197,968,522	41.99	306,993,787	40.37
Repeatmasker	57,063,830	5.42	8,747,672	1.74	17,696,555	1.95	5,285,306	1.12	4,895,541	0.64
Proteinmask	232,943,682	22.12	52,379,961	10.45	17,421,608	1.92	40,640,832	8.62	95,721,204	12.59
TRF	40,809,714	3.88	23,785,375	4.74	6,136,408	0.68	19,615,677	4.16	28,179,344	3.71
**Total**	**701,490,526**	**66.62**	**183,317,571**	**36.54**	**253,110,695**	**27.86**	**22,4928,828**	**47.70**	**358,972,887**	**47.21**

Table S9. Transposable elements (TEs) content in the assembled genomes of of *L. yunnana* (Lyu), *A. terminalis* (Ate), and three other fireflies with the reference genomes published (*Aquatica lateralis* (Ala), *Photinus pyralis* (Ppy), *Pyrocoelia pectoralis* (Ppe)) identified by different methods.

Species	Type	Repbase TEs		TE proteins		*De novo*		Combined TEs	
		Length(bp)	% in genome	Length (bp)	% in genome	Length (bp)	% in genome	Length (bp)	% in genome
Lyu	DNA	37586343	3.569570	127765855	12.133905	333328959	31.656204	367551516	34.906316
	LINE	6869318	0.652378	65992513	6.267300	141044661	13.394992	162115182	15.396056
	SINE	38013	0.003610	0	0.000000	2166335	0.205737	2204348	0.209347
	LTR	13411378	1.273677	39750358	3.775086	1766904	0.167803	1766904	0.167803
	Other	1382	0.000131	0	0.000000	0	0.000000	1382	0.000131
	Unknown	0	0.000000	0	0.000000	18297342	1.737696	0	0.000000
	**Total**	**57063830**	**5.419344**	**232943682**	**22.122629**	**623230560**	**59.188118**	**688292463**	**65.367038**
Ate	DNA	2839291	0.566317	18230049	3.636115	76189599	15.196566	88325208	17.617101
	LINE	2206067	0.440016	22068765	4.401775	39941303	7.966581	49456973	9.864550
	SINE	16648	0.003321	0	0.000000	698687	0.139358	715335	0.142679
	LTR	3736938	0.745359	12179369	2.429263	4638621	0.925206	4638621	0.925206
	Other	60	0.000012	0	0.000000	0	0.000000	60	0.000012
	Unknown	0	0.000000	0	0.000000	5450638	1.087169	0	0.000000
	**Total**	**8747672**	**1.744786**	**52379961**	**10.447562**	**139802186**	**27.884556**	**169247169**	**33.757570**
Ala	DNA	6558939	0.721893	13906569	1.530592	120487530	13.261163	134817777	14.838386
	LINE	2134389	0.234916	10265972	1.129899	57880893	6.370518	67707232	7.452030
	SINE	11057	0.001217	0	0.000000	1173052	0.129109	1184109	0.130326

	LTR	9002078	0.990792	10632278	1.170215	10438364	1.148873	2704957	0.297714
	Other	177	0.000019	0	0.000000	0	0.000000	177	0.000019
	Unknown	0	0.000000	0	0.000000	9323415	1.026159	0	0.000000
	**Total**	**17696555**	**1.947728**	**17421608**	**1.917466**	**230705592**	**25.392042**	**249489409**	**27.459437**
Ppy	DNA	1813482	0.384611	11712543	2.484043	109595067	23.243362	118270813	25.083349
	LINE	1162698	0.246590	16774305	3.557562	31820721	6.748666	40283641	8.543516
	SINE	21239	0.004504	0	0.000000	1728937	0.366680	1748154	0.370756
	LTR	2323283	0.492731	12490078	2.648946	9967599	2.113968	9967599	2.113968
	Other	0	0.000000	0	0.000000	0	0.000000	0	0.000000
	Unknown	0	0.000000	0	0.000000	9853553	2.089781	9853553	2.089781
	**Total**	**5285306**	**1.120929**	**40640832**	**8.619271**	**195952122**	**41.558313**	**218446960**	**46.329109**
Ppe	DNA	1811035	0.238164	33931821	4.462270	196564941	25.849655	216259419	28.439616
	LINE	1015533	0.133550	40267134	5.295408	55871362	7.347472	74408155	9.785189
	SINE	4779	0.000628	0	0.000000	1503300	0.197694	1508079	0.198323
	LTR	2070975	0.272348	21724372	2.856906	13117000	1.724977	1325666	0.174334
	Other	0	0.000000	0	0.000000	0	0.000000	0	0.000000
	Unknown	0	0.000000	0	0.000000	12683902	1.668021	0	0.000000
	**Total**	**4895541**	**0.643798**	**95721204**	**12.588003**	**306904780**	**40.360111**	**350008557**	**46.028557**

Table S10. Statistics of predicted protein-coding genes in the assembled genomes of of *L. yunnana* (Lyu) and *A. terminalis* (Ate).

Species	Gene set	Gene number	Average mRNA length (bp)	Total exon number	Average exon length (bp)	Average cds length (bp)	Average exon number	Total intron length (bp)
Lyu	Augustus	20040	11242.168	93751	292.295	1367.413	4.678	197,890,086
	Dme	10986	8031.403	44685	277.418	1128.382	4.067	75,836,593
	Agl	17522	8230.714	69707	293.632	1168.143	3.978	123,750,374
	Dpo	13620	9391.321	59263	282.051	1227.253	4.351	111,194,614
	Nvi	6916	9888.201	30870	279.444	1247.314	4.464	59,760,373
	Tca	14369	10237.814	64695	289.026	1301.312	4.502	128,408,595
	PASA	51966	7906.887	95162	391.011	716.034	1.831	373,679,917
	Transdecoder	14627	22084.760	42974	358.211	1052.421	2.938	193,915,699
	BUSCO	125	2417.088	350	259.56	726.768	2.8	211,290
	**EVM + PASA**	**19443**	**16619.390**	**92188**	**290.556**	**1377.869**	**4.742**	**265,352,354**
Ate	Augustus	21379	5598.328	101870	312.137	1487.318	4.765	87,889,289
	Dme	12272	4701.537	47562	285.308	1105.755	3.876	44,127,438
	Agl	17642	5037.857	70114	298.077	1184.637	3.974	67,978,512
	Dpo	14815	5515.681	61735	291.178	1213.357	4.167	63,738,940
	Nvi	14755	5823.259	64716	288.873	1267.010	4.386	67,227,455
	Tca	15569	6203.200	67809	294.585	1283.030	4.355	76,602,123
	PASA	56462	5251.655	94178	411.046	685.621	1.668	257,807,421
	Transdecoder	11932	17094.119	43293	314.940	1142.700	3.628	115,384,942
	BUSCO	197	1773.914	439	274.380	611.437	2.228	229,008
	**EVM + PASA**	**21024**	**7767.975**	**100194**	**311.203**	**1483.382**	**4.767**	**121,305,613**

Table S11. The evaluation on the completeness of proteins for the predicted genes in the assembled genomes of *L. yunnana* (Lyu) and *A. terminalis* (Ate) by Benchmarking Universal Sing-Copy Orthologs (BUSCO) with Insecta_odb9 Dataset.

	**Lyu**		**Ate**	
	**Numbers**	**Ratio (%)**	**Numbers**	**Ratio (%)**
Complete BUSCOs (C)	1596	**96.3**	1609	**97.0**
Complete and single-copy BUSCOs (S)	1562	94.2	1547	93.3
Complete and duplicated BUSCOs (D)	34	2.1	62	3.7
Fragmented BUSCOs (F)	34	2.1	27	1.6
Missing BUSCOs (M)	28	1.6	22	1.4
Total BUSCO groups searched	1658	-	1658	-

Table S12. Comparisons of gene features among fireflies (Lyu, Ate, Ala, Ppy, Ppe), other beetles (Ilu, Agl, Dpo, Tca) and fruit fly (Dme).

**Species ***	**Total genes number**	**Average gene length (bp)**	**Average CDS length (bp)**	**Average exons per gene**	**Average exon length (bp)**	**Average intron length (bp)**
Dme	13,918	4489.22	1610.35	3.96	406.64	972.89
Tca	16,590	5726.09	1359.37	4.62	294.46	1207.71
Dpo	13,088	3868.39	1262.69	5.9	214.11	532.26
Agl	22,253	7414.48	1131.02	4.52	250.08	1784.03
Ilu	27,557	5372.50	1071.33	3.96	270.34	1266.76
Ppe	23,092	4689.19	1384.32	5.40	256.08	750.35
Ppy	15,773	8112.91	1489.20	5.15	289.09	1595.49
Ala	14,284	15055.18	1368.68	4.94	277.28	3268.66
Ate	21,024	7767.31	1483.31	4.77	311.20	1532.06
Lyu	19,443	16618.92	1377.84	4.74	290.54	3647.36

* fireflies: *L. yunnana* (Lyu), *A. terminalis* (Ate), *A. lateralis* (Ala), *P. pyralis* (Ppy), *P. pectoralis* (Ppe); other beetles (*I. luminosus* (Ilu), *A. glabripenni* (Agl), *D. ponderosae* (Dpo), *T. castaneum* (Tca); fruit fly (*D. melanogaster* (Dme)).

Table S13. Summary of gene function annotation of the assembled genomes of *L. yunnana* (Lyu) and *A. terminalis* (Ate).

	**Lyu**		**Ate**	
	**Number**	**Percentage (%)**	**Number**	**Percentage (%)**
Total	19443	-	21024	-
InterPro	13016	66.954733	15987	76.056137
GO	10434	53.67284	12655	60.204567
KEGG	10411	53.554527	12956	61.636537
Swissprot	11633	59.840535	14062	66.898192
TrEMBL	15128	77.81893	18165	86.417697
NR	15468	79.567901	18293	87.026641
Unanotated	3529	18.15	2326	11.06
**Annotated**	**15914**	**81.85**	**18698**	**88.94**

Table S14. The 40 mitogenomes of 11 Elateroidea families and *Tribolium castaneum*.

Family	Subfamily	Species	Length (bp)	GenBank accession
**Lampyridae**	**Luciolinae**	***Abscondita terminalis***	**16402**	**MK292092**
Lampyridae	Luciolinae	*Aquatica lateralis*	16719	NC_035755.1
Lampyridae	Luciolinae	*Luciola substriata*	16248	NC_027176.1
Lampyridae	Luciolinae	*Pteroptyx maipo*	16127	NC_036353.1
Lampyridae	Luciolinae	*Asymmetricata circumdata*	15967	NC_032062.1
**Lampyridae**	**incertae sedis**	***Lamprigera yunnana***	**16203**	**MK292091**
Lampyridae	Lampyrinae	*Photinus pyralis*	17081	KY778696.1
Lampyridae	Lampyrinae	*Lampyris noctiluca*	17221	KX087302.1
Lampyridae	Lampyrinae	*Pyrocoelia rufa*	17739	NC_003970.1
Lampyridae	Photurinae	*Bicellonychia lividipennis*	16466	NC_030060.1
Phengodidae	Mastinocerinae	*Brasilocerus* sp. 2 DTA-2012	14458	KJ938490.1
Phengodidae	Phengodinae	*Phrixothrix hirtus*	20919	KM923891.1
Rhagophthalmidae	Rhagophthalminae	*Rhagophthalmus ohbai*	15704	NC_010964.1
Rhagophthalmidae	Rhagophthalminae	*Rhagophthalmus lufengensis*	15982	NC_010969.1
Elateridae	Agrypninae	*Hapsodrilus ignifer*	16072	NC_030058.1
Elateridae	Agrypninae	*Pyrearinus termitilluminans*	16007	NC_030059.1
Elateridae	Agrypninae	*Ignelater luminosus*	16079	MG242621.1
Elateridae	Agrypninae	*Pyrophorus divergens*	16120	NC_009964.1
Elateridae	Elaterinae	*Agriotes obscurus ?*	16264	KT876879.1
Elateridae	Elaterinae	*Adrastus rachifer*	16168	KX087232.1
Elateridae	Melanotinae	*Melanotus villosus*	15907	KT876904.1
Elateridae	Prosterninae	*Anostirus castaneus*	16376	KX087237.1
Elateridae	Denticollinae	*Limonius californicus*	16467	NC_028541.1
Elateridae	Denticollinae	*Athous haemorrhoidalis*	16529	KT876881.1
Elateridae	Sinophorinae	*Sinopyrophorus schimmeli*	15951	MH065615.1
Elateridae	Physodactylinae	*Teslasena femoralis*	15331	KJ938491.1
Elateridae	Cardiophorinae	*Dicronychus cinereus*	16703	KX087283.1
Elateridae	Drilidae	*Drilus flavescens*	12519	HQ232815.1
Cantharidae	Cantharinae	*Cantharis pellucida*	12400	HQ232817.1
Cantharidae	Chauliognathinae	*Chauliognathus opacus*	14893	NC_013576.1
Lycidae	Erotinae	*Dictyoptera aurora*	14298	JX412733.1
Lycidae	Leptolycinae	*Platerodrilus* sp	16394	KU878647.1
Lycidae	Lycinae	*Lycus dentipes*	12257	HQ232814.1
Throscidae	None	*Trixagus* sp. TRI01	10533	JX412793.1
Cerophytidae	None	*Cerophytidae* sp BMNH 900085	15741	KX035161.1
Eucnemidae	None	*Melasis buprestoides*	15729	KX087315.1
Eucnemidae	None	*Eucnemidae* sp. 4 ACP-2013	16170	MH923241.1
Artematopodidae	None	*Eurypogon* sp. EUR01	10562	JX412809.1
Omethidae	None	*Drilonius striatulus*	10751	JX412822.1
Tenebrionidae	Tenebrionidae insect sedis	*Tribolium castaneum*	15,881	NC_003081.2

Table S15. Summary of gene families in 21 species.

Order	Family	Species	Total genes	Genes in families	Unclustered genes	Families	Unique families	Genes per family	Maximum gene family size
Coleoptera	Lampyridae	*Lamprigera yunnana* (Lyu)	19,443	16,663	2,780	10,233	438	1.63	841
	Lampyridae	*Abscondita terminalis* (Ate)	21,024	15,556	5,468	11,189	658	1.39	175
	Lampyridae	*Aquatica lateralis* (Ala)	13,422	11,990	1,432	10,282	134	1.16	117
	Lampyridae	*Photinus pyralis* (Ppy)	15,504	14,306	1,198	10,600	281	1.34	63
	Lampyridae	*Pyrocoelia pectoralis* (Ppe)	23,054	21,010	2,044	11,663	720	1.80	81
	Elateridae	*Ignelater luminosus* (Ilu)	26,224	21,001	5,223	11,959	1,385	1.75	131
	Cerambycidae	*Anoplophora glabripennis* (Agl)	22,228	16,637	5591	11,850	665	1.40	82
	Buprestidae	*Agrilus planipennis* (Apl)	11,180	10,159	1,021	8,851	113	1.14	22
	Curculionidae	*Dendroctonus ponderosae* (Dpo)	13,050	11,320	1,730	8,983	260	1.26	20
	Scarabaeidae	*Onthophagus taurus* (Ota)	17,472	13,592	3,880	10,502	488	1.29	29
	Tenebrionidae	*Tribolium castaneum* (Tca)	16,523	12,892	3,631	10,186	343	1.26	63
Lepidoptera	Bombycidae	*Bombyx mori* (Bmo)	14,612	12,117	2,495	10,280	158	1.17	96
	Papilionidae	*Papilio machaon* (Pma)	15,481	13,292	2,189	11,712	53	1.13	25
	Papilionidae	*Papilio xuthus* (Pxu)	15,316	13,232	2,084	11,603	54	1.14	23
Diptera	Drosophilidae	*Drosophila melanogaster* (Dme)	13,556	10,511	3,045	8,058	507	1.30	44
Hymenoptera	Apidae	*Apis mellifera* (Ame)	13,089	9,271	3,818	8,411	72	1.10	34
	Pteromalidae	*Nasonia vitripennis* (Nvi)	8,024	6,930	1,094	5,786	199	1.19	20
Hemiptera	Aphididae	*Acyrthosiphon pisum* (Api)	32,073	25,235	6,838	9,887	1,849	2.55	434
Phthiraptera	Pediculidae	*Pediculus humanus* (Phu)	10,750	8,890	1,860	8,215	51	1.08	15
Isoptera	Termopsidae	*Zootermopsis nevadensis* (Zne)	14,610	10,701	3,909	8,800	240	1.21	165
Outgroup	Daphniidae	*Daphnia pulex* (Dpu)	29,684	22,271	7,413	9,234	2,085	2.41	354
**All**	**-**	**-**	**366,319**	**297,576**	**68,743**	**208,284**	**10,753**	**1.42**	**-**

Table S16. The GO enrichment of Lampyridae-Elateridae specific gene families (P<0.05).

GO	GO_Type	GO_term	P value
GO:0006508	biological_process	proteolysis	2.50E-08
GO:0019538	biological_process	protein metabolic process	8.75E-04
GO:0008152	biological_process	metabolic process	3.12E-03
GO:0043170	biological_process	macromolecule metabolic process	3.47E-02
GO:0004222	molecular_function	metalloendopeptidase activity	1.02E-07
GO:0070011	molecular_function	peptidase activity, acting on L-amino acid peptides	2.60E-07
GO:0008233	molecular_function	peptidase activity	3.13E-07
GO:0008237	molecular_function	metallopeptidase activity	6.00E-07
GO:0004175	molecular_function	endopeptidase activity	7.32E-07
GO:0052689	molecular_function	carboxylic ester hydrolase activity	3.06E-04
GO:0016787	molecular_function	hydrolase activity	5.20E-04
GO:0005549	molecular_function	odorant binding	2.36E-03
GO:0008236	molecular_function	serine-type peptidase activity	7.05E-03
GO:0017171	molecular_function	serine hydrolase activity	7.05E-03
GO:0003824	molecular_function	catalytic activity	1.42E-02
GO:0020037	molecular_function	heme binding	1.91E-02
GO:0046906	molecular_function	tetrapyrrole binding	1.98E-02
GO:0005506	molecular_function	iron ion binding	2.06E-02
GO:0016705	molecular_function	oxidoreductase activity, acting on paired donors, with incorporation or reduction of molecular oxygen	2.26E-02
GO:0046914	molecular_function	transition metal ion binding	2.53E-02
GO:0016624	molecular_function	oxidoreductase activity, acting on the aldehyde or oxo group of donors, disulfide as acceptor	4.49E-03

Table S17. The KEGG enrichment of Lampyridae-Elateridae specific gene families (P<0.05).

KEGG_A_class	KEGG_B_class	Pathway ID	Pathway	P value
Organismal Systems	Digestive system	ko04977	Vitamin digestion and absorption	1.56E-06
Metabolism	Lipid metabolism	ko00561	Glycerolipid metabolism	4.19E-06
Organismal Systems	Digestive system	ko04975	Fat digestion and absorption	8.42E-06
Organismal Systems	Immune system	ko04612	Antigen processing and presentation	1.19E-04
Metabolism	Biosynthesis of other secondary metabolites	ko00940	Phenylpropanoid biosynthesis	4.31E-04
Organismal Systems	Immune system	ko04620	Toll-like receptor signaling pathway	8.67E-04
Metabolism	Amino acid metabolism	ko00360	Phenylalanine metabolism	1.81E-03
Metabolism	Metabolism of cofactors and vitamins	ko00130	Ubiquinone and other terpenoid-quinone biosynthesis	2.20E-03
Organismal Systems	Digestive system	ko04972	Pancreatic secretion	4.90E-03
Environmental Information Processing	Signal transduction	ko02020	Two-component system	5.08E-03
Organismal Systems	Endocrine system	ko04915	Estrogen signaling pathway	1.39E-02
Organismal Systems	Immune system	ko04640	Hematopoietic cell lineage	2.23E-02
Organismal Systems	Digestive system	ko04978	Mineral absorption	3.69E-02

Table S18. The GO enrichment of Lampyridae-specific gene families (P<0.05).

GO ID	GO_Type	GO_term	P value
GO:0006595	biological_process	polyamine metabolic process	6.53E-05
GO:0006596	biological_process	polyamine biosynthetic process	6.53E-05
GO:0009309	biological_process	amine biosynthetic process	6.53E-05
GO:0042401	biological_process	cellular biogenic amine biosynthetic process	6.53E-05
GO:0002084	biological_process	protein depalmitoylation	4.69E-04
GO:0042159	biological_process	lipoprotein catabolic process	4.69E-04
GO:0098734	biological_process	macromolecule depalmitoylation	4.69E-04
GO:0006576	biological_process	cellular biogenic amine metabolic process	1.89E-03
GO:0009308	biological_process	amine metabolic process	1.89E-03
GO:0044106	biological_process	cellular amine metabolic process	1.89E-03
GO:0006508	biological_process	proteolysis	3.62E-03
GO:0035601	biological_process	protein deacylation	4.20E-03
GO:0098732	biological_process	macromolecule deacylation	4.20E-03
GO:0042157	biological_process	lipoprotein metabolic process	1.90E-02
GO:0001578	biological_process	microtubule bundle formation	2.51E-02
GO:0035082	biological_process	axoneme assembly	2.51E-02
GO:0036158	biological_process	outer dynein arm assembly	2.51E-02
GO:0070286	biological_process	axonemal dynein complex assembly	2.51E-02
GO:0046836	biological_process	glycolipid transport	3.75E-02
GO:1901264	biological_process	carbohydrate derivative transport	4.97E-02
GO:0005615	cellular_component	extracellular space	3.07E-03
GO:0044421	cellular_component	extracellular region part	1.28E-02
GO:0036157	cellular_component	outer dynein arm	2.30E-02
GO:0016021	cellular_component	integral component of membrane	2.93E-02
GO:0031224	cellular_component	intrinsic component of membrane	2.93E-02
GO:0044425	cellular_component	membrane part	3.86E-02
GO:0005858	cellular_component	axonemal dynein complex	4.56E-02
GO:0005930	cellular_component	axoneme	4.56E-02
GO:0044447	cellular_component	axoneme part	4.56E-02
GO:0097014	cellular_component	ciliary plasm	4.56E-02
GO:0005549	molecular_function	odorant binding	2.27E-13
GO:0008236	molecular_function	serine-type peptidase activity	4.20E-05
GO:0017171	molecular_function	serine hydrolase activity	4.20E-05

GO:0004252	molecular_function	serine-type endopeptidase activity	2.92E-04
GO:0008474	molecular_function	palmitoyl-(protein) hydrolase activity	1.04E-03
GO:0098599	molecular_function	palmitoyl hydrolase activity	1.04E-03
GO:0042302	molecular_function	structural constituent of cuticle	1.11E-03
GO:0016790	molecular_function	thiolester hydrolase activity	3.55E-03
GO:0070011	molecular_function	peptidase activity, acting on L-amino acid peptides	4.13E-03
GO:0004175	molecular_function	endopeptidase activity	4.51E-03
GO:0008233	molecular_function	peptidase activity	4.67E-03
GO:0003993	molecular_function	acid phosphatase activity	4.83E-03
GO:0016787	molecular_function	hydrolase activity	1.75E-02
GO:0017089	molecular_function	glycolipid transporter activity	3.95E-02
GO:0004857	molecular_function	enzyme inhibitor activity	4.34E-02

Table S19. The KEGG enrichment of Lampyridae-specific gene families (P<0.05).

KEGG_A_class	KEGG_B_class	Pathway ID	Pathway	P value
Cellular Processes	Transport and catabolism	ko04142	Lysosome	3.79E-02
Metabolism	Amino acid metabolism	ko00300	Lysine biosynthesis	9.84E-05
Metabolism	Amino acid metabolism	ko00310	Lysine degradation	4.33E-03
Metabolism	Carbohydrate metabolism	ko00051	Fructose and mannose metabolism	1.49E-02
Metabolism	Biosynthesis of other secondary metabolites	ko00940	Phenylpropanoid biosynthesis	2.38E-02
Metabolism	Global and Overview	ko01230	Biosynthesis of amino acids	2.42E-02
Metabolism	Lipid metabolism	ko00561	Glycerolipid metabolism	2.99E-02
Metabolism	Amino acid metabolism	ko00330	Arginine and proline metabolism	3.97E-02
Metabolism	Carbohydrate metabolism	ko00562	Inositol phosphate metabolism	4.13E-02
Organismal Systems	Digestive system	ko04977	Vitamin digestion and absorption	4.22E-04
Organismal Systems	Digestive system	ko04975	Fat digestion and absorption	1.03E-02
Organismal Systems	Digestive system	ko04978	Mineral absorption	1.49E-02

Table S20. The GO enrichment of Lampyrinae-specific gene families (P<0.01).

GO ID	GO_Type	GO_term	P value
GO:0055114	biological_process	oxidation-reduction process	1.23E-12
GO:0006810	biological_process	transport	2.83E-11
GO:0051234	biological_process	establishment of localization	3.40E-11
GO:0051179	biological_process	localization	6.11E-11
GO:0055085	biological_process	transmembrane transport	1.01E-10
GO:0007218	biological_process	neuropeptide signaling pathway	4.07E-09
GO:0044765	biological_process	single-organism transport	4.85E-05
GO:1902578	biological_process	single-organism localization	6.01E-05
GO:0006508	biological_process	proteolysis	1.18E-04
GO:0007608	biological_process	sensory perception of smell	5.52E-04
GO:0016486	biological_process	peptide hormone processing	9.70E-04
GO:0042445	biological_process	hormone metabolic process	3.16E-03
GO:0044710	biological_process	single-organism metabolic process	5.03E-03
GO:0010817	biological_process	regulation of hormone levels	7.07E-03
GO:0016021	cellular_component	integral component of membrane	3.66E-05
GO:0031224	cellular_component	intrinsic component of membrane	3.66E-05
GO:0044425	cellular_component	membrane part	5.71E-04
GO:0016020	cellular_component	membrane	1.30E-03
GO:0031012	cellular_component	extracellular matrix	3.50E-03
GO:0000796	cellular_component	condensin complex	9.41E-03
GO:0005549	molecular_function	odorant binding	4.21E-47
GO:0020037	molecular_function	heme binding	4.77E-36
GO:0046906	molecular_function	tetrapyrrole binding	9.79E-36

GO:0005506	molecular_function	iron ion binding	1.27E-34
GO:0016705	molecular_function	oxidoreductase activity, acting on paired donors, with incorporation or reduction of molecular oxygen	6.10E-31
GO:0046914	molecular_function	transition metal ion binding	2.81E-16
GO:0005215	molecular_function	transporter activity	1.55E-13
GO:0004222	molecular_function	metalloendopeptidase activity	6.81E-13
GO:0046872	molecular_function	metal ion binding	2.54E-08
GO:0016491	molecular_function	oxidoreductase activity	4.50E-08
GO:0043169	molecular_function	cation binding	4.87E-08
GO:0008237	molecular_function	metallopeptidase activity	6.84E-08
GO:0004175	molecular_function	endopeptidase activity	1.71E-07
GO:0004497	molecular_function	monooxygenase activity	2.31E-06
GO:0008146	molecular_function	sulfotransferase activity	5.09E-06
GO:0004144	molecular_function	diacylglycerol O-acyltransferase activity	6.87E-06
GO:0016411	molecular_function	acylglycerol O-acyltransferase activity	6.87E-06
GO:0016782	molecular_function	transferase activity, transferring sulfur-containing groups	1.75E-05
GO:0008374	molecular_function	O-acyltransferase activity	3.43E-05
GO:0016747	molecular_function	transferase activity, transferring acyl groups other than amino-acyl groups	8.08E-05
GO:0016746	molecular_function	transferase activity, transferring acyl groups	9.95E-05
GO:0070011	molecular_function	peptidase activity, acting on L-amino acid peptides	1.00E-03
GO:0004984	molecular_function	olfactory receptor activity	1.25E-03
GO:0008233	molecular_function	peptidase activity	1.61E-03
GO:0003993	molecular_function	acid phosphatase activity	7.17E-03
GO:0022857	molecular_function	transmembrane transporter activity	8.11E-03

Table S21. The KEGG enrichment of Lampyrinae-specific gene families (P<0.05).

KEGG_A_class	KEGG_B_class	Pathway	P value
Metabolism	Metabolism of cofactors and vitamins	Ubiquinone and other terpenoid-quinone biosynthesis	1.55E-06
Metabolism	Biosynthesis of other secondary metabolites	Phenylpropanoid biosynthesis	3.00E-04
Metabolism	Lipid metabolism	Steroid biosynthesis	1.59E-03
Metabolism	Xenobiotics biodegradation and metabolism	Drug metabolism - other enzymes	2.44E-03
Metabolism	Metabolism of cofactors and vitamins	Retinol metabolism	4.48E-03
Metabolism	Biosynthesis of other secondary metabolites	Flavonoid biosynthesis	2.37E-02
Metabolism	Biosynthesis of other secondary metabolites	Glucosinolate biosynthesis	2.37E-02
Metabolism	Glycan biosynthesis and metabolism	Glycosaminoglycan biosynthesis - keratan sulfate	4.69E-02
Organismal Systems	Digestive system	Fat digestion and absorption	4.62E-02

Table S22. The GO enrichment of Luciolinae-specific gene families (P<0.01).

GO ID	GO_Type	GO_term	P value
GO:0006743	biological_process	ubiquinone metabolic process	2.87E-06
GO:0006744	biological_process	ubiquinone biosynthetic process	2.87E-06
GO:0042181	biological_process	ketone biosynthetic process	6.05E-06
GO:1901661	biological_process	quinone metabolic process	6.05E-06
GO:1901663	biological_process	quinone biosynthetic process	6.05E-06
GO:0006147	biological_process	guanine catabolic process	6.49E-06
GO:0046098	biological_process	guanine metabolic process	6.49E-06
GO:0006145	biological_process	purine nucleobase catabolic process	1.61E-05
GO:0072523	biological_process	purine-containing compound catabolic process	1.61E-05
GO:0042180	biological_process	cellular ketone metabolic process	1.69E-05
GO:0046113	biological_process	nucleobase catabolic process	3.19E-05
GO:0006144	biological_process	purine nucleobase metabolic process	4.29E-04
GO:0006733	biological_process	oxidoreduction coenzyme metabolic process	1.08E-03
GO:0009108	biological_process	coenzyme biosynthetic process	3.32E-03
GO:0007018	biological_process	microtubule-based movement	3.78E-03
GO:0009112	biological_process	nucleobase metabolic process	4.32E-03
GO:0006928	biological_process	movement of cell or subcellular component	5.52E-03
GO:0051188	biological_process	cofactor biosynthetic process	6.91E-03
GO:0006508	biological_process	proteolysis	7.97E-03
GO:0016021	cellular_component	integral component of membrane	8.12E-03
GO:0031224	cellular_component	intrinsic component of membrane	8.12E-03
GO:0046872	molecular_function	metal ion binding	3.41E-74
GO:0043169	molecular_function	cation binding	3.88E-73

GO:0003676	molecular_function	nucleic acid binding	3.15E-53
GO:0043167	molecular_function	ion binding	3.01E-32
GO:1901363	molecular_function	heterocyclic compound binding	1.96E-23
GO:0097159	molecular_function	organic cyclic compound binding	2.12E-23
GO:0005488	molecular_function	binding	4.06E-06
GO:0008425	molecular_function	2-polyprenyl-6-methoxy-1,4-benzoquinone methyltransferase activity	5.22E-05
GO:0030580	molecular_function	quinone cofactor methyltransferase activity	5.22E-05
GO:0008169	molecular_function	C-methyltransferase activity	7.62E-05
GO:0008892	molecular_function	guanine deaminase activity	1.28E-04
GO:0005549	molecular_function	odorant binding	2.75E-04
GO:0004144	molecular_function	diacylglycerol O-acyltransferase activity	7.39E-03
GO:0016411	molecular_function	acylglycerol O-acyltransferase activity	9.18E-03

Table S23. The KEGG enrichment of Luciolinae-specific gene families (P<0.05).

KEGG_A_class	KEGG_B_class	Pathway	Pvalue
Cellular Processes	Transport and catabolism	Peroxisome	2.69E-02
Environmental Information Processing	Signaling molecules and interaction	ECM-receptor interaction	2.43E-02
Environmental Information Processing	Signaling molecules and interaction	Cell adhesion molecules (CAMs)	1.16E-02
Metabolism	Metabolism of cofactors and vitamins	Ubiquinone and other terpenoid-quinone biosynthesis	4.75E-10
Metabolism	Glycan biosynthesis and metabolism	Glycosaminoglycan biosynthesis - keratan sulfate	2.32E-06
Metabolism	Biosynthesis of other secondary metabolites	Phenylpropanoid biosynthesis	1.01E-05
Metabolism	Glycan biosynthesis and metabolism	Glycosphingolipid biosynthesis - globo series	6.75E-05
Metabolism	Amino acid metabolism	Phenylalanine metabolism	1.41E-04
Metabolism	Metabolism of terpenoids and polyketides	Insect hormone biosynthesis	2.35E-04
Metabolism	Glycan biosynthesis and metabolism	N-Glycan biosynthesis	6.51E-04
Metabolism	Xenobiotics biodegradation and metabolism	Caprolactam degradation	1.02E-03
Metabolism	Xenobiotics biodegradation and metabolism	Metabolism of xenobiotics by cytochrome P450	2.19E-03
Metabolism	Carbohydrate metabolism	Ascorbate and aldarate metabolism	3.32E-03
Metabolism	Global and Overview	Degradation of aromatic compounds	5.32E-03
Metabolism	Global and Overview	2-Oxocarboxylic acid metabolism	1.34E-02
Metabolism	Xenobiotics biodegradation and metabolism	Drug metabolism - cytochrome P450	1.42E-02
Metabolism	Metabolism of other amino acids	Glutathione metabolism	2.91E-02
Metabolism	Carbohydrate metabolism	Pentose phosphate pathway	3.48E-02
Organismal Systems	Immune system	Platelet activation	1.53E-02
Organismal Systems	Immune system	Leukocyte transendothelial migration	1.84E-02

Table S24. The GO enrichment of *L. yunnana* specific genes (FDR<0.01).

GO ID	GO_Type	GO_term	FDR
GO:0003676	molecular_function	nucleic acid binding	0.00E+00
GO:0043169	molecular_function	cation binding	0.00E+00
GO:0046872	molecular_function	metal ion binding	0.00E+00
GO:1901363	molecular_function	heterocyclic compound binding	3.81E-235
GO:0097159	molecular_function	organic cyclic compound binding	4.53E-235
GO:0043167	molecular_function	ion binding	2.33E-193
GO:0005488	molecular_function	binding	2.32E-53
GO:0005549	molecular_function	odorant binding	3.71E-09
GO:0008169	molecular_function	C-methyltransferase activity	4.38E-06
GO:0008425	molecular_function	2-polyprenyl-6-methoxy-1,4-benzoquinone methyltransferase activity	4.38E-06
GO:0030580	molecular_function	quinone cofactor methyltransferase activity	4.38E-06
GO:0008146	molecular_function	sulfotransferase activity	5.18E-05
GO:0016782	molecular_function	transferase activity, transferring sulfur-containing groups	2.73E-04
GO:0006743	biological_process	ubiquinone metabolic process	3.12E-08
GO:0006744	biological_process	ubiquinone biosynthetic process	3.12E-08
GO:0042181	biological_process	ketone biosynthetic process	3.12E-08
GO:1901661	biological_process	quinone metabolic process	3.12E-08
GO:1901663	biological_process	quinone biosynthetic process	3.12E-08
GO:0008152	biological_process	metabolic process	1.18E-06
GO:0044710	biological_process	single-organism metabolic process	1.18E-06
GO:0042180	biological_process	cellular ketone metabolic process	1.72E-06
GO:0051188	biological_process	cofactor biosynthetic process	6.57E-06
GO:0006733	biological_process	oxidoreduction coenzyme metabolic process	7.24E-06
GO:0009108	biological_process	coenzyme biosynthetic process	7.24E-06
GO:0044283	biological_process	small molecule biosynthetic process	7.95E-06
GO:0051186	biological_process	cofactor metabolic process	1.61E-05
GO:0006732	biological_process	coenzyme metabolic process	2.50E-05
GO:0055114	biological_process	oxidation-reduction process	5.11E-05
GO:0044249	biological_process	cellular biosynthetic process	5.97E-04
GO:1901576	biological_process	organic substance biosynthetic process	1.20E-03
GO:0000160	biological_process	phosphorelay signal transduction system	2.50E-03
GO:0044281	biological_process	small molecule metabolic process	3.11E-03
GO:0009058	biological_process	biosynthetic process	3.44E-03
GO:0071103	biological_process	DNA conformation change	4.25E-03
GO:0044711	biological_process	single-organism biosynthetic process	8.39E-03

Table S25. The KEGG enrichment of expanded gene families in the ancestor of Elateridae-Lampyridae beetles (FDR<0.05).

KEGG_A_class	KEGG_B_class	Pathway ID	Pathway	FDR
Cellular Processes	Cellular commiunity	ko04540	Gap junction	1.19E-05
Cellular Processes	Transport and catabolism	ko04145	Phagosome	6.66E-05
Environmental Information Processing	Membrane transport	ko02010	ABC transporters	1.07E-25
Environmental Information Processing	Signal transduction	ko04024	cAMP signaling pathway	1.05E-06
Environmental Information Processing	Signal transduction	ko04152	AMPK signaling pathway	1.41E-03
Metabolism	Xenobiotics biodegradation and metabolism	ko00983	Drug metabolism - other enzymes	1.97E-32
Metabolism	Metabolism of cofactors and vitamins	ko00830	Retinol metabolism	3.68E-19
Metabolism	Metabolism of cofactors and vitamins	ko00860	Porphyrin and chlorophyll metabolism	1.65E-17
Metabolism	Carbohydrate metabolism	ko00053	Ascorbate and aldarate metabolism	8.50E-17
Metabolism	Metabolism of terpenoids and polyketides	ko00981	Insect hormone biosynthesis	2.56E-16
Metabolism	Lipid metabolism	ko00140	Steroid hormone biosynthesis	9.95E-16
Metabolism	Biosynthesis of other secondary metabolites	ko00940	Phenylpropanoid biosynthesis	1.10E-15
Metabolism	Carbohydrate metabolism	ko00040	Pentose and glucuronate interconversions	8.21E-13
Metabolism	Carbohydrate metabolism	ko00500	Starch and sucrose metabolism	8.21E-13
Metabolism	Amino acid metabolism	ko00360	Phenylalanine metabolism	9.42E-13
Metabolism	Amino acid metabolism	ko00260	Glycine, serine and threonine metabolism	6.26E-11
Metabolism	Xenobiotics biodegradation and metabolism	ko00980	Metabolism of xenobiotics by cytochrome P450	6.89E-11
Metabolism	Lipid metabolism	ko00061	Fatty acid biosynthesis	1.08E-10

Metabolism	Xenobiotics biodegradation and metabolism	ko00982	Drug metabolism - cytochrome P450	1.08E-10
Metabolism	Metabolism of cofactors and vitamins	ko00130	Ubiquinone and other terpenoid-quinone biosynthesis	1.66E-10
Metabolism	Global and Overview	ko01212	Fatty acid metabolism	4.33E-08
Metabolism	Carbohydrate metabolism	ko00030	Pentose phosphate pathway	6.84E-04
Metabolism	Energy metabolism	ko00920	Sulfur metabolism	1.42E-03
Metabolism	Biosynthesis of other secondary metabolites	ko00261	Monobactam biosynthesis	2.44E-03
Metabolism	Glycan biosynthesis and metabolism	ko00511	Other glycan degradation	4.53E-03
Metabolism	Lipid metabolism	ko00592	alpha-Linolenic acid metabolism	1.03E-02
Metabolism	Amino acid metabolism	ko00350	Tyrosine metabolism	1.08E-02
Metabolism	Biosynthesis of other secondary metabolites	ko00965	Betalain biosynthesis	2.08E-02
Metabolism	Metabolism of cofactors and vitamins	ko00740	Riboflavin metabolism	2.08E-02
Metabolism	Lipid metabolism	ko00071	Fatty acid degradation	2.35E-02
Metabolism	Carbohydrate metabolism	ko00052	Galactose metabolism	2.63E-02
Metabolism	Biosynthesis of other secondary metabolites	ko00232	Caffeine metabolism	2.91E-02
Metabolism	Metabolism of other amino acids	ko00460	Cyanoamino acid metabolism	2.91E-02
Organismal Systems	Digestive system	ko04976	Bile secretion	3.61E-31
Organismal Systems	Immune system	ko04612	Antigen processing and presentation	1.41E-03
Organismal Systems	Digestive system	ko04973	Carbohydrate digestion and absorption	8.64E-03
Organismal Systems	Endocrine system	ko03320	PPAR signaling pathway	2.94E-02
Organismal Systems	Endocrine system	ko04910	Insulin signaling pathway	3.55E-02

Table S26. The KEGG enrichment of expanded gene families in Lampyridae (FDR<0.01).

KEGG_A_class	KEGG_B_class	Pathway ID	Pathway	FDR
Cellular Processes	Cellular commiunity	ko04540	Gap junction	4.29E-07
Environmental Information Processing	Membrane transport	ko02010	ABC transporters	8.49E-30
Environmental Information Processing	Signal transduction	ko04024	cAMP signaling pathway	1.68E-08
Environmental Information Processing	Signal transduction	ko04152	AMPK signaling pathway	3.87E-03
Metabolism	Xenobiotics biodegradation and metabolism	ko00983	Drug metabolism - other enzymes	8.26E-25
Metabolism	Carbohydrate metabolism	ko00053	Ascorbate and aldarate metabolism	1.16E-14
Metabolism	Amino acid metabolism	ko00260	Glycine, serine and threonine metabolism	2.88E-14
Metabolism	Metabolism of cofactors and vitamins	ko00830	Retinol metabolism	7.83E-12
Metabolism	Metabolism of cofactors and vitamins	ko00860	Porphyrin and chlorophyll metabolism	1.68E-11
Metabolism	Xenobiotics biodegradation and metabolism	ko00980	Metabolism of xenobiotics by cytochrome P450	8.10E-11
Metabolism	Lipid metabolism	ko00061	Fatty acid biosynthesis	1.52E-10
Metabolism	Global and Overview	ko01212	Fatty acid metabolism	2.77E-10
Metabolism	Lipid metabolism	ko00140	Steroid hormone biosynthesis	2.77E-10
Metabolism	Carbohydrate metabolism	ko00500	Starch and sucrose metabolism	1.07E-09
Metabolism	Xenobiotics biodegradation and metabolism	ko00982	Drug metabolism - cytochrome P450	2.69E-09
Metabolism	Carbohydrate metabolism	ko00040	Pentose and glucuronate interconversions	2.98E-08
Metabolism	Biosynthesis of other secondary metabolites	ko00940	Phenylpropanoid biosynthesis	8.78E-05
Metabolism	Metabolism of terpenoids and polyketides	ko00981	Insect hormone biosynthesis	2.50E-03
Metabolism	Carbohydrate metabolism	ko00030	Pentose phosphate pathway	3.79E-03
Metabolism	Amino acid metabolism	ko00360	Phenylalanine metabolism	5.31E-03

Metabolism	Metabolism of other amino acids	ko00480	Glutathione metabolism	7.59E-03
Metabolism	Metabolism of cofactors and vitamins	ko00130	Ubiquinone and other terpenoid-quinone biosynthesis	8.55E-03
Metabolism	Lipid metabolism	ko00071	Fatty acid degradation	8.93E-03
Organismal Systems	Digestive system	ko04976	Bile secretion	1.93E-32
Organismal Systems	Immune system	ko04640	Hematopoietic cell lineage	1.68E-07
Organismal Systems	Endocrine system	ko04614	Renin-angiotensin system	1.57E-04
Organismal Systems	Endocrine system	ko04910	Insulin signaling pathway	3.87E-03

Table S27. The KEGG enrichment of expanded gene families in the ancestor of Lampyrinae (*P. pyralis* and *P. pectoralis*) (FDR<0.01).

KEGG_A_class	KEGG_B_class	Pathway ID	Pathway	FDR
Cellular Processes	Transport and catabolism	ko04145	Phagosome	1.66E-06
Cellular Processes	Cellular commiunity	ko04540	Gap junction	1.74E-06
Cellular Processes	Cell motility	ko02030	Bacterial chemotaxis	5.84E-05
Environmental Information Processing	Membrane transport	ko02010	ABC transporters	2.88E-03
Metabolism	Lipid metabolism	ko00140	Steroid hormone biosynthesis	4.67E-20
Metabolism	Metabolism of cofactors and vitamins	ko00830	Retinol metabolism	1.79E-17
Metabolism	Biosynthesis of other secondary metabolites	ko00940	Phenylpropanoid biosynthesis	1.82E-14
Metabolism	Amino acid metabolism	ko00360	Phenylalanine metabolism	1.27E-10
Metabolism	Carbohydrate metabolism	ko00053	Ascorbate and aldarate metabolism	4.99E-10
Metabolism	Xenobiotics biodegradation and metabolism	ko00627	Aminobenzoate degradation	8.83E-10
Metabolism	Lipid metabolism	ko00591	Linoleic acid metabolism	9.36E-10
Metabolism	Metabolism of terpenoids and polyketides	ko00903	Limonene and pinene degradation	1.35E-09
Metabolism	Amino acid metabolism	ko00340	Histidine metabolism	4.10E-09
Metabolism	Metabolism of other amino acids	ko00460	Cyanoamino acid metabolism	7.44E-09
Metabolism	Carbohydrate metabolism	ko00040	Pentose and glucuronate interconversions	1.74E-08
Metabolism	Metabolism of other amino acids	ko00410	beta-Alanine metabolism	8.07E-08
Metabolism	Biosynthesis of other secondary metabolites	ko00945	Stilbenoid, diarylheptanoid and gingerol biosynthesis	2.96E-07
Metabolism	Xenobiotics biodegradation and metabolism	ko00624	Polycyclic aromatic hydrocarbon degradation	1.72E-06
Metabolism	Xenobiotics biodegradation and metabolism	ko00363	Bisphenol degradation	1.72E-06
Metabolism	Xenobiotics biodegradation and metabolism	ko00982	Drug metabolism - cytochrome P450	3.29E-06
Metabolism	Metabolism of cofactors and vitamins	ko00860	Porphyrin and chlorophyll metabolism	1.04E-05

Metabolism	Carbohydrate metabolism	ko00500	Starch and sucrose metabolism	1.12E-05
Metabolism	Amino acid metabolism	ko00260	Glycine, serine and threonine metabolism	1.44E-05
Metabolism	Xenobiotics biodegradation and metabolism	ko00983	Drug metabolism - other enzymes	2.93E-05
Metabolism	Lipid metabolism	ko00071	Fatty acid degradation	5.12E-05
Metabolism	Metabolism of cofactors and vitamins	ko00130	Ubiquinone and other terpenoid-quinone biosynthesis	2.56E-04
Metabolism	Xenobiotics biodegradation and metabolism	ko00980	Metabolism of xenobiotics by cytochrome P450	6.00E-04
Metabolism	Metabolism of cofactors and vitamins	ko00790	Folate biosynthesis	1.09E-03
Metabolism	Biosynthesis of other secondary metabolites	ko00944	Flavone and flavonol biosynthesis	2.74E-03
Metabolism	Amino acid metabolism	ko00330	Arginine and proline metabolism	9.30E-03
Metabolism	Amino acid metabolism	ko00280	Valine, leucine and isoleucine degradation	9.36E-03
Organismal Systems	Digestive system	ko04976	Bile secretion	3.64E-15
Organismal Systems	Endocrine system	ko03320	PPAR signaling pathway	3.08E-04
Organismal Systems	Immune system	ko04610	Complement and coagulation cascades	6.07E-03

Table S28. The KEGG enrichment of expanded gene families in the ancestor of Luciolinae (*A. terminalis* and *A. lateralis*) (FDR<0.01).

KEGG_A_class	KEGG_B_class	Pathway ID	Pathway	FDR
Cellular Processes	Transport and catabolism	ko04146	Peroxisome	1.03E-08
Environmental Information Processing	Membrane transport	ko02010	ABC transporters	3.64E-42
Environmental Information Processing	Signal transduction	ko04024	cAMP signaling pathway	2.29E-41
Metabolism	Biosynthesis of other secondary metabolites	ko00232	Caffeine metabolism	4.82E-08
Metabolism	Xenobiotics biodegradation and metabolism	ko00983	Drug metabolism - other enzymes	6.84E-07
Metabolism	Lipid metabolism	ko00592	alpha-Linolenic acid metabolism	2.88E-06
Metabolism	Metabolism of cofactors and vitamins	ko00130	Ubiquinone and other terpenoid-quinone biosynthesis	3.84E-06
Metabolism	Biosynthesis of other secondary metabolites	ko00940	Phenylpropanoid biosynthesis	9.03E-06
Metabolism	Lipid metabolism	ko01040	Biosynthesis of unsaturated fatty acids	1.20E-04
Metabolism	Amino acid metabolism	ko00360	Phenylalanine metabolism	5.25E-04
Metabolism	Glycan biosynthesis and metabolism	ko00533	Glycosaminoglycan biosynthesis - keratan sulfate	2.93E-03
Metabolism	Lipid metabolism	ko00071	Fatty acid degradation	3.31E-03
Organismal Systems	Digestive system	ko04976	Bile secretion	2.29E-41
Organismal Systems	Endocrine system	ko03320	PPAR signaling pathway	3.36E-04

Table S29. The KEGG enrichment of expanded gene families in *L. yunnana* (FDR<0.01).

KEGG_A_class	KEGG_B_class	Pathway ID	Pathway	FDR
Cellular Processes	Transport and catabolism	ko04145	Phagosome	3.06E-06
Environmental Information Processing	Signaling molecules and interaction	ko04514	Cell adhesion molecules (CAMs)	2.80E-08
Organismal Systems	Digestive system	ko04972	Pancreatic secretion	3.94E-06
Organismal Systems	Digestive system	ko04974	Protein digestion and absorption	1.90E-05
Organismal Systems	Digestive system	ko04976	Bile secretion	3.86E-05
Organismal Systems	Endocrine system	ko03320	PPAR signaling pathway	1.06E-03

Table S30. The KEGG enrichment of expanded gene families in *A. terminalis* (P<0.05).

KEGG_A_class	KEGG_B_class	Pathway ID	Pathway	P value
Cellular Processes	Transport and catabolism	ko04146	Peroxisome	1.60E-03
Cellular Processes	Cellular commiunity	ko04540	Gap junction	3.53E-02
Environmental Information Processing	Membrane transport	ko02010	ABC transporters	2.11E-13
Environmental Information Processing	Signal transduction	ko04024	cAMP signaling pathway	1.31E-12
Environmental Information Processing	Signal transduction	ko04152	AMPK signaling pathway	2.10E-04
Environmental Information Processing	Signal transduction	ko04020	Calcium signaling pathway	1.09E-02
Genetic Information Processing	Replication and repair	ko03420	Nucleotide excision repair	1.08E-03
Genetic Information Processing	Folding, sorting and degradation	ko03060	Protein export	2.51E-02
Genetic Information Processing	Transcription	ko03040	Spliceosome	3.02E-02
Genetic Information Processing	Transcription	ko03020	RNA polymerase	3.24E-02
Genetic Information Processing	Folding, sorting and degradation	ko04122	Sulfur relay system	4.42E-02
Metabolism	Xenobiotics biodegradation and metabolism	ko00983	Drug metabolism - other enzymes	5.42E-18
Metabolism	Carbohydrate metabolism	ko00053	Ascorbate and aldarate metabolism	2.29E-17
Metabolism	Metabolism of cofactors and vitamins	ko00830	Retinol metabolism	7.37E-17
Metabolism	Xenobiotics biodegradation and metabolism	ko00982	Drug metabolism - cytochrome P450	4.18E-16
Metabolism	Xenobiotics biodegradation and metabolism	ko00980	Metabolism of xenobiotics by cytochrome P450	9.85E-14
Metabolism	Carbohydrate metabolism	ko00040	Pentose and glucuronate interconversions	1.54E-13
Metabolism	Metabolism of cofactors and vitamins	ko00860	Porphyrin and chlorophyll metabolism	8.15E-13

Metabolism	Amino acid metabolism	ko00260	Glycine, serine and threonine metabolism	3.06E-11
Metabolism	Lipid metabolism	ko00140	Steroid hormone biosynthesis	4.11E-10
Metabolism	Carbohydrate metabolism	ko00500	Starch and sucrose metabolism	3.22E-09
Metabolism	Global and Overview	ko01210	2-Oxocarboxylic acid metabolism	1.32E-05
Metabolism	Lipid metabolism	ko00061	Fatty acid biosynthesis	1.69E-04
Metabolism	Global and Overview	ko01212	Fatty acid metabolism	1.97E-04
Metabolism	Metabolism of terpenoids and polyketides	ko00981	Insect hormone biosynthesis	3.27E-04
Metabolism	Biosynthesis of other secondary metabolites	ko00232	Caffeine metabolism	3.86E-04
Metabolism	Amino acid metabolism	ko00220	Arginine biosynthesis	1.02E-03
Metabolism	Biosynthesis of other secondary metabolites	ko00401	Novobiocin biosynthesis	1.82E-03
Metabolism	Energy metabolism	ko00190	Oxidative phosphorylation	4.59E-03
Metabolism	Carbohydrate metabolism	ko00030	Pentose phosphate pathway	6.45E-03
Metabolism	Energy metabolism	ko00195	Photosynthesis	7.60E-03
Metabolism	Lipid metabolism	ko01040	Biosynthesis of unsaturated fatty acids	7.95E-03
Metabolism	Global and Overview	ko01230	Biosynthesis of amino acids	8.42E-03
Metabolism	Biosynthesis of other secondary metabolites	ko00940	Phenylpropanoid biosynthesis	1.06E-02
Metabolism	Amino acid metabolism	ko00250	Alanine, aspartate and glutamate metabolism	1.79E-02
Metabolism	Metabolism of terpenoids and polyketides	ko01051	Biosynthesis of ansamycins	1.91E-02
Metabolism	Carbohydrate metabolism	ko00630	Glyoxylate and dicarboxylate metabolism	3.01E-02
Metabolism	Lipid metabolism	ko00592	alpha-Linolenic acid metabolism	3.72E-02
Metabolism	Metabolism of terpenoids and polyketides	ko00909	Sesquiterpenoid and triterpenoid biosynthesis	4.27E-02
Metabolism	Metabolism of terpenoids and polyketides	ko01053	Biosynthesis of siderophore group nonribosomal peptides	4.27E-02

Metabolism	Amino acid metabolism	ko00360	Phenylalanine metabolism	4.80E-02
Organismal Systems	Digestive system	ko04976	Bile secretion	3.05E-33
Organismal Systems	Immune system	ko04612	Antigen processing and presentation	3.18E-04
Organismal Systems	Endocrine system	ko03320	PPAR signaling pathway	1.71E-03
Organismal Systems	Endocrine system	ko04614	Renin-angiotensin system	2.65E-03
Organismal Systems	Endocrine system	ko04910	Insulin signaling pathway	9.20E-03
Organismal Systems	Endocrine system	ko04915	Estrogen signaling pathway	1.39E-02
Organismal Systems	Endocrine system	ko04913	Ovarian Steroidogenesis	2.94E-02

Table S31. The GO enrichment of positively selected genes (PSGs) in the Luciolinae and Lampyrinae.

	GO ID	GO_Type	GO_term	P value
	GO:0006996	biological_process	organelle organization	1.85E-03
	GO:0005044	molecular_function	scavenger receptor activity	8.39E-03
	GO:0038024	molecular_function	cargo receptor activity	8.39E-03
	GO:0070925	biological_process	organelle assembly	1.00E-02
	GO:1902589	biological_process	single-organism organelle organization	1.61E-02
	GO:0044710	biological_process	single-organism metabolic process	1.67E-02
Luciolinae	GO:0044699	biological_process	single-organism process	2.04E-02
	GO:0007017	biological_process	microtubule-based process	2.55E-02
	GO:0009163	biological_process	nucleoside biosynthetic process	2.55E-02
	GO:0042455	biological_process	ribonucleoside biosynthetic process	2.55E-02
	GO:1901659	biological_process	glycosyl compound biosynthetic process	2.55E-02
	GO:0006739	biological_process	NADP metabolic process	2.81E-02
	GO:0004812	molecular_function	aminoacyl-tRNA ligase activity	3.00E-02
	GO:0016875	molecular_function	ligase activity, forming carbon-oxygen bonds	3.00E-02
	GO:0016876	molecular_function	ligase activity, forming aminoacyl-tRNA and related compounds	3.00E-02
	GO:0019898	cellular_component	extrinsic component of membrane	3.09E-02
	GO:0006418	biological_process	tRNA aminoacylation for protein translation	3.78E-02
	GO:0043038	biological_process	amino acid activation	3.78E-02
	GO:0043039	biological_process	tRNA aminoacylation	3.78E-02
	GO:0051276	biological_process	chromosome organization	3.78E-02
	GO:0016043	biological_process	cellular component organization	3.81E-02
	GO:0034654	biological_process	nucleobase-containing compound biosynthetic process	3.87E-02
	GO:0004842	molecular_function	ubiquitin-protein transferase activity	4.21E-02

	GO:0019787	molecular_function	ubiquitin-like protein transferase activity	4.21E-02
	GO:0015103	molecular_function	inorganic anion transmembrane transporter activity	4.45E-02
	GO:0015108	molecular_function	chloride transmembrane transporter activity	4.45E-02
	GO:0071944	cellular_component	cell periphery	1.55E-03
	GO:0005886	cellular_component	plasma membrane	8.40E-03
Lampyrinae	GO:0072522	biological_process	purine-containing compound biosynthetic process	2.09E-04
	GO:0006164	biological_process	purine nucleotide biosynthetic process	5.98E-04
	GO:0019438	biological_process	aromatic compound biosynthetic process	1.05E-03
	GO:0072521	biological_process	purine-containing compound metabolic process	1.30E-03
	GO:0034654	biological_process	nucleobase-containing compound biosynthetic process	1.55E-03
	GO:0009127	biological_process	purine nucleoside monophosphate biosynthetic process	1.69E-03
	GO:0009168	biological_process	purine ribonucleoside monophosphate biosynthetic process	1.69E-03
	GO:0018130	biological_process	heterocycle biosynthetic process	1.71E-03
	GO:1901362	biological_process	organic cyclic compound biosynthetic process	1.71E-03
	GO:0044271	biological_process	cellular nitrogen compound biosynthetic process	2.06E-03
	GO:0006163	biological_process	purine nucleotide metabolic process	2.25E-03
	GO:0009152	biological_process	purine ribonucleotide biosynthetic process	3.62E-03
	GO:0044281	biological_process	small molecule metabolic process	5.67E-03
	GO:1901137	biological_process	carbohydrate derivative biosynthetic process	6.56E-03
	GO:0009126	biological_process	purine nucleoside monophosphate metabolic process	6.65E-03
	GO:0009167	biological_process	purine ribonucleoside monophosphate metabolic process	6.65E-03
	GO:0044249	biological_process	cellular biosynthetic process	8.32E-03
	GO:1901576	biological_process	organic substance biosynthetic process	9.36E-03

Table S32. The KEGG enrichment of positively selected genes (PSGs) in the Luciolinae and Lampyrinae.

	KEGG_A_class	KEGG_B_class	Pathway ID	Pathway	P value
Luciolinae	Genetic Information Processing	Translation	ko00970	Aminoacyl-tRNA biosynthesis	1.30E-03
	Genetic Information Processing	Translation	ko03013	RNA transport	4.40E-02
	Genetic Information Processing	Transcription	ko03022	Basal transcription factors	4.84E-02
Lampyrinae	Metabolism	Nucleotide metabolism	ko00230	Purine metabolism	5.28E-03
	Environmental Information Processing	Signal transduction	ko04340	Hedgehog signaling pathway	1.93E-02
	Environmental Information Processing	Signal transduction	ko04011	MAPK signaling pathway - yeast	3.11E-02
	Metabolism	Amino acid metabolism	ko00270	Cysteine and methionine metabolism	3.11E-02

Table S33. The statistic data of transcriptome reads in *L. yunnana* (Lyu) and *A. terminalis* (Ate).

Species	Stages	Tissue	Library	Raw length (bp)	Raw reads	Raw base (Gb)	Clean reads	Clean base (Gb)
**Lyu**	Male adult	whole body	Lg-Y10	150	21,766,280	6.53	20,830,224	6.25
	Female adult	whole body	Lg-Y23	150	22,642,193	6.79	21,723,864	6.52
	larva	whole body	Lg-YL7	150	22,271,236	6.68	21,270,667	6.38
	**Total**				**66,679,709**	**20.00**	**63,824,755**	**19.15**
	Female adult	luminous organs	Lg-YF17	150	20,047,628	6.01	20,014,360	5.60
		luminous organs	Lg-YF18	150	23,645,540	7.09	23,597,841	6.61
		luminous organs	Lg-YF19	150	26,336,259	7.90	26,288,608	7.36
	Male adult	luminous organs	Lg -YM16	150	23,476,758	7.04	23,432,096	6.56
		luminous organs	Lg -YM17	150	21,639,753	6.49	21,593,416	6.05
		luminous organs	Lg -YM18	150	21,065,975	6.32	20,991,144	5.88
	**Total**				**136,211,913**	**40.86**	**135,917,465**	**38.06**
**Ate**	Female adult	whole body	Lo-TM46	150	19,033,478	5.71	18,119,919	5.44
	Male adult	whole body	Lo-TM37	150	19,883,173	5.96	18,964,605	5.69
	Female pupa	whole body	Lo-TPF	150	23,940,563	7.18	22,871,413	6.86
	Male pupa	whole body	Lo-TPM	150	26,686,479	8.01	25,538,671	7.66
	larva	whole body	Lo-TL1	150	47,716,638	14.31	45,836,295	13.75
	**Total**				**137,260,331**	**41.18**	**131,330,903**	**39.4**
	Female adult	luminous organs	Lo-TF 45	150	19,792,559	5.94	19,765,179	5.53
		luminous organs	Lo-TF 46	150	18,714,895	5.61	18,673,237	5.23
		luminous organs	Lo-TF 47	150	19,685,688	5.91	19,650,356	5.50
	Male adult	luminous organs	Lo-TM90	150	18,029,075	5.41	17,998,079	5.04
		luminous organs	Lo-TM91	150	18,796,245	5.64	18,767,941	5.26
		luminous organs	Lo-TM92	150	19,444,696	5.83	19,415,191	5.44
	**Total**				**114,463,158**	**34.34**	**114,269,983**	**32.00**

Table S34. The results of *de novo* transcriptome assembly in *L. yunnana* (Lyu) and *A. terminalis* (Ate).

	Lyu		Ate	
	Size (bp)	Number	Size (bp)	Number
N90	302	106,384	299	100,427
N80	508	66,619	510	60,875
N70	831	42,772	866	37,808
N60	1,321	27,959	1,429	24,023
N50	1,975	18,407	2,221	15,450
Longest	36,130	-	35,264	-
Average length	905	-	931	-
Total size	154,017,925	-	152,264,687	-
Total number (≥1kb)	-	36,146	-	33,207
Total number (≥2kb)	-	18,140	-	17,304

Table S35. The assessment of the completeness of *de novo* transcriptome assemblies in *L. yunnana* (Lyu) and *A. terminalis* (Ate) by Benchmarking Universal Sing-Copy Orthologs (BUSCO) with Insecta_odb9 Dataset.

	Lyu		Ate	
	Numbers	Ratio (%)	Numbers	Ratio (%)
Complete BUSCOs (C)	1595	96.2	1577	95.1
Complete and single-copy BUSCOs (S)	1279	77.1	1063	64.1
Complete and duplicated BUSCOs (D)	316	19.1	514	31.0
Fragmented BUSCOs (F)	40	2.4	35	2.1
Missing BUSCOs (M)	23	1.4	46	2.8
Total BUSCO groups searched	1658	-	1658	-

Table S36. Interspecies comparison of genes expressed in luminous organs of *L. yunnana* (Lyu) and *A. terminalis* (Ate). FO: luminous organ of female adult; MO: luminous organ of male adult.

	FO	MO	Overlap of FO-MO
Lyu_up	586	575	388
Ate_up	658	612	413
Total	1244	1187	801

Table S37. The genes belonging to both differentially expressed genes (DEGs) and high expression genes (HEGs) in the luminous organs of *L. yunnanna* (Lyu) and *A. terminalis* (Ate). FO: luminous organ of female adult, MO: luminous organ of male adult.

Sepcies	Gene_ID	FPKM		Function
		FO	MO	
Lyu	LY05976	5058	3060	myosin regulatory light chain 2; EF-hand domain; EF-Hand 1, calcium-binding site
	LY06682	2242	953	60S ribosomal protein L35; Ribosomal protein L29 domain
Ate	LT09717	1612	827	6-pyruvoyl tetrahydrobiopterin synthase
	LT01236	4614	1574	Chitinase-like protein Idgf4; chitinase; Glycoside hydrolase superfamily
	LT03196	241	508	ATP synthase subunit gamma, mitochondrial; F-type H+-transporting ATPase subunit gamma; ATPase, F1 complex, gamma subunit
	LT08366	2012	768	uncharacterized protein
	LT17298	6621	380	Defensin, invertebrate/fungal; Knottin, scorpion toxin-like
	LT17299	11106	371	Knottin, scorpion toxin-like

Table S38. Summary on samples used for proteomic sequencing of *L. yunnanna* (Lyu) and *A. terminalis* (Ate). FO: luminous organ of female adult, MO: luminous organ of male adult.

Specimen	Sex	Repeat	Individuals
Lyu-MO	male	Lyu-MO_bio1	6
		Lyu-MO_bio2	6
		Lyu-MO_bio3	6
Lyu-FO	female	Lyu-FO_bio1	6
		Lyu-FO_bio2	6
		Lyu-FO_bio3	6
Ate-MO	male	Ate-MO_bio1	40
		Ate-MO_bio2	40
		Ate-MO_bio3	40
Ate-FO	female	Ate-FO_bio1	70
		Ate-FO_bio2	70
		Ate-FO_bio3	70

Table S39. The number of identified proteins expressed in the luminous organs of *L. yunnanna* (Lyu) and *A. terminalis* (Ate).

Species	Spectrum	Peptides	Proteins
Lyu	194272	18257	1933
Ate	264253	19825	2177

Table S40. The GO enrichment of high abundance protein (HAP) genes in both female and male luminous organs for both *L. yunnanna* and *A. terminalis*.

GO	GO_Type	GO_term	P value
GO:0046034	biological_process	ATP metabolic process	5.90E-07
GO:0015992	biological_process	proton transport	4.02E-06
GO:0006108	biological_process	malate metabolic process	2.67E-05
GO:0015991	biological_process	ATP hydrolysis coupled proton transport	7.56E-05
GO:0019752	biological_process	carboxylic acid metabolic process	5.29E-03
GO:0006869	biological_process	lipid transport	7.32E-03
GO:0006096	biological_process	glycolytic process	1.83E-02
GO:0015986	biological_process	ATP synthesis coupled proton transport	1.83E-02
GO:0000103	biological_process	sulfate assimilation	1.96E-02
GO:0006084	biological_process	acetyl-CoA metabolic process	1.96E-02
GO:0042026	biological_process	protein refolding	1.96E-02
GO:0043401	biological_process	steroid hormone mediated signaling pathway	1.96E-02
GO:0006457	biological_process	protein folding	2.48E-02
GO:0006281	biological_process	DNA repair	3.88E-02
GO:0006820	biological_process	anion transport	3.88E-02
GO:0008218	biological_process	bioluminescence	3.88E-02
GO:0044070	biological_process	regulation of anion transport	3.88E-02
GO:0033178	cellular_component	proton-transporting two-sector ATPase complex, catalytic domain	1.74E-06
GO:0033180	cellular_component	proton-transporting V-type ATPase, V1 domain	2.17E-03
GO:0000015	cellular_component	phosphopyruvate hydratase complex	1.96E-02
GO:0008091	cellular_component	spectrin	1.96E-02
GO:0016820	molecular_function	hydrolase activity, acting on acid anhydrides, catalyzing transmembrane movement of substances	4.02E-06
GO:0005524	molecular_function	ATP binding	2.36E-04
GO:0016615	molecular_function	malate dehydrogenase activity	3.70E-04
GO:0030060	molecular_function	L-malate dehydrogenase activity	3.70E-04
GO:0008289	molecular_function	lipid binding	5.29E-03
GO:0046933	molecular_function	proton-transporting ATP synthase activity, rotational mechanism	5.29E-03
GO:0046961	molecular_function	proton-transporting ATPase activity, rotational mechanism	7.32E-03
GO:0016616	molecular_function	oxidoreductase activity, acting on the CH-OH group of donors, NAD	1.83E-02

		or NADP as acceptor	
GO:0003824	molecular_function	catalytic activity	1.92E-02
GO:0003707	molecular_function	steroid hormone receptor activity	1.96E-02
GO:0004020	molecular_function	adenylylsulfate kinase activity	1.96E-02
GO:0004096	molecular_function	catalase activity	1.96E-02
GO:0004634	molecular_function	phosphopyruvate hydratase activity	1.96E-02
GO:0004781	molecular_function	sulfate adenylyltransferase (ATP) activity	1.96E-02
GO:0005200	molecular_function	structural constituent of cytoskeleton	2.91E-02
GO:0051287	molecular_function	NAD binding	2.91E-02
GO:0004332	molecular_function	fructose-bisphosphate aldolase activity	3.88E-02
GO:0004470	molecular_function	malic enzyme activity	3.88E-02
GO:0004471	molecular_function	malate dehydrogenase (decarboxylating) (NAD+) activity	3.88E-02
GO:0005543	molecular_function	phospholipid binding	3.88E-02
GO:0008308	molecular_function	voltage-gated anion channel activity	3.88E-02

Table S41. The statistics of quantitative identified protein in *L. yunnanna* (Lyu) and *A. terminalis* (Ate). FO: luminous organ of female adult; MO: luminous organ of male adult.

Type	Lyu	Lyu-FO/Lyu-MO	Ate	Ate-FO/Ate-MO	Lyu-Ate	Ate-FO/Lyu-FO	Ate-MO/Lyu-MO
Quant gene number(orthologs)	1933	1279	2177	1516	1151	981	935
Quant gene number(orthologs) (>0)*	1279	1279	1516	1516	804	981	935
DAPs number	-	165	-	204	-	210	299
Up number	-	71	-	123	-	107	140
Down number	-	94	-	81	-	103	159

* The protein abundance of genes more than 0 in both female and male luminous organs.

Table S42. The KEGG enrichment of expressed genes in both female and male luminous organs of both *L. yunnanna* and *A. terminalis* at proteomic level (P<0.05).

KEGG_A_class	KEGG_B_class	Pathway ID	Pathway	Pvalue
Cellular Processes	Transport and catabolism	ko04145	Phagosome	9.13E-04
Cellular Processes	Transport and catabolism	ko04142	Lysosome	7.38E-03
Cellular Processes	Transport and catabolism	ko04144	Endocytosis	4.34E-02
Genetic Information Processing	Folding, sorting and degradation	ko03050	Proteasome	1.37E-16
Genetic Information Processing	Folding, sorting and degradation	ko04141	Protein processing in endoplasmic reticulum	1.08E-05
Genetic Information Processing	Translation	ko03010	Ribosome	1.32E-04
Metabolism	Global and Overview	ko01200	Carbon metabolism	7.66E-18
Metabolism	Energy metabolism	ko00190	Oxidative phosphorylation	1.86E-17
Metabolism	Global and Overview	ko01230	Biosynthesis of amino acids	3.94E-10
Metabolism	Carbohydrate metabolism	ko00020	Citrate cycle (TCA cycle)	1.27E-07
Metabolism	Carbohydrate metabolism	ko00010	Glycolysis / Gluconeogenesis	3.26E-07
Metabolism	Energy metabolism	ko00710	Carbon fixation in photosynthetic organisms	6.04E-07
Metabolism	Amino acid metabolism	ko00280	Valine, leucine and isoleucine degradation	1.10E-05
Metabolism	Carbohydrate metabolism	ko00051	Fructose and mannose metabolism	7.78E-05
Metabolism	Lipid metabolism	ko00071	Fatty acid degradation	1.04E-04
Metabolism	Carbohydrate metabolism	ko00030	Pentose phosphate pathway	1.73E-04
Metabolism	Global and Overview	ko01210	2-Oxocarboxylic acid metabolism	8.39E-04
Metabolism	Energy metabolism	ko00680	Methane metabolism	8.47E-04
Metabolism	Amino acid metabolism	ko00310	Lysine degradation	9.41E-04
Metabolism	Amino acid metabolism	ko00270	Cysteine and methionine metabolism	9.70E-04
Metabolism	Global and Overview	ko01212	Fatty acid metabolism	1.62E-03
Metabolism	Carbohydrate metabolism	ko00620	Pyruvate metabolism	1.63E-03

Metabolism	Metabolism of cofactors and vitamins	ko00740	Riboflavin metabolism	1.67E-03
Metabolism	Lipid metabolism	ko00120	Primary bile acid biosynthesis	2.38E-03
Metabolism	Carbohydrate metabolism	ko00640	Propanoate metabolism	3.12E-03
Metabolism	Energy metabolism	ko00920	Sulfur metabolism	3.81E-03
Metabolism	Carbohydrate metabolism	ko00630	Glyoxylate and dicarboxylate metabolism	8.84E-03
Metabolism	Metabolism of cofactors and vitamins	ko00670	One carbon pool by folate	9.59E-03
Metabolism	Biosynthesis of other secondary metabolites	ko00950	Isoquinoline alkaloid biosynthesis	9.72E-03
Metabolism	Amino acid metabolism	ko00250	Alanine, aspartate and glutamate metabolism	1.07E-02
Metabolism	Metabolism of other amino acids	ko00410	beta-Alanine metabolism	1.69E-02
Metabolism	Amino acid metabolism	ko00350	Tyrosine metabolism	2.01E-02
Metabolism	Metabolism of other amino acids	ko00480	Glutathione metabolism	2.63E-02
Metabolism	Xenobiotics biodegradation and metabolism	ko00643	Styrene degradation	3.49E-02
Metabolism	Biosynthesis of other secondary metabolites	ko00965	Betalain biosynthesis	3.49E-02
Metabolism	Carbohydrate metabolism	ko00650	Butanoate metabolism	4.07E-02
Metabolism	Lipid metabolism	ko00062	Fatty acid elongation	4.07E-02
Metabolism	Amino acid metabolism	ko00220	Arginine biosynthesis	4.15E-02
Metabolism	Biosynthesis of other secondary metabolites	ko00960	Tropane, piperidine and pyridine alkaloid biosynthesis	4.89E-02
Organismal Systems	Excretory system	ko04966	Collecting duct acid secretion	1.96E-06
Organismal Systems	Nervous system	ko04721	Synaptic vesicle cycle	1.04E-05
Organismal Systems	Immune system	ko04612	Antigen processing and presentation	5.54E-04
Organismal Systems	Excretory system	ko04964	Proximal tubule bicarbonate reclamation	4.16E-03
Organismal Systems	Circulatory system	ko04260	Cardiac muscle contraction	1.01E-02
Organismal Systems	Excretory system	ko04961	Endocrine and other factor-regulated calcium reabsorption	3.06E-02

Table S43. The KEGG enrichment of different abundance protein genes (DAPs) of the comparison of female luminous organs (FO) and comparison of male luminous organs (MO) between *L. yunnanna* and *A. terminalis* (P < 0.05).

Comparison group	KEGG_A_class	KEGG_B_class	Pathway ID	Pathway	P value
FO	Metabolism	Global and Overview	ko01230	Biosynthesis of amino acids	1.84E-06
	Metabolism	Carbohydrate metabolism	ko00030	Pentose phosphate pathway	9.77E-05
	Metabolism	Energy metabolism	ko00710	Carbon fixation in photosynthetic organisms	1.67E-04
	Metabolism	Carbohydrate metabolism	ko00051	Fructose and mannose metabolism	2.00E-04
	Metabolism	Carbohydrate metabolism	ko00010	Glycolysis / Gluconeogenesis	3.14E-04
	Metabolism	Amino acid metabolism	ko00250	Alanine, aspartate and glutamate metabolism	4.59E-04
	Metabolism	Global and Overview	ko01200	Carbon metabolism	8.26E-04
	Metabolism	Amino acid metabolism	ko00220	Arginine biosynthesis	1.02E-03
	Metabolism	Amino acid metabolism	ko00270	Cysteine and methionine metabolism	1.22E-03
	Environmental Information Processing	Signal transduction	ko04066	HIF-1 signaling pathway	1.23E-02
	Metabolism	Lipid metabolism	ko00561	Glycerolipid metabolism	2.26E-02
	Organismal Systems	Endocrine system	ko04922	Glucagon signaling pathway	2.34E-02
	Metabolism	Amino acid metabolism	ko00400	Phenylalanine, tyrosine and tryptophan biosynthesis	3.37E-02
	Metabolism	Biosynthesis of other	ko00960	Tropane, piperidine and pyridine	3.37E-02

		secondary metabolites		alkaloid biosynthesis	
	Metabolism	Global and Overview	ko01210	2-Oxocarboxylic acid metabolism	3.54E-02
	Metabolism	Carbohydrate metabolism	ko00040	Pentose and glucuronate interconversions	4.11E-02
	Metabolism	Amino acid metabolism	ko00360	Phenylalanine metabolism	4.20E-02
	Metabolism	Metabolism of other amino acids	ko00450	Selenocompound metabolism	4.59E-02
	Metabolism	Biosynthesis of other secondary metabolites	ko00950	Isoquinoline alkaloid biosynthesis	4.59E-02
	Metabolism	Energy metabolism	ko00920	Sulfur metabolism	4.59E-02
MO	Metabolism	Energy metabolism	ko00190	Oxidative phosphorylation	5.00E-04
	Organismal Systems	Circulatory system	ko04260	Cardiac muscle contraction	8.10E-04
	Metabolism	Global and Overview	ko01200	Carbon metabolism	8.74E-04
	Metabolism	Global and Overview	ko01210	2-Oxocarboxylic acid metabolism	2.28E-03
	Organismal Systems	Excretory system	ko04966	Collecting duct acid secretion	2.43E-03
	Metabolism	Carbohydrate metabolism	ko00051	Fructose and mannose metabolism	6.33E-03
	Metabolism	Global and Overview	ko01230	Biosynthesis of amino acids	7.51E-03
	Organismal Systems	Nervous system	ko04721	Synaptic vesicle cycle	8.12E-03
	Metabolism	Carbohydrate metabolism	ko00020	Citrate cycle (TCA cycle)	1.32E-02
	Metabolism	Carbohydrate metabolism	ko00010	Glycolysis / Gluconeogenesis	1.70E-02
	Metabolism	Xenobiotics biodegradation and	ko00362	Benzoate degradation	2.10E-02

	metabolism			
Metabolism	Energy metabolism	ko00720	Carbon fixation pathways in prokaryotes	2.25E-02
Organismal Systems	Circulatory system	ko04261	Adrenergic signaling in cardiomyocytes	4.02E-02
Metabolism	Carbohydrate metabolism	ko00500	Starch and sucrose metabolism	4.02E-02

Table S44. The GO enrichment of the genes belongint to both different abundance proteins (DAPs) and differentially expressed genes (DEGs) in the luminous organs of *L. yunnanna* and *A. terminalis*. FO: luminous organ of female adult; MO: luminous organ of male adult.

Overlap groups	GO	GO_Type	GO_term	P value
FO_DAPs_DEGs	GO:0006535	biological_process	cysteine biosynthetic process from serine	1.50E-02
	GO:0019343	biological_process	cysteine biosynthetic process via cystathionine	1.50E-02
	GO:0046168	biological_process	glycerol-3-phosphate catabolic process	1.50E-02
	GO:0006810	biological_process	transport	2.49E-02
	GO:0006072	biological_process	glycerol-3-phosphate metabolic process	2.97E-02
	GO:0005978	biological_process	glycogen biosynthetic process	4.43E-02
	GO:0009331	cellular_component	glycerol-3-phosphate dehydrogenase complex	2.97E-02
	GO:0004122	molecular_function	cystathionine beta-synthase activity	1.50E-02
	GO:0004367	molecular_function	glycerol-3-phosphate dehydrogenase [NAD+] activity	1.50E-02
	GO:0004373	molecular_function	glycogen (starch) synthase activity	1.50E-02
	GO:0004563	molecular_function	beta-N-acetylhexosaminidase activity	1.50E-02
	GO:0008430	molecular_function	selenium binding	1.50E-02
	GO:0005549	molecular_function	odorant binding	2.49E-02
	GO:0004332	molecular_function	fructose-bisphosphate aldolase activity	2.97E-02
	GO:0019205	molecular_function	nucleobase-containing compound kinase activity	2.97E-02
	GO:0052689	molecular_function	carboxylic ester hydrolase activity	2.97E-02
	GO:0042803	molecular_function	protein homodimerization activity	4.43E-02
MO_DAPs_DEGs	GO:0006869	biological_process	lipid transport	1.37E-04
	GO:0006729	biological_process	tetrahydrobiopterin biosynthetic process	4.03E-02
	GO:0006779	biological_process	porphyrin-containing compound biosynthetic process	4.03E-02

GO:0019509	biological_process	L-methionine salvage from methylthioadenosine	4.03E-02
GO:0005576	cellular_component	extracellular region	2.37E-04
GO:0008289	molecular_function	lipid binding	2.29E-03
GO:0005319	molecular_function	lipid transporter activity	3.78E-03
GO:0003874	molecular_function	6-pyruvoyltetrahydropterin synthase activity	2.03E-02
GO:0004104	molecular_function	cholinesterase activity	2.03E-02
GO:0004373	molecular_function	glycogen (starch) synthase activity	2.03E-02
GO:0004853	molecular_function	uroporphyrinogen decarboxylase activity	2.03E-02
GO:0016844	molecular_function	strictosidine synthase activity	2.03E-02
GO:0043874	molecular_function	acireductone synthase activity	2.03E-02
GO:0042302	molecular_function	structural constituent of cuticle	3.51E-02

Table S45. Seven genes belonging to both different abundance proteins (DAPs) and differentially expressed genes (DEGs) at protein abundance and transcriptomic levels in *L. yunnanna* (Lyu) and *A. terminalis* (Ate). FO: luminous organ of female adult; MO: luminous organ of male adult.

**ID**		**Ate-FO/Lyu-FO**				**Ate-MO/Lyu-MO**			
		**Proteome**		**Transcriptome**		**Proteome**		**Transcriptome**	
Ate_id	Lyu_id	logFC	diff_stat	logFC	diff_stat	logFC	diff_stat	logFC	diff_stat
LT00651	LY00572	2.21	up	11.08	up	1.08	up	8.35	up
LT18522	LY04558	1.5	up	13.88	up	0.91	up	14.43	up
LT18952	LY16313	-1.14	down	-5.38	down	-1.12	down	-9.02	down
LT08366	LY08852	-0.37	down	15.59	up	-0.82	down	14.15	up
LT00926	LY11580	-0.91	down	12.66	up	-1.08	down	12.58	up
LT12212	LY08627	-2.22	down	-6.42	down	-1.72	down	-6.76	down
LT13566	LY05682	0.51	up	5.50	up	0.49	up	6.16	up

Table S46. The GO enrichment of high expression genes shared in the luminous organs of *L. yunnanna* and *A. terminalis* at both transcriptomic and proteomic levels.

GO	GO_Type	GO_term	P value
GO:0046034	biological_process	ATP metabolic process	3.36E-07
GO:0015992	biological_process	proton transport	4.87E-06
GO:0006108	biological_process	malate metabolic process	5.86E-05
GO:0015991	biological_process	ATP hydrolysis coupled proton transport	1.59E-03
GO:0019752	biological_process	carboxylic acid metabolic process	3.67E-03
GO:0015986	biological_process	ATP synthesis coupled proton transport	1.23E-02
GO:0000103	biological_process	sulfate assimilation	2.57E-02
GO:0006084	biological_process	acetyl-CoA metabolic process	2.57E-02
GO:0006820	biological_process	anion transport	2.57E-02
GO:0008218	biological_process	bioluminescence	2.57E-02
GO:0044070	biological_process	regulation of anion transport	2.57E-02
GO:1902017	biological_process	regulation of cilium assembly	2.57E-02
GO:0033178	cellular_component	proton-transporting two-sector ATPase complex, catalytic domain	1.44E-04
GO:0000015	cellular_component	phosphopyruvate hydratase complex	2.57E-02
GO:0016820	molecular_function	hydrolase activity, acting on acid anhydrides, catalyzing transmembrane movement of substances	2.83E-04
GO:0016615	molecular_function	malate dehydrogenase activity	6.32E-04
GO:0030060	molecular_function	L-malate dehydrogenase activity	6.32E-04
GO:0005524	molecular_function	ATP binding	1.02E-03
GO:0008289	molecular_function	lipid binding	3.67E-03
GO:0046933	molecular_function	proton-transporting ATP synthase activity, rotational mechanism	3.67E-03
GO:0046961	molecular_function	proton-transporting ATPase activity, rotational mechanism	8.90E-03

GO:0016616	molecular_function	oxidoreductase activity, acting on the CH-OH group of donors, NAD or NADP as acceptor	2.04E-02
GO:0004020	molecular_function	adenylylsulfate kinase activity	2.57E-02
GO:0004096	molecular_function	catalase activity	2.57E-02
GO:0004634	molecular_function	phosphopyruvate hydratase activity	2.57E-02
GO:0004781	molecular_function	sulfate adenylyltransferase (ATP) activity	2.57E-02
GO:0008308	molecular_function	voltage-gated anion channel activity	2.57E-02
GO:0003824	molecular_function	catalytic activity	2.78E-02
GO:0051287	molecular_function	NAD binding	3.56E-02

Table S47. The gene number of sulfatase (SULF) subgroups in the genomes of 11 beetles, silkworm and fruit fly.

Order	Family	Species	Sulf-Group-A	Sulf-Group-B	Sulf-Group-C	Sulf-Group-D	Sulf-Group-E	Sulf_sum
Coleoptera	Lampyridae	*Lamprigera yunnana*	4	0	1	1	2	8
Coleoptera	Lampyridae	*Abscondita terminalis*	3	2	1	1	3	10
Coleoptera	Lampyridae	*Aquatica lateralis*	4	0	1	1	2	8
Coleoptera	Lampyridae	*Photinus pyralis*	7	0	1	1	3	12
Coleoptera	Lampyridae	*Pyrocoelia pectoralis*	9	1	1	3	1	15
Coleoptera	Elateridae	*Ignelater luminosus*	9	0	1	1	2	13
Coleoptera	Buprestidae	*Agrilus planipennis*	6	0	1	1	2	10
Coleoptera	Scarabaeidae	*Onthophagus taurus*	6	0	1	1	5	13
Coleoptera	Cerambycidae	*Anoplophora glabripennis*	6	0	1	1	6	14
Coleoptera	Curculionidae	*Dendroctonus ponderosae*	4	0	1	1	2	8
Coleoptera	Tenebrionidae	*Tribolium castaneum*	6	0	1	1	2	10
Lepidoptera	Bombycidae	*Bombyx mori*	5	0	1	1	4	11
Diptera	Drosophilidae	*Drosophila melanogaster*	4	0	1	1	3	9

Table S48. The statistics of genes containing peroxisomal targeting signal (PTS) in the genomes of 11 beetles and fruit fly.

**Order**	**Family**	**Species**	**PTS1***	**PTS2***	**PTS1 and PTS2**	**mPTS-I (PMP) ***	**PTS1 and mPTS-I**	**PTS2 and mPTS-I**	**Total (%†)**
Coleoptera	Lampyridae	*Lamprigera yunnana*	1042	149	23	110	3	0	1275 (6.56)
Coleoptera	Lampyridae	*Abscondita terminalis*	1152	127	33	188	8	0	1424 (6.77)
Coleoptera	Lampyridae	*Aquatica lateralis*	854	90	26	117	5	1	1029 (7.20)
Coleoptera	Lampyridae	*Pyrocoelia pectoralis*	1304	169	38	154	5	0	1584 (6.86)
Coleoptera	Lampyridae	*Photinus pyralis*	899	98	25	113	6	0	1079 (6.84)
Coleoptera	Elateridae	*Ignelater luminosus*	1382	182	49	105	4	1	1615 (5.86)
Coleoptera	Cerambycidae	*Anoplophora glabripennis*	1175	141	32	102	7	0	1379 (6.26)
Coleoptera	Buprestidae	*Agrilus planipenni*	578	65	17	77	4	0	699 (3.16)
Coleoptera	Curculionidae	*Dendroctonus ponderosae*	592	92	15	104	5	0	768 (5.87)
Coleoptera	Scarabaeidae	*Onthophagus taurus*	940	101	27	90	6	1	1097 (5.06)
Coleoptera	Tenebrionidae	*Tribolium castaneum*	919	104	26	112	4	0	1105 (6.74)
Diptera	Drosophilidae	*Drosophila melanogaster*	735	78	23	84	5	0	868 (4.98)

* PTS1, the C-terminal peroxisomal targeting signal 1; PTS2, the N-terminal peroxisomal targeting signal 2; mPTS-I, the type I of targeting signals for the peroxisomal membrane proteins; † shows the percentage of PTS genes in whole genome genes.

Table S49. The target sites and primers used in *Abd-B* gene editing of *A. terminalis*.

Gene	Exon	Target No.	Target sites (GGN18)	Strand	PAM	Sanger verification	Primer name	Primers
LT07795 (*Abd-B*)	2nd	LT07795_T22053	GGGTGTACCACCACCGGCTA	-	CGG	ok	LT07795_ex2-F1	CGATATTCTAAAAGAGATT
	2nd	LT07795_T22120	GGTGACATGAAGATACAGCG	-	GGG	ok	LT07795_ex2-F1	GACTAAATGTATCTGGTGTTG
	2nd	LT07795_T22341	GGACCCGAACCGGTTCCTAC	+	CGG	ok	-	-
	2nd	LT07795_T22349	GGGTAAGGGCCTGTAGGAAC	-	CGG	ok	-	-
	2nd	LT07795_T22355	GGTGGTGGGTAAGGGCCTGT	-	AGG	ok	-	-
	3rd	LT07795_T23987	GGGTGTAGGTGTACCGTGCG	+	GGG	ok	LT07795_ex3-F3	AATTTGTGTTGGTTTGATTCG
	3rd	LT07795_T23999	GGATTCGTCCCTGCCCCGCA	-	CGG	ok	LT07795_ex3-R3	ATGGTGATTAACTACATGGT
	3rd	LT07795_T24286	GGTGTGTCACATGATGCCCA	-	TGG	ok	-	-

Table S50. Mutagenesis induced by different combinations in *Abd-B* (LT07795) of *A. terminalis*.

Date	Treat No.	Combination	Injected final con. (ng/μl)	Injected eggs	Hatched: Phenotype	Sequenced No.	Mutation rate	Mutation types
2017	LT-Abd-B-I	LT07795_T22053/T22120/T22341/T22349/T22355; Cas9	800; 1000	59	NA: 3 (NA)	2	100%	Del; Mut
2017	LT-Abd-B-II	LT07795_T23987/T23999/T24286; Cas9	800; 1000	73	NA: 0 (NA)	4	0%	
2017	LT-Abd-B-III	LT07795_T23987/T23999/T24286; Cas9	600; 1000	130	NA: 0 (NA)	11	0%	
2017	LT-Abd-B-IV	LT07795_T23987/T23999/T24286; Cas9	400; 1000	35	NA: 0 (NA)	NA	0%	
2018	LT-Abd-B-V	LT07795_T22120/T22341/T22355; Cas9	1000; 1000	71	50: 50 (100%)	3	100%	Del; In; Mut:
2018	*WT(V)*			106	101			

NA: No statistics; Del: deletion; In: insertion; Mut: mutation.

Table S51. Mutation rate among different morphological mutants and mutated clones across different target sites.

Target site	Mutants		Mutation rate of target site *
	LT-III-1	LT-III-12	
T22120	100% (9/9)	100% (10/10)	100% (19/19)
T22341	100% (9/9)	100% (10/10)	100% (19/19)
T22355	100% (9/9)	100% (10/10)	100% (19/19)
**Mutation rate of mutants**	**100% (9/9)**	**100% (10/10)**	**100% (19/19)**

* Numbers in brackets denote positive clones/total clones.

Table S52. The GO enrichment of down-regulated genes in *A. terminalis* mutants (P<0.05).

GO ID	GO Type	GO_term	P value
GO:0006743	biological_process	ubiquinone metabolic process	4.13E-03
GO:0006744	biological_process	ubiquinone biosynthetic process	4.13E-03
GO:0042181	biological_process	ketone biosynthetic process	4.13E-03
GO:1901661	biological_process	quinone metabolic process	4.13E-03
GO:1901663	biological_process	quinone biosynthetic process	4.13E-03
GO:0042180	biological_process	cellular ketone metabolic process	8.05E-03
GO:0006733	biological_process	oxidoreduction coenzyme metabolic process	2.39E-02
GO:0009108	biological_process	coenzyme biosynthetic process	2.90E-02
GO:0051188	biological_process	cofactor biosynthetic process	3.88E-02
GO:0008218	biological_process	bioluminescence	2.59E-02
GO:0006629	biological_process	lipid metabolic process	1.82E-02
GO:0044710	biological_process	single-organism metabolic process	1.57E-02
GO:0044699	biological_process	single-organism process	3.26E-02
GO:0005576	cellular_component	extracellular region	5.33E-03
GO:0005615	cellular_component	extracellular space	1.99E-02
GO:0044421	cellular_component	extracellular region part	3.33E-02
GO:0019012	cellular_component	virion	4.58E-02
GO:0019028	cellular_component	viral capsid	4.58E-02
GO:0044423	cellular_component	virion part	4.58E-02
GO:0005549	molecular_function	odorant binding	4.58E-08
GO:0016491	molecular_function	oxidoreductase activity	3.71E-02
GO:0020037	molecular_function	heme binding	4.51E-02
GO:0046906	molecular_function	tetrapyrrole binding	4.58E-02
GO:0008169	molecular_function	C-methyltransferase activity	3.38E-03
GO:0008425	molecular_function	2-polyprenyl-6-methoxy-1,4-benzoquinone methyltransferase activity	3.38E-03
GO:0030580	molecular_function	quinone cofactor methyltransferase activity	3.38E-03
GO:0008757	molecular_function	S-adenosylmethionine-dependent methyltransferase activity	3.01E-02
GO:0008146	molecular_function	sulfotransferase activity	2.74E-02
GO:0016782	molecular_function	transferase activity, transferring sulfur-containing groups	3.59E-02
GO:0004565	molecular_function	beta-galactosidase activity	2.70E-02
GO:0015925	molecular_function	galactosidase activity	2.70E-02
GO:0008289	molecular_function	lipid binding	4.86E-02

Table S53. The KEGG enrichment of down-regulated genes in *A. terminalis* mutants (P<0.05).

KEGG_A_class	KEGG_B_class	Pathway ID	Pathway	P value
Metabolism	Metabolism of other amino acids	ko00410	beta-Alanine metabolism	1.60E-02
Metabolism	Amino acid metabolism	ko00330	Arginine and proline metabolism	3.22E-02
Metabolism	Amino acid metabolism	ko00360	Phenylalanine metabolism	4.20E-02
Metabolism	Amino acid metabolism	ko00350	Tyrosine metabolism	3.09E-02
Metabolism	Biosynthesis of other secondary metabolites	ko00965	Betalain biosynthesis	1.68E-03
Metabolism	Biosynthesis of other secondary metabolites	ko00950	Isoquinoline alkaloid biosynthesis	2.44E-03
Metabolism	Metabolism of cofactors and vitamins	ko00130	Ubiquinone and other terpenoid-quinone biosynthesis	5.28E-03
Metabolism	Lipid metabolism	ko01040	Biosynthesis of unsaturated fatty acids	9.74E-03

Table S54. Gene numbers of phosphoinositide-specific phospholipase c (PLC) families in different insect species.

Family	Species	PLCβ	PLCδ	PLCε	PLCγ	PLCη	PLCζ	Total
Lampyridae	*Lamprigera yunnana* (Lyu)	2	0	1	1	0	0	4
Lampyridae	*Abscondita terminalis* (Ate)	2	0	1	1	0	0	4
Lampyridae	*Aquatica lateralis* (Ala)	2	0	1	1	0	0	4
Lampyridae	*Photinus pyralis* (Ppy)	2	0	1	1	0	0	4
Lampyridae	*Pyrocoelia pectoralis* (Ppe)	3	0	2	1	0	0	6
Elateridae	*Ignelater luminosus* (Ilu)	2	0	1	2	0	0	5
Buprestidae	*Agrilus planipennis* (Apl)	2	0	1	1	0	0	4
Scarabaeidae	*Onthophagus taurus* (Ota)	1	0	1	2	0	0	4
Cerambycidae	*Anoplophora glabripennis* (Agl)	2	0	1	1	0	0	4
Curculionidae	*Dendroctonus ponderosae* (Dpo)	2	0	0	1	0	0	3
Tenebrionidae	*Tribolium castaneum* (Tca)	2	0	1	1	0	0	4
Bombycidae	*Bombyx mori* (Bmo)	2	0	0	1	0	0	3
Drosophilidae	*Drosophila melanogaster* (Dme)	2	0	0	1	0	0	3

Table S55. Gene numbers of transient receptor potential channels (TRP) in different insect species. TRPC (canonical), TRPV (vanilloid), TRPM (melastatin), TRPP (polycystin), TRPML (mucolipin), TRPA (ankyrin) and TRPN (NOMPC-like).

**Family**	**Species**	**TRPA**	**TRPC**	**TRPM**	**TRPML**	**TRPN**	**TRPP**	**TRPV**	**Total**
Lampyridae	*Lamprigera yunnana* (Lyu)	5	3	0	1	0	1	2	12
Lampyridae	*Abscondita terminalis* (Ate)	4	4	1	0	1	1	2	13
Lampyridae	*Aquatica lateralis* (Ala)	5	3	1	0	1	1	2	13
Lampyridae	*Photinus pyralis* (Ppy)	4	3	1	1	0	1	2	12
Lampyridae	*Pyrocoelia pectoralis* (Ppe)	8	3	2	1	1	0	3	18
Elateridae	*Ignelater luminosus* (Ilu)	7	3	2	1	0	1	2	16
Buprestidae	*Agrilus planipennis* (Apl)	4	3	1	0	1	0	0	9
Scarabaeidae	*Onthophagus taurus* (Ota)	5	5	1	1	1	2	2	17
Cerambycidae	*Anoplophora glabripennis* (Agl)	6	4	2	1	0	2	0	15
Curculionidae	*Dendroctonus ponderosae* (Dpo)	3	4	1	1	0	2	4	15
Tenebrionidae	*Tribolium castaneum* (Tca)	5	4	1	1	0	1	3	15
Bombycidae	*Bombyx mori* (Bmo)	5	3	1	1	1	0	2	13
Drosophilidae	*Drosophila melanogaster* (Dme)	4	3	1	2	1	4	2	17

Table S56. Gene numbers of P-type ATPase in different insect species.

Order	Family	Species	P-type ATPase *						
			P1B	P2A	P2B	P2C	P4	P5	Total
Coleoptera	Lampyridae	*Lamprigera yunnana* (Lyu)	2	1	1	2	4	3	13
Coleoptera	Lampyridae	*Abscondita terminalis* (Ate)	2	2	1	3	4	4	16
Coleoptera	Lampyridae	*Aquatica lateralis* (Ala)	1	2	1	2	4	4	14
Coleoptera	Lampyridae	*Photinus pyralis* (Ppy)	1	2	1	2	4	4	14
Coleoptera	Lampyridae	*Pyrocoelia pectoralis* (Ppe)	2	2	1	3	6	6	20
Coleoptera	Elateridae	*Ignelater luminosus* (Ilu)	1	1	1	3	4	5	15
Coleoptera	Buprestidae	*Agrilus planipennis* (Apl)	1	2	2	3	4	4	16
Coleoptera	Scarabaeidae	*Onthophagus taurus* (Ota)	1	2	1	3	3	5	15
Coleoptera	Cerambycidae	*Anoplophora glabripennis* (Agl)	1	2	1	3	4	4	15
Coleoptera	Curculionidae	*Dendroctonus ponderosae* (Dpo)	1	2	1	2	6	3	15
Coleoptera	Tenebrionidae	*Tribolium castaneum* (Tca)	1	2	1	4	4	4	16
Lepidoptera	Bombycidae	*Bombyx mori* (Bmo)	1	1	1	1	5	2	11
Diptera	Drosophilidae	*Drosophila melanogaster* (Dme)	1	2	1	2	6	2	14

* P1B (Cu^{+}, Ag^{+}, Cu^{2+}, Cd^{2+}, Zn^{2+}, Pb^{2+}, Co^{2+}), P2A (Ca^{2+}, Mn^{2+}; including SERCA pumps), P2B (Ca^{2+}; including plasma membrane Ca^{2+} ATPase (PMCA) pumps), P2C (Na^{+}/K^{+}; H^{+}/K^{+}), P4 (phospholipids ?), P5 (no assigned specifity).

Table S57. Gene numbers of sodium-calcium exchanger (NCX) in different insect species.

Order	Family	Species	NCX	NCKX	NCLX	Total
Coleoptera	Lampyridae	*Lamprigera yunnana* (Lyu)	1	3	0	4
Coleoptera	Lampyridae	*Abscondita terminalis* (Ate)	1	5	0	6
Coleoptera	Lampyridae	*Aquatica lateralis* (Ala)	2	6	0	8
Coleoptera	Lampyridae	*Photinus pyralis* (Ppy)	2	6	0	8
Coleoptera	Lampyridae	*Pyrocoelia pectoralis* (Ppe)	1	4	0	5
Coleoptera	Elateridae	*Ignelater luminosus* (Ilu)	1	6	0	7
Coleoptera	Buprestidae	*Agrilus planipennis* (Apl)	1	5	0	6
Coleoptera	Scarabaeidae	*Onthophagus taurus* (Ota)	1	5	0	6
Coleoptera	Cerambycidae	*Anoplophora glabripennis* (Agl)	1	4	0	5
Coleoptera	Curculionidae	*Dendroctonus ponderosae* (Dpo)	1	5	0	6
Coleoptera	Tenebrionidae	*Tribolium castaneum* (Tca)	1	5	0	6
Lepidoptera	Bombycidae	*Bombyx mori* (Bmo)	1	5	0	6
Diptera	Drosophilidae	*Drosophila melanogaster* (Dme)	1	5	0	6

NCX: Na^{+}/Ca^{2+} exchangers; NCKX: $Na^{+}/Ca^{2+}/K^{+}$ exchangers; NCLX: Ca^{2+}/Cation exchangers.

Table S58. Gene numbers of calcium uniporter in different insect species.

Order	Family	Species	Calcium uniporter *					
			MCU	MICU1	MICU2	MCUR1	EMRE	Total
Coleoptera	Lampyridae	*Lamprigera yunnana* (Lyu)	1	1	1	0	0	3
Coleoptera	Lampyridae	*Abscondita terminalis* (Ate)	1	1	1	0	0	3
Coleoptera	Lampyridae	*Aquatica lateralis* (Ala)	1	1	1	0	0	3
Coleoptera	Lampyridae	*Photinus pyralis* (Ppy)	1	1	1	0	0	3
Coleoptera	Lampyridae	*Pyrocoelia pectoralis* (Ppe)	2	2	1	0	1	6
Coleoptera	Elateridae	*Ignelater luminosus* (Ilu)	1	1	1	0	1	4
Coleoptera	Buprestidae	*Agrilus planipennis* (Apl)	1	1	1	0	0	3
Coleoptera	Scarabaeidae	*Onthophagus taurus* (Ota)	1	1	1	0	1	4
Coleoptera	Cerambycidae	*Anoplophora glabripennis* (Agl)	1	1	1	0	0	3
Coleoptera	Curculionidae	*Dendroctonus ponderosae* (Dpo)	1	1	1	0	1	4
Coleoptera	Tenebrionidae	*Tribolium castaneum* (Tca)	1	1	2	0	1	5
Lepidoptera	Bombycidae	*Bombyx mori* (Bmo)	0	2	0	0	1	3
Diptera	Drosophilidae	*Drosophila melanogaster* (Dme)	1	2	1	0	1	5

*MCU: mitochondrial calcium uniporter; MICU1: Mitochondrial Calcium Uptake 1; MICU2: Mitochondrial Calcium Uptake 2; EMRE: Essential MCU Regulator; MCUR1: Mitochondrial Calcium Uniporter Regulator 1.

Supplementary Videos

Video S1. Light organs of *L. yunnana* are glowing as a pair of spots on two sides of ventrite.

Video S2. Light organs of *L. yunnana* are glowing on two sides of ventrite.

Video S3. Two light organs of *A. terminalis* female are glowing in ventrite.

Video S4. A light organ of *A. terminalis* female is glowing in ventrite.
